# Transcriptome analysis reveals unique metabolic features in the *Cryptosporidium parvum* Oocysts associated with environmental survival and stresses

**DOI:** 10.1186/1471-2164-13-647

**Published:** 2012-11-21

**Authors:** Haili Zhang, Fengguang Guo, Huaijun Zhou, Guan Zhu

**Affiliations:** 1 Department of Veterinary Pathobiology, College of Veterinary Medicine & Biomedical Sciences, Texas A&M University, College Station, Texas, 77843, USA; 2 Faculty of Genetics Program, Texas A&M University, College Station, Texas, 77843, USA; 3 Institute of Genetics, College of Life Science, Zhejiang University, Hangzhou, 310058, China; 4 Department of Animal Science, University of California, Davis, CA, 95616, USA

**Keywords:** Apicomplexan, *Cryptosporidium parvum*, Oocysts, Agilent microarray, Transcriptome, Ultraviolent (UV) irradiation

## Abstract

**Background:**

*Cryptosporidium parvum* is a globally distributed zoonotic parasite and an important opportunistic pathogen in immunocompromised patients. Little is known on the metabolic dynamics of the parasite, and study is hampered by the lack of molecular and genetic tools. Here we report the development of the first Agilent microarray for *C*. *parvum* (CpArray15K) that covers all predicted ORFs in the parasite genome. Global transcriptome analysis using CpArray15K coupled with real-time qRT-PCR uncovered a number of unique metabolic features in oocysts, the infectious and environmental stage of the parasite.

**Results:**

Oocyst stage parasites were found to be highly active in protein synthesis, based on the high transcript levels of genes associated with ribosome biogenesis, transcription and translation. The proteasome and ubiquitin associated components were also highly active, implying that oocysts might employ protein degradation pathways to recycle amino acids in order to overcome the inability to synthesize amino acids de novo. Energy metabolism in oocysts was featured by the highest level of expression of lactate dehydrogenase (LDH) gene. We also studied parasite responses to UV-irradiation, and observed complex and dynamic regulations of gene expression. Notable changes included increased transcript levels of genes involved in DNA repair and intracellular trafficking. Among the stress-related genes, TCP-1 family members and some thioredoxin-associated genes appear to play more important roles in the recovery of UV-induced damages in the oocysts. Our observations also suggest that UV irradiation of oocysts results in increased activities in cytoskeletal rearrangement and intracellular membrane trafficking.

**Conclusions:**

CpArray15K is the first microarray chip developed for *C*. *parvum*, which provides the *Cryptosporidium* research community a needed tool to study the parasite transcriptome and functional genomics. CpArray15K has been successfully used in profiling the gene expressions in the parasite oocysts as well as their responses to UV-irradiation. These observations shed light on how the parasite oocysts might adapt and respond to the hostile external environment and associated stress such as UV irradiation.

## Background

*Cryptosporidium parvum* is a globally distributed zoonotic pathogen belonging to the phylum Apicomplexa that contains many important human and animal pathogens, such as *Plasmodium*, *Toxoplasma*, *Eimeria*, *Babesia* and *Theileria*. *Cryptosporidium* can cause severe watery diarrhea in humans and animals, and is an important opportunistic pathogen in immunocompromised individuals such as AIDS patients [1-5]. It is also listed as one of the Category B agents in the National Institutes of Health (NIH) biodefense program. Despite its importance to both human and animal heaths, research on *Cryptosporidium* is limited not only by the difficulties in manipulating the parasite, but also by the lack of tools for genetic study such as robust culture systems, transfection methods and parasite-specific microarrays.

*Cryptosporidium* is transmitted between humans and/or animals by the infectious oocysts that can survive in the natural environments for a substantial length of time. Each *C*. *parvum* oocyst contains four sporozoites enclosed within an oocyst wall structure that is highly resistant to disinfectants, including commonly used chlorine treatments [6]. Molecular and cellular studies on the oocyst stage have been largely devoted to understanding the composition of oocyst wall, such as the family of oocyst wall proteins (COWPs) [7-10], and little is known on the biochemical events of this parasite stage. Understanding the global gene expression profiles in oocysts would not only deepen our understanding on the biology and metabolic features of this opportunistic pathogen, but also provide new insight into how the oocysts survive without any nutrient supply in the field and withstand various stresses in the natural environments. For example, *C*. *parvum* lacks a citrate cycle and a cytochrome-based respiratory chain, thus relying solely on glycolysis and fermentation as an energy and carbon source [11,12]. It is therefore important to understand how the oocysts function in the oxygen-rich external environment, with metabolic machinery that is seemingly specialized for the anaerobic conditions of the host gut, as well as streamlined for nutrient scavenging. Oocysts need to survive in the external environment for months or years, where they might face a range of hostile environmental stresses. Determining the gene expression profile changes in oocysts in response ultraviolent (UV) irradiation will help to understand how the parasite responds to stress, with practical relevance to the UV exposure encountered in the external environment, as well as UV irradiation commonly used by humans to treat drinking and surface waters [13,14].

Here we report the development of an Agilent platform-based, *C*. *parvum*-specific microarray using 60-base oligonucleotides that represent all predicted protein-encoding genes in the parasite. Assays of transcript levels using this microarray permitted us for the first time to systematically study gene transcript profiles for a global view of unique metabolic features of parasite oocysts, as well response to a common stress UV irradiation.

## Results and discussion

### Quality of the CpArray15K chips and data

The newly developed CpArray15K is an Agilent microarray that contains 15,208 spots, spanning 10,009 unique probes, covering all predicted protein coding genes in the *C*. *parvum* genome. The array was used to profile global gene transcript levels in oocysts, with or without UV irradiation, as described in the Materials and Methods. Medians of signal and background intensities were acquired for subsequent analysis.

**Figure 1:**
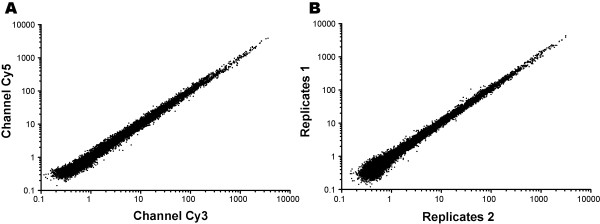
**Correlation plots of signal intensities (means of normalized median signals) between Cy3 and Cy5 dye swaps (R**^**2**^**=0**.**9879) (A) and biological replicates (R**^**2**^**=0**.**9886) (B)**. In panel **B**, data for replicates 1 and 2 were derived from 0.5 h and 5 h groups, each including two untreated controls and two UV-irradiation samples (oocysts) that were allowed to recover for 0.5 h or 5 h, respectively.

**Figure 2:**
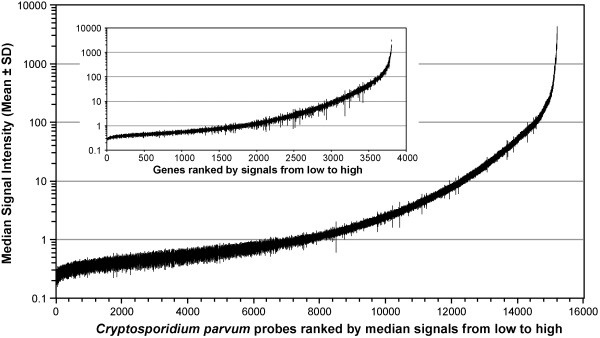
**Evaluation of inter-array variations by plotting of the mean signal intensities with standard deviations (SDs) of all individual probes among all 8 microarrays**, **and inter-probe variations by plotting the means and SDs of all individual genes from multiple probes (inset).**

**Figure 3:**
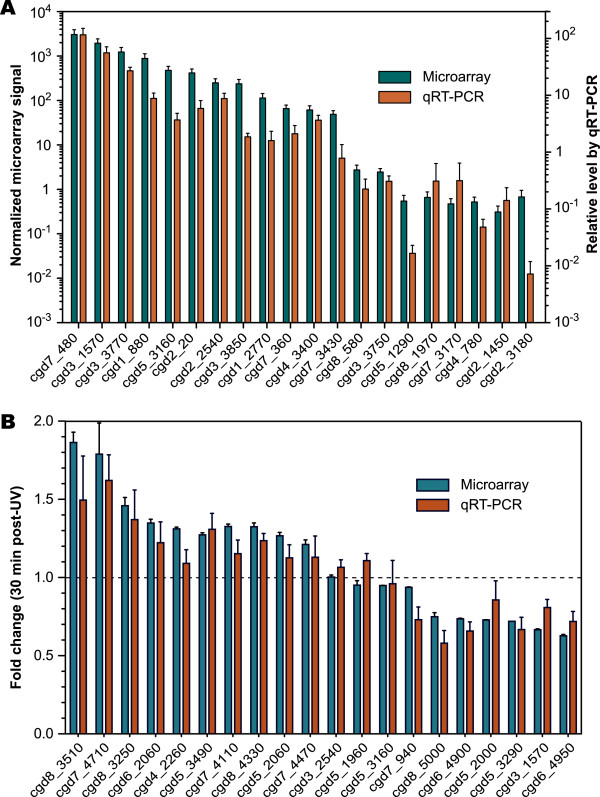
**Validation of microarray data by real-time qRT-PCR for selected *C. parvum *genes.** (**A**) Relative mRNA levels of *C*. *parvum* genes in the oocyst stage. Microarray signals are shown as the means of normalized signals derived from all probes in all 8 arrays (left axis). Levels determined by semi-qRT-PCR are displayed as the fold differences in relative to the mean of all samples in at least three biological replicates (right axis). (**B**) Fold changes of the gene expressions in *C*. *parvum* oocysts recovered for 30 min after UV-treatment. Bars indicate standard deviations (SDs).

Correlation plots of normalized signals from all 8 hybridized arrays suggested no or minimal bias between Cy3 and Cy5-labeled samples or between two biological replicates (Figure 1). We calculated the means and standard deviations (SDs) of all individual probes among the 8 arrays to evaluate the inter-array variations, as well as those for all individual genes from multiple probes to evaluate the inter-probe variations (Figure 2). Although a few of the inter-probe variations were relatively large, there were no contradictory results among different probes and arrays, confirming that both inter-array and inter-probe variations were minimal. We also validated the microarray data by real-time RT-PCR analysis on a panel of selected parasite genes, and observed a general agreement between microarray and qRT-PCR data, both in the relative level of expression in oocysts (Figure 3A) and in the fold changes in expressions and directions in response to UV-treatment (Figure 3B). Collectively, these observations confirm the overall quality of microarray design and the acquired signal data.

### Metabolic features in the *C*. *parvum* oocysts

The experimental design included determination of the gene expression profiles in untreated oocyst samples, as a means to understand metabolic features in the oocyst stage. To minimize the effect of temperature change on gene expression, parasite oocysts were removed from 4°C storage and placed overnight at room temperature. During this process they appeared to maintain their morphological integrity (Additional file 1: Figure S1). Since all oocysts had matured to contain 4 sporozoites, the gene expression levels in oocysts, as well as their fold changes in response to UV irradiation as described in later sections, represent metabolism of sporozoites within the oocysts.

From the total of 8 untreated samples, and based on the signal to noise ratio (SNR) >2.0 and signal to background ratio (SBR) >2.6 criterions [15], we have identified a total of 1,924 genes that were positively expressed in oocysts (i.e., Group I genes). This number represents 51% of the total 3,805 genes in the *C*. *parvum* genome. Among the remaining unexpressed genes, 1,213 genes had SNR ≤2.0 and SBR ≤2.6 in all probes and all arrays (Group II) that were considered as unexpressed or extremely lowly expressed in the oocysts. The remaining 668 genes (Group III) with positive signals in some, but not all, probes or arrays could be judged as lowly expressed, rather than unexpressed.

**Figure 4:**
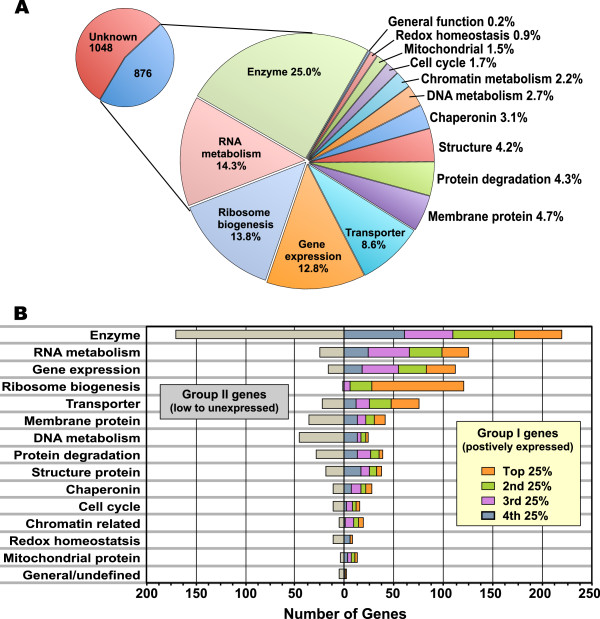
**Features of expressed genes in the *C***. ***parvum *oocysts** (**Group I genes**) **as determined by microarray analysis**. (**A**) Categorization of expressed Group I genes by major functional groups. (**B**) Functional categorization of Group I genes by expression levels (by quartiles) in comparison with the lowly or unexpressed Group II genes.

Based on the expression levels, we ranked the Group I genes by quartiles, and categorized them into major functional groups in comparison with unexpressed genes in Group II. Among the 1,924 Group I genes, only 876 genes (46%) were annotated and could be assigned into functional categories (Figure 4). The remaining 1,048 genes (54.46%) were hypothetical, including those with undefined functions, containing certain conserved domains, or possible membrane proteins. A complete list of all categorized genes in Groups I and II were provided as part of the supplementary materials (Additional file 2: Table S1). Additionally, we compiled a list of the top 10% (193) highest expressed genes with more detailed subcategories (Table 1).

**Table 1:** **Feature summary of the top 10**% highest expressed genes in the oocysts*

**Major Category(Genes #)**	**Feature Description**	**Representative Gene and description**		**Signal**
**Enzyme** (**20**)				
**Energy** (**3**)	Glycolytic enzymes	cgd7_480	Lactate dehydrogenase (LDH)	3,064.62
**Lipid** (**1**)	Membrane remodeling	cgd8_1150	Choline-phosphate cytidylyltransferase	185.50
**Nucleotide** (**4**)	Nucleotide metabolic enzymes	cgd5_1470	Nucleoside-diphosphate kinase domain	1,562.52
**Protein modification** (**9**)	Peptidase, kinase, phosphatase	cgd7_4790	Ptc7p phosphatase (PP2C family)	333.12
**Mitochondrial** (**2**)	lscU-like, F-ATPase headpiece	cgd2_1360	Mitochondrial ATP synthase β-chain	351.08
**Transporter** (**7**)	ABC, ion, nutrients, V-type	cgd3_510	Putative fucose translocator	398.70
**Stress**-**related** (**5**)				
**Chaperonine** (**3**)	DnaJ, HSP20, HSP90	cgd3_3770	HSP90	1,229.35
**Redox homeostasis** (**2**)	Glutaredoxin/thioredoxin related	cgd2_2540	Glutaredoxin related protein	250.42
**Cytoskeleton** (**3**)	Microfilament elements	cgd5_3160	Actin	475.92
**Membrane** (**7**)	All mucin-like proteins	cgd2_430	Mucin-like glycoprotein	1,732.54
**Cell cycle** (**1**)	Cyclin family protein	cgd7_3780	Cyclin	318.54
**Gene expression** (**14**)				
**Transcription** (**6**)	RNA Pol, transcription factors	cgd3_4150	Cutinase negative acting protein	315.25
**Translation** (**8**)	Translation factors, RNA helicases, etc.	cgd1_880	Eukaryotic initiation factor 4A	889.38
**Protein degradation** (**1**)	Proteosome/ubiquitin subunits	cgd1_420	20S proteasome beta subunit D2	168.66
**Ribosome biogenesis** (**38**)				
**Ribosomal proteins** (**32**)	RPL, RPS, or associated proteins	cgd3_2250	60S ribosomal protein L37A	303.99
**rRNA processing** (**6**)	GTPase, ribonucleoprotein, etc.	cgd4_1700	GNog1p. GTPase	276.15
**RNA metabolism** (**11**)	Methylase, Helicase, RNA-splicing, RNA-binding, etc.	cgd7_940	T22E16.120 SC35-like splicing factor	871.81
**DNA metabolism** (**2**)	Methytranferase, DNA-binding	cgd2_3070	HMG-box protein	425.65
**Unknown** (**82**)				
**Conserved domains** (**18**)	Conserved domain-containing	cgd2_200	Possible apicomplexan-specific protein	1,742.09
**Membrane** (**15**)	Predicted membrane proteins	cgd6_780	Predicted extracellular protein	849.61
**Unknown** (**49**)	Hypothetical/unknown	cgd3_1570	Unknown, possible sporozoite antigen	1,957.29

* A list of genes with detailed information on the normalized expression levels, their ranks, and categories is provided in Additional file 2, Table S1.

Approximately 41% of the annotated Group I genes were associated with gene expression functions, including those involved in core functions in expression (e.g., RNA synthesis, transcription and translation, 13% or 112 genes); ribosome biogenesis (14% or 122 genes); and RNA metabolism or modification (e.g., RNA helicases, RNA binding proteins, and factors involved in RNA degradation, splicing, methylation or polyadenylation, 17% or 150 genes) (Figure 4A). Among these genes, those involved in ribosome biogenesis, with a single exception, were expressed at top three quartiles and the majority of them (94 genes) were ranked at top quartile (Figure 4B). Another group of related genes were those associated with protein degradation, as indicated by the active expression of a large number of genes encoding proteosome subunits and ubiquitin pathway (i.e., 38 out of a total number of 67 genes), suggesting that proteins in the oocysts were actively recycled and suggesting a finite lifespan.

Genes encoding enzymes, excluding a few mitochondrial genes that were separately listed, constituted the second major group of expressed genes. Among them, 25% (or 219 genes) were expressed at varied levels, relatively evenly across the 4 quartiles (Figure 4B). A slightly less number of enzyme genes (170) were either lowly or unexpressed. Nearly half of the expressed enzymes were involved in post-translational modification of proteins (i.e., 98 out of the total 219 genes), including protein phosphatases, protein kinases, peptidases and protein folding elements. In contrast, DNA metabolism was much less active than the gene expression-associated pathways (Figure 4B). Among them, relatively more genes associated with the DNA repair than those involved in DNA replication were expressed, although none of them had the top quartile signals.

These observations indicate that *C*. *parvum* devotes significant resources to the gene expression and the synthesis, modification and degradation of proteins in the oocysts. Considering the inability of *Cryptosporidium* to synthesize any nutrients de novo, including amino acids [11,12,16], we speculate that the parasite relies heavily on protein degradation pathways to recycle amino acids. In this manner a protein synthetic capacity is maintained in environmental oocysts that have no access to the host cells to scavenge nutrients.

**Figure 5:**
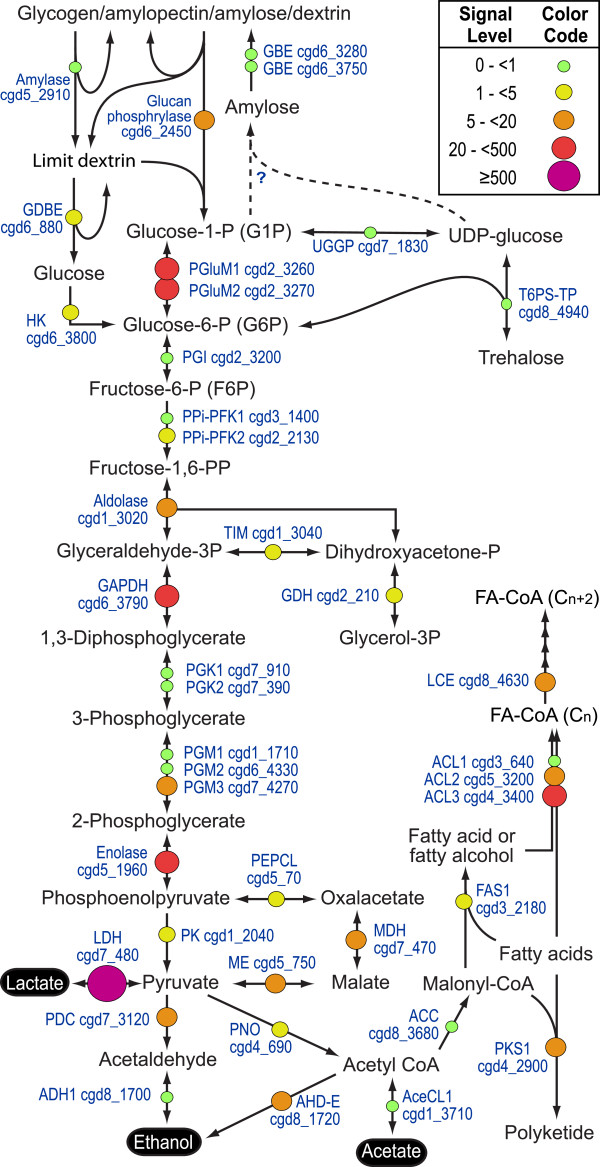
**Illustration of the expression levels of enzymes within the glycolytic pathway and major connections in the *C. parvum *oocysts as determined by microarray analysis.** The expression levels are grouped into 5 major groups by color and size. Abbreviations: ACC, acetyl-CoA carboxylase; AceACL, acetic acid-CoA ligase (aka acetyl-CoA synthetase); ACL, fatty acid-CoA ligase (aka acyl-CoA synthetase); ADH, alcohol dehydrogenase; ADH-E, type E alcohol dehydrogenase (bifunctional); FAS1, type I fatty acid synthase; GAPDH, glyceraldehyde phosphate dehydrogenase; GBE, glycogen branching enzyme; GDBE, glycogen debranching enzyme; GDH, glycerol phosphate dyhydrogenase; HK, hexokinase; LCE, long chain fatty acyl elongase; LDH, lactate dehydrogenase; MDH, malate dehydrogenase; ME, malic-enzyme; PDC, pyruvate decarboxylase; PEPCL, phosphoenolpyruvate carboxylase; PFK, phosphofructokinase; PGI, phosphoglucose isomerase; PGK, phosphoglycerate kinase; PGM, phosphoglycerate mutase; PGluM, phosphoglucose mutase; PK, pyruvate kinase; PKS1, type I polyketide synthase; PNO, pyruvate:NADP+ oxidoreductase; T6PS-TP, trehalose 6-phosphate synthase; TIM, triosephosphate isomerase; UGGP, UDP-galactose/glucose pyrophosphorylase.

In addition to protein modifications, various core metabolic pathways were active in oocysts. As expected, many enzymes involved in energy metabolism, such as glycolysis and fermentation, were expressed at relatively high levels (Figure 3, Figure 5 and Table 1). Oocysts appear to be able to use both amylopectin and hexoses as an energy and carbon source. Remarkably and highly unique, the lactate dehydrogenase (LDH) gene (cgd7_480) was expressed at the highest level among all genes (Figure 3, Figure 5 and Table 1).

**Figure 6:**
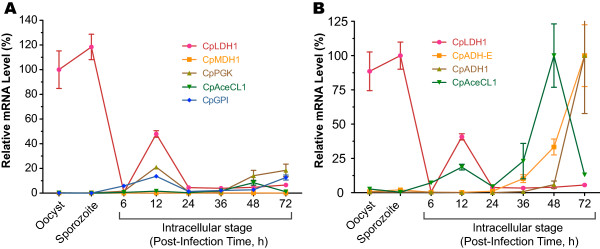
**Relative level of *CpLDH1 *gene expression in the oocysts, excystated sporozoites and various intracellular developmental stages in comparison with selected genes as determined by qRT**-**PCR.** (**A**) Comparison of *CpLDH1* expression with 4 other glycolytic genes. All levels are relative to that of *CpLDH1* in the oocysts. (**B**) Comparison of *CpLDH1* expression with three other genes responsible for producing different organic end products. In this panel, individual genes were separately calibrated, relative to the highest level within individual genes.

Because *C*. *parvum* lacks a citrate cycle and cytochrome-based respiratory chain, the parasite relies solely on the glycolysis as an energy and carbon source. To maintain the carbon flow it is capable of producing lactate, alcohol and acetate as organic end products. However, the expressions of other key enzymes for producing acetate (i.e., acetate-CoA ligase [AceCL1]) and alcohol (i.e., the bifunctional E-type alcohol dehydrogenase [ADH-E] and a monofunctional ADH [ADH1]) were much lower than that of LDH (Figure 3 and Additional file 2, Table S1). These observations prompted us to investigate the roles of the three end products in other parasite life cycle stages. Our qRT-PCR analysis of LDH, ADH-E, ADH1 and AceCL1 genes showed that the LDH gene was expressed at >100-fold higher levels in the oocysts and free sporozoites than in the intracellular developmental stages (Figure 6A). In contrast, the expression levels of genes encoding the acetate- and alcohol-producing enzymes were low in the oocysts and free sporozoites, but elevated in the intracellular stages (Figure 6B), suggesting that these genes played differentiated roles during the complex parasite life cycle.

Recently, Mauzy and colleagues have reported a comprehensive transcriptome analysis of all annotated genes in *C*. *parvum* during intracellular development by qRT-PCR, albeit the lack of data on the oocysts and free sprozoites [17]. Data extracted from their study on the expressions of LDH, ADH-E, ADH1 and AceCL genes during intracellular stages followed similar patterns as observed in this study; that is, peak expression at 12 h post-infection for LDH, but at 48–72 h for the other three genes (Additional file 3: Figure S2). *Cryptosporidium* LDH gene is unique among apicomplexan orthologs, as it is known to have evolved from the malate dehydrogenase (MDH) gene following gene duplication after the genus diverged from other apicomplexans [18]. Our data indicate that this unique gene plays a more important role in the energy metabolism in the parasite oocysts than in the intracellular stages.

As expected, oocysts were active in maintaining intracellular ion homeostasis and organellar pH, as well as subcellular transport of nutrients. Specifically, 9% (75) of the Group I genes encoded transporters that were expressed at varied levels; including ATP-binding cassette (ABC) family proteins, vacuolar (V-type) ATPases, various P-type ATPases responsible for ion transports and a subset of nutrient transporters (such as sugar transporters) (Figure 4, Table 1 and Additional file 2: Table S1).

Structure protein genes represented 4% of the expressed genes, which included mainly cytoskeletal proteins (n = 51) and five oocyst wall proteins, although most of them were expressed at low quartile levels. Among cytoskeletal proteins, actin expression was the highest, along with many other microfilamental proteins (Figure 4, Table 1 and Additional file 2: Table S1). In contrast, tubulins and other microtubule-associated proteins were mostly expressed at low to near background levels, which implies that the parasite oocysts are probably more active in maintaining intracellular trafficking than maintaining its structure.

The membrane protein group included mucins, TRAP and other thrombospondin domain-containing proteins [19-23], and proteins involved in membrane or vesicular sorting or trafficking. Other hypothetical proteins and enzymes could also be membrane-associated. Around 5% of the expressed genes belonged to this group (Figure 3 and Table 1). A subset of genes involved in membrane/vesicular sorting/transporting was expressed, indicating that the oocysts maintain certain level of membrane trafficking. The highest observed expression levels were among mucin-like proteins, particularly CpMuc5 (cgd2_430) that has been described as a marker in *C*. *parvum* infection [19-21]. The data suggest that mucin-like proteins are also important in the oocysts, possibly in maintaining and stabilizing the membrane structure under the hostile environment, or localization on the surface membrane of sporozoites in preparation to encounter the midgut environment and interaction with host cells.

Stress-related genes consists of various chaperonins/heat shock proteins (HSPs) and proteins for maintaining redox homeostasis. Among the 38 chaperon proteins of various classes, 27 were expressed. There were 4 major classes of proteins involved in cellular redox homeostasis (total 19 genes). Among them, all three glutaredoxin-associated genes and 5 (out of 13) thioredoxin-associated genes were expressed at various levels. However, signals for superoxide dismutases (SOD) and ferredoxin/ferredoxin reductase (Fdx/FdxR) were at or near background level, implying that in oocysts the SOD and the mitochondrial Fdx/FdxR pair were less important than the glutaredoxin and thioredoxin-associated pathways.

Among proteins predicted to reside in the relict mitochondria in *C*. *parvum*, a subset of genes encoding mitochondrial carrier proteins and translocases were active. Like the Fdx/FdxR pair, alternative oxidase (AOX) was not or lowly expressed. In contrast, the ion-Sulfur [FeS] cluster assembly pathway is active. It is known that *C*. *parvum* does not possess a complete F-ATPase, but only has two headpieces (i.e., α- and β-subunit) [16], for which their functional role remains to be defined. These two subunits were highly expressed in the oocysts (Table 1), suggesting that the standalone headpiece complex may play a role in the environmental oocyst stage.

A total of 1,048 hypothetical genes with undefined functions (vs. 876 functionally annotatable ones) were actively expressed at different levels in the oocysts (e.g., cgd3_1570, unknown, possible sporozoite antigen); as well as proteins containing apicomplexan-specific domains (e.g., cgd2_200), or predicted membrane proteins (e.g., cgd6_780) (Table 1 and Additional file 2: Table S1). Investigations on these genes may help to elucidate their functional roles in the survival and stress-resistance of parasite oocysts in the environment.

The environmental oocyst stage is also present in other intestinal and cyst-forming coccidia, such as *Eimeria tenella* and *Toxoplasma gondii*; although global gene expression profile data in the sporulated oocyst stage is at present only available for *T*. *gondii*[24]. Although the report mainly summarized the highly regulated *T*. *gondii* genes between tachyzoites, bradyzoites, and oocysts sporulated for various days [24], we were able to extract the expression data on individual developmental stages from ToxoDB ( http://www.ToxoDB.org) for a snapshot comparison with those in *C*. *parvum*. Among the top 100 highly expressed genes in fully sporulated *T*. *gondii* oocysts (i.e., sporulated for 10 days that is comparable with sporulated *C*. *parvum* oocysts), the majority (n = 52) were annotated as hypothetical. Among the remaining 48 genes, the largest group belonged to the secretory antigens/proteins (n = 18), including micronemal proteins (MIC), surface antigen 1-related sequences (SRS) and dense granule proteins (GRA), followed by enzymes (n = 10) (Additional file 4: Table S2), which differed from the expression profiles in *C*. *parvum* oocysts that was characterized by the high number of genes associated with ribosomal biogenesis, enzymes, and gene expression (Table 1). We also analyzed LDH gene expression profiles in *T*. *gondii*; namely, two LDH genes that are known to be differentially expressed in tachyzoites and bradyzoites [24]. The microarray data by Fritz and colleagues confirmed this finding, and also showed that the two LDH genes are differentially expressed in *T*. *gondii* oocysts. Specifically, the LDH1 gene (TGME49_032350) was expressed at much higher levels than the LDH2 gene (TGME49_091040) in oocysts with various sporulation times (Additional file 5, Figure S3). However, unlike the LDH gene in *C*. *parvum*, neither LDH1 nor LDH2 genes in *T*. *gondii* displayed extremely higher levels of expression in oocysts in comparison with either their expressions in other developmental stages or the expression of other genes in the oocysts (Additional file 5; Figure S3). Thus the difference in gene expression profiles in sporulated oocysts between *C*. *parvum* and *T*. *gondii* might reflect different metabolic features and adaptation of life cycle styles between these two apicomplexans.

### Gene expression in the oocysts in response to the UV irradiation

**Figure 7:**
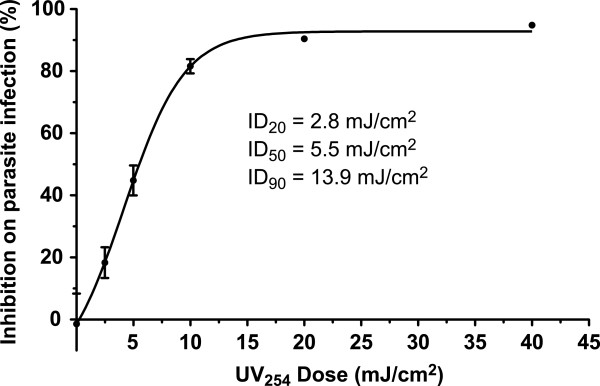
**Effect of UV**_**254 **_**irradiation on the *C. parvum *oocyst viability as determined by a qRT-PCR-based in vitro infection assay.** The ID_20_ value was determined at 2.8 mJ/cm^2^.

To identify an appropriate dose of UV treatment to study the global gene expression responses, we first determined the survival curve of *C*. *parvum* oocysts against increasing UV_254_ doses, as determined by a qRT-PCR-based assay of their ability to infect HCT-8 cells in vitro (Figure 7). We then selected the ID_20_ dose (2.8 mJ/cm^2^) that could introduce sufficient damages, but also allow the recovery of the majority of the irradiated oocysts. Both control and UV-treated oocysts were allowed to recover for 0.5 h and 5 h to represent relatively short and long post-irradiation recovery times.

Our microarray analysis identified 2,770 and 1,409 probes in the 0.5 h and 5 h groups, respectively, that were statistically significantly changed in expressions (*p* ≤ 0.05), which gave a total number of 3,659 probes (1,855 genes) that were statistically significantly changed in one or both groups (Table 2, also see Additional file 6: Table S3 for a complete list). The means of signal intensity of individual genes were determined from multiple probes and arrays, and used to calculate *p*-values by Student *t*-test and the false discovery rate (FDR) *q*-values by the Storey and Tibshirani method [25]. Using *q* ≤ 0.05 as the cutoff value, we identified 1,306 and 973 genes in the 0.5 h and 5 h groups, respectively, that were significantly changed in expression. Our qRT-PCR analysis of selected genes with different fold changes in the 0.5 h group also confirmed that the microarray data were generally reliable, although the exact fold changes might slightly vary between the two sets of data (Figure 3B). Among these regulated genes, we observed changes ranging from 2.48-fold (cgd8_5030, articulin family protein) to 0.33-fold (cgd1_1690, PDH finger containing protein) in the 0.5 h group, and from 1.97 (cgd7_2980, low complexity protein with a potential C2C2 zinc ribbon) to 0.543 (cgd5_4270, hypothetical membrane protein) in the 5 h group (Table 3).

**Table 2:** **Distribution of genes with significant changes in expressions based on their fold changes** (**threshold** = ±**1**.**2 fold**) **in the groups with 0**.**5 h and 5 h of recovery times after UV irradiation**

**Group(fold changes)**	**0**.**5 h(>1.2)**	**0.5 h(0**.**8-1**.**2)**	**0.5 h(<0.8)**	**Total**
**5 h** (>**1**.**2**)	31	100	11	142
**5 h** (**0**.**8**-**1**.**2**)	236	1,183	184	1,603
**5 h** (<**0**.**8**)	6	77	27	110
**Total**	273	1,360	222	1,855

**Table 3:** **List of top 15 up**- **and 15 down**-**regulated genes in the two UV**-**treated experimental groups**

**Functional Group**	**Fold(0.5h)**	**Fold(5 h)**	**GeneID**	**Gene Description**
**Top 15 up**-**regulated genes upon UV**-**treatment**				
Structure	2.479	1.169	cgd8_5030	articulin family protein, Pfs77 protein-related
Unknown	2.432	1.079	cgd7_3840	conserved hypothetical protein
Unknown	2.192	1.287	cgd3_1410	small hypothetical protein
Unknown	2.119	1.068	cgd3_2070	hypothetical protein
Unknown	2.003	1.074	cgd7_990	hypothetical protein
Unknown	1.985	1.032	cgd1_840	conserved hypothetical protein
Unknown	1.057	1.968	cgd7_2980	low complexity protein with C2C2 zinc ribbon
Redox homeostasis	1.861	0.987	cgd8_3510	thioredoxin-like protein, fragment
Unknown	1.860	1.111	cgd7_270	conserved hypothetical protein
Enzyme	1.845	1.142	cgd3_3320	putative phenylalanyl-tRNA synthetase
Unknown	1.032	1.831	cgd2_1040	flavohemoprotein b5+b5R (DJ676J13.1), putative
Unknown	1.808	1.068	cgd3_270	hypothetical protein
Unknown	1.808	1.089	cgd4_4030	hypothetical protein
Unknown	1.786	1.127	cgd7_4710	conserved hypothetical protein
Unknown	1.782	1.074	cgd3_220	hypothetical protein
**Top 15 down**-**regulated genes upon UV**-**treatment**				
Unknown	1.180	0.543	cgd5_4270	hypothetical protein, signal peptide & TM domain
Unknown	1.098	0.589	cgd6_4430	hypothetical protein
Unknown	1.086	0.587	cgd2_4060	hypothetical protein, 12 transmembrane domains
Enzyme	1.005	0.595	cgd4_960	phosphomannomutase
Unknown	0.979	0.549	cgd6_290	hypothetical protein
Unknown	0.974	0.552	cgd5_260	hypothetical protein
Enzyme	0.856	0.591	cgd6_4020	phosphdiesterase, putative
RNA metabolism	0.594	0.946	cgd6_410	Sgn1p-like RRM domain containing protein
Unknown	0.591	1.040	cgd6_1100	signal peptide-containing protein
Unknown	0.590	0.776	cgd6_4260	Low complexity protein
Unknown	0.582	1.260	cgd8_2760	conserved hypothetical protein
Unknown	0.577	0.850	cgd7_10	hypothetical protein
Enzyme	0.529	0.965	cgd1_3290	carboxylesterase, putative
Unknown	0.516	1.091	cgd7_1290	hypothetical protein containing a signal peptide
Unknown	0.329	1.117	cgd1_1690	PHD finger containing protein

To determine the patterns of gene expression changes in oocysts, we selected ±1.2-fold (±20%) change as the empirical cutoff value for “highly regulated” expressions, and genes were grouped into 3 clusters: >1.2 (up-regulated); between 1.2 and 0.8 (less regulated middle range); and <0.8 (down-regulated). This categorization allowed us to address the dynamics in the gene expressions after short (0.5 h) and long (5 h) post-irradiation recovery times with 9 distinguished clusters (e.g., cluster 1 genes maintaining up-regulated gene expression in both 0.5 h and 5 h groups; cluster 2 genes were first up-regulated and then resumed to near normal (middle range); cluster 3 genes were changed from up-regulated to down-regulated, and so forth) (Additional file 7: Figure S4). A complete list of genes grouped by major functions under the 9 patterns is provided in Additional file 6: Table S3, and summarized in Table 4.

**Table 4:** Functional categorization of significantly regulated genes in the oocysts upon UV irradiation

**Group 0**.**5 h**	**Up**	**Up**	**Up**	**Mid**	**Mid**	**Mid**	**Down**	**Down**	**Down**	**No**. **Total**	**% Total**
**Group 5**.**0 h**	**Up**	**Mid**	**Down**	**Up**	**Mid**	**Down**	**Up**	**Mid**	**Down**		
**Cluster**	**1**	**2**	**3**	**4**	**5**	**6**	**7**	**8**	**9**		
**Enzyme**	2	25	0	15	149	16	0	19	1	227	12.24
**RNA metabolism**	0	18	0	2	65	2	0	8	3	98	5.28
**Gene expression**	0	9	1	1	57	1	0	10	1	80	4.31
**Ribosome biogenesis**	0	7	0	0	74	0	0	7	4	92	4.96
**Transporter**	0	15	0	3	44	3	0	6	0	71	3.83
**Membrane**	0	6	0	1	24	3	0	4	0	38	2.05
**DNA metabolism**	2	9	0	0	33	1	0	2	0	47	2.53
**Protein degradation**	0	2	0	6	32	0	1	3	0	44	2.37
**Structure**	2	4	0	0	16	2	0	1	0	25	1.35
**Chaperonin**	1	2	0	2	18	2	0	2	0	27	1.46
**Cell cycle**	0	5	0	1	7	0	0	1	0	14	0.75
**Chromatin**	1	4	0	0	12	0	0	0	0	17	0.92
**Redox homeostasis**	0	2	0	0	12	1	0	1	0	16	0.86
**Mitochondrial**	0	4	0	0	5	0	0	1	1	11	0.59
**General**	0	0	0	1	2	0	0	1	0	4	0.22
**Total annotatable**	8	112	1	32	550	31	1	66	10	811	43.72
**Unknown function**	23	124	5	68	633	46	10	118	17	1044	56.28
**Grand Total**	31	236	6	100	1183	77	11	184	27	1855	100.00

Note: Up = up-regulated (fold changes >1.2), Mid = less significantly regulated “middle” group (fold changes between 0.8 to 1.2), Down = down-regulated (fold changes <0.8).

Based on the ±1.2-fold cutoff, 1,183 (~64% of the 1,855 genes with *p*-values ≤ 0.05) were considered as “less regulated (middle range)” in both 0.5 h and 5 h groups (cluster 5) (Table 4). Most of the other 672 genes fell into the following 4 clusters: 236 (cluster 2) or 184 (cluster 8) genes were first up- or down-regulated in 0.5 h and then returned to the middle range in 5 h, respectively; 100 (cluster 4) and 77 (cluster 6) genes were first in the mid-range (i.e., not highly regulated) and then became up- or down-regulated, respectively. We were able to categorize 811 genes (~44% of the 1,855 genes) into major functional groups, in which the largest group was enzymes, followed by those involved in RNA metabolism, core gene expression and ribosome biogenesis, among others (Table 4). These observations indicated that gene expressions in the oocysts were dynamically and comprehensively regulated during the cause of recovery after UV-irradiation.

At the early 0.5 h recovery time, in a few groups the proportion of up-regulated members appeared to exceed the down-regulated ones, which included those involved in cell cycle control (e.g., CDC and cyclin proteins), DNA metabolism (e.g., DNA repair and replication factors), chromatin associated protein, cytoskeletal proteins and various transporters (Table 4 and Additional file 6, Table S3). The data agreed with our general assumptions that oocysts need to activate pathways to repair DNA, other macromolecules, and cellular components damaged by UV-irradiation. The cytoskeletal rearrangement became more active, which was probably associated with the repair of damaged cytoskeletal components and intracellular trafficking based on the elevated expressions of motor molecules (e.g., dynein subunits, gamma tubulin and microtubule-associated proteins) and a number of membrane-trafficking associated proteins (e.g., various subunits of adaptin and coatomer protein complex).

A large subset of transporters was regulated. Each group of transporters possessed both up- and down-regulated members, with the top up-regulated genes were predominantly ABC-type, ion, nutrient and nuclear transporters that were mostly resumed to near normal levels in 5 h. The highly down-regulated genes were predominantly vacuolar ATPase subunits that remained down-regulated in 5 h. The increased expressions of several oocyst wall proteins; articulin proteins that are known to be important in maintaining cell shapes in *Euglena* and ciliates [26,27], and mucin-like proteins suggestive of active repairing on some cell/oocyst surface molecules.

Among stress-related genes, predominant up-regulated members included chaperonins and redox homeostasis, three putative t-complex protein 1 (TCP-1) subunits and several thioredoxin-associated genes; whereas various HSP/DNAj family members, as well as SOD and glutaredoxin-related genes were either much less regulated or down-regulated. It has been reported that a HSP70 gene (cgd2_20) exhibits >10-fold increased expression in response to heat-shock at 45°C for 20 min [28]. These data suggest that different proteins within this functional group are responsible to handle different stresses. The chaperonin containing TCP-1 (CCT) is known to be required for the production/folding of actin and tubulin among the others [29,30]. The up-regulated expression of TCP-1 subunits correlates well with those of actin and several other cytoskeletal genes.

In the 5 h group, the majority of the highly up- or down-regulated genes resumed, or displayed a clear trend toward the middle-range levels of expression (clusters 2 and 8). Only 31 or 27 genes stayed either up- or down-regulated expressions, respectively, in both 0.5 h and 5 h groups (clusters 1 and 9), in which only a small number of genes could be functionally categorized. Among them, genes encoding a DNA replication licensing factor (cgd2_1250) and DNA polymerase epsilon subunit (cgd8_1240) remained up-regulated in both 0.5 h and 5 h groups, which suggested that DNA repair machinery in the oocysts was activated soon after UV-irradiation, and lasting for >5 h. The data were generally in agreement with our previous observations on a select panel of DNA repair-associated genes in the parasite in response to UV-irradiation [31]. Additionally, the long-lasting up-regulated expressions of γ-tubulin and a microtubule-associated protein indicated that the cytoskeletal elements underwent substantial rearrangements in the parasite, probably as needed for transporting and recycling the damaged macromolecules and cellular components. Several genes involved in the ribosome biogenesis and RNA metabolism remained down-regulated in both groups, although they were expressed at relatively high levels in normal oocysts.

Few genes had reversed their expression patterns (clusters 3 and 7). However, a substantial number of genes went up or down from the middle range (clusters 4 and 6), in which mostly were enzymes among the annotatable genes. The proteasome and ubiquitin associated genes appeared to be the group after the enzymes with more genes went up in 5 h group from mid-range expressions in 0.5 h group (cluster 4). In this group, there were also three and one lowered expressed genes bounced back to mid-range or higher levels of expression, separately (clusters 7 and 9), but none went further down, indicating that the activities associated with protein degradation and amino acid recycling remained relatively high for a substantial long time after UV-irradiation.

More regulated genes could not be annotated into functional groups, which included those encoding hypothetical or membrane proteins (Table 4). Further functional analyses of these genes may not only assist in their annotations, but also shed new light into their functions and roles in response to environmental stresses.

## Conclusions

We have developed an Agilent microarray for *C*. *parvum* (CpArray15K) that covers all predicted ORFs in the parasite genome. Using CpArray15K coupled with real-time qRT-PCR of selected genes, we have observed that the parasite oocysts are highly active in protein synthesis based on the high levels of expressions of genes associated with ribosome biogenesis, transcription and translation. Proteasome and ubiquitin associated components are also highly active, implying that this environmental stage of parasite may employ protein degradation pathways to recycle amino acids in order to overcome an inability to synthesize amino acids de novo.

The energy metabolism in oocysts is highly active, as expected, and featured by the highest level of expression of the LDH gene in the oocysts. *Cryptosporidium* is able to produce lactate, alcohol and acetate as organic end products. Our follow-up qRT-PCR analysis confirms that the oocysts rely on lactate production, whereas the intracellular parasite developmental stages likely depend more on alcohol and acetate end products to maintain essential carbon flow.

In response to UV-irradiation, the parasite oocysts undergo complex and dynamic regulations in gene expression. The majority of the top regulated genes changed their expression levels by around +2.5 and −3.0 folds, respectively, with only a few genes showing ≥2-fold changes. Increased activations in DNA repair and intracellular trafficking were also observed. Among the stress-related genes, TCP-1 family members and some thioredoxin-associated genes appear to play important roles in the recovery of UV-induced damages in the oocysts. Our observations also implied increased activities in cytoskeletal rearrangement and intracellular membrane trafficking in the parasite oocysts upon UV irradiation.

CpArray15K is the first microarray chip developed for *C*. *parvum*, which provides the *Cryptosporidium* research community a needed tool to study parasite transcriptome and functional genomics. Although next generation of sequencing (NGS)-based RNA-Seq has been used increasingly in transcriptome analysis, microarray remains a powerful complementary technology with advantages on low costs, short turn-round time and ease of data generation [32]. CpArray15K has been successfully used in profiling the gene expressions in the parasite oocysts as well as their responses to UV-irradiation. The current version may be further expanded to include additional probes, such as the non-coding regions and SNPs, by taking advantage of the small parasite genome similar to the multi-functional array developed for *Toxoplasma gondii*[33].

## Methods

### Development of agilent microarray for *C*. *parvum*

Sequence information for *C*. *parvum* (IOWA-1 strain) was retrieved from CryptoDB ( http://www.CryptoDB.org; release 4.9) comprising 3,805 genes which are predicted to encode proteins. For these genes 60-mers of oligonucleotide probes were designed using Array Designer v4.0 by Premier Biosoft (Palo Alto, CA). The microarray consisted of 10,009 *C*. *parvum*-specific probes with one to three probes per gene depending on the gene size (i.e., average 2.63 probe/gene, including 650 genes with single, 106 with two, and 3,049 with three probes). Among them, an additional quality control of 30 replicate probes were made for 47 stress-related genes. The final form of the microarray contained 15,208 *C*. *parvum*-specific probe spots in the microarray, and was thus termed as CpArray15K. The array also contained 77 negative and 459 positive internal control spots specific to the Agilent platform.

### Manipulation of parasite oocysts

Fresh *C*. *parvum* oocysts (IOWA-I strain, one month old) were purchased from Bunch Grass Farm (Deary, Idaho) and used to isolate total RNA. To reduce the effects of temperature change on the gene expression, oocysts were first placed overnight at room temperature. Oocysts were microscopically examined, counted in a hemocytometer, and divided into 8 groups (9 x 10^7^ oocysts/group) in 12-well plates, in which 4 groups were treated with an ID_20_ UV dose at 254 nm (2.8 mJ/cm^2^) in a Stratalinker UV Crosslinker (model 1800, Stratagene). The remaining 4 groups (untreated controls) were placed outside of the Crosslinker during the treatment. After UV-irradiation, the oocysts were further divided into two groups (each containing two treated and two untreated samples, i.e., two biological replicates) and allowed to recover for 0.5 h and 5.0 h, respectively, followed by RNA isolation.

The ID_20_ UV dose was determined by an in vitro assay as described [34,35]. Briefly, fresh oocysts were treated with a serial UV doses (0, 2.5 to 40 mJ/cm^2^), inoculated into the HCT-8 cell monolayers cultured in a 24-well plate, and allowed for excystation and infection for 5 h at 37 °C. Infected cell monolayers were washed with PBS to remove free oocysts for isolating total RNA using Qiagen RNeasy kit, and the relative levels of parasite infection were evaluated by a qRT-PCR assay that determined the relative levels of parasite 18S rRNA [34,35].

### Preparation of cRNA probes and hybridization

Immediately after treatments, all samples were disrupted in 350 μl of lysis buffer and were snap-frozen in liquid nitrogen. Total RNA was then isolated from samples thawed on ice using an RNeasy isolation kit (Qiagen, Valencia, CA). DNA was removed from the isolated RNA samples using RNase-free DNase according to the manufacturer’s protocol. The quality and purity of RNA was determined by NanoDrop ND-1000 spectrophotometer at 260/280 nm (NanoDrop Technologies, Wilmington, Delaware). The integrity of total RNA was assessed with an Agilent Bioanalyzer 2100 and RNA 6000 Nano LabChip Kit (Agilent Technologies, Palo Alto, CA). Only RNA samples with the RNA Integrity Numbers (RINs) values of ≥6 were used in subsequent analysis.

The probe labeling and hybridization procedures generally followed the Agilent’s recommendations as described [36]. Probes labeled with cyanine 3 (Cy3) or cyanine 5 (Cy5) were prepared from the 8 RNA samples using a two-color dye-swap design using Low Input Quick Amp Labeling Kit (Agilent Technologies). Briefly, 100 ng of total RNA from each sample was first reverse-transcribed into cDNA which was in turn transcribed into Cy3- and Cy5-labeled cRNA probes, respectively (Perkin Elmer, Wellesley, MA). Labeled cRNA was purified with RNeasy Mini columns (Qiagen, Valecia, CA). The quality of each cRNA probes was verified by total yield and specificity calculated based on NanoDrop ND-1000 spectrophotometer measurement (NanoDrop Technologies) (Wilmington, DE). Only probes with specificity >8 were used for hybridization using an In Situ Hybridization Kit Plus (Agilent Technologies). Each array was incubated with an equal amount of Cy3 and Cy5 probes (300 ng) derived from a pair of control and treated samples at 65°C for 17 h in Agilent's microarray hybridization chambers. After washes, the array chips were ready for scanning according to the Agilent protocol.

### Signal acquisition, data analysis and data deposition

Median signals and background intensities in the hybridized microarrays were acquired from both Cy3 and Cy5 channels using an Agilent High-Resolution Microarray Scanner (Santa Clara, CA). Data were globally normalized using LOWESS algorithm [37]. The normalized natural log intensities were analyzed using a mixed model by SAS (SAS, Cary, NC) with a fixed effect of treatment (irradiated or non-irradiated) and dye (Cy5 or Cy3), and random effect of slide and array. We also calculated the means and standard deviations (SDs) of individual probes among 8 arrays, as well as the means and SDs of all individual genes from multiple probes, to evaluate the inter-array and inter-probe variations. Minimum Information About a Microarray Experiment (MIAME) information about this experiment has been deposited to the NCBI’s Gene Expression Omnibus (GEO) database [38]. Microarray data can be accessed with the following accession numbers: GEO platform number GPL14756, GEO series number GSE34307, and sample accession numbers GSE847065 to GSE847072.

To gain insight into the metabolic features in the parasite oocysts, we first performed analysis of data derived from the 4 untreated samples. Genes were grouped based on their expression levels using signal to noise ratio (SNR) and signal to background ratio (SBR) in the 8 arrays as criterions [37]: Group I (positively expressed), SNR > 2.0 and SBR > 2.6 in all spots in at least 5 arrays; Group II (unexpressed to lowly expressed), SNR ≤ 2.0 and SBR ≤ 2.6 in all spots and all arrays; and Group III (lowly expressed), neither Group I nor II, SNR > 2.0 and/or SBR > 2.6 in ≤ 3 arrays.

To study the gene expressions in oocysts in response to UV-irradiation, fold changes in the expressions were derived from the normalized data and expressed as the means of all respective probes in all arrays. Changes were considered as statistically significant in genes with *p*-values ≤ 0.05 by two-tailed Student *t*-test. False discovery rate (FDR) (*q*-values) was calculated using an R program QVALUE according to Storey and Tibshirani [25].

### Real-time quantitative RT-PCR (qRT-PCR)

Total RNA from *C*. *parvum* oocysts, free sporozoites and intracellular developmental stages were used for qRT-PCR analysis. Free sporozoites were obtained by an in vitro excystation procedure, in which 4x10^7^ oocysts were incubated with PBS containing 0.25% trypsin and 0.5% TDC at 37°C for 90 min. Trypsin was then quenched by adding bovine serum albumin (BSA) to the solution (final concentration = 1%), and excysted sporozoites were washed three times in 1X PBS by centrifugation before RNA isolation. Different intracellular developmental stages were obtained by infecting HCT-8 cells in vitro [31,39,40]. Briefly, HCT-8 cells were seeded into 24-well plates and allowed to grow overnight until the cell densities reached to ~80% - 90% confluence. Cells were inoculated with *C*. *parvum* at a host cell:oocyst ratio at 1:1 for 6, 12, 24 and 36 h infection times, or 5:1 for 48 and 72 h infections. After initial incubation for 3 h to allow parasite excystation and invasion, uninfected parasites were removed by a medium exchange. Infected cells were allowed to grow further for specified infection times. Total RNA was isolated from *C*. *parvum* oocysts, free sporozoites and intracellular stages using an RNeasy isolation kit (Qiagen).

A Qiagen one-step RT-PCR QuantiTect SYBR-Green RT-PCR kit was employed to evaluate gene expression levels with the listed primers (Additional file 8: Table S4) as described (e.g., [31,39,40]). Briefly, Each 20 μl reaction mixture contained 10 ng total RNA, 500 nM each primer, 10 nM FITC, 0.25 μl master mix, and 1X QuantiTect SYBR-Green. The mixtures were incubated at 50°C for 30 min to synthesize cDNA, heated at 95°C for 15 min to inactivate the reverse transcriptase, and then subjected to 40 thermal cycles of PCR amplification (95°C for 20 s, 58°C for 30 s, and 72°C for 30 s) with an iCycler iQ real-time PCR detection system (Bio-Rad Laboratories, Hercules, CA). At least 2 qRT-PCR reaction replicates were performed for each sample. The parasite 18S rRNA or GAPDH mRNA levels were used as the internal standard for normalization. All reagents for the qRT-PCR were loaded manually.

In all analyses, the ΔC_T_ values of a target gene were first calculated (i.e., *Δ*C_T[gene]_ = C_T[gene]_ − C_T[18S]_). To determine the relative levels of gene expressions in oocysts and other developmental stages, we used the overall mean of ΔC_T_ values (ΔC_T[mean]_) from all samples in each set of analysis as the baseline for computing ΔΔC_T_ (i.e., *ΔΔ*C_T[gene]_ = *Δ*C_T[gene]_ − *Δ*C_T[mean]_). The relative levels of expression (against the mean) were then expressed by the empirical formula 2^-ΔΔCT^. To determine the gene expression fold changes in response to UV-irradiation, the ΔΔC_T_ values of specified genes were computed between the UV-treated and untreated groups (i.e., $\Delta \Delta {\text{C}}_{\text{T[gene]}}=\Delta {\text{C}}_{\text{T[gene]}}^{\text{(treated samples)}}-\Delta {\text{C}}_{\text{T[gene]}}^{\text{(controls)}}$), followed by the use of the empirical formula 2^-ΔΔCT^ for calculating the fold-changes.

## Competing interests

The authors claim no competing interests.

## Authors’ contributions

GZ contributed to the microarray design, experimental design, data analysis and manuscript writing. HJZ contributed to the microarray and experimental design, participated in data analysis and manuscript writing. HLZ contributed experimental design and carried out most of the experiments including RNA isolation, probe preparation, array hybridization and scan, and qRT-PCR. FGG provided assistance in experiments. All authors read and approved the manuscript.

## Supplementary Material

Additional file 1 **Figure S1.** Morphology of *Cryptosporidium parvum* oocysts before (A) and after (B) equilibrated to room temperature overnight (RT O/N). Oocyts were stored at 4 °C prior to equilibration at room temperature overnight before UV-irradiation and RNA extraction to minimize the effect of temperature variations on gene expressions. Click here for file

Additional file 2 **Table S1.** Complete list of Groups I and II genes classified under major functional categories. Their expression levels are indicated by quartiles, i.e., L0 = lowly or unexpressed Group II genes, L1 to L4 = Group I genes with expression levels in the parasite oocysts at bottom to top quartiles. Genes with expression at L0 and L4 levels are highlighted by green and pink colors for easy identification. Click here for file

Additional file 3 ** Figure S2.** Comparison of data derived from Mauzy et al. (2012) and this study by qRT-PCR on the relative levels of *Cryptosporidium parvum* LDH, ADH and AceCL genes in the intracellular developmental stages. Data from Mauzy et al. (2012) were extracted from the CryptoDB databases ( http://www.CryptoDB.org). Click here for file

Additional file 4 **Table S2.** A) Functional clarification of the top 100 genes in *Toxoplasma gondii* with the highest levels of expression in the oocysts sporulated for 10 days; B) List of the top 100 highly expressed genes in the *Toxoplasma gondii* oocysts sporulated for 10 days. Original data were generated by Fritz et al. (2012) and extracted from http://www.ToxoDB.org. Click here for file

Additional file 5 **Figure S3.** Relative levels of the two *Toxoplasma gondii* LDH genes in oocysts, tachyzoites and bradyzoites. Data used in this analysis were extracted from the ToxoDB databases ( http://www.ToxoDB.org). Click here for file

Additional file 6 **Table S3.** Complete list of significantly regulated genes in the oocysts upon UV-irradiation and recovered for 0.5 h and 5 h. Genes are grouped into 9 clusters based their expression patterns. Click here for file

Additional file 7 **Figure S4.** Distribution of regulated genes into 9 clusters based on the expression dynamics in the UV-irradiated oocysts after 0.5 h and 5 h of recovery times. Fold changes in gene expression are shown as ratios of normalized median signals between treated and control groups (T/C). Click here for file

Additional file 8 **Table S4.** List of primers used in this study. Click here for file
