# Supplementary material for: Transcriptome analysis reveals unique metabolic features in the Cryptosporidium parvum Oocysts associated with environmental survival and stresses
Source: BMC Genomics. 2012 Nov 21;13:647. doi: 10.1186/1471-2164-13-647 (PMC3542205; doi:10.1186/1471-2164-13-647)

**Figure S1.**

Morphology of *Cryptosporidium parvum* oocysts before (**A**) and after (**B**) equilibrated to room temperature overnight (RT O/N). Oocysts were stored at 4 C prior to equilibration at room temperature overnight before UV-irradiation and RNA extraction to minimize the effect of temperature variations on gene expressions.

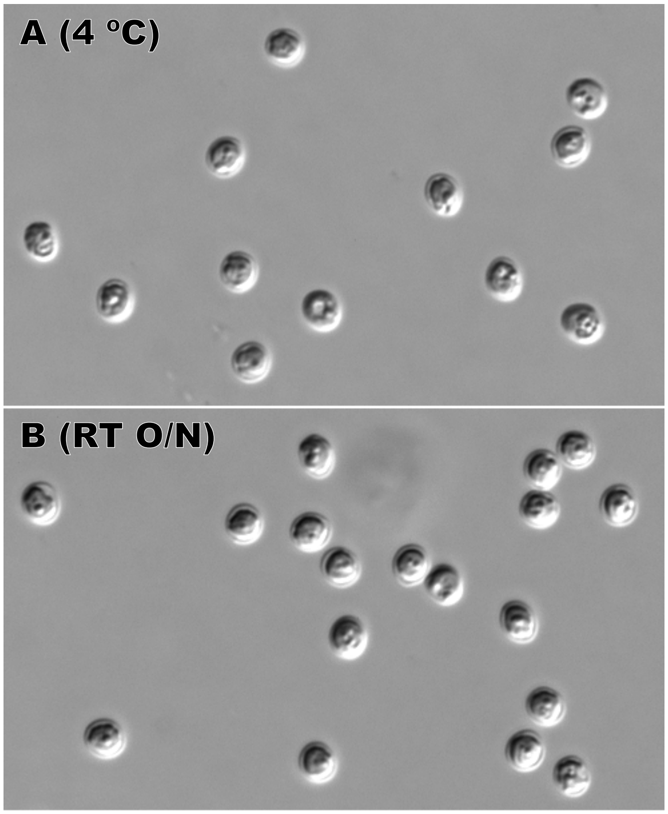

Supplement: Additional file 1 — Figure S1. Morphology of Cryptosporidium parvum oocysts before (A) and after (B) equilibrated to room temperature overnight (RT O/N). Oocyts were stored at 4 °C prior to equilibration at room temperature overnight before UV-irradiation and RNA extraction to minimize the effect of temperature variations on gene expressions. [file 1471-2164-13-647-S1.pdf]
