# Supplementary material for: Transcriptome analysis reveals unique metabolic features in the Cryptosporidium parvum Oocysts associated with environmental survival and stresses
Source: BMC Genomics. 2012 Nov 21;13:647. doi: 10.1186/1471-2164-13-647 (PMC3542205; doi:10.1186/1471-2164-13-647)
Supplement: Additional file 2 — Table S1. Complete list of Groups I and II genes classified under major functional categories. Their expression levels are indicated by quartiles, i.e., L0 = lowly or unexpressed Group II genes, L1 to L4 = Group I genes with expression levels in the parasite oocysts at bottom to top quartiles. Genes with expression at L0 and L4 levels are highlighted by green and pink colors for easy identification. [file 1471-2164-13-647-S2.pdf]

Table S1. Complete list of Groups I and II genes classified under major functional categories. Their expression levels are indicated by quartiles, i.e., L0 = lowly or unexpressed Group II genes, L1 to L4 = Group I genes with expression levels in the parasite oocysts at bottom to top quartiles. Genes with expression at L0 and L4 levels are highlighted by green and pink colors for easy identification.

Major Group	Subgroup	GeneID	Description	L0	L1	L2	L3	L4	Total	Signal Mean
**Cell cycle**	CDC related	cgd1_2180	CDC48 like AAA ATPase			1			1	4.136
		cgd2_1540	cell growth-regulating nucleolar protein, metal binding domain at N-terminus				1		1	12.836
		cgd5_2510	Cdc2-like CDK2/CDC28 like protein kinase	1					1	0.598
		cgd6_1420	cdc2-related protein kinase, putative			1			1	2.612
		cgd6_1830	HT-1080/Cdc123p - like protein			1			1	3.440
		cgd6_2060	cdc39p protein-like; C-terminal Not1, CCR4-Not complex component; Not1					1	1	41.270
		cgd7_1930	CDC68 like aminopeptidase family chromatinic protein (possible inactive enzyme)	1					1	0.382
		cgd7_280	cell division control protein 28, putative		1				1	1.657
		cgd7_4470	CDC14 phosphatase				1		1	21.845
		cgd8_2380	CDC45 like protein, possible horizontal transfer	1					1	0.389
		cgd8_4460	cell differentiation protein rcd1, putative			1			1	2.494
	CDC related Total			3	1	4	2	1	11	8.333
	Cyclin related	cgd1_2320	cyclin B, saccharomyces Clb4p like				1		1	7.617
		cgd1_3150	cyclin'cyclin'	1					1	0.397
		cgd1_3310	cyclin domain protein, possible cyclin H	1					1	0.311
		cgd1_60	cyclin dependent kinase A, putative	1					1	0.359
		cgd2_2290	cyclin 6 pcl7			1			1	3.743
		cgd3_4050	cyclin	1					1	0.393
		cgd5_3540	cyclin domain protein					1	1	66.359
		cgd6_1840	cyclin M2-like membrane-associated protein with 4 transmembrane domains and 2 CBS domains	1					1	0.568
		cgd7_1200	cyclin		1				1	1.367
		cgd7_2490	cyclin'cyclin'	1					1	0.517
		cgd7_3780	cyclin					1	1	318.539
		cgd7_430	cyclin-dependent kinase-related kinase, putative				1		1	8.899
		cgd7_520	cyclin'cyclophilin like peptidyl-prolyl cis-trans isomerase fused to WD40 repeats at the N-terminus'	1					1	0.521
		cgd7_660	cyclin; possible cyclin A	1					1	0.366
		cgd8_2510	Cyclin dependent kinase regulatory subunit, putative			1			1	5.273
	Cyclin related Total			8	1	2	2	2	15	27.682
**Cell cycle Total**				11	2	6	4	3	26	19.496
**Chaperone**	DNAj	cgd1_2280	DNAJ	1					1	0.442
		cgd1_2970	DNAJ like chaperone, 4 or more transmembrane domains			1			1	4.067
		cgd2_1330	DNAJ protein					1	1	26.709
		cgd2_2260	zuotin related factor-1 like protein with a DNAJ domain at the N-terminus and 2 SANT domains				1		1	21.815
		cgd2_3230	heat shock protein DnaJ Pfj2, putative			1			1	3.822
		cgd3_930	DnaJ domain, possible zf-CSL following				1		1	14.780
		cgd4_2780	DNAj domain protein having a signal peptide	1					1	0.344
		cgd5_2950	DNAj domain protein				1		1	9.330
		cgd5_3520	chaperone'DNAj domain protein chaperone'			1			1	3.142
		cgd5_3530	DNAj domain protein	1					1	0.411
		cgd6_1190	DNAJ protein-like, putative	1					1	0.348
		cgd6_2650	heat shock protein, putative		1				1	2.095
		cgd7_3880	DNAJ domain protein sec63 ortholog, 4 transmembrane domains	1					1	0.298
		cgd8_2820	membrane associated DNAJ with 6 transmembrane domain, signal peptide	1					1	0.350
		cgd8_2860	DNAJ protein			1			1	5.557
		cgd8_3270	DNAJ'DNAJ protein'				1		1	22.075
		cgd8_3770	DNAJ like chaperone					1	1	183.367
		cgd8_450	DNAj-like protein			1			1	3.296
		cgd8_4930	DNAJ protein	1					1	0.352
	DNAj Total			7	1	5	4	2	19	15.926
	HSP	cgd2_1800	heat shock 40 kDa protein, putative	1					1	0.434
		cgd2_20	heat shock 70 (HSP70) protein					1	1	417.813
		cgd3_3770	Hsp90					1	1	1,229.346
		cgd4_3270	heat shock 105kD; heat shock 105kD alpha; heat shock 105kD beta; heat shock 105kDa protein 1		1				1	1.673
		cgd6_1630	p23; HSP20-like chaperones fold		1				1	1.698
		cgd6_4970	Hsp60; GroEL-like chaperone (ATpase), predicted mitochondrial	1					1	0.439
		cgd7_2400	chaperonin 10 Kd subunit, putative		1				1	1.244
		cgd7_360	heat shock protein, Hsp70					1	1	65.777
		cgd7_3670	heat shock protein 90 (Hsp90), signal peptide plus ER retention motif		1				1	1.640
		cgd8_1610	sacsin like HSP90 chaperone domain, likely plant origin	1					1	0.335
	HSP Total			3	4			3	10	172.040
	Other	cgd2_1850	stress-induced protein sti1-like protein, putative [Tetratricopeptide repeat domain]			1			1	3.400
		cgd3_3440	heat shock protein HSP70, mitochondrial			1			1	3.006
		cgd5_3510	co-chaperone GrpE, putative			1			1	3.351

		cgd8_2340	cold shock RNA binding domain of the OB fold					1	1	58.377
	Other Total					3		1	4	17.033
	TCP1	cgd2_600	TCP1_alpha; TCP-1 (CTT or eukaryotic type II) chaperonin family, alpha subunit.	1					1	0.483
		cgd2_900	TCP1_theta; TCP-1 (CTT or eukaryotic type II) chaperonin family, theta subunit.			1			1	3.751
		cgd4_3850	TCP1_gamma; TCP-1 (CTT or eukaryotic type II) chaperonin family, gamma subunit.		1				1	1.847
		cgd6_5080	TCP1_beta; TCP-1 (CTT or eukaryotic type II) chaperonin family, beta subunit.			1			1	5.510
		cgd7_3180	TCP1_epsilon; TCP-1 (CTT or eukaryotic type II) chaperonin family, epsilon subunit.		1				1	1.456
	TCP1 Total			1	2	2			5	2.609
**Chaperone Total**				11	7	10	4	6	38	55.373
**Chromatin**	Chromatin protein	cgd1_1640	chromatin protein with an AT hook and a PHD finger domain at the C-terminus			1			1	2.881
		cgd3_2540	histone H3					1	1	33.649
		cgd4_3220	histone H3			1			1	5.149
		cgd4_720	putative nucleosome assembly protein 19075338; besthit Py 23481872				1		1	8.005
		cgd5_2130	chromatin associated proein with a chromodomain at the C-terminus			1			1	3.175
		cgd5_3170	histone H2B			1			1	3.830
		cgd5_940	histone H2A					1	1	58.102
		cgd6_4510	telomeric DNA binding protein; SANT DNA-binding domain				1		1	5.823
		cgd7_1700	histone H2B					1	1	75.694
		cgd8_2170	histone H2A			1			1	3.817
		cgd8_2730	multidomain chromatinic protein with the following architecture: 3x PHD-bromo-3xPHD-SET domain and associated cysteine cluster at the C-terminus	1					1	0.474
		cgd8_3970	NAP, nucleosome assembly protein			1			1	2.736
		cgd8_5230	histone H4				1		1	10.420
	Chromatin protein Total			1		6	3	3	13	16.442
	Chromatin remodeling	cgd1_1330	SWI/SNF-related, matrix associated, actin-dependent regulator of chromatin subfamily A containing DEAD/H box 1			1			1	3.424
		cgd3_3190	GCN5 like acetylase + bromodomain			1			1	4.705
		cgd4_1340	bromo domain containing protein	1					1	0.321
		cgd6_1380	histone deacetylase HDA2/Rpd3p				1		1	18.823
		cgd6_2590	histone acetyltransferase (MYST family); chromo domain + C2H2 domain					1	1	72.812
		cgd6_80	RPD3/HD1 histone deacetylase				1		1	6.611
		cgd7_2030	bacterial-like Sir2 family protein (HDAC Class III)	1					1	0.386
		cgd7_3600	Hs17p, histone methylase	1					1	0.410
		cgd7_3630	TRF4/5 nucletotidyl transferase		1				1	1.951
		cgd7_5210	bromodomain protein					1	1	24.589
		cgd7_60	Hat1-like acetyltransferase (histone acetyltransferase-like)	1					1	0.381
		cgd8_480	histone deactylase of possible bacterial origin with ankyrin repeats at the N-terminus	1					1	0.368
	Chromatin remodeling Total			5	1	2	2	2	12	11.232
**Chromatin Total**				6	1	8	5	5	25	13.941
**DNA metabolism**	DNA repair	cgd1_2840	contains a UVDDB domain that is present in CPSF_A and damage specific DNA binding protein 1			1			1	2.397
		cgd1_310	AP endonuclease of the TIM barrel fold, possible bacterial horizontal transfer		1				1	1.803
		cgd2_700	PMS1'MutL family ATpase'		1				1	2.247
		cgd4_140	Swi2/Snf2 ATpase,Rad16 ortholog				1		1	17.634
		cgd4_2960	ERCC4/XP-G-type excision repair nuclease	1					1	0.408
		cgd4_2970	RAD1, NH2 terminal ERCC4-like helicase domain	1					1	0.370
		cgd5_1900	XPA binding protein 1				1		1	7.154
		cgd5_2190	Nuc1p like endonuclease G		1				1	1.710
		cgd5_410	Rad51	1					1	0.401
		cgd5_4290	Swi/SNf2 RAD26			1			1	5.193
		cgd6_1530	DNA repair protein Rad4p		1				1	1.856
		cgd6_2610	ERCC1 excision repair 1; C-terminal HhH domain	1	1				2	1.064
		cgd6_2660	DNA repair helicase		1				1	1.976
		cgd6_2770	uvb-resistance protein uvr8, putative	1					1	0.484
		cgd7_2090	Ruv DNA-helicase-related protein				1		1	10.664
		cgd7_2660	RAD24/Rf-C activator 1 AAA+ ATpase				1		1	14.760
		cgd7_3350	eukaryotic DNA topoisomerase I		1				1	1.872
		cgd7_4730	RAD23p, UB+UBA domains protein	1					1	0.571
		cgd7_80	XPA1 binding protein-like GTpase		1				1	1.255
		cgd7_820	RAD3'DEXDc+HELICc protein'	1					1	0.389
		cgd8_1940	RAD25, helicase involved in DNA repair	1					1	0.308
		cgd8_3210	UvrD like super family I helicase involved in nucleotide excision repair, possible bacterial horizontal transfer	1					1	0.510
	DNA repair Total			9	8	2	4		23	3.308
	DNA replication	cgd1_3410	DNA replication complex GINS protein PSF2, putative	1					1	0.317
		cgd2_1100	DNA replication licensing factor MCM2 like AAA+ ATpase	1					1	0.421
		cgd2_1250	DNA replication licensing factor MCM4 like AAA+ ATpase	1					1	0.303
		cgd2_1600	DNA replication licensing factor MCM3 like	1					1	0.418

		cgd2_2500	DNA polymerase epsilon subunit	1					1	0.332
		cgd2_2730	Po1 beta superfamily nucleotidyltransferase			1			1	2.854
		cgd2_3180	replication factor C subunit 5, putative	1	1				2	0.854
		cgd2_4080	RP-A, OB fold single strand binding protein	1					1	0.403
		cgd3_1450	replication factor C like AAA+ ATpase	1					1	0.503
		cgd3_2210	replication protein A1 large subunit	1					1	0.386
		cgd3_2720	putative topoisomerase VIA	1					1	0.432
		cgd3_3170	replication factor RFC3 AAA+ ATpase	1					1	0.344
		cgd3_3820	DNA LIGASE I	1					1	0.324
		cgd3_390	RecQ SF II RNA helicase, DEXDc+HELICc				1		1	5.986
		cgd3_4290	DNA polymerase alpha catalytic subunit	1					1	0.527
		cgd4_3920	DinB/family X-type DNA polymerase	1					1	0.429
		cgd4_780	DNA topoisomerase II	1					1	0.430
		cgd4_970	DNA replication licensing factor MCM7 like AAA+ ATpase	1					1	0.347
		cgd5_1180	DNA topoisomerase III, putative	1					1	0.529
		cgd6_2390	DNA topoisomerase III beta-1, putative		1				1	1.911
		cgd6_240	DNA replication licensing factor MCM6-like AAA ATpase	1					1	0.422
		cgd6_4410	DNA polymerase delta catalytic subunit. DNAQ-like 3'-5' exonuclease; RNAseH fold	1					1	0.421
		cgd6_4420	RecQ bloom helicase (RNA helicase+hrdc)		1				1	1.573
		cgd7_1690	meiotic recombination protein DMC1-like protein	1					1	0.395
		cgd7_4690	RF-C paralog (Ctf18p) AAA+ ATpase	1	1				2	0.900
		cgd8_1240	DNA polymerase epsilon catalytic subunit	1					1	0.362
		cgd8_1350	topoisomerase VIA/SpoII nuclease subunit, toprim domain	1					1	0.392
		cgd8_1410	DNA primase large subunit	1					1	0.430
		cgd8_1620	DNA polymerase delta2, inactive calcineurin like phosphatase subunit	1					1	0.419
		cgd8_2940	replication factor C like AAA ATpase	1					1	0.416
		cgd8_4950	RecQ4 SF II RNA helicase	1					1	0.458
		cgd8_610	DNA replication repC1, AAA+ ATpase with a BRCT domain at the N-terminus	1					1	0.561
		cgd8_870	DNA dependent DNA polymerase alpha subunit, inactive calcineurin like phosphatase subunit	1					1	0.396
	DNA replication Total			29	4	1	1		35	0.770
	Other	cgd1_2680	HUSSY-3 like methyltransferase					1	1	174.010
		cgd1_3420	conserved possible MUS81 endonuclease	1					1	0.329
		cgd1_3530	Gbp1/Gbp2p-like, putative single stranded G-strand telomeric DNA-binding protein					1	1	60.981
		cgd2_2120	high mobility group small protein		1				1	1.762
		cgd2_3070	high mobility group (HMG)-box					1	1	425.649
		cgd2_4190	DEXDc+HELICc, mus308/POLQ like SFII DNA helicase, no polymerase domain	1					1	0.383
		cgd2_4280	holiday junction resolvase, S1x1p, URI domain nuclease	1					1	0.463
		cgd5_3460	nucleotidase (5'-nucleotidase/2'-cyclic phosphodiesterase) of the calcineurin superfamily	1					1	0.650
		cgd6_1580	exonuclease i/din7p-like; xeroderma pigmentosum G N-region plus xeroderma pigmentosum G I-region plus HhH2 domain	1					1	0.390
		cgd7_1720	endonuclease III, putative	1					1	0.395
		cgd7_2140	flap endonuclease 1	1					1	0.321
		cgd8_340	exoribonuclease PH, putative	1					1	0.359
	Other Total			8	1			3	12	55.474
**DNA metabolism Total**				46	13	3	5	3	70	10.982
**Enzyme**	AA synthesis	cgd3_80	S-adenosylhomocysteinase EC 3.3.1.1	1					1	0.657
		cgd5_4540	asparagine synthetase A (AsnA) like protein		1				1	2.114
		cgd5_4560	tryptophan synthase trpB of possible bacterial origin	1					1	0.431
		cgd6_3720	pyrroline-5-carboxylate reductase	1			1		2	4.051
		cgd7_2650	s-adenosylmethionine synthetase (SAM) EC 2.5.1.6				1		1	8.223
		cgd7_4940	gamma-glutamyl phosphate reductase family protein	1					1	0.567
		cgd8_2580	mitochondrial serine hydroxymethyl transferase	1					1	0.465
		cgd8_2610	cytosolic serine hydroxymethyl transferase	1					1	0.567
	AA synthesis Total			6	1		2		9	2.347
	AA-tRNA synthesis	cgd1_2130	glutaminyl-tRNA synthetase, of predicted bacterial origin				1		1	24.646
		cgd3_3320	putative phenylalanyl-tRNA synthetase			1			1	4.640
		cgd3_3840	isoleucine-tRNA synthetase			1			1	2.270
		cgd6_2970	tyrosyl-tRNA synthetase (tyrosyl-tRNA ligase; TyrRS). class-I aaRS		1				1	2.173
		cgd6_4400	proline-tRNA synthetase; class II aaRS (ybak RNA binding domain plus tRNA synthetase)	1					1	0.311
		cgd6_560	alanyl-tRNA synthetase (with HxxxH domain)		1				1	1.651
		cgd6_960	cysteinyl-tRNA synthetase					1	1	45.100
		cgd7_1490	tryptophanyl-tRNA synthetase				1		1	5.739
		cgd7_1540	aspartate--tRNA ligase					1	1	102.610
		cgd7_1710	threonyl-tRNA synthetase (RNA binding domain TGS+HxxxH+tRNA synthetase)	1					1	0.565
		cgd8_3100	valyl-tRNA synthetase		1				1	2.110
		cgd8_3320	phenylalanyl-tRNA synthetase beta chain	1					1	0.383
		cgd8_3460	methionyl-tRNA synthetase of possible bacterial origin	1					1	0.372
		cgd8_350	asparaginyl-tRNA synthetase (NOB+tRNA synthase)				1		1	10.701
		cgd8_4010	histidyl-tRNA synthetase			1			1	4.239

		cgd8_4720	seryl-tRNA synthetase, cytoplasmic			1			1	3.620
		cgd8_790	glutamate--tRNA ligase	1					1	0.357
	AA-tRNA synthesis Total			5	3	4	3	2	17	12.440
	Energy	cgd1_1710	phosphoglycerate mutase	1					1	0.320
		cgd1_2040	pyruvate kinase			1			1	4.077
		cgd1_3020	fructose-1,6-bisphosphate aldolase				1		1	16.932
		cgd1_3040	triosephosphate isomerase			1			1	2.966
		cgd2_210	glycerol-3-phosphate dehydrogenase		1				1	1.886
		cgd2_2130	pyrophosphate-dependent phosphofructokinase			1			1	3.315
		cgd2_2340	R1 like alpha-glucan water dikinase			1			1	5.585
		cgd2_3260	phosphoglucomutase, tandemly duplicated gene					1	1	35.506
		cgd2_3270	phosphoglucomutase, tandemly duplicated gene					1	1	38.373
		cgd3_1400	pyrophosphate-dependent 6-phosphofructokinase	1					1	0.583
		cgd3_1580	alpha glucosidase-like faimly 31 glycosyl hydrolases			1			1	2.422
		cgd4_690	pyruvate:ferredoxin oxidoreductase/NADPH-cytochrome P450 reductase PNO		1				1	1.539
		cgd5_1960	enolase (2-phosphoglycerate dehydratase)					1	1	323.176
		cgd5_2910	alpha amylase	1	1				2	1.076
		cgd5_70	phosphoenolpyruvate carboxylase		1				1	1.637
		cgd5_750	Mdh; malate dehydrogenase (oxaloacetate-decarboxylating)(NADP+)				1		1	9.706
		cgd6_2450	glycogen phosphorylase				1		1	12.787
		cgd6_3280	glycogen branching enzyme (1,4-alpha-glucan branching enzyme)		1				1	1.291
		cgd6_3790	glyceraldehyde 3-phosphate dehydrogenase					1	1	143.725
		cgd6_3800	hexokinase			1			1	3.319
		cgd6_4330	phosphoglycerate mutase family	1					1	0.427
		cgd6_4900	acylphosphatase, putative					1	1	65.078
		cgd6_880	Gdb1p; glycogen debranching enzyme			1			1	2.503
		cgd7_3120	pyruvate decarboxylase				1		1	13.325
		cgd7_4270	glycolytic phosphoglycerate mutase				1		1	15.288
		cgd7_470	malate dehydrogenase, adjacent gene encodes predicted lactate dehydrogenase				1		1	10.334
		cgd7_480	lactate dehydrogenase, adjacent gene encodes predicted malate dehydrogenase					1	1	3,064.620
		cgd8_1720	acetaldehyde reductase plus alcohol dehydrogenase (AdhE) of possible bacterial origin				1		1	6.279
	Energy Total			4	5	7	7	6	29	130.660
	General	cgd1_1080	alpha beta hydrolase		1				1	1.296
		cgd1_1100	alpha beta hydrolase with 5 transmembrane domains	1					1	0.547
		cgd1_1730	haloacid dehalogenase family-like horizontal transfer into apicomplexans, signal peptide			1			1	4.116
		cgd1_2750	AAA superfamily ATpase	1					1	0.384
		cgd1_280	short chain dehydrogenase/reductase of the rossmann fold, signal peptide		1				1	2.273
		cgd1_3390	katanin p60/fidgetin family AAA ATpase		1				1	2.028
		cgd1_3610	secreted protein of the alpha beta hydrolase superfamily, signal peptide				1		1	8.260
		cgd1_3730	glucosamine-fructose-6-phosphate aminotransferase	1					1	0.462
		cgd1_990	pyridine nucleotide/ NAD(P) transhydrogenase alpha plus beta subunits, duplicated gene, 12 transmembrane domain	1					1	0.432
		cgd2_1120	putative molybdopterin synthase sulphurylase				1		1	10.116
		cgd2_1190	methylase		1				1	2.094
		cgd2_2170	Ynr053p-like, Yjeq GTpase				1		1	23.790
		cgd2_380	COG0237: dephospho-CoA kinase	1					1	0.439
		cgd2_3880	possible AAA domain containing protein	1					1	0.335
		cgd3_1360	methylase	1					1	0.510
		cgd3_2050	putative prolyl 4-hydroxylase alpha subunit homolog, 2-oxoglutarate-dependent dioxygenase	1					1	0.300
		cgd3_2290	possible domain AAA, ATpase family				1		1	17.443
		cgd3_330	scully CG7113-PA, putative					1	1	356.414
		cgd3_3910	prolyl 4-hydroxylase alpha subunit				1		1	14.869
		cgd3_990	CG6144-like AlkB	1					1	0.303
		cgd4_1130	PhnP like hydrolase of the metallobetalactamase fold	1					1	0.297
		cgd4_1940	putative nucleoside-diphosphate kinase				1		1	16.183
		cgd4_1960	ThiF/moeB family			1			1	2.540
		cgd4_2250	putative phosphopantothenoylcysteine synthetase	1					1	0.434
		cgd4_2270	oxidoreductase, short-chain dehydrogenase family, putative			1			1	4.526
		cgd4_2550	putative farnesyl pyrophosphate synthase		1				1	1.750
		cgd4_430	ORC5 like AAA+ ATpase		1				1	1.147
		cgd4_4320	ORC/CDC6 like AAA+ ATpase	1					1	0.366
		cgd5_1170	CPSF metallobeta-lactamase	1					1	0.342
		cgd5_2010	nuclear VCP like protein with 2 AAA ATpase domains					1	1	54.188
		cgd5_2410	conserved possible esterase of the beta-lactamase family, of possible plant or bacterial origin, possible transmembrane domain within N terminal region	1					1	0.407
		cgd5_2670	predicted mannitol dehydrogenase; zinc dependent alcohol dehydrogenase like rossmann fold	1					1	0.404
		cgd5_2790	katanin p60/fidgetin family with AAA ATpase				1		1	6.005

		cgd5_3090	N-acetyltransferase subunit ARD1	1					1	0.350
		cgd5_310	putative polyphosphate synthetase VTC4, 3 transmembrane domains at C-terminus				1		1	7.600
		cgd5_3110	MDN1, midasin	1					1	0.302
		cgd5_3960	secreted protein of the alpha beta hydrolase superfamily					1	1	29.019
		cgd5_4330	conserved methylase				1		1	14.596
		cgd5_460	possible alpha beta hydrolase	1					1	0.364
		cgd5_900	MRP like MinD family ATpase		1				1	1.579
		cgd6_1800	acetyltransferase, GNAT family	1					1	0.338
		cgd6_2820	2 SAM dependent methyltransferase; S-adenosyl-L-methionine-dependent methyltransferases + spermidine synthase (SAM dependent methyltranferase)		1				1	1.446
		cgd6_4020	phosphdiesterase, putative	1					1	0.329
		cgd6_4270	MinD type ATpase				1		1	8.560
		cgd6_4380	phosphatase subunit gene g4-1; of possible animal origin; EF-hand, calcium binding domains	1					1	0.390
		cgd6_550	Pch2p like AAA ATpase	1					1	0.267
		cgd7_1410	AAA domain containing protein				1		1	9.647
		cgd7_160	putative methyltransferase-related					1	1	44.371
		cgd7_1640	SRP54. signal recognition 54. GTpase.				1		1	19.559
		cgd7_2310	ORC/CDC6 like AAA ATpase	1					1	0.307
		cgd7_2970	possible acyltransferase	1					1	0.438
		cgd7_3390	secreted alpha beta hydrolyse, signal peptide, secreted patatin like esterase (ralstonia best hits)				1		1	13.972
		cgd7_3730	long chain fatty acyl diphosphate synthase [Trans-Isoprenyl Diphosphate Synthase]	1					1	0.346
		cgd7_4590	Mth1. SpoU superfamily - SPOUT methylase				1		1	15.971
		cgd7_5250	methyltransfer with N-terminal ankyrin repeats				1		1	10.963
		cgd8_1700	NAD dependent dehydrogenase of possible bacterial origin	1					1	0.442
		cgd8_1970	NAD kinase involved in polyphosphate metabolism	1					1	0.541
		cgd8_2330	pyridine nucleotide/ NAD(P) transhydrogenase alpha plus beta subunits, duplicated gene, possible signal peptide plus 12 transmembrane regions				1		1	6.516
		cgd8_2410	Sey1p like P-loop GTpase of the RHD3 subfamily with a transmembrane domain near C-terminus	1					1	0.373
		cgd8_2670	secreted glucose methanol choline like oxidoreductase of the FAD dependent oxidoreductase like fold, signal peptide			1			1	3.668
		cgd8_2700	phenol 2-monooxygenase like FAD dependent oxidoreductase of plant origin in apicomplexans	1					1	0.349
		cgd8_3140	MRP like MinD family ATpase of the SIMIBI class of P-loop GTpases		1				1	1.958
		cgd8_3290	secreted bacterial type DHHB/UbiG like methyltransferase involved in ubiquinone/menaquinone biosynthesis, signal peptide		1				1	1.762
		cgd8_380	possible oxidase or dehydrogenase				1		1	7.001
		cgd8_3880	secreted alpha beta hydrolase family protein, signal peptide, duplicated adjacent gene	1					1	0.452
		cgd8_4130	secreted hydrolase superfamily, signal peptide	1					1	0.365
		cgd8_4230	FAD/NAD(P)-binding rossman fold oxidoreductase fused to a glucose-methanol-choline (GMC) oxidoreductase domain	1					1	0.529
		cgd8_5260	Srp101p GTpase. signal recognition particle receptor alpha subunit	1					1	0.361
		cgd8_590	alpha/beta hydrolase superfamily protein	1					1	0.469
		cgd8_600	secrted alpha/beta hydrolase superfamiy protein					1	1	114.509
	General Total			34	10	4	17	5	70	12.214
	Glycosylation	cgd1_230	ALG-2 like alpha-1,3 mannosyltransferase	1					1	0.356
		cgd2_1650	dolichyl-diphosphooligosaccharide-protein glycosyltransferase beta subunit (Wbp1p)	1					1	0.440
		cgd3_3590	dolichyl-phosphate-mannose-glycolipid alpha-mannosyltransferase involved in GPI anchor biosynthesis	1					1	0.290
		cgd4_2990	glycosyl transferase				1		1	10.754
		cgd4_3120	ALG6-like dolichyl pyrophosphate Man9GlcNAc2 alpha-1,3-glucosyltransferase	1					1	0.389
		cgd5_2040	dolichol phosphate mannose synthase, putative		1				1	1.321
		cgd5_2240	DPAGT1 like N-acetylglucosamine-1-phosphate transferase				1		1	11.320
		cgd5_2590	Alg5 like dolichyl-phosphate beta-glucosyltransferase	1					1	0.487
		cgd5_3140	LPS glycosyltransferase of possible cyanobacterial origin			1			1	4.376
		cgd5_3260	secreted lipopolysaccharide sugar transferase like family 8 glycosyltransferase	1					1	0.332
		cgd5_690	extracellular protein with a ricin domain and a family 2 glycosyltransferase domain		1				1	1.863
		cgd6_1450	secreted protein with signal peptide, fringe-like glycosyltransferase domain and a WcaK like glycosyltransferase domain			1			1	2.714
		cgd6_2040	oligosaccharyl transferase STT3 protein				1		1	20.928
		cgd6_5070	ribophorin I -like, putative dolichyl-diphosphooligosaccharide--protein glycosyltransferase 67kDa subunit precursor, signal peptide plus transmembrane domain at C-terminus	1					1	0.375
		cgd7_1810	ALG1 like beta-1,4 mannosyltransferase with possible signal peptide	1					1	0.455
		cgd7_4440	family 2 glycosyl transferase, possible fragment	1					1	0.354
		cgd7_4930	secreted glycosyltransferase, possible transmembrane domain near C-terminus			1			1	3.535

		cgd8_1420	secreted alpha glucosidase like family 31 glycosyltransferase, signal peptide	1					1	0.361
	Glycosylation Total			10	2	3	3		18	3.369
	Lipid	cgd1_1110	phosphatidylserine synthase I (serine-exchange enzyme I), 9x transmembrane domains		1				1	1.712
		cgd1_320	oxysterol binding protein			1			1	2.983
		cgd1_3290	carboxylesterase, putative				1		1	15.816
		cgd1_3380	phosphatidylinositol-glycan-class c, pigC, 8x transmembrane domains	1					1	0.542
		cgd1_3720	PIG-M mannosyltransferase,8 transmembrane domain	1					1	0.353
		cgd2_1090	diacylglycerol acyltransferase 1	1					1	0.409
		cgd2_1200	steroid reductase like intregral membrane protein with 4x transmembrane domains and an ubiquitin domain at its N-terminus	1					1	0.438
		cgd2_1340	possible phosphatidylinositol 3- and 4-kinase family protein		1				1	2.208
		cgd2_2890	putative biotin-(acetyl-CoA carboxylase) ligase	1					1	0.421
		cgd2_840	phosphatidylinositol N-acetylglucosaminyltransferase subunit PIG-P, involved in GPI anchor biosynthesis, multitransmembrane domain				1		1	7.314
		cgd3_2030	putative choline kinase	1					1	0.494
		cgd3_2100	phosphatidylserine decarboxylase, putative				1		1	17.060
		cgd3_2180	type I fatty acid synthase		1				1	1.573
		cgd3_2370	steroid dehydrogenase kik-i, putative	1					1	0.512
		cgd3_2630	diacylglycerol kinase	1					1	0.404
		cgd3_2870	putative acyl-CoA synthetases	1					1	0.517
		cgd3_2940	probable phosphatidylserine/phosphatidylglycerophosphate/cardiolipin synthase, 2x SMART_PLDc domains, possible bacterial origin	1					1	0.473
		cgd3_320	N-myristoyltransferase	1					1	0.440
		cgd3_640	acyl-CoA synthetase				1		1	15.090
		cgd4_2100	PIG-A like N-acetylglucosaminyl-phosphatidylinositol biosynthetic protein	1					1	0.413
		cgd4_2560	phospholipase C, delta 1 ortholog with 2 EF hands plus phospholipase C domain plus C2 domain				1		1	9.079
		cgd4_2580	oxysterol binding protein 1A-like pleckstrin homology (PH) domain containing protein	1					1	0.483
		cgd4_2590	glycerophosphodiester phosphodiesterase-like protein	1					1	0.329
		cgd4_2790	putative ethanolaminephosphotransferase (ETHPT) 9 transmembrane domain protein involved in lipid metabolism	1					1	0.561
		cgd4_2900	polyketide synthase			1			1	5.536
		cgd4_3400	putative long chain fatty acyl CoA synthetase having a signal peptide					1	1	61.470
		cgd5_1850	GPI1/PIG-Q like N-acetylglucosaminyl-phosphatidylinositol transferase involved in GIP anchor biosynthesis				1		1	17.338
		cgd5_2300	DAD1/Ost2 like dolichyl-diphospho-oligosaccharide-protein glycosyltransferase, epsilon unit				1		1	8.056
		cgd5_3070	acetylcholinesterase'secreted acetylcholinesterase of the alpha/beta hydrolase superfamily'	1					1	0.423
		cgd5_320	carboxylesterase , lysophospholipase, signal peptide				1		1	10.790
		cgd5_720	choline/ethanolamine kinase family protein		1				1	1.505
		cgd6_1270	SCT1/Gpt2p-like glycerol-3-phosphate acyltransferase signal peptide plus 3 transmembrane domain	1					1	0.509
		cgd6_2290	sphingomyelinase C precursor	1					1	0.405
		cgd6_3240	Gpi16p/PIG-T/SPBC1604.15 family; glycosyl phosphatidyl inositol 16 signal peptide and transmembrane domain or GPI anchor	1					1	0.409
		cgd6_3390	membrane associated protein with 2 transmembrane domains at the N-terminus and a phosphatidylinositol 4-kinase domain at the C-terminus	1					1	0.502
		cgd7_2950	phospholipid cytidyltransferase HIGH family	1					1	0.333
		cgd7_3480	cytidylyltransferase (HIGH family) exon-1			1			1	2.680
		cgd7_450	putative cytidine diphosphate-diacylglycerol synthase; integral membrane protein with 7 or more transmembrane domains					1	1	29.205
		cgd7_700	N-acetylglucosaminyl-phosphatidylinositolde-N-acetylase	1					1	0.404
		cgd8_1150	choline-phosphate cytidylyltransferase					1	1	185.504
		cgd8_2260	phosphatidylinositol glycan class O, integral membrane protein with signal peptide sequence and 12 or more transmembrane domains	1					1	0.428
		cgd8_2390	diacylglycerol kinase	1					1	0.428
		cgd8_3280	phosphatidylinositol-4-phosphate 5-kinase, putative				1		1	16.919
		cgd8_4500	phosphatidylinositol 4-kinase		1				1	1.434
		cgd8_4630	7 pass integral membrane proteinwith FLHWFHH motif shared with fatty-acyl elongase				1		1	8.236
		cgd8_560	CDP-diacylglycerol--inositol 3-phosphatidyltransferase isoform 1; phosphatidylinositol synthase; PtdIns synthase; PI synthase	1					1	0.434
	Lipid Total			25	5	3	10	3	46	9.404
	Nucleotide	cgd1_1900	Fur1p like uracil phosphoribosyltransferase	1					1	0.531
		cgd1_3140	P-loop nucleotide (UMP) kinase					1	1	39.507
		cgd2_1270	membrane associated adenylyl cyclase with 6 transmembrane regions and an adenylyl cyclase domain	1					1	0.436
		cgd2_1630	cytidine and deoxycytidylate deaminase family, putative	1					1	0.371
		cgd2_2780	dCMP deaminase, Dcd1p like	1					1	0.340
		cgd3_2320	cGMP phosphodiesterase A4				1		1	7.462

		cgd4_1890	adenosine monophosphate deaminase 2			1			1	4.073
		cgd4_4460	dihydrofolate reductase-thymidylate synthase	1					1	0.369
		cgd5_1290	membrane associated adenyl cyclase with 2 transmembrane regions and an adenylyl cyclase domain	1					1	0.555
		cgd5_1470	signal peptide containing protein having a NDK (nucleoside-diphosphate kinase) domain					1	1	1,562.515
		cgd5_1710	CTP synthetase (UTP-ammonia lyase)					1	1	89.058
		cgd5_3360	adenylate kinase (AMP - ADP; dAMP - dADP)					1	1	149.775
		cgd5_3630	thymidylate kinase	1					1	0.419
		cgd5_4440	thymidine kinase of likely bacterial origin		1				1	1.411
		cgd5_4520	GMP synthase		1				1	2.203
		cgd6_1570	apyrase; calcium-activated nucleotidase SCAN-1-like protein; signal peptide	1					1	0.414
		cgd6_1950	ribonucleotide-diphosphate reductase large chain; RIR1; c-terminal PFL-like glycyl radical enzymes-like fold		1				1	1.654
		cgd6_20	inosine-5-monophosphate dehydrogenase					1	1	51.213
		cgd6_500	membrane associated HD superfamily cyclic nucleotide phosphodiesterase domain containing protein	1					1	0.413
		cgd6_690	ribonucleotide reductase small subunit, duplicated adjacent gene	1					1	0.503
		cgd6_700	ribonucleotide reductase small subunit			1			1	3.098
		cgd7_2190	putative guanylate kinase		1				1	2.072
		cgd7_5170	deoxyuridinetriphosphatase, possible bacterial origin					1	1	170.965
		cgd8_2370	adenosine kinase like ribokinase (Adenosine - AMP)		1				1	2.053
		cgd8_2810	uridine kinase like P-loop NTpase	1					1	0.368
	Nucleotide Total			11	5	2	1	6	25	83.671
	Peptidase	cgd1_1680	insulinase like protease, signal peptide	1					1	0.588
		cgd1_3690	aspartyl (acid) protease, putative		1				1	2.119
		cgd1_370	carboxypeptidase probably secreted, signal peptide	1					1	0.391
		cgd1_3840	insulinase-like peptidase, telomeric gene			1			1	3.519
		cgd2_2480	methionine aminopeptidase, type II, putative , an1 domain				1		1	21.559
		cgd2_2760	peptidase'insulinase-like peptidase'	1					1	0.422
		cgd2_3320	secreted papain like protease, signal peptide	1					1	0.518
		cgd2_4270	secreted insulinase-like peptidase			1			1	2.496
		cgd2_710	predicted protease	1					1	0.508
		cgd2_920	peptidase'insulinase-like peptidase'	1					1	0.507
		cgd2_930	peptidase'insulinase-like peptidase'	1					1	0.447
		cgd3_2160	SprT like metalloprotease	1					1	0.491
		cgd3_2390	proliferation-associated protein 2G4 metalloprotease, creatinase/aminopeptidase fold				1		1	8.596
		cgd3_2920	possible peptidase family C54	1					1	0.314
		cgd3_3610	possible aspartyl aminopeptidase	1					1	0.342
		cgd3_4180	secreted insulinase like peptidase				1		1	11.207
		cgd3_4190	secreted insulinase like peptidase					1	1	99.822
		cgd3_4200	secreted insulinase like peptidase, signal peptide		1				1	2.078
		cgd3_4210	secreted insulinase like peptidase, signal peptide		1				1	1.548
		cgd3_4220	secreted insulinase like peptidase, signal peptide					1	1	32.433
		cgd3_4240	insulinase like peptidase			1			1	2.850
		cgd3_4250	secreted insulinase like peptidase, signal peptide				1		1	14.086
		cgd3_4260	peptidase'insulinase like peptidase'				1		1	25.027
		cgd3_4270	peptidase'insulinase like peptidase'				1		1	18.424
		cgd3_4280	secreted insulinase like peptidase, signal peptide			1			1	3.100
		cgd3_680	cathepsin like thiol protease possibly membrane associated, putative			1			1	4.611
		cgd4_2110	preprocathepsin c precursor, putative			1			1	4.293
		cgd4_2190	membrane associated aspartyl protease with a transmembrane domain at the C-terminus	1					1	0.390
		cgd4_2910	aminopeptidase			1			1	3.058
		cgd4_4120	metacaspase-like protein	1					1	0.586
		cgd4_4160	possible carboxypeptidase	1					1	0.322
		cgd4_4240	insulinase like peptidase			1			1	4.114
		cgd4_4430	OTU like cysteine protease				1		1	6.117
		cgd5_2810	ULP1 like chllamydin domain containing protease				1		1	6.714
		cgd6_2750	cysteine protease, putative				1		1	19.932
		cgd6_3550	endopeptidase, putative		1				1	1.348
		cgd6_3820	membrane bound aspartyl proteinase with a signal peptide plus transmembrane domain		1				1	1.784
		cgd6_4880	cryptopain - cysteine proteinase secreted, possible transmembrane domain near N-terminus	1					1	0.538
		cgd6_4990	predicted peptidase of the alpha/beta-hydrolase fold		1				1	1.502
		cgd6_5510	telomeric insulinase-like protease with signal peptide	1					1	0.260
		cgd6_5520	peptidase'insulinase like peptidase'	1					1	0.301
		cgd6_660	secreted pepsinogen like aspartyl protease having a signal peptide	1					1	0.491
		cgd6_900	SPAC25H1.04/CG16979-cterm -like; cysteine protease	1					1	0.304
		cgd7_140	SprT family metallopeptidase			1			1	2.677
		cgd7_2850	cathepsin like thiol protease possibly membrane associated		1				1	1.401
		cgd7_3020	rhomboid family membrane associated protease, 7 transmembrane domain					1	1	169.568

		cgd8_2720	insulinase like metalloprotease	1					1	0.389
		cgd8_3430	zincin/aminopeptidase N like metalloprotease				1		1	9.086
	Peptidase Total			19	7	9	10	3	48	10.275
	Protein folding	cgd1_800	protein disulfide isomerase, signal peptide plus possible ER retention motif	1					1	0.331
		cgd1_870	cyclophilin like peptidyl-prolyl cis-trans isomerase, signal peptide					1	1	42.272
		cgd2_1660	cyclophilin-like protein, putative	1					1	0.322
		cgd2_4120	20k cyclophilin, putative		1				1	1.479
		cgd6_120	disulfide-isomerase, signal peptide plus ER retention motif, putative ER protein		1				1	1.550
		cgd6_2690	macrophage infectivity potentiator (MIP);FKBP peptidyl-prolyl cis-trans isomerase-like fold, signal peptide + ER retention motif		1				1	1.525
		cgd6_3640	protein disulfide isomerase ; secreted protein with thioredoxin, signal peptide plus ER retention motif	1					1	0.357
		cgd7_210	fkbp					1	1	146.099
		cgd7_5100	protein disulfide isomerase, signal peptide plus ER retention motif					1	1	31.408
		cgd8_2350	cyclophilin-RNA interacting protein, putative		1				1	1.679
	Protein folding Total			3	4			3	10	22.702
	Protein kinase	cgd1_1220	S/T protein kinase		1				1	1.795
		cgd1_2630	Ser/Thr protein kinase	1					1	0.491
		cgd1_2850	protein kinase domain		1				1	2.000
		cgd1_810	protein kinase	1					1	0.640
		cgd1_890	protein kinase domain				1		1	7.703
		cgd2_1060	calcium/calmodulin dependent protein kinase with a kinas domain and 4 calmodulin-like EF hands					1	1	32.747
		cgd2_1300	calcium/calmodulin dependent protein kinase with a kinase domain and 4 calmodulin like EF hands		1				1	1.934
		cgd2_1880	protein kinase, putative		1				1	2.191
		cgd2_1960	mitogen-activated protein kinase 1, serine/threonine protein kinase, putative			1			1	4.731
		cgd2_3190	protein kinase, putative	1					1	0.445
		cgd2_3340	Ser/Thr protein kinase		1				1	1.447
		cgd2_3660	serine protease, subtilase family, signal peptide, putative	1					1	0.409
		cgd2_3890	Ser/Thr protein kinase	1					1	0.334
		cgd2_4340	mitogen-activated protein kinase 2, putative			1			1	3.086
		cgd3_1810	casein kinase I			1			1	5.040
		cgd3_1850	putative casein kinase II regulatory subunit; besthit Pf 23508244					1	1	39.442
		cgd3_2900	protein Roco7, putative	1					1	0.346
		cgd3_3030	MAPK, putative [Mitogen-activated protein kinase]	1					1	0.429
		cgd3_3040	protein kinase, cAMP-dependent, catalytic chain					1	1	27.906
		cgd3_3180	Ser/Thr protein kinase		1				1	1.779
		cgd3_3230	Ser/Thr protein kinase				1		1	6.811
		cgd3_3670	putative protein kinase CK2 regulatory subunit CK2B1				1		1	6.865
		cgd3_40	casein kinase I	1					1	0.478
		cgd3_920	calmodulin-domain protein kinase 1, putative					1	1	25.137
		cgd4_290	putative protein phosphatase 2A regulatory B subunit				1		1	5.922
		cgd4_3710	Ser/Thr protein kinase	1					1	0.426
		cgd4_990	Ser/Thr protein kinase	1					1	0.318
		cgd5_2270	maternal embryonic leucine zipper kinase					1	1	131.074
		cgd5_250	serine/threonine kinase-1				1		1	12.575
		cgd5_3180	Ser/Thr protein kinase	1					1	0.338
		cgd5_4390	Ser/Thr protein kinase	1					1	0.318
		cgd5_820	calcium/calmodulin dependent protein kinase with a kinase domain and 4 calmodulin like EF hands			1			1	4.439
		cgd6_3400	protein kinase			1			1	3.867
		cgd6_4840	serine protease, subtilase family, signal peptide	1					1	0.481
		cgd6_4960	serine/threonine-protein kinase, putative		1				1	1.809
		cgd6_5060	Ser/Thr protein kinase			1			1	2.548
		cgd6_520	Ser/Thr protein kinase			1			1	5.318
		cgd6_5240	protein kinase	1					1	0.634
		cgd6_620	casein kinase II, alpha subunit, putative					1	1	74.113
		cgd7_1190	Ser/Thr protein kinase					1	1	33.549
		cgd7_120	cAMP-dependent protein kinase regulatory subunit					1	1	50.411
		cgd7_1260	calcium/calmodulin dependent protein kinase with an EF hand N-terminal to the kinase domain and 4 calmodulin like EF hands at the C-terminus	1					1	0.441
		cgd7_1320	casein kinase II, alpha subunit, putative			1			1	2.430
		cgd7_1330	protein kinase with possible transmembrane region near N-terminus	1				1	2	22.162
		cgd7_1840	calcium/calmodulin-dependent protein kinase with a kinase domain and 4 calmodulin like EF hands				1		1	7.214
		cgd7_2000	Bub1p related protein kinase	1					1	0.379
		cgd7_2670	serine/threonine protein phosphatase, putative					1	1	173.545
		cgd7_3050	protein kinase			1			1	3.350
		cgd7_3430	protein kinase					1	1	49.044
		cgd7_3890	calcium-dependent protein kinase 7 (CDPK)(CPK7)	1					1	0.280

		cgd7_40	calcium/calmodulin-dependent protein kinase with a kinase domain and 2 calmodulin-like EF hands	1					1	0.525
		cgd7_440	RIO-like kinase domain; N-terminal region conserved					1	1	126.665
		cgd7_590	Ser/Thr protein kinase	1					1	0.386
		cgd8_1230	proteine kinase	1					1	0.354
		cgd8_1660	protein kinase		1				1	2.308
		cgd8_2430	Ser/Thr protein kinase with MORN repeats at the N-terminus and a sterile alpha motif (SAM_ domain	1					1	0.381
		cgd8_3070	serine/threonine protein kinase KKIALRE			1			1	3.836
		cgd8_5120	protein kinase NPK2	1					1	0.349
		cgd8_5320	Ser/Thr protein kinase				1		1	7.371
		cgd8_750	cyclic nucleotide (cGMP)-dependent protein kinase with 3 cNMP binding domains and a Ser/Thr kinase domain	1					1	0.389
	Protein kinase Total			24	8	10	7	12	61	15.179
	Protein modification	cgd1_1980	protein geranyl-geranyltransferase beta subunit			1			1	4.136
		cgd1_2660	n-terminal acetyltransferase complex ard1			1			1	3.864
		cgd1_730	glutamine cyclotransferase, predicted bacterial/plant origin	1					1	0.399
		cgd1_820	DHHC family palmitoyl transferases with 4 transmembrane domains	1					1	0.368
		cgd2_1460	SUMO-1 activating enzyme subunit 2, putative	1					1	0.373
		cgd2_2190	DHHC family palmitoyl transferase fused to a KOW domain, signal peptide				1		1	6.329
		cgd2_30	cysteine desulfurase/selenocysteine lyase-like PLP dependent transferase superfamily protein			1			1	4.312
		cgd3_2350	possible RAB geranylgeranyl transferase, b subunit1, pernyltransferase (prenyltransferase)			1			1	4.366
		cgd3_4310	sexual stage-specific protein kinase		1				1	1.666
		cgd4_2080	DHHC family palmitoyl transferase with 4 transmembrane regions	1					1	0.439
		cgd5_3770	putative arginine N-methyltransferase					1	1	34.651
		cgd5_430	70 kDa peptidylprolyl isomerase, putative	1					1	0.555
		cgd6_2510	farnesyltransferase	1					1	0.352
		cgd7_1560	DHHC family palmitoyl transferase with a signal peptide and 4 transmembrane domains		1				1	1.961
		cgd8_1490	bacterial type serine/tyrosine phosphatase				1		1	13.749
		cgd8_2010	span like RimI family protein amino acetyltransferase		1				1	1.395
		cgd8_4730	DHHC family palmitoyl transferase with a signal peptide and 4 transmembrane domains		1				1	1.486
		cgd8_4760	arginine n-methyltransferase					1	1	29.544
	Protein modification Total			6	4	4	2	2	18	6.108
	Protein phosphatase	cgd1_2120	protein phosphatase regulator like heat repeats		1				1	1.665
		cgd1_2360	protein phosphatase 4 (formerly X), catalytic subunit; Protein phosphatase 4, catalytic subunit					1	1	53.151
		cgd1_2470	phosphotyrosyl phosphatase activator protein-related	1					1	0.443
		cgd1_760	dual specificity phosphatase					1	1	86.436
		cgd2_1640	calcineurin like phosphatase with 3x Efhand domains at N-terminus	1					1	0.441
		cgd2_2960	phosphoprotein phosphatase related, putative			1			1	5.836
		cgd2_640	possible conserved acid phosphatase		1				1	1.981
		cgd3_1470	dual specificity phosphatase	1					1	0.423
		cgd3_1890	membrane bound phosphatase with 3 transmembrane domains and a dual specificity phosphatase	1					1	0.486
		cgd3_2020	PP2C like protein phosphatase	1					1	0.430
		cgd3_2150	protein phosphatase, signal peptide, 2-6 transmembrane domain protein	1					1	0.366
		cgd3_250	protein serine/threonine phosphatase alpha	1					1	0.464
		cgd6_1010	PP2C like protein phosphatase	1					1	0.503
		cgd6_3570	myotubularin related protein 1, C-terminus protein tyrosine/serine phosphatase, catalytic domain	1					1	0.442
		cgd6_4200	protein phosphatase 2B catalytic subunit, calcineurin like phosphatse superfamily		1				1	2.359
		cgd6_440	PP2Cc like protein phosphatase	1					1	0.326
		cgd6_5000	phosphoprotein phosphatase 2A 65K regulatory chain-like with HEAT repeats					1	1	56.488
		cgd7_3530	PP2C-like phosphatase	1					1	0.473
		cgd7_4150	PP2C phosphatase		1				1	2.050
		cgd7_4640	PP2C phosphatase					1	1	248.430
		cgd7_4790	Ptc7p phosphatase (PP2C family)					1	1	333.117
		cgd7_4970	protein phosphatase 2A regulatory subunit (contains a conserved version of EF hands)		1				1	1.630
		cgd7_810	protein phosphatase PP2A, calcineurin like phosphoesterase superfamily	1					1	0.453
		cgd8_1430	PP2C like protein phosphatase			1			1	2.709
		cgd8_290	serine-threonine protein phosphatase	1					1	0.432
		cgd8_3180	protein phosphatase regulator like HEAT repeats containing protein that folds into a alpha-alpha superhelix			1			1	2.474
	Protein phosphatase Total			13	5	3		5	26	30.923
	Sugar & derivatives	cgd1_3060	oligosaccharide deacetylase domain	1					1	0.552
		cgd2_1770	mannose-1-phosphate guanylyltransferase	1					1	0.534
		cgd2_1900	dTDP-glucose 4-6-dehydratase-like protein, putative		1				1	1.749
		cgd4_2600	UDP-glucose 4-epimerase			1			1	2.593

		cgd4_810	UDP-N-acetylglucosamine pyrophosphorylase	1					1	0.395
		cgd4_960	phosphomannomutase	1					1	0.323
		cgd6_3750	1,4-alpha-glucan branching enzyme; alpha-amylase; glycogen branching enzyme	1					1	0.537
		cgd7_1830	secreted UDP-N-acetylglucosamine pyrophosphorylase family protein, signal peptide	1					1	0.471
		cgd7_2580	AMSJ/WSAK like polysaccharide polymerase (glycosyltransferase family)	1					1	0.548
		cgd7_4120	UDP-N-acetylgalactosamine: polypeptide N-acetylgalactosaminyltransferase, signal peptide	1					1	0.509
		cgd8_4940	trehalose-6-phosphate synthase of likely plant origin	1					1	0.515
		cgd8_920	UDP-glucose 6-dehydrogenase	1					1	0.338
	Sugar & derivatives Total			10	1	1			12	0.755
**Enzyme Total**				170	60	50	62	47	389	25.786
**Gene expression**	RNA synthesis	cgd1_1620	RPB7 like OB fold RNA polymerase subunit			1			1	3.826
		cgd1_1960	RPB7 subunit of the RNA polymerase III, OB fold	1					1	0.379
		cgd1_2260	RNA polymerase II B8 subunit		1				1	1.944
		cgd1_2710	RNA polymerase II B3 subunit			1			1	2.583
		cgd1_2770	RNA polymerase 1 beta subunit					1	1	114.168
		cgd2_2970	DNA-directed RNA polymerase II, putative				1		1	20.205
		cgd2_730	RNA polymerase III transcription factor (TF)IIIC subunit tau/Tfc1p	1	1				2	1.766
		cgd2_980	putative DNA-directed RNA polymerase 2				1		1	21.906
		cgd2_990	queuine tRNA-guanine transglycosylase					1	1	36.009
		cgd3_1870	DNA-directed RNA polymerase 2 subunit		1				1	1.560
		cgd3_2620	putative DNA-directed RNA polymerase,possible RNA polymerase A/beta'/A" subunit, long PHYSPTS repeat at C-terminus					1	1	28.707
		cgd4_3260	putative DNA-directed RNA polymerases I, II, and III 8.3 kda polypeptide				1		1	17.908
		cgd5_730	DNA-directed RNA polymerase I, putative				1		1	5.562
		cgd6_3290	DNA-directed RNA polymerase III C1 subunit			1			1	4.484
		cgd6_4580	RNA polymerase III C34 subunit; rpc34p ortholog				1		1	16.501
		cgd7_3720	RNA polymerase beta subunit			1			1	3.276
		cgd7_4250	RNA pol II carboxy terminal domain phosphatase of the HAD superfamily with a BRCT domain at the C-terminus				1		1	18.369
		cgd7_4370	RNA polymerase II, putative		1				1	1.250
		cgd7_4390	NudC ortholog		1				1	1.897
		cgd7_4770	DNA-directed RNA polymerase subunit					1	1	72.834
		cgd7_510	RNA polymerase III subunit C11		1				1	1.950
		cgd8_170	DNA-directed RNA polymerase beta subunit			1			1	5.245
		cgd8_300	RNA polymerase III C5 subunit					1	1	82.368
		cgd8_3400	RNA polymerase II CTD/NL1 interacting protein like phosphatase	1					1	0.469
		cgd8_4810	RNA pol II carboxy terminal domain phosphatase of the HAD superfamily with a BRCT domain at the C-terminus	1					1	0.348
	RNA synthesis Total			4	6	5	6	5	26	17.972
	Transcription	cgd1_1560	domain KOG2577, transcription factor E2F/dimerization partner (TDP)					1	1	81.378
		cgd1_1570	transcription factor E2F					1	1	111.751
		cgd1_1700	GATA type DNA binding domain fused to an AT hook		1				1	2.221
		cgd1_2450	ELP3 like acetyltransferase involved in transcription					1	1	30.452
		cgd1_2600	transcription factor TAF5p, TBP associated protein involved in transcription	1					1	0.486
		cgd1_3620	transcription factor TAF7p/TAFII55			1			1	3.834
		cgd1_3780	domain KOG1015, transcription regulator XNP/ATRX, DEAD-box superfamily, signal peptide			1			1	3.057
		cgd2_1760	gata/ArfGAP, putative			1			1	3.512
		cgd2_2060	possible transcription factor TFIIH			1			1	3.906
		cgd2_220	transcription factor TFIIF		1				1	2.172
		cgd2_2820	transcription factor, putative			1			1	2.405
		cgd2_3420	programmed cell death 2, putative				1		1	9.343
		cgd3_1120	SNAPc like transcription factor with 4 MYB/SANT domains				1		1	6.242
		cgd3_3110	transcription factor TFIIH with a vWA domain	1					1	0.454
		cgd3_3250	CCAAT-binding factor (CBF)/MAK21 family protein				1		1	11.631
		cgd3_3750	multiprotein bridging factor type 1 like transcriptional co-activator			1			1	2.464
		cgd3_4060	histone transcription regulator (HIR1)-like WD40 repeat protein		1				1	1.489
		cgd3_4150	cutinase negative acting protein, putative					1	1	315.246
		cgd3_760	HMG-like nuclear protein, Nhp2p, pelota RNA binding domain containing protein					1	1	105.529
		cgd4_1270	transcription factor TFIIIB, SANT domain			1			1	3.689
		cgd4_1590	chromodomain-helicase-DNA-binding'multidomain chromatin protein with the following architecture: chromo-bromo-chromo-SNF2 ATPase'		1				1	1.472
		cgd4_1720	CCAAT-box DNA binding protein subunit B		1				1	1.824
		cgd4_310	CCAAT-binding factor chain HAP5 like histone	1					1	0.345
		cgd4_3810	putative transcription factor IIE alpha subunit	1	1				2	1.123
		cgd4_710	Ynr054cp, basal transcriptional activator hABT1, RRM domain containing protein				1		1	5.676

		cgd4_880	ADA2 ortholog with a ZZ finger, SANT domain and a SWIRM domain					1	1	33.563
		cgd5_1280	tfc4p like TFIIIC subunit; TPR repeat containing basal transcription factor		1				1	1.677
		cgd5_1690	cactin-like protein		1				1	1.400
		cgd5_3290	transcription elongation factor TFIIS					1	1	69.364
		cgd5_3790	SNF7 ortholog				1		1	12.616
		cgd5_4200	transcription initiation factor IIA	1					1	0.309
		cgd5_670	reptin52; reptin like TIP49 family AAA+ ATpase			1			1	4.952
		cgd6_1430	transcription factor E2F wHTH only					1	1	169.529
		cgd6_2430	BTF domain, basal transcription factor					1	1	94.671
		cgd6_2480	CCR4-NOT transcription complex, subunit 2; NOT2. C terminal Not2/Not3 domains			1			1	2.929
		cgd6_3860	SNF2 helicase, putative			1			1	3.447
		cgd6_4650	5kows transcription initiation protein SPT5			1			1	4.155
		cgd6_5250	transcription elongation factor, SPT6-like				1		1	10.880
		cgd7_3240	transcription activator, putative					1	1	34.500
		cgd7_3410	SNF5 like protein	1		1			2	1.981
		cgd7_3650	DP1 DNA binding protein					1	1	91.275
		cgd8_1850	E2F like domain containing transcription factor with the wHTH fold					1	1	63.696
		cgd8_2030	TATA-box factor binding protein					1	1	77.689
		cgd8_210	transcription factor TFIID, TBP			1			1	4.473
		cgd8_2420	transcription initiation factor IIF/ Rap30 like winged HTH	1					1	0.353
		cgd8_2770	SNF2L ortholog with a SWI/SNF2 like ATpase and a Myb domain				1		1	13.645
		cgd8_4400	transcription elongation factor TFIIS			1			1	2.778
		cgd8_880	transcription initiation factor TFIIB Sua7p; ZnR+2cyclins			1			1	2.575
	Transcription Total			7	8	15	7	13	50	28.345
	Translation	cgd1_1860	GCN3p like EIF2B,translation initiation factor eIF-2B alpha subunit			1			1	3.932
		cgd1_2420	SUA5 like RNA binding domain containing protein					1	1	100.769
		cgd1_880	eukaryotic initiation factor 4A (eIF4A) (eIF-4A)					1	1	889.383
		cgd2_110	Dbp6p, eIF4a-1 family RNA SFII helicase					1	1	28.753
		cgd2_1160	HSPC021/HSPC025 family protein			1			1	2.508
		cgd2_1500	possible translation initiation factor with possible PINT domain		1				1	1.710
		cgd2_2070	translation elongation factor EF-1, subunit alpha, putative				1		1	7.846
		cgd2_2430	ximpact ortholog conserved protein seen in bacteria and eukaryotes			1			1	2.767
		cgd2_270	translation initiation factor eIF-3 subunit 7			1			1	4.994
		cgd2_360	prtip-like IF39 eukaryotic translation initiation factor 3				1		1	7.079
		cgd2_3920	eIF4G eukaryotic initiation factor 4, Nic domain containing protein			1			1	2.630
		cgd2_3930	deoxyhypusine synthase/spermidine:eIF5A-lysine 4-aminobutyltransferase				1		1	14.982
		cgd2_3950	putative translation elongation factor 1 beta 1		1				1	2.292
		cgd2_820	putative translation initiation factor 1 (eIF1), SUI1p					1	1	178.680
		cgd3_1650	eIF2G GTpase. eukaryotic translation initiation factor 2 gamma subunit (ZnR+GTpase)			1			1	3.535
		cgd3_280	mRNA translation inhibitor SKI2 SFII helicase, DEXDc+HELICc	1					1	0.332
		cgd3_3020	possible guaning nucleotide exchange factor, eIF-2B				1		1	15.514
		cgd3_3340	SECIS binding protein. pelota RNA binding domain containing protein					1	1	85.730
		cgd3_3380	Dbp9p, eIF4A-1-family RNA SFII helicase, DEXDc+HELICc				1		1	16.378
		cgd3_4020	eIF-3 p25/subunit 11				1		1	9.589
		cgd4_1030	eukaryotic translation initiation factor			1			1	3.984
		cgd4_1840	Dbp7p, eIF4A-a-family RNA SFII helicase (DEXDc+HELICc)				1		1	14.212
		cgd4_3130	putative eIF6, translation initiation factor 6					1	1	55.583
		cgd4_3180	Rrp3p, eIF4A-1-family RNA SFII helicase (DEXDc+HELICc)			1			1	5.396
		cgd5_1600	translation initiation factor if-2B beta; eIF-2			1			1	4.386
		cgd5_390	putative eukaryotic translation initiation factor 3			1			1	3.508
		cgd5_680	eukaryotic translation initiation factor 3 37.28 kDa subunit, putative			1			1	2.863
		cgd6_1020	translation initiation factor if-4E		1				1	1.433
		cgd6_1200	diphthine synthase; diphthamide biosynthesis methyltransferase				1		1	8.786
		cgd6_1590	eukaryotic translation initiation factor 4 gamma; Nic domain containing protein				1		1	13.961
		cgd6_2180	Fun12p GTpase; translation initiation factor IF2					1	1	35.952
		cgd6_2230	diphthamide biosynthesis; OVCA1/DPH2 protein-like; AE group (Note: old=diphtamide; Diphthamide is a modified histidine in eEF-2)			1			1	4.029
		cgd6_2260	Hca4p helicase DBP4 (helicase CA4). EIF4A-1-family RNA SFII helicase				1		1	8.122
		cgd6_3210	Rok1p, eIF4A-1-family RNA SFII helicase				1		1	18.835
		cgd6_3380	Erf1 eukaryotic translation termination factor 1; N-terminal RNAseH plus pelota domain containing protein			1			1	4.351
		cgd6_3420	translation initiation factor EIF-2B epsilon subunit	1					1	0.496
		cgd6_3960	elongation factor-like protein				1		1	13.004
		cgd6_3990	elongation factor 1 alpha					1	1	156.058
		cgd6_4830	Drs1p, eIF4a-1-family RNA SFII helicase			1			1	3.459

		cgd6_4860	nucleolar protein GU2. eIF4A-1-family. RNA SFII helicase					1	1	354.202
		cgd7_1080	eIF3-p47 with JAB/PAD domain			1			1	3.133
		cgd7_2300	translation initiation factor if-5A					1	1	1,056.848
		cgd7_2430	translation initiation factor eIF-5; Tif5p, ZnR+W2 domains					1	1	1,025.727
		cgd7_3940	eIF4A-1; eukaryotic translation initiation factor 4A-1; RNA SFII helicase		1				1	1.640
		cgd7_5180	eIF3 gamma/P40 with JAB/PAD domains; translation initiation factor IF-3 subunit 3				1		1	18.624
		cgd7_5270	Translation initiation factor 2, alpha subunit(eIF2-alpha); S1-like RNA binding domain			1			1	3.336
		cgd7_880	eIF-3A like translation initiation factor that has a WD40 repeat-containing beta propeller	1					1	0.415
		cgd8_2930	Eft2p GTpase; translation elongation factor 2 (EF-2)					1	1	35.996
		cgd8_3940	eIF-2B gamma, eukaryotic translation initiation factor 2B subunit 3 that has a nucleotide diphospho sugar transferase at the N-terminus and a UDP N-acetylglucosamine acyltransferase at the C-terminus	1					1	0.379
		cgd8_4430	translation initiation factor if-2 betam beta subunit ZnR				1		1	20.879
		cgd8_4750	Dbp5p-like eIF4A-1-family RNA SFII helicase				1		1	9.068
		cgd8_800	Prp5p C terminal KH. eIF4A-1-family RNA SFII helicase	1					1	0.342
	Translation Total			5	4	16	15	12	52	82.085
**Gene expression Total**				16	18	36	28	30	128	48.070
**General**	General	cgd1_2980	14-3-3 protein	1					1	0.623
		cgd2_1700	2x Ef hands, calmodulin-like	1					1	0.511
		cgd2_810	calmodulin					1	1	53.413
		cgd3_1290	14-3-3 domain containing protein		1				1	1.619
		cgd6_730	14-3-3 domain containing protein	1					1	0.386
		cgd7_2470	14-3-3 domain containing protein	1					1	0.337
		cgd7_3950	2*FF domain protein (phosphopeptide binding)	1					1	0.310
	General Total			5	1			1	7	8.171
**General Total**				5	1			1	7	8.171
**Membrane**	Membrane traffic	cgd1_1120	epsin like ENTH domain (alpha-alpha superhelix)involved in vesicular transport		1				1	1.560
		cgd1_1340	VDP/USO1/YBL047C family vesicular transport factor	1					1	0.464
		cgd1_1770	putative Rab5-interacting protein				1		1	9.815
		cgd1_1830	TB2/DP1/HVA22 family integral membrane protein that may be involved in membrane trafficking, 3x transmembrane domains					1	1	69.400
		cgd1_1890	vesicle transport protein, putative	1					1	0.533
		cgd1_200	ADP-ribosylation factor-like protein 2 (ARL2), putative	1					1	0.413
		cgd1_2060	Rab2 GTPase, putative	1					1	0.478
		cgd1_2580	Arl1p/ARF like GTpase involved in vesicular transport	1					1	0.343
		cgd1_2830	syntaxin 5A ortholog, possible transmembrane domain or GPI at C-terminus	1					1	0.370
		cgd1_2920	golgi transport SNARE BOS1 secretory pathway protein, possible transmembrane or GPI anchor at C-terminus		1				1	1.551
		cgd1_580	dynamin-like protein			1			1	2.707
		cgd2_1140	syntaxin, putative, SMART syntaxin+tSNARE+transmembrane domain or GPI anchor at C-terminus	1					1	0.376
		cgd2_1570	putative vacuolar protein sorting/targeting protein 26		1				1	1.488
		cgd2_1940	RAB6 protein, putative	1					1	0.331
		cgd2_3010	N-ethylmaleimide-sensitive factor (NSF1)-like AAA ATpase involved in vesicular transport	1					1	0.365
		cgd2_3400	dynamin-related protein, putative	1					1	0.485
		cgd2_3410	ARF1/2 like small GTpase, putative				1		1	20.351
		cgd3_1070	synaptobrevin like SNARE	1					1	0.515
		cgd3_180	domain KOG1666, V-SNARE (intracellular trafficking, secretion, and vesicular transport)			1			1	4.264
		cgd3_1820	putative Sec23			1			1	2.720
		cgd3_3150	Rab5 like small GTpase	1					1	0.401
		cgd4_2930	vacuolar sorting protein VPS52/suppressor of actin Sac2-family protein	1					1	0.316
		cgd4_3420	Vps53-N family protein involved invacuolar protein sorting	1					1	0.453
		cgd4_4300	beta adaptin			1			1	2.884
		cgd5_3850	adapter-protein complex 1 gamma subunit (gamma adaptin)	1					1	0.324
		cgd5_4180	vacuolar protein-sorting protein VPS45, putative	1					1	0.302
		cgd6_150	synaptobrevin'synaptobrevin, adjacent duplicated gene'				1		1	11.762
		cgd6_2070	adaptin AP complex subunit alpha; ARM repeats	1					1	0.346
		cgd6_3220	RAS small GTpases RIC1/ypt1		1				1	1.598
		cgd6_4930	beta-adaptin AP complex subunit-related; ARM/HEAT repeat protein	1					1	0.419
		cgd6_510	synaptobrevin/VAMP-like protein				1		1	5.863
		cgd7_1680	Rab7 GTPase, putative			1			1	3.055
		cgd7_1820	BET3 vesicular transport protein	1					1	0.313
		cgd7_1910	coatomer SEC21 gamma subunit like (beta adaptin)	1					1	0.486
		cgd7_2060	vacuolar protein sorting 29	1					1	0.388
		cgd7_2940	coatomer complex delta chain	1					1	0.347
		cgd7_3130	ER vesicle protein; Erv41p, transmembrane region near C terminus and possible N region transmembrane		1				1	2.014
		cgd7_4210	Sec61-gamma subunit of protein translocation complex, putative		1				1	1.396

		cgd7_4380	Rablib [rab11b]	1					1	0.352
		cgd7_4740	Rab GDP dissociation inhibitor	1					1	0.367
		cgd7_5010	coatomer complex beta	1	1				2	0.986
		cgd7_5300	Rab geranylgeranyltransferase, alpha subunit	1					1	0.360
		cgd7_5460	VPS16 vacuolar sorting protein		1				1	1.701
		cgd7_670	t-SNARE domain followed by hydrophobic stretch	1					1	0.444
		cgd8_1080	STXBP/UNC-18/SEC1 syntaxin involved in golgi transport	1					1	0.427
		cgd8_1250	SEC24C-like component of COPII coatamer of ER-golgi vesicles	1					1	0.491
		cgd8_1270	clathrin heavy chain		1				1	1.801
		cgd8_1640	RAB acceptor, possible 2 or more transmembrane domains	1					1	0.352
		cgd8_1910	epsin like ENTH/VHS domain involved in endocytosis/vesicular trafficking			1			1	5.178
		cgd8_1990	dynamin like TRAFAC class GTpase domain	1					1	0.420
		cgd8_2630	APG10/ Aut1p like like autophagocytosis protein involved in vacuolar transport			1			1	3.449
		cgd8_2900	ERV41 like membrane associated protein involved in vesicular transport with a transmembrane region near the C-terminus		1				1	1.640
		cgd8_2920	Vps60p /Vps20p like protein involved in vacuolar protein sorting	1					1	0.390
		cgd8_4040	Aps1p/AP17 like clathrin adaptor protein	1					1	0.371
		cgd8_860	coatomer protein complex subunit alpha, putative				1		1	6.262
		cgd8_910	vacuolar membrane protein pep3, human vacuolar protein sorting 18-like protein with a metal binding domain at the C-terminus				1		1	6.480
		cgd8_950	clathrin coat assembly protein AP50	1					1	0.389
	Membrane traffic Total			34	10	7	6	1	58	3.173
	Mucin	cgd1_3550	mucin-like low complexity glycoprotein with a signal peptide and an apple domain.	1					1	0.402
		cgd2_400	signal peptide, serine stretches, possible low mw mucin glycoprotein locus of 6 genes					1	1	78.202
		cgd2_410	signal peptide, serine stretches, possible low mw mucin glycoprotein, locus of 6 genes					1	1	48.299
		cgd2_420	signal peptide, serine proline rich, possible low mw mucin glycoprotein locus of 6 genes					1	1	251.769
		cgd2_430	signal peptide, serine proline rich, possible low mw mucin glycoprotein locus of 6 genes					1	1	1,732.542
		cgd2_440	signal peptide, serine proline rich, possible low mw mucin glycoprotein locus of 6 genes				1		1	21.108
		cgd2_450	signal peptide, serine proline rich, possible low mw mucin glycoprotein locus of 6 genes			1			1	2.636
		cgd3_440	C-type lectin containing protein with a transmembrane domain and mucin-like rich regions		1				1	1.791
		cgd4_3550	secreted protein with signal peptide and 12 KAZAL repeats and a mucin-like stretch of threonines					1	1	495.075
		cgd5_1210	hypothetical protein with Thr stretches, possible mucin				1		1	13.191
		cgd5_2060	hypothetical protein with signal peptide and mucin-like threonine repeats					1	1	493.875
		cgd6_5400	protein with signal peptide plus thr stretch, cys rich, possible mucin					1	1	837.635
		cgd6_5410	signal peptide plus thr stretch, charged repeats, likely mucin					1	1	1,210.260
		cgd7_4020	cryptopsoridial mucin, large thr stretch, signal peptide sequence					1	1	40.935
		cgd7_4660	secreted mucin like glycoprotein, signal peptide, thr stretch		1				1	2.207
		cgd8_3520	secreted protein with cysteine rich repeats and a mucin like threonine rich repeat, signal peptide					1	1	864.971
	Mucin Total			1	2	1	2	10	16	380.931
	TRAP	cgd1_3500	thrombospondin related adhesive protein			1			1	4.454
		cgd1_3510	TSP1 domain-containing protein TSP3 precursor		1				1	1.937
		cgd5_3420	TRAP-C2 extracellular protein	1					1	0.425
	TRAP Total			1	1	1			3	2.272
**Membrane Total**				36	13	9	8	11	77	81.633
**Mitochondrial**	Enzyme	cgd3_3120	AOX1,alternative oxidase, possible fungal or bacterial origin, 2 transmembrane regions	1					1	0.403
		cgd4_3040	NifS-like protein; cysteine desulfurase				1		1	17.058
		cgd5_3400	mitochondrial processing peptidase beta subunit		1				1	1.844
		cgd6_1050	IscU-like NifU protein, iron-sulfur protein					1	1	89.806
		cgd7_1900	mitochondrial NADH dehydrogenase				1		1	24.182
		cgd7_2080	mitochondrial processing peptidase, insulinase like metalloprotease		1				1	1.464
	Enzyme Total			1	2		2	1	6	22.460
	F-ATPase	cgd2_1360	ATP synthase beta chain, mitochondrial precursor, putative					1	1	351.081
		cgd6_610	ATP synthase alpha chain			1			1	4.079
	F-ATPase Total					1		1	2	177.580
	Membrane protein	cgd3_3560	CorA family mitochondrial membrane protein with 2 transmembrane domains at C-terminus	1					1	0.393
	Membrane protein Total			1					1	0.393
	Mito carrier protein	cgd2_1030	mitochondrial carrier protein, putative			1			1	3.194
		cgd6_2350	mitochondrial carrier protein, 4 transmembrane domain				1		1	13.061
		cgd6_2360	mitochondrial FAD carrier protein	1					1	0.513
		cgd6_3880	mitochondrial carrier protein MRS2 with 2 transmembrane domains				1		1	9.244
	Mito carrier protein Total			1		1	2		4	6.503

	Translocase	cgd1_2560	Tom40p like translocase	1					1	0.335
		cgd2_4140	mitochondrial import inner membrane translocase subunit tim17			1			1	2.460
		cgd2_520	mitochondrial phosphate translocator			1			1	4.904
		cgd4_3170	putative mitochondrial inner membrane translocase		1				1	1.740
	Translocase Total			1	1	2			4	2.360
**Mitochondrial Total**				4	3	4	4	2	17	30.927
**Protein degradation**	Proteosome	cgd1_2490	proteasome subunit beta type 1, putative					1	1	30.539
		cgd1_2890	beta transducin ortholog with WD40 repeats				1		1	27.921
		cgd1_3480	predicted proteasome regulatory complex component with a PINT domain at the C-terminus			1			1	4.194
		cgd1_420	20S proteasome beta subunit D2 (PBD2) , putative					1	1	168.663
		cgd2_1020	proteasome regulatory subunit Rpn9, PINT domain			1			1	2.890
		cgd2_1350	26S proteasome regulatory subunit, S6a like AAA ATpase		1				1	2.150
		cgd2_1440	putative proteasome regulatory subunit, NTN hydrolase fold	1					1	0.394
		cgd2_2210	proteasome regulatory subunit Rpn7/26S proteasome subunit 6, PINT domain containing protein				1		1	6.435
		cgd2_3370	proteasome regulatory subunit Rpn12 family	1					1	0.520
		cgd2_860	Pre3p/proteasome regulatory subunit beta type 6, NTN hydrolase fold	1					1	0.562
		cgd3_2170	proteasome subunit alpha1	1					1	0.529
		cgd3_2530	PUP1/proteasome subunit beta type 7, NTN hydrolase fold			1			1	3.081
		cgd4_1170	26S proteasome regulatory subunit S4 like AAA ATpase	1					1	0.540
		cgd4_250	proteasome subunit alpha type 4, NTN hydrolase fold				1		1	10.631
		cgd4_2540	26S proteasome regulatory subunit 26b like AAA ATpase				1		1	14.557
		cgd4_530	26S proteasome regulatory subunit 5a with a vWA domain and two ubiquitin interacting motifs (UIM)	1					1	0.385
		cgd5_2360	p27 like 26S proteasomal subunit with a PDZ domain	1					1	0.464
		cgd5_4210	Pre2p/proteasome subunit beta type 5; NTN hydrolase fold	1	1				2	1.051
		cgd6_2300	Cut4/Apc1p/TSG24 family protein; meiotic check point regulator and 26S proteasome regulatory complex; PC-rep repeats	1					1	0.265
		cgd6_3270	26S proteasome-associated Mov34/MPN/PAD-1 family. JAB domain.	1					1	0.488
		cgd6_3590	26S proteasome regulatory subunit Rpn6-like; PINT domain containing protein	1					1	0.393
		cgd6_4350	26S proteasome regulatory subunit 7 (RPT1)-like. AAA atpase	1					1	0.408
		cgd6_920	26s protease regulatory subunit 8, putative	1					1	0.420
		cgd7_2900	26S proteasome regulatory subunit, inactive JAB domain protein			1			1	4.459
		cgd7_3660	proteasome subunit alpha2, protease of the acylase family and NTN hydrolase fold					1	1	41.786
		cgd8_3090	proteasome activator p28/ Ki autoantigen				1		1	20.335
		cgd8_4060	26S proteasomal subunit S3; PINT domain containing protein	1					1	0.550
		cgd8_630	26S proteasome regulatory complex, subunit PSMD5				1		1	7.003
	Proteosome Total			14	2	4	6	3	29	12.161
	Ubiquitin	cgd1_1170	ubiquitin C-terminal hydrolase		1				1	1.448
		cgd1_1530	Rpn5 like 26S proteasomal regulatory subunit 12, PINT domain containing protein		1				1	1.535
		cgd1_1800	transducin / WD-40 repeat protein family	1					1	0.395
		cgd1_290	Ub6p like ubiquitin at N-terminus and ubiquitin C terminal hydrolase at the C-terminus	1					1	0.336
		cgd1_410	ubiquitin conjugating enzyme	1					1	0.304
		cgd1_860	ubiquitin-like protein, putative			1			1	4.694
		cgd2_3450	ubiquitin C-terminal hydrolase of the cysteine proteinase fold	1					1	0.549
		cgd3_2190	ubiquitin domain containing protein with a UBA domain at the C-terminus				1		1	6.636
		cgd3_2410	ubiquitin-fusion degadation-2 (UFD2) family protein with a UBOX at the C-terminus	1					1	0.432
		cgd3_2460	UbiA prenyltransferase family member (3I27) , putative		1				1	1.711
		cgd3_2670	protein with UBC domain, ubiquitin conjugating enzyme E2		1				1	2.143
		cgd4_1200	ubiquitin fusion degradation protein (UFD1); double Psi beta barrel fold			1			1	3.823
		cgd4_210	Ubc1p like ubiquitin-conjugating enzyme E2 fused to a UBA domain (UBC+UBA)	1					1	0.367
		cgd4_2360	DSK2 like protein with a ubiquitin domain, 2 STI1 motifs and a UBA domain at its C-terminus		1				1	2.339
		cgd4_3150	cullin-like protein of probable plant origin			1			1	2.419
		cgd4_40	APG7-like ubiquitin activating enzyme E1			1			1	5.432
		cgd4_570	ubiquitin-conjugating enzyme				1		1	10.077
		cgd5_2480	ubiquitin domain at the C-terminus	1					1	0.374
		cgd5_2500	ubiquitin C-terminal hydrolase of the cysteine proteinase fold		1				1	1.680
		cgd6_1850	anaphase promoting complex subunit 10, putative	1	1				2	1.001
		cgd6_2490	e3 ubiquitin-protein ligase, putative			1			1	3.439
		cgd6_4040	cullin domain containing protein	1					1	0.429
		cgd6_4180	ubiquitin-conjugating enzyme				1		1	10.291
		cgd6_4550	UBP_znfinger+UBA; UBP5 like deubiquitinating enzyme with a UB hydrolase domain and two UBA domains at the C-terminus	1					1	0.478
		cgd7_2800	ubiquitin-conjugating enzyme E2, putative		1				1	1.981
		cgd7_2840	ubiquitin conjugating enzyme, putative			1			1	4.735
		cgd7_4900	ubiqutin C-terminal hydrolase of the cysteine proteinase fold		1				1	1.361

		cgd7_4990	ubiquitin ligase with a HECT domain at the C-terminus	1					1	0.458
		cgd7_5020	ubiqutin family protein		1				1	1.696
		cgd8_1200	E3A like HECT domain containing ubiquitin protein ligase	1					1	0.356
		cgd8_1530	ubiquitin carboxyl terminal hydrolase domain that is fused to a MATH domain		1				1	1.628
		cgd8_160	ubiquitin activating enzyme E1	1					1	0.438
		cgd8_1730	Uba3p like ubiquitin activating enzyme E1	1					1	0.418
		cgd8_3850	Ubc6p like ubiquiting conjugating enzyme E2, possible transmembrane domain at C			1			1	3.408
		cgd8_4340	ubiquitin carboxyl-terminal hydrolase of the cysteine proteinase fold			1			1	4.183
		cgd8_580	ubiquitin-conjugating enzyme E2, putative			1			1	2.735
		cgd8_930	RBX1 ortholog, RING finger (Associated with E3 ubiquitin-protein ligase complex)	1					1	0.433
	Ubiquitin Total			15	11	9	3		38	2.294
**Protein degradation Total**				29	13	13	9	3	67	6.565
**Redox homeostasis**	Ferredoxin/FdxR	cgd6_3000	ferredoxin-like protein Fd1, putative	1					1	0.595
		cgd8_2710	NADPH:ferredoxin--NADP+ reductase with a rossman fold nucleotide binding domain and a 2Fe-2S ferredoxin domain	1					1	0.368
	Ferredoxin/FdxR Total			2					2	0.481
	Glutaredoxin	cgd2_2540	glutaredoxin related protein					1	1	250.416
		cgd3_460	glutathione peroxidase				1		1	7.518
		cgd6_3970	glutaredoxin-like protein; 2 thioredoxin folds		1				1	1.456
	Glutaredoxin Total				1		1	1	3	86.463
	SOD	cgd5_3230	superoxide dismutase	1					1	0.489
	SOD Total			1					1	0.489
	Thioredoxin	cgd2_1780	PDI like thioredoxin domain containing protein	1					1	0.383
		cgd2_4320	thioredoxin reductase 1					1	1	122.554
		cgd4_1650	possible thioredoxin H-type of possible fungal or plant origin, small protein		1				1	1.427
		cgd4_740	thioredoxin peroxidase-like protein		1				1	1.488
		cgd5_2030	thioredoxin, putative	1					1	0.405
		cgd5_2230	membrane associated thioredoxin	1					1	0.445
		cgd5_660	thioredoxin domain containing protein	1					1	0.486
		cgd6_2470	thioredoxin (quiescin Q6 like) having signal peptide and 12 transmembrane domains		1				1	2.249
		cgd6_850	thioredoxin; protein disulfide isomerase A6, signal peptide, possible transmembrane domain in C-terminal region	1					1	0.330
		cgd7_3170	thioredoxin fold protein related to phosducin	1					1	0.433
		cgd7_4840	phosducin related thioredoxin fold protein	1					1	0.369
		cgd7_5330	thioredoxin/PDI, cyanobacterial type, signal peptide plus 4 transmembrane domains		1				1	1.675
		cgd8_3510	thioredoxin-like protein, fragment	1					1	0.477
	Thioredoxin Total			8	4			1	13	10.209
**Redox homeostasis Total**				11	5		1	2	19	20.714
**Ribosome biogenesi**	Associated E/Pep	cgd1_3130	conserved eukaryotic nuclear protein that shares a domain with yeast Lcp5p, a component of the U3 small nucleolar ribonucleoprotein					1	1	42.968
		cgd2_2420	Yd1036cp-like RluD, S4+type 1 pseudouridine synthase			1			1	4.514
		cgd2_3680	eukaryotic ribosome biogenesis; Erb1p, putative					1	1	53.157
		cgd2_4090	YawG/Kre35p-like, Yjeq GTPase				1		1	18.447
		cgd2_50	SIK1 nucleolar protein Nop56					1	1	135.992
		cgd3_1340	NMD3p like protein involved in nonsense mediated decay					1	1	160.626
		cgd4_1480	BMS1 like GTpase involved in ribosome biogenesis					1	1	77.938
		cgd4_1520	Brx1p nucleolar protein required for biogenesis of the 60S ribosomal subunit					1	1	83.744
		cgd4_1580	Spb1p-like, FtsJ methylase					1	1	100.386
		cgd4_3320	Noc3 like protein involved in nuclear export of pre-ribosomes				1		1	13.214
		cgd4_4040	Yer006wp-like. Yjeq GTpase					1	1	39.810
		cgd4_660	Nip7 like PUA domain containing protein involved in ribosomal biogenesis					1	1	75.875
		cgd5_1190	nucleolar protein NOP4; rrm domain containing protein				1		1	11.459
		cgd5_1240	Tsr1p GTpase, multitransmembrane region protein					1	1	68.678
		cgd6_3230	Nop2p family of SUN/fmu RNA methylase				1		1	8.442
		cgd7_1620	nop15p/nopp34; nucleolar protein with 1 RRM domain					1	1	24.411
		cgd7_4110	WD40 protein (part of U3 processesome)					1	1	38.307
		cgd7_4130	Ynl022cp-like. SUN family methylase				1		1	12.053
		cgd8_1550	S. cerevisiae YLR387c-like protein with 2x C2H2 like zinc fingers conserved across eukaryotes plus an apicomplexan-specific globular domain			1			1	2.783
		cgd8_2000	KRE33p like superfamily I ATPase fused to an acetylase					1	1	54.173
		cgd8_3360	S. pombe SPAC1687.04 like conserved eukaryotic protein that shares a domain with several MCM7, probably involved in DNA replication			1			1	2.357
		cgd8_3380	Rrp12p like nucleolar protein					1	1	42.952
		cgd8_4530	ribosomal processing protein, putative				1		1	16.800
		cgd8_900	Ydr449cp/Utp6p; small (ribosomal) subunit (SSU) processosome (contains U3 snoRNA). HAT repeats					1	1	26.978
	Associated E/Pep Total					3	6	15	24	46.503
	Ribosomal protein	cgd1_1660	60S ribosomal protein L36					1	1	93.087

		cgd1_2270	40S ribosomal protein S26				1		1	19.394
		cgd1_300	40S ribosomal protein S21					1	1	91.571
		cgd1_3000	60S ribosomal protein L27					1	1	91.898
		cgd1_850	40S ribosomal protein S19					1	1	58.470
		cgd2_1070	40S ribosomal protein S25					1	1	90.960
		cgd2_120	ribosomal protein L29					1	1	70.698
		cgd2_130	60S acidic ribosomal protein LP2				1		1	14.045
		cgd2_170	40s ribosomal protein s24				1		1	13.318
		cgd2_2200	60S ribosomal protein L37					1	1	35.157
		cgd2_280	60S ribosomal protein L15					1	1	124.917
		cgd2_2870	40S ribosomal protein S28, no good start Met					1	1	25.847
		cgd2_2990	60S ribosomal protein L13, putative					1	1	33.295
		cgd2_3000	40S ribosomal protein S16				1		1	17.480
		cgd2_350	60S ribosomal protein L39					1	1	36.593
		cgd2_4260	40S ribosomal protein S6					1	1	63.051
		cgd3_1250	ribosomal protein L14					1	1	33.055
		cgd3_1300	60S ribosomal protein L12					1	1	64.008
		cgd3_2090	40S ribosomal protein SAe					1	1	41.474
		cgd3_2250	60S ribosomal protein L37A					1	1	303.987
		cgd3_2440	ribosomal protein S18A, rps18ap, HhH domain					1	1	33.305
		cgd3_3790	60S ribosomal protein L19				1		1	12.318
		cgd3_3890	60S ribosomal protein L30, pelota RNA binding domain containing protein					1	1	27.176
		cgd3_3930	60S ribosomal protein L27A or L27a					1	1	122.472
		cgd3_4100	conserved protein, COG SSU ribosomal protein S8E					1	1	40.342
		cgd3_830	60S ribosomal protein L35					1	1	72.728
		cgd4_1230	60S ribosomal protein L28					1	1	27.811
		cgd4_2260	ribosomal protein PO like protein of the L10 family					1	1	122.290
		cgd4_2400	60S ribosomal protein L31					1	1	121.935
		cgd4_3080	ribosomal protein S27a, ubiquitin plus zincribbon, UB13p					1	1	36.928
		cgd4_3160	putative 40S ribosomal protein S3A					1	1	46.278
		cgd4_4020	40S ribosomal protein S2/S5. DSRBD RNA binding domain					1	1	42.169
		cgd4_470	60S ribosomal protein L18					1	1	134.102
		cgd4_840	possible 60S ribosomal protein L24					1	1	45.215
		cgd5_2110	protein with conserved N-terminal localized cysteine-rich domain; predicted archaeo-eukaryotic ribosomal protein			1			1	3.085
		cgd5_2210	40S ribosomal protein S13					1	1	80.909
		cgd5_2370	60S acidic ribosomal protein LP1 like protein of possible plant origin					1	1	25.728
		cgd5_2630	60S ribosomal protein L7-B; Rp17bp; L30 RNA binding domain					1	1	33.575
		cgd5_2780	40S ribosomal protein S10				1		1	8.092
		cgd5_3040	40S ribosomal protein S7					1	1	99.993
		cgd5_3720	40S ribosomal protein S17					1	1	201.603
		cgd5_3740	40S ribosomal protein S12. pelota RNA binding domain containing protein					1	1	80.500
		cgd5_970	60S ribosomal protein L13A					1	1	86.027
		cgd6_1390	ribosomal protein S23					1	1	166.953
		cgd6_2170	60S ribosomal protein L11					1	1	216.518
		cgd6_2460	60s ribosomal protein L21					1	1	55.563
		cgd6_3180	40S ribosomal protein S15				1		1	7.340
		cgd6_3190	60S ribosomal protein-like, putative					1	1	85.370
		cgd6_3340	60S ribosomal protein L32					1	1	27.353
		cgd6_3710	40S ribosomal protein S30					1	1	28.469
		cgd6_4190	60S ribosomal protein L10, alpha/beta hammerhead					1	1	85.290
		cgd6_4320	40S ribosomal protein S5				1		1	15.540
		cgd6_4620	60S ribosomal protein L26					1	1	31.469
		cgd6_4630	40S ribosomal protein S8					1	1	146.828
		cgd6_570	60S ribosomal protein L23					1	1	137.766
		cgd7_1040	Imp3p-like 40S ribosomal protein S9. S4 RNA binding domain					1	1	50.011
		cgd7_130	40S ribosomal protein S14					1	1	79.747
		cgd7_1460	ribosomal protein S27					1	1	193.971
		cgd7_1600	ribosomal protein of the PO/L10 family					1	1	101.283
		cgd7_1880	60S ribosomal protein L44					1	1	90.826
		cgd7_2110	60S ribosomal proteins L8/L2					1	1	122.703
		cgd7_2250	40S ribosomal protein S3, KH domain					1	1	30.563
		cgd7_2280	60S ribosomal protein L40					1	1	49.851
		cgd7_2420	60S ribosomal protein L22				1		1	10.990
		cgd7_2540	60S ribosomal protein L35A					1	1	164.629
		cgd7_320	putative 60S ribosomal protein L18A					1	1	45.051
		cgd7_4050	ribosomal protein L38					1	1	127.806
		cgd7_4460	60S ribosomal protein L6					1	1	76.053
		cgd7_4760	ribosomal protein S4, putative					1	1	88.747
		cgd7_5060	putative 40S ribosomal protein S20				1		1	21.823
		cgd7_5310	60S ribosomal protein L24					1	1	206.286
		cgd8_1840	40S ribosomal subunit protein S9, putative					1	1	29.131
		cgd8_2870	60S ribosomal protein L10A					1	1	27.680
		cgd8_3450	60S ribosomal protein L17					1	1	33.615

		cgd8_3480	60S ribosomal protein L34					1	1	359.597
		cgd8_400	60S ribosomal protein L9					1	1	76.419
		cgd8_4050	ribosomal protein S29					1	1	36.522
		cgd8_430	60S ribosomal protein L7A					1	1	47.129
		cgd8_4350	60S ribosomal protein L7					1	1	64.096
		cgd8_4360	40S ribosomal protein S15A				1		1	13.826
		cgd8_440	60S ribosomal protein L5					1	1	91.543
		cgd8_650	60S ribosomal protein L23A					1	1	34.770
	Ribosomal protein Total					1	11	70	82	74.464
	rRNA processing	cgd1_1670	bystin/ S.cerevisiae En1p like adaptor domain					1	1	38.816
		cgd1_3160	mRNA 3' end processing protein RNA14, HAT repeats	1					1	0.404
		cgd2_160	IMP4-like U3 small nucleolar ribonucleoprotein (snoRNP)					1	1	42.116
		cgd3_1090	Rrp9p/U3-55K-family snoRNP-associated protein with several WD40 repeats				1		1	11.642
		cgd3_2950	ribosomal RNA assembly protein mis3/dribble/Krr1p. KH domain					1	1	53.167
		cgd3_3850	Cbf5p; centromere-binding factor 5 like PUA domain containing protein with a type I pseudouridine synthase domain					1	1	236.852
		cgd3_3970	Yer007c-ap/MCT-1 like PUA RNA binding domain containing protein			1			1	3.618
		cgd4_1700	GNog1p. GTpase					1	1	276.149
		cgd4_2740	IMP4 U3 small nucleolar ribonucleoprotein					1	1	88.930
		cgd4_2750	PWP1 family protein with WD40 repeats					1	1	239.214
		cgd4_3800	Rrp8p like methyltransferase involved in rRNA processing				1		1	8.222
		cgd5_3560	CNcl1p/MJ0026/YebU-like. SUN family methylase			1			1	5.874
		cgd7_2570	adrift-like. FTSJ family RNA methylase				1		1	6.987
		cgd7_3590	Mpp10 family of U3 processosome protein				1		1	6.951
		cgd8_2050	RRP5 like protein involved in rRNA biogenesis with 7 S1 domains and 5 HAT repeats					1	1	146.228
		cgd8_3390	S.cerevisiae Ssf2p/drosophila peter pan like protein that has an IMP4 domain at its N-terminus and is involved in rRNA processing					1	1	242.943
	rRNA processing Total			1		2	4	9	16	88.007
**Ribosome biogenesis Total**				1		6	21	94	122	70.739
**RNA metabolism**	RNA binding	cgd1_1280	domain KOG1676, K-homology type RNA binding proteins		1				1	1.439
		cgd1_1580	RING finger domain at N-terminus, CCCH domain at C-terminus, involved in RNA metabolism, HC-23 protein					1	1	256.528
		cgd1_250	ATP-dependent RNA helicase, putative			1			1	5.533
		cgd1_2760	yyaF/YCHF TRANSFAC/OBG family small GTpase plus RNA binding domain TGS				1		1	11.760
		cgd1_2960	protein kinase CMGC group, Sky1p like S/T protein kinase probably involved in RNA metabolism					1	1	55.879
		cgd1_830	sen1p/ NAM7 like superfamily I RNA helicase			1			1	2.655
		cgd3_1590	Spb4p, eIF4a-1-family RNA SFII helicase, DEXDc+HELICc domains					1	1	34.575
		cgd3_200	zfwd1 protein, CCCH like RNA binding domain fused to WD repeats					1	1	168.129
		cgd3_2580	RNA binding protein				1		1	9.912
		cgd3_2840	FmRP interacting protein like, contains 2 C2H2 zinc fingers involved in RNA metabolism				1		1	10.285
		cgd3_3920	DEAD/DEAH box ATP-dependent RNA helicase, putative					1	1	92.761
		cgd4_3000	Dbp1p, eIF4a-1 family RNA SFII helicase (DEXDC+HELICc)				1		1	18.046
		cgd4_3880	emap RNA binding domain protein (N terminal low complexity region)				1		1	6.720
		cgd4_4230	ATP-dependent helicase, putative					1	1	72.883
		cgd5_1070	CCCH RNA binding domain involved in RNA metabolism			1			1	2.353
		cgd5_1380	F11M21.28-like protein having 3 CCCH RNA binding domains; involved in RNA metabolism		1				1	1.705
		cgd5_1830	F11M21.28-like 3 CCCH RNA binding domain protein involved in RNA metabolism	1					1	0.546
		cgd5_2070	SPAC694.02. SKI family SFII helicase				1		1	12.737
		cgd5_2460	RNA binding RGG repeats plus RRM domain containing protein			1			1	4.249
		cgd5_2870	F11M21.28-like; 3 CCH RNA binding domain protein involved in RNA metabolism	1					1	0.346
		cgd5_3870	yprA. Lhr1/Ski2 family RNA SFII helicase	1					1	0.366
		cgd6_3010	poly(a)-binding protein fabm, putative					1	1	480.181
		cgd6_4910	F11M21.28-like protein with 3 CCCH RNA binding domains, involved in RNA metabolism					1	1	482.085
		cgd7_2560	Sgd1p; NIC plus MI domains containing protein involved in RNA metabolism		1				1	1.845
		cgd7_3510	helicase, putative		1				1	1.453
		cgd8_1820	ATP-dependent RNA helicase, putative			1			1	5.336
		cgd8_2520	Mtr4p like SKI family SFII helicase					1	1	32.047
		cgd8_3220	La domain fused to two RRMs (as in the La autoantigen) which is further fused to a C-terminal domain present in the plant DCL protein and the C-terminus of the alpha subunit of the plant RNA polymeras					1	1	73.442
	RNA binding Total			3	4	5	6	10	28	65.921
	RNA degradation	cgd2_2090	mitotic control protein dis3, putative			1			1	2.745
		cgd3_3270	possible nucleotide kinase related to CMP and AMP kinases				1		1	7.351
		cgd3_350	Pop2p-like 3'5' exonuclease, CCR4-NOT transcription complex				1		1	12.238

		cgd3_800	POP4 like ribonuclease P protein subunit			1			1	3.031
		cgd4_1950	RPR46-like RNAse PH domain			1			1	2.306
		cgd4_3930	archeo-eukaryotic exosomal RNAse				1		1	5.706
		cgd6_2080	Kem1p-like 5'-3' exonuclease		1				1	1.889
		cgd7_230	RPR45-like archaeo-eukaryotic exosomal RNAse PH			1			1	3.742
		cgd7_2380	RRP45-like archaeo-eukaryotic exosomal rnase, PH domain			1			1	3.607
		cgd8_2250	Rat1 Kar1/Rat1 like 5'-3' exonuclease				1		1	8.608
		cgd8_3310	S. cerevisiae Ylr022cp like protein that has a C2H2 zinc finger and is a component of the exosome		1				1	1.837
		cgd8_5150	ssd1p/F48E8.6-like RNAseII			1			1	2.723
	RNA degradation Total				2	6	4		12	4.649
	RNA modification	cgd1_1310	cleavage stimulation factor subunit 2 that has a CSF1 like RRM domain				1		1	8.330
		cgd1_1720	tRNA (Gm18) ribose methylase; trm3p; SpoU superfamily - SPOUT methylase	1					1	0.455
		cgd1_3440	Dus1p, tRNA dihydrouridine synthase		1				1	1.984
		cgd2_2710	TrmA RNA methylase			1			1	4.826
		cgd2_3570	YjbN-like Dus1p tRNA dihydouriding synthase Tim barrel				1		1	10.357
		cgd2_4300	Maf protein like, involved in RNA metabolism			1			1	5.300
		cgd2_570	tRNA/rRNA methyltransferase-like protein, putative			1			1	2.499
		cgd3_2040	Cca1p, tRNA adenylyltransferase	1					1	0.336
		cgd3_3500	Nop12p nucleolar protein, RRM domain			1			1	2.333
		cgd3_4330	Dbr1p-like RNA lariat debranching enzyme	1					1	0.374
		cgd3_450	Ctl1p-like mRNA capping enzyme beta subunit (polynucleotide 5'-triphosphatase)	1					1	0.507
		cgd4_2830	Mra1/NEP1 like protein, involved in pre-rRNA processing, adjacent genes putative paralogs					1	1	73.550
		cgd4_2840	Mra1/NEP1 like protein, involved in pre-rRNA processing, adjacent genes putative paralogs					1	1	40.794
		cgd4_3250	Trm1p. N2,N2-dimethylguanosine tRNA methyltransferase			1			1	5.492
		cgd4_3740	Ydr140wp-like HemK family methylase. archaeal-like. RNA methylase	1					1	0.283
		cgd4_930	pap1p; poly A polymerase (eukaryotic type)			1			1	2.808
		cgd5_1730	nclip/MJ0026/YebU-like; SUN family methylase				1		1	7.363
		cgd5_2390	GCD10p. RNA methylase				1		1	5.960
		cgd5_2470	GCD14 RNA methylase			1			1	3.800
		cgd5_2880	C1p1p GTpase. Pre-mRNA cleavage complex protein	1					1	0.443
		cgd5_4420	MJ0710-like (thump + methylase domains). RNA methylase			1			1	4.952
		cgd5_520	MJ1157-like thiouridine synthase (Pploop atpase) plus Zn ribbon. involved in RNA metabolism.	1					1	0.402
		cgd6_1230	GTpase Grc3p-like Pre-mRNA cleavage complex			1			1	3.775
		cgd6_1520	2-methylthioadenine synthetase; MiaB		1				1	1.416
		cgd6_2010	YbiN-like RNA methylase (NPPY variety; HemK family)	1					1	0.317
		cgd6_2380	mRNA capping enzyme; RNA guanylyltransferase Ceg1p		1				1	2.241
		cgd6_2540	tRNA delta(2)-isopentenylpyrophosphate transferase, putative			1			1	4.034
		cgd6_3560	MJ0041-family pseudouridylate synthase; Zn finger +thump + Psusyn				1		1	11.840
		cgd6_400	Pre-mRNA polyadenylation factor			1			1	3.801
		cgd6_410	Sgn1p-like RRM domain containing protein			1			1	2.895
		cgd6_5130	Trm5 RNA methyltransferase				1		1	11.827
		cgd6_930	Dim1p-like ERMB/KSGA methylase (dimethyladenosine transferase)				1		1	6.579
		cgd7_1530	ART-4 protein; PIN+Zn ribbon domains. involved in RNA metabolism				1		1	24.409
		cgd7_1630	Ncl1p/MJ0026/YebU-like. SUN family RNA methylase	1					1	0.380
		cgd7_2810	ortholog of kin17 (RNA metabolism proteins): a family of highly conserved zinc binding C-rich proteins with KOW domains	1					1	0.515
		cgd7_830	queunine tRNA-ribosyltransferase				1		1	7.701
		cgd8_1100	NRAP like nucleolar RNA associated protein		1				1	1.586
		cgd8_1120	HBS1 eRFS. GTpase. (RNA metabolism; translation)			1			1	4.375
		cgd8_1460	Sex-lethal interacting like				1		1	9.297
		cgd8_1580	saccharomyces Yor006cp like protein conserved across euks and archaea			1			1	3.079
		cgd8_1650	partner of Nob1; Pno1p; Yor145cp like KH domain containing protein					1	1	76.629
		cgd8_3660	RNA 3'-Terminal Phosphate Cyclase-like protein				1		1	8.624
		cgd8_4150	RRM+RGG RNA binding repeats			1			1	5.751
		cgd8_4330	fibrillarin. RNA methylase					1	1	106.146
		cgd8_460	cleavage and polyadenylation specifity factor protein, CPSF metallobeta-lactamase	1					1	0.404
		cgd8_510	Pus1p-like type II pseudousynthas TruA			1			1	3.111
		cgd8_5280	Deg1p-like type II pseudouridylate synthase TruA		1				1	2.186
	RNA modification Total			11	5	16	11	4	47	10.342
	RNA splicing	cgd1_2250	small nucealr riboprotein SMD3, SM domain		1				1	2.202
		cgd1_2730	Ro ribonucleoprotein-binding protein 1, RNA binding protein with 3x RRM domains					1	1	29.012
		cgd1_3080	splicing factor U2AF like SnRNP auxilary factor large subunit, RRM domain	1					1	0.335
		cgd1_930	U2 snrnp-specific A' protein		1				1	1.836
		cgd2_1480	splicing factor U2AF U2 SnRNP auxiliary factor large subunit, RRM domain		1				1	2.094

		cgd2_2770	U5 small nuclear ribonucleoprotein 200kDA helicase, Pre-mRNA splicing helicase BRR2 2 (RNA helicase plus Sec63 domain)	1					1	0.397
		cgd2_3510	Cus1p U2 snRNP protein		1				1	1.504
		cgd2_740	cut up CG6998-PA, putative			1			1	3.650
		cgd3_1060	U1 like C2H2 zinc finger	1					1	0.431
		cgd3_1230	SPAC14C4.06c-like PWI+4CCCH domains			1			1	4.723
		cgd3_1920	possible PRP38 pre-mRNA splicing factor family member	1					1	0.497
		cgd3_2110	nucleolar protein NOP5/NOP58-like pre-mRNA splicinig factor prp31					1	1	111.170
		cgd3_2880	U2 small nuclear ribonucleoprotein A' like LRR repeats	1					1	0.569
		cgd3_3690	U5 snRNP 100 kD protein, putative			1			1	2.353
		cgd3_3880	Snu114p GTpase, U5 snRNP-specific protein, 116 kDa				1		1	15.924
		cgd4_150	pre-mRNA splicing protein; Prp31p--like		1				1	2.225
		cgd4_270	30 kDa splicing factor, SPF 30, tudor domain containing protein				1		1	5.659
		cgd4_3190	small nuclear ribonucleoprotein			1			1	3.633
		cgd5_1330	SPAC25G10.01-like RRM domain containing protein					1	1	329.994
		cgd5_1740	conserved protein, possible U4/U6 associated RNA splicing factor	1					1	0.490
		cgd5_1760	small nucleolar RNP protein , Gar1 protein RNA binding region					1	1	31.331
		cgd5_2220	small nuclear ribonucleoprotein D1. SM domain containing protein		1				1	2.190
		cgd5_2960	U5snrp Brr2 SFII RNA helicase (sec63 and the second part of the RNA			1			1	5.387
		cgd5_3710	small nuclear ribonucleoprotein U6			1			1	2.410
		cgd5_4090	DHR1/Ecm16p/kurz. HrpA family SFII helicase			1			1	2.720
		cgd5_700	musashi. RRM domain containing protein, splicing related					1	1	52.477
		cgd5_920	Pre-mRNA splicing factor Pro1/Prp6. HAT repeat protein				1		1	5.868
		cgd6_1410	pre-mRNA splicing factor ATP-dependent RNA helicase, putative			1			1	5.044
		cgd6_1680	splicing factor U2AF U2 snRNP auxiliary factor large subunit; 3 RRM domains	1					1	0.420
		cgd6_2150	U1 snrnp, putative				1		1	13.022
		cgd6_2830	splicing factor 3a 66kD; N-terminus C2H2 domain	1					1	0.531
		cgd6_3850	HOI-POLLOI protein; U4/U6.U5 snRNP component; Snu13p; pelota RNA binding domain containing protein					1	1	494.940
		cgd6_4490	U2 snRNP. Hsh49p, RRM domain containing protein	1					1	0.389
		cgd6_4670	Prp9p-like splicing factor 3a subunit 3 snRNP. C-terminal C2H2			1			1	4.092
		cgd6_4710	5x RRM. Mrd1p like, splicing related					1	1	48.769
		cgd6_4750	splicing factor 3B subunit1-like HEAT repeat containing protein	1		1			2	1.965
		cgd6_990	U1 snrp Snp1p. RRM domain containing protein				1		1	6.729
		cgd7_1070	snRNP core protein		1				1	1.994
		cgd7_1750	step II splicing factor SLU7, putative		1				1	2.254
		cgd7_2040	small nuclear ribonucleo protein					1	1	40.264
		cgd7_2160	Pre-mRNA splicing factor SF3a. 2xSWAP domain protein		1				1	1.790
		cgd7_3610	RPR domain protein (involved in RNA metabolism)		1				1	1.394
		cgd7_3960	pleiotropic regulator 1 (spliceosome/RNA splicing)			1			1	3.349
		cgd7_4580	U6 snRNA-associated Sm-like protein LSm5. SM domain					1	1	27.760
		cgd7_5220	splicing factor with 3 RRM domains			1			1	2.318
		cgd7_5230	possible D-Tyr-tRNatyr deacylase		1				1	1.549
		cgd7_5490	PRP18 (SFM+PRP18 domains)				1		1	21.072
		cgd7_640	Prp16p pre-mRNA splicing factor. HrpA family SFII helicase				1		1	13.492
		cgd7_690	small nuclear ribonucleoprotein			1			1	4.467
		cgd7_940	splicing factor RRM domain containing protein; T22E16.120 SC35-like splicing factor					1	1	871.806
		cgd8_1480	RPR domain containing protein, present in proteins involved in mRNA splicing					1	1	33.306
		cgd8_190	Mak5 pre-mRNA splicing RNA SFII helicase				1		1	22.297
		cgd8_1950	shares a domain with a conserved HREF motif with the CWF15 protein that is involved in mRNA splicing		1				1	1.842
		cgd8_3340	PWI domain containing protein that is typically seen in spliceosomal proteins				1		1	20.209
		cgd8_3370	splicing factor SRP40 like 2x RRM domains		1				1	1.666
		cgd8_3690	mitosis protein DIM1			1			1	4.316
		cgd8_4100	PRP43 involved in spliceosome disassembly mRNA splicing			1			1	2.591
		cgd8_5240	U2AG splicing factor U2AF U2snRNP auxilliary factor small subunit CCCh+RRM+CCCh-like	1					1	0.444
	RNA splicing Total			11	13	15	9	11	59	38.629
	Rnase	cgd1_980	RNase L inhibitor-like protein				1		1	12.043
		cgd5_100	RRPp/PMC2 like exosome 3'-5' exoribonuclease subunit with an RNAseD domain and an HRDc domain				1		1	17.426
		cgd6_1040	RNAse P Rpr2/Rpp21 subunit domain				1		1	12.210
	Rnase Total						3		3	13.893
	tRNA synthesis	cgd8_4870	tRNA synthetase class II, putative					1	1	117.491
	tRNA synthesis Total							1	1	117.491
**RNA metabolism Total**				25	24	42	33	26	150	32.173
**Structure**	Cytoskeleton	cgd1_1540	dynein intermediate chain			1			1	3.460
		cgd1_2430	ARP3 actin related protein		1				1	1.667
		cgd1_2530	TRAFAC type P-loop GTpase that may be related to kinesin		1				1	1.835
		cgd1_2740	centromere/kinetochore protein		1				1	2.215

		cgd1_3460	F-actin capping protein, beta subunit	1					1	0.363
		cgd1_3570	myosin regulatory light chain				1		1	7.894
		cgd1_920	actin		1				1	1.558
		cgd2_1430	dynactin subunit p62, putative	1					1	0.387
		cgd2_4150	articulin family protein		1				1	1.771
		cgd3_140	b-tubulin specific chaparone	1					1	0.344
		cgd3_2120	myosin fused to 3 IQ motifs (that interact with calmodulin) and Rcc1 domain			1			1	2.251
		cgd3_2590	kinesin-related protein K2	1					1	0.302
		cgd3_3650	centrin like EF hand protein	1					1	0.520
		cgd4_2860	alpha tubulin			1			1	2.574
		cgd4_3290	articulin family protein, adjacent paralogous gene		1				1	1.613
		cgd4_3300	articulin family protein, adjacent paralogous gene			1			1	4.038
		cgd5_1320	actin-like protein, putative		1				1	1.832
		cgd5_2330	actin-like	1					1	0.394
		cgd5_2800	actin depolymerizing factor					1	1	122.394
		cgd5_3160	actin					1	1	475.922
		cgd5_4080	articulin family protein	1					1	0.470
		cgd5_60	centrin like protein with 4x EF hands	1					1	0.609
		cgd5_640	articulin family protein			1			1	4.728
		cgd6_1900	kinesin heavy chain, putative				1		1	13.879
		cgd6_1920	putative kinesin heavy chain	1					1	0.285
		cgd6_3370	microtubule-associated protein					1	1	35.773
		cgd6_3410	tubulin, gamma tubulin complex protein 3	1					1	0.315
		cgd6_4150	formin-related protein, putative		1				1	2.108
		cgd6_4210	kinesin-like boursin, putative				1		1	10.185
		cgd6_4340	myosin-related protein, putative		1				1	2.314
		cgd6_4560	myosin'myosin'				1		1	10.295
		cgd6_4760	tubulin beta chain	1					1	0.407
		cgd7_1660	STOP repeat protein involved in microtubule stabilization (possible variant metal binding site at N-terminus)		1				1	1.572
		cgd7_1980	gamma tubulin	1					1	0.404
		cgd7_2210	kinesin-like protein				1		1	12.644
		cgd7_2370	EB1 like microtubule binding protein	1					1	0.503
		cgd7_3060	gamma-tubulin complex associated protein		1				1	1.624
		cgd7_3330	Ced12 family of proteins that are conserved throughout eukaryotes		1				1	1.839
		cgd7_3790	unconventional myosin					1	1	293.367
		cgd7_4090	alpha superhelical protein, divergent TPR like repeats, possible microtubule associated protein		1				1	1.203
		cgd7_4350	myosin'unconventional myosin fused to an RCC1 domain (unique)'			1			1	2.722
		cgd8_1070	actin	1					1	0.434
		cgd8_1280	centrin, caltractin	1					1	0.545
		cgd8_1980	actin-related protein Arp23 like WD 40 repeate protein	1					1	0.299
		cgd8_2200	BRG1/brm-associated factor 53A like actin		1				1	1.359
		cgd8_2450	formin homology 2 domain (FH2) protein		1				1	1.720
		cgd8_3300	centromere associated Kip3p, kinesin like P-loop NTpase that belongs to the TRAFAC class GTpase superfamily				1		1	7.440
		cgd8_4120	caltractin (centrin)-like, contains 3x EFh domains	1					1	0.404
		cgd8_500	caltractin, centrin			1			1	3.538
		cgd8_5030	articulin family protein, Pfs77 protein-related			1			1	2.483
		cgd8_710	myosin heavy chain	1					1	0.351
	Cytoskeleton Total			18	15	8	6	4	51	20.572
	Oocyst wall	cgd4_3090	oocyst wall protein 6			1			1	5.214
		cgd6_200	oocyst wall protein 8				1		1	19.890
		cgd6_2090	CpCOWP1, oocyst wall protein with type I and type II cysteine-rich repeats				1		1	19.920
		cgd7_1800	oocyst wall protein 2	1					1	0.390
		cgd8_3350	oocyst wall protein 4		1				1	1.637
	Oocyst wall Total			1	1	1	2		5	9.410
**Structure Total**				19	16	9	8	4	56	19.575
**Transporter**	ABC	cgd1_1350	ATM1-like ABC transporter with 6 transmembrane domains	1					1	0.410
		cgd1_700	ATP-binding cassette protein				1		1	12.399
		cgd2_3870	putative ABC transporter with 2x AAA and 11+ transmembrane domains	1					1	0.357
		cgd2_70	ABC transporter, with 12 x transmembrane domains and 2x AAA domains					1	1	39.530
		cgd2_80	ABC transporter family protein, 2x AAA domain			1			1	2.792
		cgd2_90	ABC transporter with 9x transmembrande domains and 2xAAA					1	1	38.365
		cgd3_410	putative ABC transporter, 12x transmembrane domains plus 2x AAA					1	1	89.437
		cgd3_690	ABC transporter	1					1	0.561
		cgd4_1370	possible ABC transporter with 11 transmembrane domains, AAA domain					1	1	32.291
		cgd4_1380	possible ABC transporter with two AAA domains and 14 transmembrane regions		1				1	1.577
		cgd6_5260	ABC transporter ATpase with 2 AAA domains	1					1	0.398
		cgd6_5350	putative ABC transporter protein	1					1	0.391

		cgd6_5450	ABC transporter, AAA domain		1				1	1.474
		cgd7_2730	ABC transporter with ATpase domain plus 3 transmembrane regions		1				1	1.561
		cgd7_4510	ATP-binding cassette, sub-family C (CFTR/MRP), member 2; Canalicular multispecific organic anion transporter; multidrug resist					1	1	215.298
		cgd7_4520	ABC ATpase (2 ABC domains) with 10 transmembrane domains, adjacent duplicated gene					1	1	59.214
		cgd7_4800	ABC transporter, amino acid transporter 12 transmembrane spanning subunit				1		1	5.654
		cgd8_3250	ABC1 like protein kinase			1			1	3.306
	ABC Total			5	3	2	2	6	18	28.056
	Ion	cgd1_2190	K+/H+ antiporter of possible bacterial origin, signal peptide	1					1	0.314
		cgd1_2620	calcium antiporter, Na/Ca exchange associated membrane protein with 11 transmembrane domains					1	1	48.636
		cgd1_3050	cation diffusion facilitator like membrane protein transporter, 6x transmembrane domains	1					1	0.441
		cgd2_2680	possible MgtA, cation transport ATpase, signal peptide, 12 transmembrane domains			1			1	3.968
		cgd3_2730	protein with 10 transmembrane domains, possible calcium transporting ATpase or aminophospholipid transporter	1					1	0.343
		cgd3_3520	putative sodium/calcium cation exchanger having 12 transmembrane domains and signal peptide				1		1	25.496
		cgd4_1470	vacuolar proton translocating ATpase with 7 transmembrane regions near C-terminus					1	1	33.580
		cgd6_130	possible Na+/H+ and K+/H+ antiporter with 12 transmembrane domains, duplicated adjacent gene			1			1	4.089
		cgd6_140	possible Na+/H+ and K+/H+ antiporter with 12 transmembrane domains, duplicated adjacent gene					1	1	99.176
		cgd7_4070	arsenical pump-driving ATPase	1					1	0.378
		cgd7_4190	cation-transporting P-type ATpase with 11 or more transmembrane domains				1		1	9.950
		cgd7_90	high affinity sulfate transporter-related			1			1	2.295
		cgd8_2090	cation-transporting ATpase 2 with 8 transmembrane domains				1		1	15.144
	Ion Total			4		3	3	3	13	18.755
	MFS	cgd2_1450	major facilitator superfamily protein, putative, 12 transmembrane domain	1					1	0.294
		cgd7_3450	major facilitator (MFS) superfamily transporter containing 12 transmembrane domains				1		1	7.739
	MFS Total			1			1		2	4.016
	Nuclear transport	cgd3_2360	putative nucleoporin, FG-rich motifs within N-terminal region		1				1	1.722
		cgd3_300	rasputin. nuclear transport factor 2 (NTF2) domain plus RRM domain	1					1	0.399
		cgd3_3060	putative exportin 1			1			1	2.438
		cgd5_3950	Ran-binding protein		1				1	2.101
		cgd6_4610	mRNA export protein, putative				1		1	7.614
		cgd7_1670	nuclear pore protein RBP16/17 (RAN binding protein 16/17)				1		1	8.334
		cgd7_1780	tRNA exportin type nuclear export protein					1	1	25.649
		cgd7_1970	nuclear pore associated protein (NLP4) with N-terminal ubiquitin domain	1					1	0.559
		cgd7_220	GTP-binding nuclear protein ran/tc4					1	1	59.764
		cgd7_3030	importin/karyopherin	1					1	0.527
		cgd7_3270	importin/karyopherin (Arm repeats)					1	1	32.072
		cgd8_2100	RAN binding domain				1		1	21.037
		cgd8_2210	RCC1 like beta propeller protein [The regulator of chromosome condensation (RCC1), interact with Ran GTPase]	1					1	0.353
		cgd8_3260	importin alpha subunit		1				1	1.551
		cgd8_3440	importin beta like ARM repeat alpha superhelix			1			1	2.939
	Nuclear transport Total			4	3	2	3	3	15	11.137
	Nutrient	cgd1_40	UDP-galactose transporter, predicted signal peptide and 9 transmembrane domains	1					1	0.448
		cgd1_600	oxoglutarate/malate translocator protein				1		1	10.621
		cgd2_2400	ATPase, class II, type 9B, putative				1		1	18.081
		cgd2_2660	UDP N-acetylglucosamine transporter-like nucleotide sugar transporter with 10 transmembrane domains					1	1	153.274
		cgd2_2670	UDP N-acetylglucosamine transporter-like nucleotide sugar transporter with 10 transmembrane domains					1	1	53.888
		cgd2_310	nucleoside transporter, 10 transmembrane domain domain, nucleoside binding domain					1	1	69.085
		cgd2_590	nucleotide-sugar transporter, UDP N-acetylglucosamine-like, signal peptide, 9 or more transmembrane domains	1					1	0.363
		cgd3_1610	protein containing a possible START(Steroidogenic acute regulatory (STAR) related lipid transfer) domain				1		1	10.286
		cgd3_3100	12 transmembrane domain protein MFS family sugar transporter			1			1	5.019
		cgd3_4070	putative sugar transporter with 12 transmembrane domains					1	1	34.775
		cgd3_490	putative fucose translocator with 8 transmembrane domains, within locus of 3 paralogous genes		1				1	2.022
		cgd3_500	putative fucose translocator with 8 transmembrane domains, within locus of 3 paralogous genes					1	1	32.577
		cgd3_510	putative fucose translocatorw ith 8 transmembrane domains, within locus of 3 paralogous genes					1	1	398.701
		cgd4_2770	11 transmembrane domain protein, possible aa transporter					1	1	51.906

		cgd4_2870	putative sugar transporter, 12x transmembrane domain protein				1		1	14.544
		cgd4_80	Sac1p family protein				1		1	11.114
		cgd5_1140	cinnamyl-alcohol dehydrogenase-like nucleoside diphosphate sugar epimerase	1					1	0.521
		cgd5_3410	possible phosphate/phosphoenolpyruvate translocator with 9 transmembrane domains	1					1	0.324
		cgd6_2020	BT1 family protein (biopterin transporter 1)	1					1	0.611
		cgd8_1370	DP-fucose transporter with 9 transmembrane domains	1					1	0.358
		cgd8_1440	nucleotide sugar transporter like integral membrane protein with 9 transmembrane domains		1				1	1.327
		cgd8_1600	sugar phosphate permease with 11 transmembrane domains			1			1	3.082
		cgd8_3640	niemann-Pick type C1 disease protein/ patched like cholesterol transporter of the SecD family, 12 transmembrane domain		1				1	1.870
		cgd8_3740	Yer119cp like amino acid transporter, 11 transmembrane domain					1	1	87.487
		cgd8_80	10 transmembrane domain, possible aa transporter					1	1	27.209
	Nutrient Total			6	3	2	5	9	25	39.580
	P-type ATPase	cgd3_1110	P-type ATpase fused to two adenyl cyclase domains and 21 predicted transmembrane regions	1					1	0.286
		cgd3_4140	P-type ATpase3, 13 transmembrane regions				1		1	17.025
		cgd4_2720	P-type ATpase involved in cation transport			1			1	3.238
		cgd7_1760	P-type ATpase (calcium/phospholipid-transporter), 9 transmembrane domains		1				1	1.619
	P-type ATPase Total			1	1	1	1		4	5.542
	Undefined	cgd4_3110	10 transmembrane domain protein, possible translocator	1					1	0.571
		cgd4_4050	putative transporter with signal peptide and 12 transmembrane domains		1				1	2.012
		cgd5_1570	YIP1 protein like transporter GTpase interacting factor having 5 transmembrane domain		1				1	1.932
		cgd5_3580	putative transport protein				1		1	9.507
		cgd5_3590	putative transport protein			1			1	3.503
		cgd6_4890	transporter protein with conserved Zn ribbon C11C7CxxC				1		1	5.637
		cgd6_490	zinc ZIP transporter protein, putative				1		1	5.787
		cgd7_3290	cleft lip and palate family of eukaryotic membrane proteins (potential transporters) with 9 transmembrane domains			1			1	2.945
		cgd7_4290	membrane associated transporter, 10 transmembrane domain					1	1	53.911
	Undefined Total			1	2	2	3	1	9	9.534
	V-ATPase	cgd1_520	vacuolar ATP synthetase subunit					1	1	88.252
		cgd1_540	vacuolar ATP synthase subunit, possible signal peptide					1	1	47.341
		cgd2_3960	vacuolar ATP synthase subunit 54kD				1		1	9.850
		cgd4_540	putative vacuolar ATP synthase subunit C				1		1	9.712
		cgd5_3340	putative vacuolar ATP synthase subunit d					1	1	60.151
		cgd5_530	vacuolar H-ATpase subunit D					1	1	33.137
		cgd5_850	vacuolar ATP synthase subunit F					1	1	67.741
		cgd7_5000	vacuolar ATP synthase subunit A				1		1	12.560
		cgd8_1670	vacuolar ATP synthase subunit B			1			1	3.837
		cgd8_360	putative vacuolar ATP synthase subunit E					1	1	61.054
		cgd8_4790	proteolipid subunit of the vacuolar ATpase				1		1	11.382
	V-ATPase Total					1	4	6	11	36.820
**Transporter Total**				22	12	13	22	28	97	25.014
**Unknown**	Conserved	cgd1_1050	conserved hypothetical protein			1			1	3.063
		cgd1_1130	conserved hypothetical protein	1					1	0.313
		cgd1_1210	conserved hypothetical protein	1					1	0.526
		cgd1_140	conserved hypothetical protein			1			1	5.432
		cgd1_1550	conserved hypothetical protein	1					1	0.443
		cgd1_160	conserved hypothetical protein	1					1	0.335
		cgd1_2220	apicomplexan specific protein			1			1	4.609
		cgd1_2720	hypothetical protein with predicted apicomplexan-conserved domain	1					1	0.436
		cgd1_2910	conserved hypothetical protein	1					1	0.334
		cgd1_3070	multidomain protein with a conserved eukaryotic domain also present in the human DRIM protein at N- terminus and an archaeal-bacterial domain at C-terminus					1	1	27.886
		cgd1_3340	conserved hypothetical protein				1		1	6.873
		cgd1_360	conserved hypothetical protein				1		1	17.909
		cgd1_3630	conserved hypothetical protein				1		1	10.329
		cgd1_3700	conserved hypothetical protein		1				1	1.938
		cgd1_3750	conserved hypothetical protein	1					1	0.415
		cgd1_380	conserved hypothetical protein	1					1	0.391
		cgd1_3810	conserved hypothetical protein					1	1	501.576
		cgd1_720	apicomplexan-conserved protein			1			1	2.411
		cgd1_770	hypothetical protein, possible apicomplexan-conserved	1					1	0.318
		cgd1_840	conserved hypothetical protein				1		1	28.197
		cgd2_1010	conserved hypothetical protein			1			1	2.547
		cgd2_1210	conserved hypothetical protein			1			1	3.756
		cgd2_1220	conserved hypothetical protein				1		1	13.063
		cgd2_140	conserved hypothetical protein					1	1	836.204
		cgd2_190	conserved hypothetical protein			1			1	4.060
		cgd2_200	possible apicomplexan-specific protein					1	1	1,742.091

		cgd2_2000	apicomplexan protein with signal peptide and 3 transmembrane domains		1				1	1.584
		cgd2_2230	conserved hypothetical protein					1	1	25.464
		cgd2_2320	conserved hypothetical protein	1					1	0.369
		cgd2_250	conserved hypothetical protein					1	1	52.866
		cgd2_2510	conserved hypothetical protein	1					1	0.509
		cgd2_2610	conserved hypothetical protein	1					1	0.460
		cgd2_2790	conserved hypothetical protein		1				1	1.653
		cgd2_2810	conserved hypothetical protein	1					1	0.444
		cgd2_2920	conserved hypothetical protein	1					1	0.463
		cgd2_2940	conserved hypothetical protein					1	1	85.144
		cgd2_320	conserved hypothetical protein			1			1	5.125
		cgd2_3380	conserved hypothetical protein		1				1	1.488
		cgd2_3520	conserved hypothetical protein				1		1	14.974
		cgd2_3600	conserved hypothetical protein	1					1	0.397
		cgd2_3650	conserved hypothetical protein				1		1	12.884
		cgd2_3730	conserved hypothetical protein					1	1	648.512
		cgd2_3760	conserved hypothetical protein			1			1	2.971
		cgd2_40	conserved hypothetical protein	1					1	0.519
		cgd2_4170	conserved hypothetical protein		1				1	1.901
		cgd3_10	conserved hypothetical protein				1		1	6.573
		cgd3_1140	conserved hypothetical protein		1				1	1.859
		cgd3_1150	conserved hypothetical protein		1				1	2.080
		cgd3_1160	conserved hypothetical protein				1		1	15.224
		cgd3_1170	conserved hypothetical protein		1				1	2.263
		cgd3_1420	conserved small protein					1	1	30.229
		cgd3_1560	apicomplexan specific region near N-terminus			1			1	5.696
		cgd3_1620	conserved hypothetical protein		1				1	2.147
		cgd3_1700	conserved hypothetical protein	1					1	0.472
		cgd3_1710	conserved hypothetical protein		1				1	2.241
		cgd3_1720	conserved hypothetical protein	1					1	0.396
		cgd3_1730	conserved hypothetical protein		1				1	1.294
		cgd3_1750	conserved hypothetical protein				1		1	16.963
		cgd3_1760	conserved hypothetical protein				1		1	10.677
		cgd3_1770	conserved hypothetical protein			1			1	2.984
		cgd3_1780	conserved hypothetical protein	1					1	0.454
		cgd3_1790	conserved hypothetical protein	1					1	0.518
		cgd3_1940	conserved hypothetical protein					1	1	354.170
		cgd3_2240	conserved hypothetical protein	1					1	0.464
		cgd3_230	conserved hypothetical protein	1					1	0.307
		cgd3_2520	conserved hypothetical protein	1					1	0.444
		cgd3_2570	conserved protein	1					1	0.426
		cgd3_2820	conserved hypothetical protein	1					1	0.538
		cgd3_2970	conserved hypothetical protein	1					1	0.308
		cgd3_2980	conserved COOH terminal-specific cysteine rich domain	1					1	0.462
		cgd3_3090	conserved hypothetical protein	1					1	0.431
		cgd3_3360	conserved hypothetical protein				1		1	5.916
		cgd3_3390	conserved hypothetical protein	1					1	0.408
		cgd3_3660	conserved hypothetical protein				1		1	8.462
		cgd3_3810	conserved hypothetical protein	1					1	0.309
		cgd3_3870	conserved hypothetical protein					1	1	94.766
		cgd3_4010	conserved protein				1		1	7.066
		cgd3_4160	conserved hypothetical protein					1	1	198.349
		cgd3_4320	conserved hypothetical protein	1					1	0.550
		cgd3_4360	conserved hypothetical protein	1					1	0.450
		cgd3_50	conserved hypothetical protein		1				1	1.289
		cgd3_670	conserved hypothetical protein					1	1	35.845
		cgd3_730	conserved expressed protein			1			1	3.959
		cgd3_950	hypothetical conserved protein	1					1	0.408
		cgd4_1080	conserved hypothetical protein			1			1	3.738
		cgd4_1090	conserved hypothetical protein				1		1	6.043
		cgd4_1150	hypothetical conserved protein with possible signal peptide	1					1	0.360
		cgd4_1190	hypothetical protein, possible apicomplexan conserved	1					1	0.398
		cgd4_1220	conserved hypothetical protein					1	1	49.713
		cgd4_1400	conserved hypothetical protein					1	1	40.717
		cgd4_1440	apicomplexan specific protein		1				1	1.278
		cgd4_1510	conserved hypothetical protein			1			1	2.308
		cgd4_1530	conserved protein				1		1	21.579
		cgd4_1550	conserved hypothetical protein			1			1	3.800
		cgd4_1570	hypothetical protein, possible conserved				1		1	8.908
		cgd4_1680	conserved hypothetical protein	1					1	0.396
		cgd4_1910	conserved hypothetical protein		1				1	1.853
		cgd4_2010	conserved hypothetical protein			1			1	5.265
		cgd4_2020	conserved hypothetical protein				1		1	5.904
		cgd4_2060	conserved hypothetical protein	1					1	0.385
		cgd4_2210	conserved hypothetical protein	1					1	0.429

		cgd4_2390	conserved hypothetical protein	1					1	0.622
		cgd4_2850	hypothetical protein, possible apicomplexan conserved			1			1	4.047
		cgd4_3100	conserved hypothetical protein	1					1	0.441
		cgd4_3570	conserved hypothetical protein			1			1	3.333
		cgd4_3580	conserved hypothetical protein		1				1	2.245
		cgd4_3590	conserved hypothetical protein				1		1	10.942
		cgd4_3630	conserved hypothetical protein					1	1	32.062
		cgd4_3650	conserved hypothetical protein					1	1	38.743
		cgd4_3770	conserved hypothetical protein				1		1	8.784
		cgd4_4110	hypothetical conserved protein, possible transmembrane domain near C-terminus					1	1	44.812
		cgd4_4140	conserved hypothetical protein				1		1	21.495
		cgd4_4280	conserved hypothetical protein	1					1	0.324
		cgd4_4490	conserved hypothetical protein	1					1	0.377
		cgd4_70	conserved hypothetical protein				1		1	11.116
		cgd4_700	conserved protein of possible plant or bacterial origin		1				1	1.442
		cgd4_790	small conserved protein	1					1	0.360
		cgd5_1020	apicomplexan conserved protein with 9 transmembrane domain	1					1	0.427
		cgd5_1030	apicomplexan conserved protein			1			1	5.334
		cgd5_1270	conserved protein having 3 transmembrane domains and possible ER retention motif; possible ER protein	1					1	0.469
		cgd5_1310	conserved protein with possible signal peptide and ER retention motif; possible ER protein			1			1	2.461
		cgd5_1340	conserved hypothetical protein				1		1	11.264
		cgd5_1410	conserved protein	1					1	0.339
		cgd5_1540	conserved hypothetical protein		1				1	1.551
		cgd5_1790	conserved eukaryotic protein		1				1	1.817
		cgd5_1860	conserved hypothetical protein				1		1	22.521
		cgd5_1880	conserved probable chaperonin containing TCP-1 delta subunit		1				1	1.432
		cgd5_2380	unchacterized apicomplexan-specific serine rich low complexity protein		1				1	1.593
		cgd5_2520	conserved hypothetical protein	1					1	0.343
		cgd5_270	Unc45-like TPR repeat containing protein	1					1	0.395
		cgd5_2740	conserved hypothetical protein		1				1	2.064
		cgd5_3660	hypothetical conserved protein			1			1	3.297
		cgd5_3890	possible apicomplexan specific, low complexity protein	1					1	0.430
		cgd5_3940	hypothetical protein, possible apicomplexan-specific				1		1	8.912
		cgd5_4060	conserved hypothetical protein	1					1	0.534
		cgd5_4370	conserved hypothetical protein	1					1	0.497
		cgd5_4530	conserved hypothetical protein				1		1	5.858
		cgd5_4580	conserved hypothetical protein		1				1	1.493
		cgd5_4600	conserved hypothetical protein			1			1	2.389
		cgd5_490	conserved hypothetical protein	1					1	0.343
		cgd5_500	conserved protein		1				1	1.732
		cgd5_620	conserved hypothetical protein			1			1	3.964
		cgd5_650	conserved hypothetical protein		1				1	1.747
		cgd5_790	conserve protein having a signal peptide	1					1	0.378
		cgd6_1070	conserved hypothetical protein	1					1	0.359
		cgd6_1080	conserved hypothetical protein				1		1	23.828
		cgd6_1300	conserved hypothetical protein	1					1	0.464
		cgd6_1310	hypothetical protein, possible conservation in apicomplexa	1					1	0.438
		cgd6_1480	conserved hypothetical protein		1				1	1.974
		cgd6_1550	uncharacterized conserved protein	1					1	0.416
		cgd6_1640	conserved hypothetical protein	1					1	0.349
		cgd6_1650	conserved hypothetical protein	1					1	0.370
		cgd6_1660	conserved hypothetical protein		1				1	1.965
		cgd6_1820	conserved hypothetical protein					1	1	47.127
		cgd6_1910	conserved hypothetical protein			1			1	3.510
		cgd6_230	conserved hypothetical protein	1					1	0.370
		cgd6_2420	conserved hypothetical protein				1		1	11.272
		cgd6_2550	conserved hypothetical protein	1					1	0.677
		cgd6_30	conserved hypothetical protein				1		1	9.953
		cgd6_3260	conserved hypothetical protein	1					1	0.350
		cgd6_40	conserved hypothetical protein					1	1	878.258
		cgd6_4080	conserved hypothetical protein		1				1	1.840
		cgd6_4130	conserved hypothetical protein				1		1	8.262
		cgd6_4660	possible apicomplexan-specific protein					1	1	106.625
		cgd6_5320	conserved hypothetical protein	1					1	0.457
		cgd6_5330	conserved hypothetical protein	1					1	0.350
		cgd6_5340	conserved hypothetical protein	1					1	0.422
		cgd6_5380	conserved hypothetical protein				1		1	10.469
		cgd6_5460	conserved hypothetical protein	1					1	0.633
		cgd6_5480	conserved hypothetical protein	1					1	0.448
		cgd6_5490	conserved hypothetical protein	1					1	0.410
		cgd6_680	conserved protein with possible C2 domain	1			1		2	3.188
		cgd6_70	conserved hypothetical protein			1			1	3.364
		cgd6_830	conserved hypothetical protein		1				1	1.810

		cgd6_870	possible apicomplexan conserved protein	1					1	0.438
		cgd7_1060	apicomplexan conserved protein with 2 or more transmembrane domains			1			1	2.333
		cgd7_1150	uncharacterized conserved protein	1					1	0.262
		cgd7_1160	conserved hypothetical protein		1				1	1.409
		cgd7_1180	conserved hypothetical protein			1			1	3.055
		cgd7_1340	conserved hypothetical protein		1				1	1.616
		cgd7_1380	conserved hypothetical protein			1			1	2.382
		cgd7_1390	conserved hypothetical protein			1			1	2.436
		cgd7_1500	conserved protein	1					1	0.405
		cgd7_250	conserved protein				1		1	20.096
		cgd7_2510	conserved hypothetical protein				1		1	9.015
		cgd7_2520	conserved hypothetical protein				1		1	21.146
		cgd7_2690	conserved hypothetical protein			1			1	2.914
		cgd7_270	conserved hypothetical protein	1		1			2	2.618
		cgd7_310	conserved hypothetical protein		1				1	2.181
		cgd7_3100	conserved hypothetical protein				1		1	16.123
		cgd7_3160	conserved hypothetical protein			1			1	3.784
		cgd7_3220	conserved hypothetical protein			1			1	2.454
		cgd7_3300	conserved hypothetical protein	1					1	0.503
		cgd7_340	conserved hypothetical protein	1					1	0.588
		cgd7_3490	conserved hypothetical protein					1	1	65.965
		cgd7_350	conserved hypothetical protein	1					1	0.430
		cgd7_3540	conserved hypothetical protein		1				1	2.220
		cgd7_3550	conserved hypothetical protein				1		1	17.376
		cgd7_3740	apicomplexan P36 family of proteins, appears to be a divergent HAD family phosphatase	1					1	0.390
		cgd7_3810	conserved hypothetical protein				1		1	6.003
		cgd7_3830	conserved hypothetical protein			1			1	3.059
		cgd7_3840	conserved hypothetical protein			1			1	4.068
		cgd7_3860	conserved hypothetical protein	1					1	0.332
		cgd7_3870	conserved hypothetical protein	1					1	0.311
		cgd7_4410	conserved protein with YSHH motif. SOme fused to polo box.				1		1	8.532
		cgd7_4710	conserved hypothetical protein					1	1	101.754
		cgd7_4780	conserved hypothetical protein	1					1	0.357
		cgd7_5080	conserved hypothetical protein	1					1	0.454
		cgd7_5120	conserved hypothetical protein	1					1	0.551
		cgd7_5400	conserved hypothetical protein	1					1	0.347
		cgd7_5450	conserved hypothetical protein	1					1	0.496
		cgd7_610	apicomplexan conserved protein		1				1	1.594
		cgd7_650	conserved hypothetical protein			1			1	3.889
		cgd8_1060	uncharacterized conserved protein				1		1	18.004
		cgd8_130	conserved hypothetical protein	1					1	0.510
		cgd8_1340	conserved hypothetical protein	1	1				2	0.976
		cgd8_2080	conserved hypothetical protein		1				1	1.584
		cgd8_2110	conserved hypothetical protein	1					1	0.441
		cgd8_230	conserved hypothetical protein			1			1	2.337
		cgd8_2320	hypothetical protein, potential AT hook	1					1	0.444
		cgd8_2490	conserved hypothetical protein					1	1	160.860
		cgd8_2540	apicomplexan specific coiled coil protein	1					1	0.445
		cgd8_2550	apicomplexan specific membrane protein, 4+ transmembrane domain	1					1	0.387
		cgd8_2640	conserved metal binding motif protein	1					1	0.337
		cgd8_2650	conserved hypothetical protein	1					1	0.406
		cgd8_270	conserved hypothetical protein	1					1	0.393
		cgd8_2780	conserved protein	1					1	0.561
		cgd8_280	apcomplexan conserved protein			1			1	3.966
		cgd8_2880	possible apicomplexan conserved motif		1				1	2.134
		cgd8_2890	conserved hypothetical protein		1				1	1.435
		cgd8_3150	hypothetical conserved protein				1		1	18.461
		cgd8_3230	conserved hypothetical protein					1	1	507.630
		cgd8_3240	apicomplexan specific protein				1		1	12.144
		cgd8_3330	conserved hypothetical protein				1		1	8.735
		cgd8_3630	conserved hypothetical protein				1		1	18.339
		cgd8_3730	conserved hypothetical protein		1				1	1.245
		cgd8_4030	apicomplexan specific coiled coil protein	1					1	0.465
		cgd8_4170	conserved hypothetical protein			1			1	3.792
		cgd8_4220	conserved hypothetical protein					1	1	320.236
		cgd8_4260	conserved hypothetical protein					1	1	35.637
		cgd8_4320	possible conserved domain (Zn finger?)					1	1	1,685.645
		cgd8_4370	conserved protein					1	1	73.348
		cgd8_4670	apicomplexan specific Pf 23612804 and Py 23478322			1			1	4.020
		cgd8_4680	conserved protein; Py hit 23481194					1	1	63.692
		cgd8_4850	conserved hypothetical protein	1	1				2	1.001
		cgd8_5000	hypothetical protein, possible conserved					1	1	99.274
		cgd8_5130	conserved hypothetical protein		1				1	1.656
		cgd8_5160	uncharacterized apicomplexan-specific protein				1		1	13.739

	cgd8_5350	conserved hypothetical protein				1		1	7.709
	cgd8_5360	conserved hypothetical protein					1	1	119.462
	cgd8_5380	conserved hypothetical protein				1		1	16.047
	cgd8_5390	conserved hypothetical protein				1		1	10.551
	cgd8_5400	conserved hypothetical protein		1				1	1.761
	cgd8_5420	conserved hypothetical protein					1	1	153.778
	cgd8_690	conserved hypothetical protein	1					1	0.311
	cgd8_70	conserved hypothetical protein				1		1	8.491
	cgd8_940	conserved hypothetical protein					1	1	205.198
Conserved Total			97	41	40	49	34	261	39.608
Membrane	cgd1_10	predicted membrane associated protein with 11 transmembrane domains regions		1				1	1.697
	cgd1_1470	hypothetical protein, possible transmembrane domain within N-terminus				1		1	6.311
	cgd1_1480	predicted membrane associated protein, 4 transmembrane domains and signal peptide			1			1	5.762
	cgd1_1820	predicted integral membrane protein, 4 transmembrane domains				1		1	15.664
	cgd1_20	predicted membrane associated protein with 11 transmembrane domains regions			1			1	3.252
	cgd1_2030	membrane associated protein with over 9 transmembrane domains, signal peptide			1			1	2.737
	cgd1_2080	hypothetical protein containing signal peptide and 3 transmembrane domain regions		1				1	1.910
	cgd1_2090	hypothetical protein, C2C2 like finger		1				1	2.170
	cgd1_2510	hypothetical protein, transmembrane domain within N-terminus	1					1	0.438
	cgd1_2780	hypothetical protein, 4 transmembrane domains				1		1	16.287
	cgd1_2880	13 kda membrane protein subunit		1				1	2.242
	cgd1_30	predicted membrane protein with 12+xtransmembrane domain	1					1	0.579
	cgd1_3200	hypothetical protein with 2 transmembrane domains, possible apicomplexan conservation	1					1	0.380
	cgd1_3250	signal peptide, possible transmembrane domain near N-terminus, proline rich and His-stretch at C-terminus				1		1	12.450
	cgd1_3590	hypothetical protein, possible transmembrane domain	1					1	0.501
	cgd1_3680	extracellular membrane associated protein with 3 EGF domains and a transmembrane domain	1					1	0.361
	cgd1_3820	hypothetical protein, transmembrane domain or GPI anchor near C-terminus					1	1	45.793
	cgd1_3850	signal peptide, possible 4-5x transmembrane domain, SKSR family paralog, telomeric gene					1	1	172.377
	cgd1_780	hypothetical protein, possible 2 transmembrane domains		1				1	1.471
	cgd1_950	hypothetical protein, membrane associated ankyrin repeats, 3X signal peptide plus transmembrane domain or GPI anchor		1				1	1.907
	cgd2_1290	hypothetical protein, signal peptide, transmembrane domain near C-terminus	1	1				2	0.909
	cgd2_1310	11 transmembrane domain protein				1		1	21.320
	cgd2_1590	extracellular protein with signal peptide, 5xEGF and apple domains		1				1	1.739
	cgd2_1710	hypothetical protein with signal peptide and one or more transmembrane domain regions	1					1	0.383
	cgd2_1990	hypothetical protein with possible transmembrane domain or GPI at C-terminus	1					1	0.315
	cgd2_2250	putative integral membrane protein			1			1	3.186
	cgd2_2390	hypothetical protein with 8 transmembrane domains				1		1	6.463
	cgd2_2490	hypothetical protein, possible transmembrane domain at C-terminus	1					1	0.388
	cgd2_2630	hypothetical protein, signal peptide plus 11 transmembrane domains		1				1	1.682
	cgd2_2650	hypothetical protein, signal peptide, transmembrane domain near C-terminus				1		1	5.900
	cgd2_2840	pleckstrin homology (PH) domain containing protein with N-terminal ankyrin repeats		1				1	1.359
	cgd2_3080	CpTSP10 protein		1				1	2.004
	cgd2_3100	hypothetical protein, 8 transmembrane domains			1			1	2.458
	cgd2_3120	possible transmembrane domain near N					1	1	24.537
	cgd2_3470	hypothetical protein with signal peptide and one or more transmembrane domains	1					1	0.230
	cgd2_3560	hypothetical protein with 8 transmembrane domains	1					1	0.290
	cgd2_3750	hypothetical protein, transmembrane domain near C-terminus		1				1	1.471
	cgd2_4060	hypothetical protein with 12 transmembrane domains	1					1	0.360
	cgd2_4350	putative patched family protein with 12 transmembrane domain		1				1	1.572
	cgd2_650	hypothetical protein with transmembrane domain within N-terminus	1					1	0.352
	cgd2_790	CpCCp3, multidomain extracellular protein with a signal peptide and the following architecture: LH2+LCCL+2xSR+LCCL+pentraxin+2xLCCL			1			1	4.267
	cgd2_800	hypothetical protein with 10 transmembrane domains	1					1	0.418
	cgd2_940	signal peptide plus transmembrane domain or GPI anchor					1	1	240.895
	cgd3_1010	hypothetical protein with 3 transmembrane domains within C-terminus	1					1	0.538
	cgd3_1370	hypothetical protein, transmembrane domain or GPI anchor at C-terminus	1					1	0.312

		cgd3_1520	hypothetical protein with transmembrane or GPI anchor sequence at carboxy terminus			1			1	2.618
		cgd3_1600	hypothetical protein with 4 transmembrane domains	1					1	0.413
		cgd3_1640	hypothetical protein with 7 transmembrane domains			1			1	4.519
		cgd3_1740	hypothetical protein with signal peptide and 2 transmembrane domains				1		1	9.113
		cgd3_1800	hypothetical protein with 12 transmembrane domains		1				1	1.303
		cgd3_1860	extracellular protein with a signal peptide and 9 EGF domains	1					1	0.421
		cgd3_1930	hypothetical protein with 4 transmembrane domains	1					1	0.391
		cgd3_2080	large protein with 8 or more transmembrane domains within NH2 region			1			1	4.105
		cgd3_2300	hypothetical protein with transmembrane domain within C-terminus				1		1	18.959
		cgd3_2500	hypothetical protein with transmembrane region near N-terminus	1					1	0.444
		cgd3_2780	hypothetical protein with transmembrane domain at carboxy terminus	1					1	0.468
		cgd3_2990	hypothetical protein with signal peptide and 7 transmembrane domains		1				1	2.094
		cgd3_340	hypothetical protein with signal peptide and 5 transmembrane domains				1		1	15.006
		cgd3_3420	protein with 2x EF hands and 4 transmembrane regions	1					1	0.386
		cgd3_3430	extracellular protein with a signal peptide sequence, MAM domain and a Cu amine oxidase domain		1				1	1.291
		cgd3_3940	predicted membrane associated protein					1	1	189.171
		cgd3_4030	hypothetical protein with 7 transmembrane domain domain					1	1	83.491
		cgd3_4300	possible t-snare domain, possible transmembrane domain or GPI anchor signal at C-terminus				1		1	8.767
		cgd3_470	hypothetical protein with 10 transmembrane domains					1	1	72.023
		cgd3_540	protein with 12 transmembrane domains					1	1	95.706
		cgd3_550	protein with 12 transmembrane domains					1	1	172.497
		cgd3_590	hypothetical protein with 4 transmembrane domains, possible unusual phyletic distribution		1				1	1.585
		cgd3_610	hypothetical protein with possible 4 transmembrane domains					1	1	166.828
		cgd3_740	hypothetical protein with 7 transmembrane domains			1			1	4.818
		cgd3_970	hypothetical protein, possible 2 transmembrane domains	1					1	0.367
		cgd4_1120	WD40 repeat containing protein that has a transmembrane region at the C-terminus	1					1	0.319
		cgd4_1160	conserved protein with transmembrane region at C-terminus					1	1	36.075
		cgd4_1310	hypothetical protein with transmembrane domain			1			1	2.641
		cgd4_160	hypothetical protein, possible transmembrane domain within N-terminus				1		1	9.120
		cgd4_1670	hypothetical protein with signal peptide and 3 to 6 transmembrane domains		1				1	1.856
		cgd4_1760	hypothetical protein with 2 transmembrane domains	1					1	0.382
		cgd4_1860	hypothetical protein with 10 transmembrane domains		1				1	1.436
		cgd4_1970	protein with central transmembrane domain followed by gly-met-pro repeat			1			1	4.711
		cgd4_2180	hypothetical protein with 12+ transmembrane domains	1					1	0.376
		cgd4_2420	extracelllular membrane associated protein with a signal peptide, 5 ankyrin repeats, SUSHI domain and a transmembrane region		1				1	1.438
		cgd4_2430	hypothetical protein containing a signal peptide and 2 transmembrane domains				1		1	11.650
		cgd4_2530	possible transmembrane domain near C					1	1	27.238
		cgd4_3340	pleckstrin homology (PH) domain containing protein					1	1	68.746
		cgd4_3470	hypothetical protein with signal peptide and transmembrane domain near C, within large locus of signal peptide containing proteins		1				1	1.483
		cgd4_3480	hypothetical protein with signal peptide plus possible GPI anchor or transmembrane domain near C-terminus, within large locus of signal peptide containing proteins					1	1	95.528
		cgd4_350	hypothetical protein with 7 transmembrane domains	1					1	0.440
		cgd4_3520	hypothetical protein with signal peptide and repeats, within large locus of signal peptide containing proteins	1					1	0.450
		cgd4_360	hypothetical protein with 5 transmembrane domains	1					1	0.457
		cgd4_380	protein with signal peptide plus 12 transmembrane domain domain protein, possible tranporter					1	1	55.249
		cgd4_3970	signal peptide plus transmembrane domain or GPI anchor, proline rich, acidic stretches			1			1	3.008
		cgd4_3990	hypothetical protein with possible transmembrane domain near N-terminus	1					1	0.354
		cgd4_420	signal peptide + 4 transmembrane domain protein					1	1	132.630
		cgd4_4210	hypothetical protein with signal peptide plus transmembrane domain		1				1	2.085
		cgd4_4470	putative secreted, membrane-associated protein		1				1	2.226
		cgd4_4480	putative secreted, membrane-associated protein	1					1	0.344
		cgd4_60	extracellular membrane associated protein with a signal peptide, an EGF domain followed by 10 transmembrane domains	1					1	0.370
		cgd4_640	extracellular protein with a signal peptide, FN3 domain and a predicted transmembrane region	1					1	0.526
		cgd4_750	extracellular protein with a signal peptide and 8 kazal repeats	1					1	0.438

		cgd4_800	Yip1p like integral membrane protein	1					1	0.367
		cgd4_980	hypothetical protein with 7 transmembrane domains, possible signal peptide		1				1	1.580
		cgd5_1250	hypothetical protein with transmembrane domain near N-terminus	1					1	0.432
		cgd5_130	ferlin like type II membrane associated protein with 5 C2 domains and a transmembrane domain or GPI anchor sequence at the C-terminus		1				1	1.531
		cgd5_1560	hypothetical protein, 2 transmembrane domains near C-terminus				1		1	11.530
		cgd5_1630	protein with signal peptide, and 11 transmembrane domains			1			1	4.479
		cgd5_1920	pleckstrin homology (PH) domain containing protein			1			1	2.352
		cgd5_1950	hypothetical protein with 5 transmembrane domains	1					1	0.400
		cgd5_2020	extracellular protein			1			1	2.756
		cgd5_2150	protein with signal peptide and 8 transmembrane domains; possible ZIP zinc transporter family member					1	1	87.906
		cgd5_2490	hypothetical protein with 5 transmembrane domains within C terminus	1					1	0.395
		cgd5_2530	protein with 12 transmembrane domains, possible apicomplexan-specific protein	1					1	0.413
		cgd5_2580	hypothetical protein with signal peptide, possible transmembrane domain near C-terminus and predicted SMART Znf_C2H2 domain			1			1	3.155
		cgd5_290	hypothetical protein, 6 transmembrane domains	1					1	0.302
		cgd5_2990	hypothetical protein with transmembrane domain within C-terminal region				1		1	20.209
		cgd5_3130	large hypothetical protein with 8 or more transmembrane domains	1					1	0.531
		cgd5_3330	hypothetical protein with 5 transmembrane domains			1			1	2.482
		cgd5_3500	hypothetical protein having 10 transmembrane domains					1	1	245.766
		cgd5_360	conserved protein with 2 transmembrane domain	1					1	0.331
		cgd5_3690	protein with 8 transmembrane domains			1			1	4.705
		cgd5_3970	protein with 3 transmembrane domains, ring domain, and possible signal peptide		1				1	2.199
		cgd5_3980	hypothetical protein with possible transmembrane domain near N-terminus		1				1	1.511
		cgd5_3990	protein with 4 transmembrane domains, signal peptide and a RING domain			1			1	3.176
		cgd5_4050	hypothetical protein with 4 transmembrane domains	1					1	0.399
		cgd5_4140	hypothetical protein with 4 transmembrane domains within C-terminus	1					1	0.417
		cgd5_4220	membrane associated protein with a signal peptide, EGF domain and 7 transmembrane domains at the C-terminus	1					1	0.502
		cgd5_4270	hypothetical protein with signal peptide and transmembrane region at C-terminus, possible N-terminal region transmembrane domain	1					1	0.355
		cgd5_4280	hypothetical protein with signal peptide and possible transmembrane region at C-terminus	1					1	0.494
		cgd5_4310	hypothetical protein with 7 transmembrane domains	1					1	0.367
		cgd5_4450	hypothetical protein with 8 transmembrane domains	1					1	0.366
		cgd5_4470	CpTSP7; extracellular membrane associated protein with a signal peptide followed by 2 TSP1 repeats, an EGF domain and a transmembrane region	1					1	0.332
		cgd5_570	hypothetical protein, 5 transmembrane domains near N-terminus	1					1	0.303
		cgd5_580	hypothetical protein with 5 transmembrane domains near N-terminus	1					1	0.463
		cgd5_590	hypothetical protein with possible transmembrane domain within C	1					1	0.479
		cgd6_110	conserved protein with signal peptide and transmembrane domain or GPI anchor signal near C-terminus	1					1	0.415
		cgd6_1320	hypothetical protein, possible transmembrane domain near N-terminus			1			1	4.625
		cgd6_1350	hypothetical protein with possible transmembrane domain within C-terminus	1					1	0.350
		cgd6_1490	dbj				1		1	8.143
		cgd6_1510	hypothetical protein with 6 transmembrane domains		1				1	1.398
		cgd6_2030	transmembrane protein with 6 transmembrane domains			1			1	3.869
		cgd6_2120	hypothetical protein, conserved cysteine residues, possible transmembrane domain or GPI anchor at C-terminus			1			1	2.494
		cgd6_2130	hypothetical protein with a C-terminus transmembrane domain		1				1	1.513
		cgd6_2210	membrane-associated protein with 6 transmembrane domains possible apicomplexan conserved, duplicated adjacent gene			1			1	4.047
		cgd6_2220	membrane-associated protein with 6 transmembrane domains possible apicomplexan conserved, duplicated adjacent gene			1			1	3.262
		cgd6_2280	hypothetical protein with signal peptide and 12 or more transmembrane domains				1		1	6.014
		cgd6_2340	hypothetical protein with signal peptide and 12 or more transmembrane domains				1		1	6.891
		cgd6_2440	hypothetical protein having 6 transmembrane domains					1	1	29.134
		cgd6_260	hypothetical protein with 4 transmembrane domains	1					1	0.331
		cgd6_2620	vesicle-associated membrane protein,MSP (major sperm protein) domain, transmembrane domain or GPI at C					1	1	63.320
		cgd6_2640	hypothetical protein with 12 transmembrane domains	1					1	0.314

		cgd6_2730	protein with signal peptide plus possible GPI signal or transmembrane domain at C-terminus				1		1	16.173
		cgd6_3020	hypothetical protein with 8 transmembrane domains			1			1	3.109
		cgd6_3350	hypothetical protein with transmembrane domain	1					1	0.306
		cgd6_3530	hypothetical protein with possible 4 or more transmembrane domains		1				1	1.459
		cgd6_3580	hypothetical protein with 3 or more transmembrane domains				1		1	9.550
		cgd6_3630	secreted protein with ankyrin repeats, signal peptide plus transmembrane domain or GPI anchor		1				1	1.767
		cgd6_3730	large extracellular protein with a signal peptide, apple domain and a transmembrane region			1			1	2.950
		cgd6_3870	hypothetical integral membrane protein with 12 transmembrane domains	1					1	0.365
		cgd6_3910	pleckstrin homology (PH) domain containing protein				1		1	24.578
		cgd6_4310	hypothetical low complexity	1					1	0.417
		cgd6_4440	hypothetical protein with transmembrane region near N-terminus	1					1	0.387
		cgd6_4680	hypothetical protein with 12 transmembrane domains	1					1	0.376
		cgd6_4730	hypothetical protein with 6 transmembrane domains		1				1	2.255
		cgd6_4740	hypothetical protein with 6 transmembrane domains and signal peptide					1	1	29.113
		cgd6_4920	hypothetical protein with 4 transmembrane domains		1				1	2.107
		cgd6_5140	ERGIC-53-like mannose binding lectin that is a type I membrane protein, transmembrane domain near C, signal peptide			1			1	5.570
		cgd6_5430	hypothetical protein, signal peptide plus transmembrane domain or GPI anchor					1	1	79.165
		cgd6_590	hypothetical protein with possible transmembrane domain or GPI anchor signal at C-terminus	1					1	0.367
		cgd6_60	hypothetical protein with signal peptide and proline stretch at C-terminus	1					1	0.279
		cgd6_670	extracellular protein with a signal peptide, clostripain like caspase/hemoglobinase domain, notch domain and 2 EGF domains	1					1	0.248
		cgd6_760	conserved protein with 6 transmembrane domain	1					1	0.413
		cgd6_780	CpTSP8,predicted extracellular protein with 3 TSP1 repeats, an EGF domain and a C-terminal transmembrane domain. adjacent TSP domain containing gene.					1	1	849.606
		cgd6_800	CpTSP9, extracellular protein with 3 TSP1 domains and an EGF domain	1					1	0.513
		cgd6_90	hypothetical protein with 5 transmembrane domains					1	1	68.318
		cgd7_1310	extracellular protein with a signal peptide followed by a family 2 glycosyltransferase and ricin domains	1					1	0.346
		cgd7_150	hypothetical protein with signal peptide and transmembrane domain near C-terminus	1					1	0.424
		cgd7_1650	protein with signal peptide and 6 transmembrane domains		1				1	1.391
		cgd7_1730	CpCCp1/Cpa135				1		1	6.106
		cgd7_1860	P24 like gold domain protein, possible transmembrane region near C-terminus, signal peptide		1				1	1.688
		cgd7_1960	membrane protein with multiple cysteines at the N-terminus.			1			1	4.872
		cgd7_2150	transmembrane protein, putative	1					1	0.418
		cgd7_2170	hypothetical membrane protein				1		1	5.912
		cgd7_2590	integral membrane protein with 10 transmembrane domains		1				1	1.951
		cgd7_300	CpCCP2; extracellular protein with a signal peptide, ricin, discoidin, NEC, LCCL, 2 levanase and an apicomplexan-specific cysteine-rich repeat (apicA repeat)		1				1	1.654
		cgd7_3040	membrane protein conserved in eukaryotes		1				1	1.729
		cgd7_3070	hypothetical protein, signal peptide, transmembrane domain near C-terminus				1		1	19.438
		cgd7_3250	membrane protein with no close homologs,					1	1	464.455
		cgd7_3380	putative secreted cysteine rich protein, possible transmembrane domain near C, signal peptide			1			1	3.418
		cgd7_3560	hypothetical protein, signal peptide, possible 3 transmembrane domains	1					1	0.372
		cgd7_3910	cysteine rich protein with two potential transmembrane domain regions	1					1	0.374
		cgd7_4160	extracellular protein with a signal peptide followed by family 2 glycosyltransferase and ricin domains	1					1	0.533
		cgd7_4310	extracellular protein with a signal peptide and 4 SCP domains			1			1	3.296
		cgd7_4540	CP15/60 sporozoite 60K protein			1			1	4.289
		cgd7_4610	divergent membrane protein				1		1	5.624
		cgd7_4810	CpFNPA, extracellular protein with a signal peptide, FN2 domain and an anthrax toxin-N-terminus like domain. starts at second Met with signal peptide following			1			1	5.244
		cgd7_4880	signal peptide plus GPI anchored membrane protein					1	1	36.784
		cgd7_4910	conserved 3 transmembrane domain membrane associated RING finger domain (shared by plants and apicomplexans)				1		1	8.880
		cgd7_4920	membrane protein	1					1	0.326
		cgd7_5140	A surface protein with 2 conserved cysteines		1				1	1.658
		cgd7_5430	possible 2 transmembrane domains, low complexity protein	1					1	0.369
		cgd7_5480	very large membrane protein				1		1	5.866
		cgd7_550	hypothetical protein wtih 9 transmembrane domains, possible apicomplexan-specific protein				1		1	5.913

	cgd7_70	integral membrane protein with 6 transmembrane domains and possible ring domain	1					1	0.365
	cgd7_720	PASILLA splice variant 3-like 2KH domains, transmembrane domain at C-terminus	1					1	0.530
	cgd7_900	hypothetical protein with 3 or more transmembrane domains	1					1	0.286
	cgd7_930	hypothetical transmembrane protein	1					1	0.366
	cgd8_1160	signal peptide plus His and thr repeats, signal peptide plus transmembrane domain or GPI anchor, predicted mucin			1			1	5.490
	cgd8_150	CpTSP4; extracellular protien with signal peptide and 2 repeats of an apple domain followed by a TSP1 domain	1					1	0.280
	cgd8_1900	predicted transmembrane domain, transmembrane domain within N-terminus	1					1	0.315
	cgd8_2280	uncharacterized protein with 5 transmembrane domain					1	1	40.631
	cgd8_240	hypothetical protein with 6 transmembrane domains	1					1	0.563
	cgd8_2470	hypothetical protein, signal peptide, 4 transmembrane domains					1	1	31.094
	cgd8_2480	signal peptide plus possible transmembrane domain paralogs				1		1	28.197
	cgd8_2500	hypothetical protein, signal peptide, membrane protein, transmembrane domain at C-terminus, paralogs				1		1	7.175
	cgd8_2790	pleckstrin homology (PH domain) containing protein					1	1	27.645
	cgd8_2910	ferlin-like type II membrane associated protein with 5 C2 domains, transmembrane domain or GPI anchor at C-terminus	1					1	0.374
	cgd8_2950	cervesiae Yh1017wp/PTM1 like protein; signal peptide plus 7 pass transmembrane regions		1				1	1.639
	cgd8_3000	SYG1/ ERD1 like integral membrane protein required for retention of ER lumen proteins, with 8-10 transmembrane domains				1		1	15.278
	cgd8_3470	membrane associated protein with a RING finger, 4xtransmembrane domain					1	1	215.651
	cgd8_3620	hypothetical protein with 6 transmembrane domains	1					1	0.528
	cgd8_3760	predicted membrane associated protein, signal peptide, transmembrane domain near C			1			1	2.684
	cgd8_3890	membrane protein with signal peptide plus 12x transmembrane domains				1		1	13.850
	cgd8_3920	hypothetical protein, signal peptide, predicted membrane protein, transmembrane domain near C-terminus	1					1	0.387
	cgd8_4090	hypothetical protein, signal peptide + 5 transmembrane domains					1	1	28.561
	cgd8_4160	hypothetical protein, transmembrane domain			1			1	3.755
	cgd8_4410	hypothetical protein, possible transmembrane domain				1		1	16.317
	cgd8_4440	protein with 12x transmembrane regions	1					1	0.404
	cgd8_4880	3 transmembrane region protein					1	1	124.333
	cgd8_5090	hypothetical protein, possible transmembrane domain within N-terminus	1					1	0.427
	cgd8_5140	possible coated vesicle proteins, emp24/gp25L/p24 family, signal peptide, possible transmembrane domain at C-terminus	1					1	0.340
	cgd8_5200	5 transmembrane domain proteiin					1	1	47.298
	cgd8_5220	signal peptide, 4+ transmembrane domains	1					1	0.367
	cgd8_5270	integral membrane protein predicted amino acid permease, 11x transmembrane domains	1					1	0.371
	cgd8_530	hypothetical protein with 4 transmembrane domains		1				1	1.952
	cgd8_540	CpTSP12; extracellular protein with 1 TSP1 domain	1					1	0.435
	cgd8_60	signal peptide, possible transmembrane domain near N					1	1	175.821
	cgd8_850	predicted membrane associated protein with 8 transmembrane domains	1					1	0.484
Membrane Total			92	41	36	33	34	236	21.029
Unknown	cgd1_100	hypothetical protein	1					1	0.413
	cgd1_1000	hypothetical protein	1					1	0.377
	cgd1_1020	hypothetical protein	1					1	0.422
	cgd1_1040	hypothetical protein	1					1	0.386
	cgd1_1060	hypothetical protein	1					1	0.433
	cgd1_1070	RRM domain protein			1			1	3.300
	cgd1_110	predicted secreted protein, signal peptide, paralogs				1		1	10.376
	cgd1_1150	hypothetical protein	1					1	0.360
	cgd1_1190	hypothetical protein	1					1	0.350
	cgd1_1200	hypothetical protein					1	1	220.734
	cgd1_1240	Sec14 domain containing protein	1					1	0.465
	cgd1_1250	predicted secreted protein, signal peptide	1					1	0.400
	cgd1_1260	hypothetical protein	1					1	0.348
	cgd1_1290	apicomplexa specific secreted protein Pf (23508265), signal peptide				1		1	16.723
	cgd1_130	signal peptide, predicted secreted protein with a cysteine cluster at the C-terminus					1	1	51.078
	cgd1_1320	developmental protein, putative	1					1	0.426
	cgd1_1390	hypothetical protein	1					1	0.424
	cgd1_1400	hypothetical protein	1					1	0.418
	cgd1_1430	hypothetical protein	1					1	0.425
	cgd1_1440	hypothetical protein		1				1	1.758
	cgd1_1450	predicted secreted protein, signal peptide					1	1	42.773
	cgd1_1460	hypothetical protein	1					1	0.466
	cgd1_1490	NEK2 protein, putative	1					1	0.447
	cgd1_150	predicted secreted protein, signal peptide			1			1	3.693

		cgd1_1500	hypothetical protein		1				1	1.336
		cgd1_1510	predicted secreted protein, signal peptide		1				1	1.523
		cgd1_1590	hypothetical protein				1		1	22.164
		cgd1_1600	histidine triad (HIT) family zinc binding protein					1	1	123.796
		cgd1_1630	hypothetical protein				1		1	8.652
		cgd1_1650	hypothetical protein	1					1	0.438
		cgd1_1690	PHD finger containing protein	1					1	0.450
		cgd1_170	hypothetical protein	1					1	0.478
		cgd1_1740	hypothetical coiled coil protein	1					1	0.338
		cgd1_1780	hypothetical protein		1				1	1.997
		cgd1_1790	RING-H2 finger containing membrane associated protein, 8x transmembrane domain	1					1	0.621
		cgd1_1810	hypothetical protein	1					1	0.381
		cgd1_1840	hypothetical protein					1	1	151.632
		cgd1_1850	G-patch domain containing protein	1					1	0.407
		cgd1_1880	hypothetical protein	1					1	0.305
		cgd1_190	narf-like protein nuclear prelamin A recognition factor	1					1	0.482
		cgd1_1920	large protein with a SPRY domain and HECT domain			1			1	2.524
		cgd1_1940	possible RAN guanine nucleotide release factor			1			1	4.637
		cgd1_1950	hypothetical protein					1	1	25.027
		cgd1_1970	hypothetical protein			1			1	3.612
		cgd1_1990	hypothetical protein			1			1	3.460
		cgd1_2000	predicted coiled coil protein					1	1	86.923
		cgd1_2070	hypothetical protein	1					1	0.460
		cgd1_2140	hypothetical protein		1				1	1.803
		cgd1_2150	hypothetical protein with GYF domain at the amino terminus	1					1	0.361
		cgd1_2160	hypothetical protein with BTB/POZ domain and GYF domain	1					1	0.368
		cgd1_2170	SET domain containing protein with a cysteine cluster at the C-terminus				1		1	11.333
		cgd1_2200	hypothetical protein	1					1	0.326
		cgd1_2210	hypothetical protein		1				1	1.721
		cgd1_2230	WD40 protein Ciao1	1					1	0.357
		cgd1_2240	AT hook motif protein, putative	1					1	0.684
		cgd1_2290	predicted coiled coil protein		1				1	1.339
		cgd1_2310	unnamed protein product		1				1	1.643
		cgd1_2330	hypothetical protein with predicted SANT domain		1				1	1.250
		cgd1_2340	hypothetical protein	1					1	0.309
		cgd1_2350	hypothetical protein				1		1	16.095
		cgd1_2390	hypothetical secreted/membrane associated protein signal peptide plus possible GPI signal		1				1	1.927
		cgd1_240	predicted secreted protein			1			1	5.573
		cgd1_2400	signal peptide, secreted protein					1	1	226.789
		cgd1_2440	eukaryotic orthologous group, signal peptide	1	1				2	1.052
		cgd1_2460	hypothetical protein					1	1	47.223
		cgd1_2480	hypothetical protein			1			1	3.837
		cgd1_2500	signal recognition particle SPR68	1					1	0.467
		cgd1_2520	hypothetical protein	1					1	0.463
		cgd1_2570	possible conserved eukaryotic alpha beta hydrolase			1			1	2.339
		cgd1_2590	PGPD14 protein with at least one predicted RING finger, possible plant origin	1					1	0.340
		cgd1_260	predicted secreted protein, signal peptide	1					1	0.380
		cgd1_2610	hypothetical protein	1					1	0.401
		cgd1_2650	hypothetical protein		1				1	1.890
		cgd1_2670	ankyrin-related protein, putative		1				1	1.765
		cgd1_2790	hypothetical protein	1					1	0.308
		cgd1_2810	S. pombe like (SPAC1805) eukaryotic protein with a possible metal binding domain at the N-terminus	1					1	0.308
		cgd1_2860	hypothetical protein	1					1	0.386
		cgd1_2870	N-terminal domain cerevisiae Yos9p-like that is involved in GPI-anchored protein transport in the golgi, a cation independent mannose-6-po4 receptor like conserved Cys motif and a C-terminal E. coli y	1					1	0.609
		cgd1_2900	hypothetical protein			1			1	2.824
		cgd1_2930	hypothetical protein	1					1	0.395
		cgd1_2940	hypothetical protein	1					1	0.335
		cgd1_2950	hypothetical protein			1			1	5.829
		cgd1_3030	hypothetical protein containing WD40 repeats fused to a RING finger	1	1				2	0.842
		cgd1_3090	very low complexity hypothetical protein, possible unreal ORF	1					1	0.239
		cgd1_3100	hypothetical protein		1				1	1.628
		cgd1_3110	hypothetical protein	1					1	0.295
		cgd1_3120	peptide synthase like condensation domain	1					1	0.393
		cgd1_3180	hypothetical protein	1					1	0.394
		cgd1_3220	erythrocyte membrane-associated antigen	1					1	0.364
		cgd1_3260	hypothetical protein				1		1	6.565
		cgd1_3270	hypothetical protein					1	1	59.300
		cgd1_3280	hypothetical protein			1			1	4.890
		cgd1_3300	ring domain protein			1			1	2.979

		cgd1_3320	ring domain protein	1					1	0.346
		cgd1_3330	hypothetical protein	1					1	0.323
		cgd1_3350	hypothetical protein	1					1	0.279
		cgd1_3360	predicted AFG1 ATpase family AAA ATpase	1					1	0.386
		cgd1_3400	hypothetical protein		1				1	1.981
		cgd1_3430	hypothetical protein	1					1	0.414
		cgd1_3450	hypothetical protein		1				1	1.340
		cgd1_3520	hypothetical protein			1			1	2.558
		cgd1_3540	TPR repeat proteini				1		1	13.434
		cgd1_3560	hypothetical protein	1					1	0.345
		cgd1_3580	hypothetical protein	1					1	0.511
		cgd1_3640	hypothetical protein, signal peptide					1	1	87.129
		cgd1_3650	hypothetical protein, signal peptide					1	1	34.068
		cgd1_3660	hypothetical protein	1					1	0.577
		cgd1_3740	ankyrin repeat protein with over 9 transmembrane domains at C-terminus, signal peptide	1					1	0.330
		cgd1_3760	hypothetical coiled coil protein	1					1	0.293
		cgd1_3770	hypothetical protein					1	1	79.010
		cgd1_3790	hypothetical protein, signal peptide				1		1	6.004
		cgd1_3830	hypothetical protein	1					1	0.495
		cgd1_3860	hypothetical protein					1	1	98.631
		cgd1_440	hypothetical protein			1			1	3.025
		cgd1_450	hypothetical protein		1				1	1.937
		cgd1_460	hypothetical protein	1					1	0.422
		cgd1_470	predicted secreted protein, signal peptide, low complexity serine-threonine rich, possible mucin			1			1	5.540
		cgd1_490	hypothetical protein	1					1	0.474
		cgd1_50	hypothetical protein with 2 TPR domains	1					1	0.366
		cgd1_500	hypothetical protein	1					1	0.472
		cgd1_510	hypothetical protein	1					1	0.406
		cgd1_530	hypothetical protein					1	1	38.741
		cgd1_560	hypothetical protein		1				1	1.484
		cgd1_570	predicted secreted protein, signal peptide	1					1	0.373
		cgd1_590	hypothetical protein, proline rich C-terminus, signal peptide, very large protein	1	1				2	1.176
		cgd1_610	hypothetical protein				1		1	12.825
		cgd1_620	hypothetical protein		1				1	1.485
		cgd1_630	hypothetical protein			1			1	3.936
		cgd1_640	signal peptide, large protein					1	1	1,146.669
		cgd1_650	predicted secreted protein, signal peptide	1					1	0.390
		cgd1_660	predicted secreted protein, signal peptide	1					1	0.646
		cgd1_670	predicted secreted protein, signal peptide		1				1	1.525
		cgd1_680	hypothetical protein					1	1	26.652
		cgd1_690	hypothetical protein		1				1	1.966
		cgd1_70	hypothetical protein		1				1	1.461
		cgd1_710	coiled coil protein				1		1	5.831
		cgd1_740	hypothetical protein	1					1	0.475
		cgd1_910	ZnF A20 and Znf AN1 domains, involved in signaling					1	1	249.040
		cgd1_940	hypothetical protein	1					1	0.632
		cgd1_960	RING finger containing protein			1			1	2.614
		cgd1_970	RING finger containing protein			1			1	4.263
		cgd2_10	hypothetical protein	1					1	0.349
		cgd2_1040	flavohemoprotein b5+b5R (DJ676J13.1) (flavohemoprotein b5+b5R cytochrome b-type NAD(P)H oxidoreductase), putative	1					1	0.363
		cgd2_1050	signal peptide					1	1	65.673
		cgd2_1080	hypothetical protein		1				1	1.289
		cgd2_1110	silencer-associated factor, putative	1					1	0.360
		cgd2_1130	hypothetical protein	1					1	0.400
		cgd2_1150	hypothetical protein					1	1	41.522
		cgd2_1170	zinc finger protein				1		1	12.855
		cgd2_1230	hypothetical protein			1			1	2.711
		cgd2_1260	hypothetical protein	1					1	0.436
		cgd2_1280	hypothetical protein	1					1	0.405
		cgd2_1370	hypothetical coiled coil protein			1			1	4.253
		cgd2_1380	hypothetical protein	1					1	0.498
		cgd2_1390	hypothetical protein			1			1	3.279
		cgd2_1410	possible domain KOG3410, conserved alpha-helical protein					1	1	29.743
		cgd2_1420	hypothetical protein				1		1	13.272
		cgd2_1470	hypothetical protein	1					1	0.434
		cgd2_1490	hypothetical protein with signal peptide and 7 transmembrane domains	1					1	0.381
		cgd2_1520	hypothetical protein					1	1	93.538
		cgd2_1560	hypothetical protein		1				1	1.795
		cgd2_1670	ING1 ike protein with an N-terminal globular domain and a PHD domain	1					1	0.296
		cgd2_1680	hypothetical protein				1		1	6.113
		cgd2_1720	hypothetical protein	1					1	0.350

		cgd2_1730	hypothetical protein	1					1	0.418
		cgd2_1740	RSC8 ortholog with a swirm domain, ZZ finger and Myb			1			1	2.541
		cgd2_1750	ring domain		1				1	1.608
		cgd2_1790	hypothetical protein			1			1	4.008
		cgd2_180	hypothetical protein				1		1	7.810
		cgd2_1820	hypothetical protein					1	1	24.622
		cgd2_1830	hypothetical protein	1	1				2	1.202
		cgd2_1840	signal peptide					1	1	133.856
		cgd2_1860	TBC domain containing protein			1			1	5.556
		cgd2_1870	guanine nucleotide-binding protein, putative					1	1	66.422
		cgd2_1890	hypothetical protein		1				1	1.504
		cgd2_1920	hypothetical protein	1					1	0.324
		cgd2_1950	SAP+PHD+MIZ domains containing protein	1					1	0.468
		cgd2_1970	hypothetical protein	1					1	0.374
		cgd2_1980	hypothetical protein		1				1	1.526
		cgd2_2010	hypothetical protein	1					1	0.628
		cgd2_2020	hypothetical protein	1					1	0.443
		cgd2_2080	hypothetical protein		1				1	1.483
		cgd2_2100	hypothetical protein, possible signal peptide			1			1	2.637
		cgd2_2110	hypothetical protein, possible signal peptide			1			1	2.406
		cgd2_2150	hypothetical protein			1			1	3.148
		cgd2_2180	hypothetical protein		1				1	1.494
		cgd2_2270	hypothetical protein, signal peptide		1				1	1.380
		cgd2_2330	hypothetical protein	1					1	0.345
		cgd2_2350	MA3 domain containing protein					1	1	34.524
		cgd2_2360	Dop1p family protein	1					1	0.581
		cgd2_2370	hypothetical protein	1					1	0.329
		cgd2_2380	hypothetical protein	1					1	0.471
		cgd2_2410	SSM4 like RING finger protein				1		1	10.936
		cgd2_2440	hypothetical protein					1	1	123.210
		cgd2_2450	hypothetical protein					1	1	29.351
		cgd2_2460	hypothetical protein		1				1	2.039
		cgd2_2520	hypothetical protein				1		1	14.384
		cgd2_2530	hypothetical protein, signal peptide			1			1	4.807
		cgd2_2550	hypothetical protein, signal peptide	1	1				2	1.117
		cgd2_2560	hypothetical protein, signal peptide					1	1	57.962
		cgd2_2570	hypothetical protein, possible signal peptide					1	1	31.925
		cgd2_2580	hypothetical protein			1			1	4.451
		cgd2_2590	hypothetical protein	1					1	0.323
		cgd2_260	hypothetical protein					1	1	94.265
		cgd2_2600	hypothetical protein				1		1	6.383
		cgd2_2620	hypothetical protein		1				1	2.236
		cgd2_2640	hypothetical protein			1			1	3.430
		cgd2_2690	hypothetical protein				1		1	20.841
		cgd2_2700	Brf1p like coiled coil protein			1			1	5.475
		cgd2_2720	ARM repeat protein			1			1	3.969
		cgd2_2740	hypothetical protein				1		1	7.770
		cgd2_2800	THUMP RNA binding domain containing protein, Yg1232wp-like			1			1	2.839
		cgd2_2850	hypothetical protein, possible signal peptide	1					1	0.435
		cgd2_290	hypothetical protein	1					1	0.432
		cgd2_2910	large WD repeat protein			1			1	4.738
		cgd2_2930	hypothetical protein		1				1	1.729
		cgd2_2950	ring domain at very C-terminus of large protein		1				1	1.474
		cgd2_2980	hypothetical protein			1			1	4.842
		cgd2_300	kelch repeat-containing proteins that is fused to a HSP90-like ATpase	1					1	0.396
		cgd2_3020	hypothetical protein	1					1	0.525
		cgd2_3030	hypothetical protein, signal peptide	1					1	0.423
		cgd2_3050	hypothetical protein		1				1	1.643
		cgd2_3060	hypothetical protein			1			1	3.288
		cgd2_3090	hypothetical protein containing a signal peptide				1		1	7.387
		cgd2_3110	hypothetical protein containing a signal peptide					1	1	147.737
		cgd2_3130	hypothetical protein with possible transcriptional repressor EZH1 domain	1					1	0.507
		cgd2_3140	hypothetical protein	1					1	0.478
		cgd2_3160	hypothetical protein	1					1	0.338
		cgd2_3210	hypothetical protein			1			1	2.672
		cgd2_3220	Sc6p-like, SM domain				1		1	10.398
		cgd2_3240	ARF GAP-like zinc finger-containing protein	1					1	0.597
		cgd2_3250	3x TPR domain-containing protein		1				1	1.843
		cgd2_3280	hypothetical protein	1					1	0.251
		cgd2_3290	hypothetical protein with a signal peptide plus thr stretch, possible mucin					1	1	25.356
		cgd2_330	notchless				1		1	10.941
		cgd2_3300	hypothetical protein	1					1	0.288
		cgd2_3310	hypothetical protein		1				1	1.874

		cgd2_3350	hypothetical protein			1			1	2.668
		cgd2_3360	hypothetical protein	1					1	0.383
		cgd2_340	signal peptide, large protein				1		1	6.139
		cgd2_3430	SDA1-like protein				1		1	24.345
		cgd2_3460	C- terminal region conserved, zinc finger, myb DNA binding domain	1					1	0.386
		cgd2_3490	protein with low complexity regions and an apicomplexan specific domain			1			1	3.475
		cgd2_3530	possible RNA binding motif, signal peptide	1					1	0.316
		cgd2_3610	2x WD domain containing protein	1					1	0.417
		cgd2_3630	hypothetical protein	1					1	0.500
		cgd2_3640	hypothetical protein with region conserved in apicomplexa					1	1	26.158
		cgd2_3690	WD repeat protein			1			1	2.648
		cgd2_3710	Pfa MAL6P1.309 like protein	1					1	0.418
		cgd2_3720	hypothetical protein	1					1	0.435
		cgd2_3740	hypothetical protein, signal peptide	1					1	0.482
		cgd2_3780	uncharacterized protein rich in Thr and Ser residues		1				1	1.793
		cgd2_3800	hypothetical protein	1					1	0.421
		cgd2_3820	hypothetical protein	1					1	0.307
		cgd2_3830	hypothetical protein			1			1	3.677
		cgd2_3840	hypothetical protein		1				1	1.645
		cgd2_3850	possible FH2 formin homology domain	1					1	0.299
		cgd2_3860	BBOX Zn finger domain containing protein		1				1	2.050
		cgd2_390	hypothetical protein		1				1	1.817
		cgd2_3910	hypothetical protein			1			1	2.363
		cgd2_3980	Myb domain containing protein			1			1	4.911
		cgd2_4010	MAL7P1.13-like			1			1	4.138
		cgd2_4020	signal peptide				1		1	17.160
		cgd2_4100	hypothetical protein					1	1	392.489
		cgd2_4110	possible UBC domain containing protein	1					1	0.340
		cgd2_4160	hypothetical protein having possible hemolytic activity					1	1	25.970
		cgd2_4180	hypothetical protein			1			1	3.574
		cgd2_4200	hypothetical protein		1				1	2.204
		cgd2_4210	uncharacterized protein with several coiled coil regions	1					1	0.579
		cgd2_4220	hypothetical protein		1				1	2.245
		cgd2_4250	SPAC1486.03c-like tuftelin-interacting protein, G-patch domain	1					1	0.474
		cgd2_4290	hypothetical protein				1		1	8.404
		cgd2_4310	hypothetical protein					1	1	342.642
		cgd2_4360	hypothetical protein					1	1	35.148
		cgd2_4370	signal peptide					1	1	37.937
		cgd2_4380	signal peptide, repeats, gene anchored to telomere				1		1	7.503
		cgd2_470	very low complexity large protein, possible unreal ORF?				1		1	19.177
		cgd2_480	hypothetical protein				1		1	6.723
		cgd2_490	hypothetical protein with signal peptide				1		1	14.691
		cgd2_500	hypothetical protein	1					1	0.365
		cgd2_530	possible proteasome component	1					1	0.413
		cgd2_560	large low complexity protein	1					1	0.434
		cgd2_60	hypothetical protein			1			1	4.221
		cgd2_680	hypothetical protein	1					1	0.347
		cgd2_690	large low complexity protein with glycine-rich repeats			1			1	5.312
		cgd2_720	hypothetical protein				1		1	5.633
		cgd2_750	bub3'bub3-like protein with WD40 repeats'	1					1	0.486
		cgd2_770	hypothetical protein					1	1	25.904
		cgd2_780	hypothetical protein				1		1	17.990
		cgd2_850	hypothetical protein, signal peptide		1				1	2.326
		cgd2_870	drosophila CG6013 like HMG domain containing protein with a coiled coil region at the N-terminus				1		1	12.682
		cgd2_910	hypothetical protein	1					1	0.477
		cgd2_950	hypothetical protein	1					1	0.408
		cgd2_970	hypothetical protein		1				1	1.472
		cgd3_100	hypothetical protein with signal peptide	1					1	0.363
		cgd3_1020	hypothetical protein				1		1	9.711
		cgd3_1030	uncharacterized low complexity protein	1					1	0.456
		cgd3_1040	hypothetical protein	1					1	0.282
		cgd3_1050	hypothetical protein	1					1	0.439
		cgd3_1080	hypothetical protein	1					1	0.272
		cgd3_1100	hypothetical protein	1					1	0.381
		cgd3_1130	hypothetical protein	1					1	0.304
		cgd3_120	hypothetical protein	1					1	0.431
		cgd3_1200	possible prefoldin-related protein				1		1	6.512
		cgd3_1210	hypothetical protein having a signal peptide, conserved region, and three or more transmembrane domains	1					1	0.459
		cgd3_1220	hypothetical protein					1	1	51.370
		cgd3_1240	hypothetical protein	1					1	0.448
		cgd3_1260	ring domain protein			1			1	5.428
		cgd3_1280	hypothetical protein	1					1	0.531

		cgd3_130	DRG like OBG family GTpase fused to an RNA binding domain TGS domain, Fun11p	1					1	0.379
		cgd3_1310	hypothetical protein	1					1	0.432
		cgd3_1330	hypothetical protein having a signal peptide		1				1	1.879
		cgd3_1390	hypothetical protein		1				1	1.659
		cgd3_1410	small hypothetical protein					1	1	44.129
		cgd3_1460	GAF domain containing protein	1					1	0.455
		cgd3_1490	hypothetical protein	1					1	0.363
		cgd3_150	hypothetical protein	1					1	0.564
		cgd3_1500	hypothetical protein				1		1	13.340
		cgd3_1530	hypothetical protein				1		1	16.831
		cgd3_1540	large protein with signal peptide. cysteine-rich, threonine-rich, possible mucin	1					1	0.475
		cgd3_1550	Sec14 domain containing protein			1			1	5.563
		cgd3_1570	sporozoite antigen, putative					1	1	1,957.286
		cgd3_160	hypothetical protein					1	1	36.880
		cgd3_1630	hypothetical protein				1		1	5.622
		cgd3_170	hypothetical protein				1		1	8.071
		cgd3_1830	hypothetical protein having a signal peptide	1					1	0.476
		cgd3_1840	large uncharacterized protein	1					1	0.438
		cgd3_1880	protein with 2x rrm domains		1				1	2.163
		cgd3_190	latent transforming growth factor beta binding protein like (3F865)			1			1	4.444
		cgd3_1910	hypothetical protein	1					1	0.421
		cgd3_1970	hypothetical protein	1					1	0.495
		cgd3_1980	hypothetical protein				1		1	10.129
		cgd3_1990	hypothetical protein	1					1	0.456
		cgd3_20	hypothetical protein containing a signal peptide	1					1	0.395
		cgd3_2000	WD repeat protein	1					1	0.419
		cgd3_2010	hypothetical protein	1					1	0.380
		cgd3_2060	ring domain protein		1				1	1.443
		cgd3_2070	hypothetical protein				1		1	30.385
		cgd3_210	hypothetical protein	1					1	0.518
		cgd3_2130	hypothetical protein with signal peptide		1				1	1.219
		cgd3_2140	hypothetical protein		1				1	1.720
		cgd3_220	hypothetical protein	1	1				2	1.201
		cgd3_2230	hypothetical protein				1		1	10.783
		cgd3_2260	hypothetical protein					1	1	28.261
		cgd3_2270	WD repeat protein			1			1	3.945
		cgd3_2310	hypothetical protein			1			1	5.446
		cgd3_2330	hypothetical protein					1	1	238.164
		cgd3_2340	hypothetical protein				1		1	14.584
		cgd3_2380	hypothetical protein	1					1	0.595
		cgd3_240	hypothetical protein	1					1	0.456
		cgd3_2400	Yn1201cp-family protein		1				1	2.078
		cgd3_2470	hypothetical protein					1	1	236.906
		cgd3_2480	hypothetical protein	1					1	0.487
		cgd3_2490	hypothetical protein	1					1	0.403
		cgd3_2510	myb domain-containing protein		1				1	1.971
		cgd3_2550	hypothetical protein with carboxy terminus motif shared with DNA-directed RNA polymerase subunit and TFIIS				1		1	11.483
		cgd3_260	hypothetical protein	1					1	0.430
		cgd3_2640	hypothetical protein with conserved domain					1	1	35.087
		cgd3_2660	hypothetical protein				1		1	11.451
		cgd3_2700	Trafficking protein particle complex subunit 6B, putative	1					1	0.326
		cgd3_2750	hypothetical protein			1			1	4.007
		cgd3_2800	hypothetical protein				1		1	12.280
		cgd3_2830	hypothetical protein				1		1	8.344
		cgd3_2860	hypothetical protein	1					1	0.478
		cgd3_2890	Prp8. JAB/PAD domain			1			1	3.159
		cgd3_3000	TBC domain containing protein			1			1	4.058
		cgd3_3010	hypothetical protein			1			1	5.372
		cgd3_3050	hypothetical protein	1					1	0.422
		cgd3_3070	WD-40 repeat protein, SMART LisH domain	1					1	0.362
		cgd3_3080	hypothetical protein				1		1	8.899
		cgd3_3130	hypothetical protein	1					1	0.418
		cgd3_3140	hypothetical protein	1					1	0.449
		cgd3_3200	hypothetical protein			1			1	2.487
		cgd3_3210	PV1H14080_P	1					1	0.416
		cgd3_3240	hypothetical protein	1					1	0.464
		cgd3_3260	hypothetical protein			1			1	4.404
		cgd3_3290	hypothetical protein with possible signal peptide and one transmembrane region	1					1	0.389
		cgd3_3330	hypothetical protein	1					1	0.570
		cgd3_3350	hypothetical protein				1		1	12.080
		cgd3_3370	large hypothetical protein with signal peptide				1		1	18.710
		cgd3_3400	hypothetical protein	1					1	0.386

		cgd3_3410	hypothetical protein	1					1	0.301
		cgd3_3450	hypothetical protein	1					1	0.351
		cgd3_3460	hypothetical protein with cysteine rich regions		1				1	1.620
		cgd3_3470	proliferating cell nuclear antigen	1					1	0.416
		cgd3_3480	large Sec7 domain containing protein	1					1	0.300
		cgd3_3510	hypothetical protein			1			1	2.332
		cgd3_3540	hypothetical protein	1					1	0.459
		cgd3_3580	DRG-like OBG family GTpase fused to an RNA binding domain TGS domain, Fun11p				1		1	16.266
		cgd3_360	hypothetical protein				1		1	7.058
		cgd3_3640	hypothetical protein having a signal peptide	1					1	0.344
		cgd3_3680	UIM domain and EF hand containing protein that also has a conserved domain between				1		1	10.058
		cgd3_3710	hypothetical protein	1					1	0.342
		cgd3_3760	small protein with possible EF hand domains, calmodulin like	1	1				2	0.866
		cgd3_3780	hypothetical protein			1			1	6.539
		cgd3_3830	hypothetical protein				1		1	22.761
		cgd3_3860	coiled coil protein		1				1	2.001
		cgd3_3900	hypothetical protein			1			1	2.564
		cgd3_3990	hypothetical protein	1					1	0.325
		cgd3_400	hypothetical protein			1			1	2.890
		cgd3_4000	hypothetical protein	1		1			2	1.539
		cgd3_4040	hypothetical protein with signal peptide, proline rich			1			1	3.122
		cgd3_4120	hypothetical protein			1			1	4.765
		cgd3_4130	hypothetical protein	1					1	0.382
		cgd3_420	hypothetical protein	1					1	0.363
		cgd3_4230	hypothetical protein, signal peptide	1					1	0.498
		cgd3_480	hypothetical protein with a signal peptide				1		1	18.854
		cgd3_530	large hypothetical protein with signal peptide			1			1	3.456
		cgd3_60	hypothetical protein	1					1	0.386
		cgd3_630	hypothetical protein with signal peptide	1					1	0.402
		cgd3_650	hypothetical protein, signal peptide			1			1	4.158
		cgd3_660	hypothetical protein, signal peptide	1					1	0.368
		cgd3_70	hypothetical protein					1	1	43.229
		cgd3_710	large hypothetical protein with signal peptide		1				1	1.597
		cgd3_720	very large probable mucin, 11700 aa long protein with signal peptide and pronounced Thr repeat (308 aa long)	1					1	0.349
		cgd3_750	hypothetical protein					1	1	256.523
		cgd3_780	hypothetical protein				1		1	6.367
		cgd3_790	hypothetical protein	1					1	0.317
		cgd3_820	hypothetical protein	1					1	0.672
		cgd3_840	RSC6/BAF60A ortholog with a SWIB domain				1		1	10.051
		cgd3_850	hypothetical protein			1			1	4.512
		cgd3_870	flavoprotein, putative	1					1	0.453
		cgd3_880	hypothetical protein					1	1	214.247
		cgd3_90	hypothetical protein with signal peptide	1					1	0.395
		cgd3_900	hypothetical protein	1					1	0.419
		cgd3_910	hypothetical protein	1					1	0.509
		cgd3_940	hypothetical protein	1					1	0.349
		cgd3_960	hypothetical protein			1			1	2.526
		cgd3_980	hypothetical protein					1	1	121.126
		cgd4_100	hypothetical protein	1					1	0.340
		cgd4_1000	hypothetical protein	1					1	0.349
		cgd4_1020	hypothetical protein	1					1	0.314
		cgd4_1070	MYND finger domain protein				1		1	21.170
		cgd4_110	hypothetical protein		1				1	1.489
		cgd4_1100	hypothetical protein			1			1	2.343
		cgd4_1180	hypothetical protein	1					1	0.301
		cgd4_120	WD repeat protein	1					1	0.320
		cgd4_1210	Ms15p; KH + 2 Znknuckle (C2HC)			1			1	5.235
		cgd4_1240	hypothetical protein			1			1	4.555
		cgd4_1250	hypothetical protein	1					1	0.244
		cgd4_1260	possible tRNA-INTRON ENDONUCLEASE	1					1	0.393
		cgd4_130	RRM domain and KH domain protein (SPAC30D11.14-like KH)	1					1	0.390
		cgd4_1300	uncharacterized secreted protein with thr rich regions, possible mucin	1					1	0.415
		cgd4_1420	hypothetical protein		1				1	1.929
		cgd4_1430	hypothetical protein	1					1	0.402
		cgd4_1460	hypothetical protein	1					1	0.370
		cgd4_1500	hypothetical protein		1				1	1.399
		cgd4_1540	hypothetical protein					1	1	47.307
		cgd4_1560	hypothetical protein					1	1	36.056
		cgd4_1620	hypothetical protein			1			1	3.778
		cgd4_1630	hypothetical protein	1					1	0.424
		cgd4_1640	hypothetical protein	1					1	0.438
		cgd4_170	hypothetical protein				1		1	10.594
		cgd4_1710	Low complexity protein			1			1	3.354

		cgd4_1770	hypothetical protein			1			1	2.566
		cgd4_180	hypothetical protein	1					1	0.456
		cgd4_1810	hypothetical protein	1					1	0.410
		cgd4_1870	hypothetical protein	1					1	0.433
		cgd4_1880	hypothetical protein	1					1	0.488
		cgd4_1900	hypothetical protein	1					1	0.323
		cgd4_1930	possible origin recognition complex protein subunit 2, orc2	1					1	0.515
		cgd4_1980	TBC domain containing protein		1				1	1.656
		cgd4_1990	RRp1-like protein					1	1	40.954
		cgd4_200	large low complexity coiled coil protien with large repeat region					1	1	24.512
		cgd4_2040	SMART RPR domain at NH2 terminus, SMART AZnF_C2H2	1					1	0.388
		cgd4_2120	PX domain containing protein	1					1	0.431
		cgd4_2130	TBC domain containing protein				1		1	13.805
		cgd4_2140	hypothetical protein		1				1	2.181
		cgd4_2150	hypothetical protein					1	1	28.948
		cgd4_2170	hypothetical protein	1					1	0.415
		cgd4_2200	SMC2 protein	1					1	0.444
		cgd4_2220	possible double-stranded DNA-binding domain, small conserved protein	1					1	0.425
		cgd4_2230	uncharacterized protein	1					1	0.481
		cgd4_2240	hypothetical protein	1					1	0.333
		cgd4_2280	hypothetical protein				1		1	23.834
		cgd4_2290	hypothetical protein				1		1	10.438
		cgd4_230	hypothetical protein containing a signal peptide	1					1	0.378
		cgd4_2310	hypothetical protein	1					1	0.441
		cgd4_2320	At3g18860/MCB22_3	1					1	0.394
		cgd4_2350	multitransmembrane protein with signal peptide and GMGPP repeat at C-terminus	1					1	0.492
		cgd4_2380	possible SAND family protein	1					1	0.457
		cgd4_2410	hypothetical protein		1				1	1.909
		cgd4_2440	small hypothetical protein with signal peptide				1		1	8.578
		cgd4_2460	hypothetical protein with a signal peptide, gene within locus of signal-peptide containing hypothetical proteins					1	1	160.198
		cgd4_2470	hypothetical protein with a signal peptide, gene within locus of signal-peptide containing hypothetical proteins			1			1	2.909
		cgd4_2480	hypothetical protein with a signal peptide, gene within locus of signal-peptide containing hypothetical proteins			1			1	2.488
		cgd4_2510	hypothetical protein with a signal peptide, gene within locus of signal-peptide containing hypothetical proteins		1				1	1.984
		cgd4_2520	hypothetical protein with a signal peptide, gene within locus of signal-peptide containing hypothetical proteins				1		1	17.848
		cgd4_2570	WD repeat protein			1			1	3.278
		cgd4_260	WD repeat protein				1		1	10.152
		cgd4_2610	hypothetical protein				1		1	9.421
		cgd4_2620	hypothetical protein		1				1	1.455
		cgd4_2630	hypothetical protein				1		1	16.559
		cgd4_2640	hypothetical protein				1		1	18.862
		cgd4_2650	hypothetical protein			1			1	3.247
		cgd4_2660	similarity at COOH terminus with programmed cell death protein 2		1				1	1.650
		cgd4_2690	sec7 domain containing protein, possibly of plant origin	1					1	0.405
		cgd4_2730	predicted coiled-coil protein	1					1	0.518
		cgd4_2760	hypothetical protein	1					1	0.371
		cgd4_2800	hypothetical protein having a signal peptide		1				1	1.356
		cgd4_2810	hypothetical protein, signal peptide	1					1	0.327
		cgd4_2880	hypothetical protein				1		1	6.438
		cgd4_2920	hypothetical protein	1					1	0.401
		cgd4_2950	hypothetical protein			1			1	3.814
		cgd4_2980	RhoGAP domain containing protein with a Sec14D domain at the N-terminus					1	1	33.744
		cgd4_30	hypothetical protein		1				1	1.625
		cgd4_3010	hypothetical protein				1		1	16.478
		cgd4_3020	hypothetical protein with a signal peptide			1			1	3.341
		cgd4_3030	hypothetical protein with a signal peptide				1		1	9.488
		cgd4_3050	hypothetical protein					1	1	232.621
		cgd4_3060	hypothetical protein	1					1	0.258
		cgd4_3070	hypothetical protein			1			1	2.330
		cgd4_31	hypothetical protein	1					1	0.498
		cgd4_3140	WD repeat protein		1				1	1.628
		cgd4_32	hypothetical protein		1				1	2.021
		cgd4_3200	hypothetical protein				1		1	16.066
		cgd4_3210	hypothetical protein			1			1	2.913
		cgd4_3230	hypothetical protein with a signal peptide				1		1	27.260
		cgd4_3240	possible NLI interacting factor CTD-like phosphatase	1					1	0.363
		cgd4_3280	hypothetical protein					1	1	52.507
		cgd4_330	hypothetical protein				1		1	23.099
		cgd4_3330	hypothetical protein					1	1	64.007
		cgd4_3350	hypothetical protein	1					1	0.449

		cgd4_3360	WD repeat protein					1	1	67.441
		cgd4_3370	hypothetical protein			1			1	5.206
		cgd4_3390	neutral sphingomyelinase activation associated factor-like BEACH domain containing protein			1			1	2.456
		cgd4_340	protein with possible conserved beta tubulin folding factor domain	1					1	0.397
		cgd4_3430	hypothetical protein with signal peptide and central coiled-coil region, within large locus of signal peptide containing proteins				1		1	13.655
		cgd4_3500	hypothetical protein	1					1	0.290
		cgd4_3510	hypothetical protein	1					1	0.390
		cgd4_3530	large hypothetical protein with signal peptide, within large locus of signal peptide containing proteins		1				1	1.767
		cgd4_3560	hypothetical protein					1	1	30.768
		cgd4_3600	hypothetical protein with possible signal peptide, 'HCD family'paralog					1	1	42.823
		cgd4_3610	hypothetical protein with signal peptide				1		1	10.442
		cgd4_3620	immunodominant antigen 23393226					1	1	787.563
		cgd4_3640	hypothetical protein					1	1	112.981
		cgd4_3660	hypothetical protein	1					1	0.372
		cgd4_3670	uncharacterized low complexity protein				1		1	9.023
		cgd4_3680	hypothetical protein				1		1	11.611
		cgd4_3690	large glycine-rich repeat low complexity protein					1	1	28.345
		cgd4_3720	possible 2 TPR domains at N-terminus	1					1	0.472
		cgd4_3730	hypothetical protein		1				1	1.856
		cgd4_3750	hypothetical protein with signal peptide				1		1	6.132
		cgd4_3760	hypothetical protein		1				1	1.417
		cgd4_3780	hypothetical protein	1			1		2	3.769
		cgd4_3820	hypothetical protein	1					1	0.422
		cgd4_3830	Ank repeat protein with possible signal peptide	1					1	0.382
		cgd4_3840	Ank, ankyrin repeats containing protein with 9 transmembrane domains at C-terminus	1					1	0.338
		cgd4_3870	large protein with signal peptide	1					1	0.419
		cgd4_3890	hypothetical protein	1					1	0.299
		cgd4_3900	hypothetical protein	1					1	0.309
		cgd4_3910	hypothetical protein					1	1	44.273
		cgd4_3960	hypothetical protein	1					1	0.426
		cgd4_3980	hypothetical protein			1			1	2.863
		cgd4_400	protein with possible 2 TPR domains	1					1	0.395
		cgd4_4000	possible ribosomal-protein-alanine acetyltransferase	1					1	0.448
		cgd4_4030	hypothetical protein		1				1	2.267
		cgd4_4060	B-box zinc finger domain containing protein	1					1	0.560
		cgd4_4070	hypothetical protein	1					1	0.367
		cgd4_410	protein with 4xEZ_heat domains					1	1	51.034
		cgd4_4100	hypothetical protein	1					1	0.309
		cgd4_4130	hypothetical protein	1					1	0.331
		cgd4_4150	hypothetical protein	1					1	0.327
		cgd4_4170	hypothetical protein	1					1	0.432
		cgd4_4180	hypothetical protein	1					1	0.297
		cgd4_4190	hypothetical protein with signal peptide	1					1	0.389
		cgd4_4220	hypothetical protein	1					1	0.279
		cgd4_4250	hypothetical protein		1				1	1.650
		cgd4_4270	hypothetical protein			1			1	2.633
		cgd4_4310	ring finger domain containing protein					1	1	481.612
		cgd4_4330	hypothetical protein		1				1	1.509
		cgd4_4350	hypothetical protein	1	1				2	1.009
		cgd4_4360	ring finger domain containing protein	1					1	0.313
		cgd4_4370	hypothetical protein	1					1	0.287
		cgd4_4380	hypothetical protein, signal peptide	1					1	0.305
		cgd4_4400	hypothetical protein					1	1	73.638
		cgd4_4410	2x PHD domain containing protein		1				1	2.093
		cgd4_4420	hypothetical protein			1			1	5.465
		cgd4_4440	hypothetical protein			1			1	2.500
		cgd4_4450	possible transporter with 11 transmembrane domains	1					1	0.337
		cgd4_450	very large low complexity protein	1					1	0.494
		cgd4_4500	signal peptide containing cysteine rich protein, gene anchored to telomere.				1		1	15.850
		cgd4_460	hypothetical protein			1			1	2.496
		cgd4_480	hypothetical protein	1					1	0.473
		cgd4_490	hypothetical protein				1		1	20.780
		cgd4_50	hypothetical protein	1					1	0.453
		cgd4_510	hypothetical protein		1				1	1.599
		cgd4_520	hypothetical protein	1					1	0.497
		cgd4_550	hypothetical protein	1					1	0.343
		cgd4_560	hypothetical protein	1					1	0.397
		cgd4_600	hypothetical protein	1					1	0.525
		cgd4_610	MJ050-like PP-loop ATpase	1					1	0.379
		cgd4_680	hypothetical protein		1				1	1.222
		cgd4_730	hypothetical protein	1					1	0.367

		cgd4_760	possible ASF1 anti-silencing function 1 like protein	1					1	0.446
		cgd4_770	Low complexity protein with large Glu repeat	1					1	0.294
		cgd4_820	WD repeat protein	1					1	0.470
		cgd4_850	large protein with signal peptide	1					1	0.441
		cgd4_860	possible WWE domain				1		1	10.894
		cgd4_90	hypothetical protein					1	1	38.676
		cgd4_900	WD repeat containing protein				1		1	9.413
		cgd4_940	hypothetical protein				1		1	12.471
		cgd4_950	WD repeat protein	1					1	0.342
		cgd5_10	signal peptide containing protein					1	1	596.842
		cgd5_1010	hypothetical protein			1			1	4.613
		cgd5_1040	arfgap'arfgap like finger domain containing protein'	1					1	0.611
		cgd5_1050	hypothetical protein	1					1	0.372
		cgd5_1060	hypothetical protein	1	1				2	0.957
		cgd5_1080	hypothetical protein				1		1	7.228
		cgd5_1110	C2H2 finger domain containing protein	1					1	0.490
		cgd5_1120	DNMAP1 like Myb domain	1					1	0.566
		cgd5_1130	WD-40 repeat protein family / small nuclear ribonucleoprotein Prp4p-related	1					1	0.428
		cgd5_1150	iwsip-like protein		1				1	2.024
		cgd5_1160	POZ domain protein that is fused to a MATH domain at its N-terminus	1					1	0.403
		cgd5_1200	ring finger and bbox containing protein, putative				1		1	23.380
		cgd5_1220	hypothetical protein	1	1				2	0.881
		cgd5_1230	hypothetical protein		1				1	1.296
		cgd5_1370	hypothetical protein			1			1	5.073
		cgd5_140	ARF GTPase activating protein, putative	1					1	0.574
		cgd5_1400	hypothetical protein	1					1	0.378
		cgd5_1420	putative secreted protein		1				1	1.446
		cgd5_1440	large protein with signal peptide	1					1	0.443
		cgd5_1450	hypothetical protein with signal peptide			1			1	3.570
		cgd5_1460	hypothetical protein with signal peptide		1				1	1.719
		cgd5_1480	cysteine rich protein having a signal peptide			1			1	2.252
		cgd5_1500	hypothetical protein	1					1	0.466
		cgd5_1530	hypothetical protein having a signal peptide	1					1	0.563
		cgd5_1580	hypothetical protein					1	1	177.577
		cgd5_1590	hypothetical protein		1				1	1.878
		cgd5_160	hypothetical protein					1	1	28.240
		cgd5_1610	hypothetical protein, possibly apicomplexan conserved			1			1	4.965
		cgd5_1620	hypothetical protein with short conserved N-terminal motif, possible cysteine binding domain				1		1	22.852
		cgd5_1640	MORN repeat protein			1			1	3.186
		cgd5_1650	hypothetical protein			1			1	4.511
		cgd5_1660	hypothetical protein with signal peptide and acidic stretch		1				1	1.767
		cgd5_1670	hypothetical protein with conserved apicomplexan domain			1			1	2.710
		cgd5_1680	protein with an N-terminal domain shared with the TOG/ALp4p ike microtubule associated proteins	1					1	0.568
		cgd5_170	hypothetical protein			1			1	3.481
		cgd5_1780	MJ1625/yease Yp1009cp-like HhH domain					1	1	85.961
		cgd5_180	hypothetical protein with signal peptide				1		1	7.055
		cgd5_1800	hypothetical protein	1					1	0.512
		cgd5_1810	SMART 2xt_SNARE domain containing protein	1					1	0.543
		cgd5_1840	Vir superfamily protein					1	1	30.925
		cgd5_1870	LETM1/MRS7 family protein with a transmembrane region at the N-terminus	1					1	0.275
		cgd5_1890	hypothetical protein			1			1	5.989
		cgd5_1930	hypothetical protein	1					1	0.479
		cgd5_1970	AtPH1 like protein with a pleckstrin homology (PH) domain			1			1	3.580
		cgd5_1980	hypothetical protein			1			1	6.009
		cgd5_1990	hypothetical protein			1			1	2.889
		cgd5_20	signal peptide containing protein			1			1	2.466
		cgd5_200	hypothetical protein	1					1	0.312
		cgd5_2000	protein with RIO domain within N-terminal region					1	1	46.919
		cgd5_2050	protein with forkhead associated (FHA) domain within N-terminal region and possible central coiled coil domain	1	1				2	1.409
		cgd5_2080	protein with PHD finger and a nipped-B/Mis4/Scc2 like domain at the C-terminus		1				1	1.904
		cgd5_2090	FCH domain containing protein	1					1	0.335
		cgd5_210	hypothetical protein	1					1	0.355
		cgd5_2120	hypothetical protein		1				1	1.611
		cgd5_2140	hypothetical protein					1	1	61.975
		cgd5_2170	hypothetical protein			1			1	2.401
		cgd5_2180	large low complexity protein with proline/alanine-rich repeat					1	1	61.504
		cgd5_220	hypothetical protein	1					1	0.347
		cgd5_2200	T8M16_190-plant like protein with RRM domain				1		1	8.952
		cgd5_2250	hypothetical protein				1		1	5.962
		cgd5_2260	Low complexity hypothetical protein			1			1	5.479
		cgd5_2310	Sin3 like paired amphipathic helix containing protein					1	1	24.474

		cgd5_2340	possible SET domain containing protein			1			1	5.046
		cgd5_240	G patch domain containing protein			1			1	3.844
		cgd5_2540	WD repeat protein		1				1	1.486
		cgd5_2560	MutL family ATpase	1					1	0.369
		cgd5_2570	hypothetical protein			1			1	5.281
		cgd5_260	hypothetical protein	1					1	0.451
		cgd5_2600	putative leucine aminopeptidase; of possible plant or bacterial origin		1				1	1.390
		cgd5_2620	penguin protein containing pumolio repeats				1		1	19.644
		cgd5_2640	little finger (CxxC...CxxC) domain containing protein		1				1	1.827
		cgd5_2650	hypothetical protein	1					1	0.423
		cgd5_2660	hypothetical protein	1					1	0.408
		cgd5_2700	hypothetical protein with a signal peptide				1		1	19.761
		cgd5_2720	large protein containing a signal peptide					1	1	266.329
		cgd5_2730	hypothetical protein			1			1	4.324
		cgd5_2750	hypothetical protein, signal peptide			1			1	2.431
		cgd5_2760	hypothetical protein					1	1	62.000
		cgd5_2770	ARF like small GTpase	1					1	0.381
		cgd5_2850	hypothetical protein	1					1	0.358
		cgd5_2860	hypothetical protein			1			1	5.630
		cgd5_2890	hypothetical protein		1				1	2.025
		cgd5_2920	hypothetical protein	1					1	0.522
		cgd5_2930	hypothetical protein					1	1	41.689
		cgd5_2970	hypothetical protein			1			1	4.980
		cgd5_2980	hypothetical protein				1		1	15.738
		cgd5_30	hypothetical protein					1	1	161.352
		cgd5_300	hypothetical protein	1					1	0.444
		cgd5_3010	hypothetical protein with possible plus3 domain		1				1	2.234
		cgd5_3020	hypothetical protein	1					1	0.389
		cgd5_3060	hypothetical protein	1					1	0.354
		cgd5_3100	hypothetical protein	1					1	0.440
		cgd5_3120	hypothetical protein		1				1	1.506
		cgd5_3190	hypothetical protein	1					1	0.307
		cgd5_3210	large hypothetical protein with signal peptide	1					1	0.356
		cgd5_330	hypothetical protein	1					1	0.345
		cgd5_3300	large protein with 4 PHD domains				1		1	23.238
		cgd5_3310	WD repeat protein					1	1	67.521
		cgd5_3320	hypothetical protein			1			1	3.140
		cgd5_3350	hypothetical protein				1		1	6.550
		cgd5_3370	hypothetical protein		1				1	1.711
		cgd5_3380	protein with signal peptide and possible kazal domain	1	1				2	1.160
		cgd5_3390	very large hypothetical protein	1					1	0.395
		cgd5_3440	hypothetical protein	1					1	0.336
		cgd5_3470	E. coli yfiP family protein			1			1	3.325
		cgd5_3480	hypothetical protein with possible conserved HGG motif					1	1	105.595
		cgd5_3490	hypothetical protein with signal peptide					1	1	125.868
		cgd5_350	hypothetical protein			1			1	2.522
		cgd5_3570	putative secreted protein	1					1	0.366
		cgd5_3600	predicted secreted protein	1					1	0.387
		cgd5_3620	hypothetical protein	1					1	0.502
		cgd5_3640	hypothetical protein		1				1	1.828
		cgd5_3650	hypothetical protein					1	1	191.631
		cgd5_3670	hypothetical protein	1					1	0.430
		cgd5_3680	WD repeat containing protein	1					1	0.309
		cgd5_370	predicted coiled coil protein				1		1	6.495
		cgd5_3700	Sec1 family	1					1	0.354
		cgd5_3760	hypothetical protein				1		1	12.585
		cgd5_3780	hypothetical protein with a possible cysteine-rich domain (BBOX zinc finger?)				1		1	8.113
		cgd5_3800	hypothetical protein	1					1	0.304
		cgd5_3830	hypothetical protein		1				1	1.414
		cgd5_3860	hypothetical protein	1					1	0.284
		cgd5_3880	hypothetical protein	1					1	0.523
		cgd5_3900	protein with RING finger domain at C-terminus	1					1	0.253
		cgd5_3910	hypothetical protein	1					1	0.346
		cgd5_3930	hypothetical protein		1				1	1.954
		cgd5_40	signal peptide containing large protein with proline stretches					1	1	100.741
		cgd5_400	protein with 4 PHD domains plus a SET domain and associated cysteine cluster at the C-terminus	1					1	0.309
		cgd5_4000	hypothetical protein	1					1	0.290
		cgd5_4010	hypothetical protein	1					1	0.399
		cgd5_4020	hypothetical protein	1					1	0.427
		cgd5_4030	hypothetical protein	1					1	0.316
		cgd5_4040	hypothetical protein	1					1	0.370
		cgd5_4070	hypothetical protein	1					1	0.400
		cgd5_4100	hypothetical protein conserved in apicomplexa		1				1	1.419
		cgd5_4110	possible EF hand protein		1				1	1.692

		cgd5_4120	hypothetical protein with a signal peptide	1					1	0.310
		cgd5_4130	hypothetical protein having a signal peptide and transmembrane domain near C-terminus	1					1	0.420
		cgd5_4240	hypothetical protein	1					1	0.417
		cgd5_4260	WD40 repeat protein					1	1	41.796
		cgd5_4340	hypothetical protein	1					1	0.614
		cgd5_4350	hypothetical protein					1	1	25.399
		cgd5_4360	large protein with possible RING domain			1			1	3.380
		cgd5_440	protein with 2 CAP (CARP) domains, possible adenyl cyclase-associated protein				1		1	6.338
		cgd5_4400	RIKEN cDNA 2610011N19 gen		1				1	1.970
		cgd5_4410	hypothetical protein					1	1	24.620
		cgd5_4430	WD40 repeat protein				1		1	6.831
		cgd5_4460	hypothetical protein	1					1	0.357
		cgd5_4480	large low complexity protein, possible apicomplexan-specific	1					1	0.391
		cgd5_4490	hypothetical protein	1					1	0.383
		cgd5_450	possible ring domain protein				1		1	20.869
		cgd5_4500	possible vacuolar protein sorting associated protein (VPS)	1					1	0.318
		cgd5_4510	hypothetical protein	1					1	0.473
		cgd5_4570	hypothetical protein	1	1				2	1.037
		cgd5_470	hypothetical protein	1					1	0.303
		cgd5_540	hypothetical protein	1	1				2	0.997
		cgd5_550	hypothetical protein	1					1	0.399
		cgd5_630	protein with 4 ankyrin repeats plus a bromodomain.					1	1	30.961
		cgd5_740	WD repeat protein	1					1	0.495
		cgd5_760	hypothetical protein	1					1	0.365
		cgd5_780	hypothetical protein	1					1	0.393
		cgd5_80	hypothetical protein	1					1	0.401
		cgd5_800	hypothetical protein				1		1	25.323
		cgd5_810	leucine-rich repeat protein		1				1	1.859
		cgd5_840	PP2C like phosphatase	1					1	0.589
		cgd5_860	hypothetical protein	1					1	0.377
		cgd5_870	hypothetical protein				1		1	8.797
		cgd5_880	VirR/VirH family protein		1				1	1.394
		cgd5_890	hypothetical protein	1					1	0.374
		cgd5_90	hypothetical protein		1				1	2.157
		cgd5_910	protein with conserved domain that is fused in vertebrates to neuralized domain repeats	1					1	0.523
		cgd5_960	Oxr1p like TLDc domain containing protein			1			1	3.565
		cgd5_990	hypothetical protein		1				1	1.669
		cgd6_10	hypothetical protein with signal peptide and cysteine rich region at the C-terminus					1	1	525.219
		cgd6_100	leucine rich repeat (LRR) protein					1	1	52.251
		cgd6_1000	hypothetical protein with signal peptide			1			1	3.401
		cgd6_1030	hypothetical protein with signal peptide		1				1	1.861
		cgd6_1060	protein with spectrin repeats, CG12008-like	1					1	0.473
		cgd6_1110	signal peptide-containing protein	1					1	0.336
		cgd6_1130	hypothetical protein with signal peptide	1					1	0.369
		cgd6_1140	hypothetical protein		1				1	1.945
		cgd6_1150	hypothetical protein				1		1	9.157
		cgd6_1160	hypothetical protein	1					1	0.289
		cgd6_1180	large protein with signal peptide	1					1	0.426
		cgd6_1210	hypothetical protein			1			1	3.288
		cgd6_1220	hypothetical protein	1					1	0.313
		cgd6_1240	hypothetical protein	1					1	0.442
		cgd6_1250	hypothetical protein	1					1	0.322
		cgd6_1260	hypothetical protein	1					1	0.345
		cgd6_1280	hypothetical protein with one or more transmembrane domain	1					1	0.431
		cgd6_1290	ring finger, membrane associated, possible signal peptide	1					1	0.326
		cgd6_1330	3CCCH domain containing protein	1					1	0.543
		cgd6_1400	hypothetical protein	1					1	0.447
		cgd6_1460	Sec14 domain containing protein		1				1	1.626
		cgd6_1470	protein with SET domain flanked by cysteine clusters plus a C-terminal PHD domain				1		1	6.961
		cgd6_1500	cytoachrome c oxidase subunit III with 6 transmembrane domains (Note: unknown)			1			1	3.545
		cgd6_1540	possible histone mRNA hairpin-binding protein	1					1	0.379
		cgd6_1560	Yju2p / cwf16-like; Zn finger		1				1	1.504
		cgd6_1600	hypothetical protein	1					1	0.345
		cgd6_1610	hypothetical protein			1			1	3.332
		cgd6_1670	MJ1332/Ygr210cp-like GTP binding protein; GTpase OBG family plus RNA binding domain TGS	1					1	0.326
		cgd6_1700	RRM domain containing protein			1			1	2.541
		cgd6_1710	Mcm10p-like'Mcm10p-like'	1					1	0.508
		cgd6_1740	Tra1p-like; C-terminal FAT domain plus phoshoinositide 3-kinase domain; very large protein		1				1	2.182
		cgd6_1770	protein with C2H2 Zn finger, ASR2B-like N- and C-terminus	1					1	0.417
		cgd6_1790	hypothetical protein	1					1	0.410

		cgd6_180	hypothetical protein	1					1	0.533
		cgd6_1810	hypothetical protein	1					1	0.431
		cgd6_1870	signal recognition particle SPR19			1			1	2.326
		cgd6_1880	CG9286/Bcp1p/BRCA and CDKN1A-interacting protein isoform C-like					1	1	28.430
		cgd6_1890	hypothetical predicted protein, unknown function				1		1	10.542
		cgd6_190	hypothetical protein	1					1	0.418
		cgd6_1930	uncharacterized coiled-coil protein	1					1	0.366
		cgd6_1940	hypothetical protein	1					1	0.340
		cgd6_1980	hypothetical protein with a signal peptide		1				1	1.510
		cgd6_2160	hypothetical protein		1				1	1.352
		cgd6_2190	TBC domain containing protein	1					1	0.384
		cgd6_2200	leucine-rich repeats protein		1				1	2.148
		cgd6_2240	protein with N-terminal apicomplexan-specific globular domain plus PHD domain		1				1	1.555
		cgd6_2250	myb proto-oncogene protein, putative	1					1	0.418
		cgd6_2270	hypothetical membrane-associated protein with 12 transmembrane domains				1		1	10.387
		cgd6_2320	multi-domain chromatin protein with WD40 repeats, bromodomain and PHD domain	1					1	0.331
		cgd6_2330	hypothetical protein with signal peptide				1		1	19.882
		cgd6_2370	Sec1-family protein	1					1	0.298
		cgd6_2400	hypothetical protein	1					1	0.502
		cgd6_2410	possible CutA1 divalent ion tolerance protein	1					1	0.376
		cgd6_250	hypothetical protein	1					1	0.349
		cgd6_2500	hypothetical protein with possible signal peptide			1			1	3.413
		cgd6_2530	hypothetical protein				1		1	12.706
		cgd6_2570	MORN domain repeat containing protein		1				1	2.053
		cgd6_2580	hypothetical protein of low complexity		1				1	1.795
		cgd6_2600	hypothetical protein					1	1	337.215
		cgd6_2630	hypothetical protein	1					1	0.520
		cgd6_2670	hypothetical protein	1					1	0.282
		cgd6_2680	hypothetical protein	1					1	0.411
		cgd6_270	hypothetical protein		1				1	1.398
		cgd6_2700	Low complexity protein, possible apicomplexan conserved		1				1	1.848
		cgd6_2710	hypothetical protein		1				1	2.136
		cgd6_2720	hypothetical protein having a signal peptide and transmembrane domain within C- terminus					1	1	124.461
		cgd6_2760	hypothetical protein					1	1	40.847
		cgd6_280	hypothetical protein	1					1	0.350
		cgd6_2800	t24f1.1 protein, putative	1					1	0.397
		cgd6_2810	hypothetical protein		1				1	2.239
		cgd6_2850	hypothetical protein	1					1	0.431
		cgd6_2860	uncharacterized coiled coil protein	1					1	0.556
		cgd6_2870	hypothetical protein		1				1	1.418
		cgd6_2880	hypothetical protein		1				1	1.791
		cgd6_2890	hypothetical protein	1					1	0.419
		cgd6_290	hypothetical protein	1					1	0.281
		cgd6_2910	hypothetical protein with signal peptide	1					1	0.378
		cgd6_2920	hypothetical protein with signal peptide	1					1	0.390
		cgd6_2950	large low complexity with PFL0360c_pfal like Znfinger		1				1	1.291
		cgd6_2960	Bax inhibitor-1 (BI-1). integral membrane protien with 6 or more transmembrane domains					1	1	31.965
		cgd6_2980	hypothetical protein with conserved cysteines as in riboflavin-binding protein precursor (RBP), contains signal peptide plus possible GPI anchor				1		1	5.870
		cgd6_2990	hypothetical protein	1					1	0.426
		cgd6_300	hypothetical protein	1					1	0.505
		cgd6_3030	protein with C-terminal RCC1 domain	1					1	0.397
		cgd6_3040	hypothetical protein	1					1	0.459
		cgd6_3050	hypothetical protein with a signal peptide		1				1	1.768
		cgd6_3060	hypothetical protein	1					1	0.218
		cgd6_3070	hypothetical protein with a signal peptide				1		1	7.640
		cgd6_3080	hypothetical protein with a signal peptide			1			1	2.799
		cgd6_3090	hypothetical protein	1					1	0.266
		cgd6_310	hypothetical protein	1					1	0.346
		cgd6_3110	hypothetical protein	1					1	0.412
		cgd6_3120	hypothetical protein	1					1	0.270
		cgd6_3130	hypothetical protein					1	1	50.680
		cgd6_3150	hypothetical protein	1					1	0.342
		cgd6_3160	large low complexity protein		1				1	2.261
		cgd6_3170	Sel1 protein, putative	1					1	0.538
		cgd6_3200	hypothetical protein	1					1	0.497
		cgd6_3250	hypothetical protein	1					1	0.382
		cgd6_330	hypothetical protein				1		1	11.034
		cgd6_3300	Yir323cp/Cwc24 p family; CCCH+ringfinger domains	1					1	0.365
		cgd6_3310	hypothetical protein with signal peptide	1					1	0.335
		cgd6_3320	hypothetical protein	1					1	0.257

		cgd6_3430	RRM (2xRRMs) domaincontaining protein		1				1	1.448
		cgd6_3440	hypothetical protein				1		1	13.023
		cgd6_3450	EBNA1 binding protein 2; nucleolar protein p40					1	1	138.327
		cgd6_3460	hypothetical protein			1			1	3.152
		cgd6_3470	hypothetical protein					1	1	30.420
		cgd6_3480	hypothetical protein					1	1	46.390
		cgd6_3490	RNA-binding protein, putative				1		1	9.166
		cgd6_350	hypothetical protein	1					1	0.342
		cgd6_3500	hypothetical protein			1			1	6.298
		cgd6_3510	hypothetical protein			1			1	4.376
		cgd6_3520	hypothetical protein				1		1	15.332
		cgd6_360	hypothetical protein	1					1	0.368
		cgd6_3610	hypothetical predicted WD-40 repeat protein, unknown function			1			1	5.595
		cgd6_3650	signal peptide, large secreted protein with N terminal ankyrin repeats + 8+ transmembrane domain	1					1	0.543
		cgd6_3660	hypothetical protein		1				1	1.346
		cgd6_3670	hypothetical protein	1					1	0.253
		cgd6_3680	Low complexity hypothetical protein	1					1	0.425
		cgd6_3740	41-2 protein antigen precursor			1			1	3.381
		cgd6_3760	hypothetical protein					1	1	113.466
		cgd6_3770	hypothetical protein	1					1	0.426
		cgd6_380	hypothetical protein with signal peptide and 4 transmembrane domains, possible apicomplexan conserved	1					1	0.289
		cgd6_3810	NUDIX domain protein; mRNA cleavage factor-like protein Im like, plant+animal group			1			1	4.787
		cgd6_3840	SET domain containing protein			1			1	3.366
		cgd6_3890	hypothetical protein	1					1	0.598
		cgd6_390	hypothetical protein	1					1	0.511
		cgd6_3900	hypothetical protein			1			1	2.364
		cgd6_3920	hypothetical protein with signal peptide					1	1	1,276.224
		cgd6_3930	hypothetical protein		1				1	2.404
		cgd6_3940	hypothetical protein			1			1	3.502
		cgd6_3950	uncharacterized protein	1					1	0.358
		cgd6_3980	hypothetical protein				1		1	16.534
		cgd6_4000	hypothetical protein having a signal peptide		1				1	1.864
		cgd6_4010	hypothetical protein	1					1	0.434
		cgd6_4050	uncharacterized apicomplexan-specific protein	1					1	0.419
		cgd6_4070	hypothetical protein			1			1	2.339
		cgd6_4090	hypothetical low complexity protein	1					1	0.333
		cgd6_4110	hypothetical protein					1	1	55.956
		cgd6_4140	coiled coil protein	1					1	0.275
		cgd6_4160	EF-hands domain containing protein				1		1	19.666
		cgd6_4170	hypothetical protein				1		1	6.004
		cgd6_4230	hypothetical protein containing a signal peptide					1	1	27.021
		cgd6_4240	MO25 protein	1					1	0.296
		cgd6_4260	Low complexity protein			1			1	4.521
		cgd6_4280	hypothetical protein having a signal peptide					1	1	56.925
		cgd6_4290	large protein having a MATH plus possible UBC hydrolase plus type I pseurousyn plus hexapeptide			1			1	3.691
		cgd6_4300	uncharacterized coiled coil protein	1					1	0.505
		cgd6_4360	hypothetical protein having a signal peptide and 4 transmembrane domains		1				1	1.677
		cgd6_4390	hypothetical low complexity protein	1					1	0.529
		cgd6_4430	hypothetical protein	1					1	0.394
		cgd6_4460	large protein with ARM repeats				1		1	19.317
		cgd6_4470	hypothetical low complexity protein with signal peptide					1	1	53.627
		cgd6_450	hypothetical protein		1				1	1.370
		cgd6_4520	hypothetical protein	1					1	0.460
		cgd6_4590	ankyrin-related protein, putative				1		1	9.386
		cgd6_460	hypothetical protein	1	1				2	1.033
		cgd6_4640	hypothetical protein	1					1	0.347
		cgd6_4690	SAC3/GANP/Rpn12p family				1		1	8.863
		cgd6_470	hypothetical protein	1					1	0.284
		cgd6_4720	hypothetical protein				1		1	7.312
		cgd6_4790	predicted degraded Zn-finger +bbox Zn finger protein	1					1	0.372
		cgd6_480	hypothetical protein	1					1	0.392
		cgd6_4800	hypothetical protein	1					1	0.330
		cgd6_4810	hypothetical protein	1					1	0.338
		cgd6_4820	hypothetical protein	1					1	0.346
		cgd6_4870	hypothetical protein			1			1	3.241
		cgd6_4950	hypothetical protein					1	1	114.487
		cgd6_50	hypothetical protein	1					1	0.599
		cgd6_5010	hypothetical protein		1				1	1.590
		cgd6_5020	protein with WD40 repeats		1				1	1.837
		cgd6_5030	hypothetical protein				1		1	10.895
		cgd6_5050	hypothetical protein	1					1	0.368
		cgd6_5110	large hypothetical protein with possible signal peptide				1		1	11.007

		cgd6_5120	protein with two PHD Zn fingers that is probably involved in chromatin function			1			1	3.547
		cgd6_5150	ring finger protein	1					1	0.434
		cgd6_5160	hypothetical protein with conserved carboxy terminal region (KIAA1078 protein)	1					1	0.495
		cgd6_5170	hypothetical protein	1					1	0.412
		cgd6_5180	hypothetical protein having a transmembrane domain within N-terminus	1					1	0.444
		cgd6_5220	cysteine-rich protein with zinc finger	1					1	0.286
		cgd6_5230	hypothetical protein	1					1	0.294
		cgd6_5270	hypothetical protein with possible signal peptide		1				1	1.682
		cgd6_5280	hypothetical protein with signal peptide		1				1	1.537
		cgd6_530	Low complexity protein having signal peptide and glycine-rich repeat at C-terminal region			1			1	4.522
		cgd6_5300	hypothetical protein	1					1	0.319
		cgd6_5310	hypothetical protein with signal peptide		1				1	2.057
		cgd6_5360	hypothetical protein with signal peptide	1					1	0.361
		cgd6_5370	hypothetical protein	1					1	0.374
		cgd6_5390	hypothetical protein, signal peptide		1				1	1.771
		cgd6_5420	hypothetical protein, signal peptide		1				1	1.454
		cgd6_5440	hypothetical protein, signal peptide, paralogs				1		1	18.558
		cgd6_600	hypothetical protein	1					1	0.314
		cgd6_770	hypothetical protein				1		1	6.692
		cgd6_810	swift like BRCT domain	1					1	0.479
		cgd6_820	predicted secreted low complexity protein with a signal peptide		1				1	2.042
		cgd6_840	shanti/Ykl100cp/Minor histocompatibility antigen H13-like; presenilin, signal peptide peptidase family, with 10 transmembrane domains and a signal peptide	1					1	0.558
		cgd6_860	hypothetical protein		1				1	1.384
		cgd6_940	pescadillo containing BRCT domain					1	1	36.922
		cgd6_950	Pdr17p-like Sec14 domain containing protein		1				1	2.092
		cgd6_970	hypothetical protein	1					1	0.472
		cgd7_10	hypothetical protein					1	1	31.885
		cgd7_100	ring domain-containing protein				1		1	9.033
		cgd7_1000	F27M3_19 plant like RRM plus AlkB domain containing protein		1				1	1.991
		cgd7_1010	very large low complexity protein		1				1	1.997
		cgd7_1020	G10 protein			1			1	3.007
		cgd7_1030	hypothetical protein		1				1	1.512
		cgd7_1050	hypothetical protein				1		1	15.256
		cgd7_1090	hypothetical protein	1					1	0.292
		cgd7_1120	3CCCH domain containing protein				1		1	7.978
		cgd7_1170	protein containing ringfinger+4xC2H2+littlefinger domains					1	1	45.442
		cgd7_1210	hypothetical protein containing a signal peptide	1					1	0.457
		cgd7_1220	hypothetical protein containing a signal peptide				1		1	9.343
		cgd7_1230	hypothetical protein		1				1	1.962
		cgd7_1270	large hypothetical protein			1			1	2.567
		cgd7_1280	hypothetical protein			1			1	4.180
		cgd7_1290	hypothetical protein containing a signal peptide			1			1	2.501
		cgd7_1300	hypothetical protein containing a signal peptide	1					1	0.507
		cgd7_1360	protein with signal peptide and Sec14d domain, probably secreted				1		1	16.514
		cgd7_1370	hypothetical protein			1			1	4.150
		cgd7_1400	signal peptide containing protein		1				1	1.406
		cgd7_1420	hypothetical protein					1	1	83.928
		cgd7_1440	protein with 5 TPR domains			1			1	2.511
		cgd7_1450	hypothetical protein		1				1	2.045
		cgd7_1470	hypothetical protein		1				1	1.691
		cgd7_1480	hypothetical protein			1			1	2.881
		cgd7_1510	hypothetical protein		1				1	2.009
		cgd7_1550	uncharacterized protein			1			1	2.550
		cgd7_1570	hypothetical protein			1			1	3.179
		cgd7_1580	mbl domain containing protein	1					1	0.577
		cgd7_1610	uncharacterized low complexity protein	1					1	0.374
		cgd7_170	hypothetical protein				1		1	9.088
		cgd7_1740	Ing1 like PHD finger protein				1		1	6.957
		cgd7_1770	hypothetical protein	1					1	0.496
		cgd7_1790	protein with BRIGHT domain like HTH domain at N-terminus		1				1	1.774
		cgd7_180	hypothetical protein		1				1	1.895
		cgd7_1850	SMC4'SMC4, chromosomal ATpase with giant coiled coil regions'	1					1	0.395
		cgd7_1870	hypothetical protein	1					1	0.383
		cgd7_1890	KH domain protein				1		1	13.547
		cgd7_190	hypothetical protein	1					1	0.313
		cgd7_1920	hypothetical protein	1					1	0.491
		cgd7_1990	hypothetical protein			1			1	4.679
		cgd7_200	hypothetical protein with signal peptide					1	1	30.975
		cgd7_2010	hypothetical membrane protein	1					1	0.400
		cgd7_2020	hypothetical protein	1					1	0.321

		cgd7_2050	Hch1p like mystery proteins.		1				1	2.053
		cgd7_2070	large membrane associated channel		1				1	1.309
		cgd7_2120	hypothetical protein	1					1	0.370
		cgd7_2130	uncharacterized protein	1					1	0.446
		cgd7_2180	hypothetical protein		1				1	1.589
		cgd7_2200	hypothetical protein, signal peptide				1		1	7.292
		cgd7_2220	hypothetical protein			1			1	4.994
		cgd7_2230	coiled coil protein		1				1	1.648
		cgd7_2240	hypothetical protein, signal peptide, possible secreted protein					1	1	32.330
		cgd7_2270	hypothetical low complexity protein				1		1	22.495
		cgd7_2290	hypothetical protein					1	1	49.785
		cgd7_2340	large secreted protein, signal peptide	1					1	0.387
		cgd7_2350	hypothetical small protein, possible signal peptide	1					1	0.487
		cgd7_2360	eukaryotic membrane protein with transmembrane domains near C- and N- regions	1					1	0.457
		cgd7_240	hypothetical protein			1			1	4.059
		cgd7_2410	signal peptide, secreted low complexity protein					1	1	26.511
		cgd7_2440	giant membrane protein		1				1	1.992
		cgd7_2500	Skp1 family protein, putative			1			1	3.881
		cgd7_2550	hypothetical protein				1		1	5.948
		cgd7_2600	Low complexity protein, putative			1			1	4.134
		cgd7_2610	POZ+kelch domain protein with kelch repeats at the C-terminus	1					1	0.473
		cgd7_2640	TPR repeat protein					1	1	56.445
		cgd7_2700	SMC3'SMC type chromosomal ABC ATpase'	1					1	0.555
		cgd7_2710	ataxin2 related nudix domain protein (RNA processing phosphatase/ATpase domain)	1					1	0.515
		cgd7_2720	FALZ protein	1					1	0.482
		cgd7_2740	WD40 protein	1					1	0.394
		cgd7_2770	uncharacterized low complexity protein	1					1	0.617
		cgd7_2790	regena domain protein (CCR-Not complex protein subunit 3), putative		1				1	1.288
		cgd7_2830	hypothetical low complexity protein		1				1	2.091
		cgd7_2860	uncharacterized protein with predicted coiled coil regions	1					1	0.427
		cgd7_2870	uncharacterized low complexity protein			1			1	2.529
		cgd7_2880	hypothetical protein	1					1	0.414
		cgd7_2890	SLBP family of RNA binding proteins	1					1	0.421
		cgd7_2930	hypothetical protein	1					1	0.510
		cgd7_2960	hypothetical protein	1					1	0.454
		cgd7_2980	Low complexity protein witha potential C2C2 zinc ribbon	1					1	0.302
		cgd7_2990	hypothetical protein			1			1	2.247
		cgd7_30	large uncharacterized protein				1		1	15.229
		cgd7_3000	hypothetical protein					1	1	37.047
		cgd7_3010	cysteine-rich protein					1	1	419.549
		cgd7_3080	hypothetical protein	1					1	0.296
		cgd7_3090	uncharacterized low complexity protein	1					1	0.476
		cgd7_3150	eukaryote specific membrane proteins signal peptide plus 8 transmembrane domain	1					1	0.342
		cgd7_3190	protein with 3 CCCH domains					1	1	35.565
		cgd7_3210	hypothetical protein			1			1	3.225
		cgd7_3230	SLBP family of RNA binding proteins	1					1	0.572
		cgd7_3260	SWIB domain protein	1					1	0.512
		cgd7_3280	JAB domain containing protein		1				1	1.760
		cgd7_3310	hypothetical protein			1			1	3.165
		cgd7_3320	ring finger protein					1	1	61.043
		cgd7_3340	hypothetical protein	1					1	0.457
		cgd7_3420	hypothetical protein					1	1	33.212
		cgd7_3440	hypothetical protein			1			1	3.585
		cgd7_3460	hypothetical protein	1					1	0.334
		cgd7_3470	large low comlexity protein, potential zinc ribbon	1					1	0.430
		cgd7_3500	hypothetical protein				1		1	6.395
		cgd7_3520	hypothetical protein		1				1	1.769
		cgd7_3570	hypothetical protein	1					1	0.473
		cgd7_3580	kelch repeats protein			1			1	3.080
		cgd7_3640	YjeF family of predicted nucleotide binding proteins			1			1	4.188
		cgd7_3680	TPR repeat protein	1					1	0.317
		cgd7_3690	crooked neck protein HAT repeats	1					1	0.470
		cgd7_370	protein with a conserved N-terminal region				1		1	5.916
		cgd7_3700	hypothetical protein	1					1	0.321
		cgd7_3710	LRR protein	1					1	0.342
		cgd7_3770	hypothetical protein		1				1	1.481
		cgd7_380	hypothetical protein		1				1	2.011
		cgd7_3850	hypothetical protein		1				1	1.400
		cgd7_3900	hypothetical protein	1					1	0.339
		cgd7_3920	hypothetical protein		1				1	1.747
		cgd7_3930	hypothetical protein	1					1	0.535
		cgd7_3980	PX and WD40 domain protein	1					1	0.531
		cgd7_3990	hypothetical protein					1	1	38.793

		cgd7_400	hypothetical protein, signal peptide		1				1	1.895
		cgd7_4000	hypothetical coiled coil protein				1		1	18.186
		cgd7_4030	hypothetical low complexity protein			1			1	4.739
		cgd7_4040	hypothetical protein				1		1	13.507
		cgd7_4060	signal recognition particle SPR72				1		1	13.736
		cgd7_410	hypothetical protein		1				1	1.857
		cgd7_4100	Ssm4 ring finger fused to a forkhead associated (FHA) domain (apicomplexan specific architecture)			1			1	3.457
		cgd7_4140	WD40 domain protein		1				1	2.068
		cgd7_4170	ring domain protein, possible 4 transmembrane domains		1				1	1.451
		cgd7_4180	hypothetical protein	1					1	0.520
		cgd7_420	protein with DEXDc plus ring plus HELICc; possible SNF2 domain			1			1	5.453
		cgd7_4200	hypothetical protein	1					1	0.404
		cgd7_4230	uncharacterized protein		1				1	1.848
		cgd7_4280	signal peptide, large secreted protein				1		1	7.912
		cgd7_4300	Low complexity protein with coiled coil regions					1	1	42.191
		cgd7_4320	signal peptide, secreted protein					1	1	32.552
		cgd7_4330	glycoprotein, putative			1			1	3.015
		cgd7_4340	very large secreted protein, signal peptide	1					1	0.420
		cgd7_4360	hypothetical protein					1	1	43.413
		cgd7_4400	coronin-type WD40 protein		1				1	1.401
		cgd7_4480	T8M16_190 RRM protein of possible plant origin				1		1	15.767
		cgd7_4490	signal peptide-containing protein with transmembrane domain	1					1	0.506
		cgd7_4500	sgnal peptide, large secreted protein	1					1	0.420
		cgd7_4560	gigantic extracellular protein with interesting sushi (9x) and archaeal protease type repeats having the domain architecture: signal peptide-CRYPB(3x)-sushi (9x)-archaeoglobus type repeats (2x)		1				1	1.499
		cgd7_4570	DID domain proteins (DID4/DID2 family)	1					1	0.443
		cgd7_4630	hypothetical protein					1	1	28.015
		cgd7_4650	hypothetical protein	1					1	0.342
		cgd7_4670	hypothetical protein	1					1	0.492
		cgd7_4700	ring finger domain protein and 2 transmembrane domains	1					1	0.370
		cgd7_4720	hypothetical protein			1			1	3.911
		cgd7_4750	hypothetical protein			1			1	4.721
		cgd7_4830	membrane proteins related to the mechanosensitive ion channel	1					1	0.403
		cgd7_4850	hypothetical protein		1				1	1.170
		cgd7_4860	uncharacterized protein				1		1	7.463
		cgd7_4870	hypothetical protein			1			1	3.173
		cgd7_4890	hypothetical protein	1					1	0.456
		cgd7_490	hypothetical protein					1	1	34.212
		cgd7_4960	Not4hp/Mot2p, RING finger+RRM domains				1		1	6.481
		cgd7_500	hypothetical protein					1	1	47.761
		cgd7_5030	uncharacterized low complexity protein	1					1	0.458
		cgd7_5040	uncharacterized low complexity protein	1					1	0.470
		cgd7_505	hypothetical protein		1				1	1.780
		cgd7_5090	SET domain protein with MYND insert (Skm/BOP family)			1			1	2.774
		cgd7_5110	hypothetical low complexity protein		1				1	1.447
		cgd7_5190	uncharacterized protein with coiled coil regions		1				1	2.104
		cgd7_5240	uncharacterized protein			1			1	3.565
		cgd7_5290	hedgehog-type HINT domain protein, possible animal horizontal transfer, signal peptide	1					1	0.442
		cgd7_5320	Cwf5-like with 2 ZnR domains (CCCH and RRM domains missing)					1	1	39.267
		cgd7_5360	uncharacterized protein				1		1	7.696
		cgd7_5370	LigT, 2 H domain protein, involved in RNA processing	1					1	0.483
		cgd7_5380	CtIP type zinc knuckle (C2H2)	1		1			2	2.062
		cgd7_540	hypothetical protein with signal peptide and 8 or more transmembrane domains	1					1	0.381
		cgd7_5410	WD40 protein			1			1	2.340
		cgd7_5420	uncharacterized protein	1					1	0.404
		cgd7_5440	very large low complexity protein, probably membrane associated	1					1	0.571
		cgd7_5470	uncharacterized protein				1		1	17.188
		cgd7_5500	large secreted protein	1					1	0.442
		cgd7_5520	serine/threonine rich low complexity protein				1		1	8.666
		cgd7_5530	hypothetical protein	1					1	0.326
		cgd7_560	hypothetical protein	1					1	0.387
		cgd7_570	protein with 2 TPR domains				1		1	7.043
		cgd7_600	hypothetical protein	1					1	0.297
		cgd7_620	Sec14d domain containing protein				1		1	7.669
		cgd7_680	hypothetical protein with a signal peptide			1			1	2.365
		cgd7_710	possible helicase	1					1	0.400
		cgd7_760	bruno-like protein with 2 RRM domains				1		1	8.938
		cgd7_770	hypothetical protein	1					1	0.426
		cgd7_790	hypothetical protein					1	1	676.333

		cgd7_800	hypothetical protein		1				1	1.935
		cgd7_850	putative patched family protein	1					1	0.477
		cgd7_860	large protein with possible conserved (animals and fungi) N-terminal and C-terminal regions				1		1	17.045
		cgd7_870	hypothetical protein	1					1	0.324
		cgd7_890	hypothetical protein	1					1	0.423
		cgd7_920	protein with 2 possible TPR domains, possible n-terminal acetyltransferase			1			1	2.548
		cgd7_970	Syf1p. protein with 8 HAT domains	1					1	0.455
		cgd7_980	protein with MYND plus SET domains plus 5 Ank repeats		1				1	1.717
		cgd7_990	hypothetical protein			1			1	2.532
		cgd8_10	uncharacterized protein	1					1	0.391
		cgd8_100	hypothetical protein	1					1	0.306
		cgd8_1000	hypothetical protein	1					1	0.392
		cgd8_1010	hypothetical protein	1					1	0.317
		cgd8_1020	hypothetical protein	1					1	0.479
		cgd8_1040	hypothetical protein	1					1	0.426
		cgd8_1090	hypothetical protein		1				1	1.692
		cgd8_1140	Sec14d domain containing protein		1				1	1.851
		cgd8_1180	coronin like WD40 repeat protein			1			1	3.110
		cgd8_1190	hypothetical protein				1		1	5.818
		cgd8_120	hypothetical protein	1					1	0.382
		cgd8_1220	uncharacterized protein with threonine repeats	1					1	0.415
		cgd8_1260	hypothetical protein	1					1	0.494
		cgd8_1310	hypothetical protein	1					1	0.448
		cgd8_1360	hypothetical protein				1		1	13.676
		cgd8_1380	hypothetical protein				1		1	5.564
		cgd8_1450	11x WD40 repeats containing protein of plant origin					1	1	47.471
		cgd8_1470	hypothetical protein				1		1	12.307
		cgd8_1500	FH2 domain containing protein				1		1	7.457
		cgd8_1520	coiled coil protein	1					1	0.337
		cgd8_1540	coiled coil, low complexity protein	1					1	0.602
		cgd8_1590	uncharacterized protein	1					1	0.327
		cgd8_1630	hypothetical protein	1					1	0.370
		cgd8_1680	hypothetical protein				1		1	8.481
		cgd8_1690	hypothetical protein	1					1	0.359
		cgd8_1710	hypothetical protein				1		1	13.960
		cgd8_1740	predicted secrete protein, paralog within GGC gene family of secreted cryptospordium proteins	1					1	0.518
		cgd8_1760	secreted protein, signal peptide			1			1	2.679
		cgd8_1770	HxYxP motif containing repeats seen only in C. elegans F29G6.3b, signal peptide					1	1	121.562
		cgd8_1780	hypothetical protein, signal peptide	1					1	0.356
		cgd8_180	hypothetical protein				1		1	9.192
		cgd8_1810	hypothetical protein					1	1	61.027
		cgd8_1830	pelota/Dom34; pelota like RNA binding domain		1				1	1.996
		cgd8_1860	LisH domain at N + WD40 repeats				1		1	23.827
		cgd8_1890	hypothetical protein			1			1	4.716
		cgd8_1930	large protein with a GCN1 domain	1					1	0.343
		cgd8_1960	hypothetical protein	1					1	0.332
		cgd8_2040	POZ domain protein, related to tetramerization domain of K channel		1				1	1.692
		cgd8_2120	hypothetical protein	1					1	0.537
		cgd8_2140	hypothetical protein	1					1	0.350
		cgd8_2150	proliferating cell nuclear antigen PCNA	1					1	0.417
		cgd8_2160	shares a domain with poly(ADP) ribose glycohydrolases, some protein kinase A anchoring proteins and baculovirus HzNV Orf103, possible transmembrane domain within N-terminus	1					1	0.348
		cgd8_2230	hypothetical protein	1					1	0.299
		cgd8_2290	hypothetical protein		1				1	2.556
		cgd8_2300	brahma like protein with a HSA domain, SNF2 like helicase and a bromo domain		1				1	1.468
		cgd8_2310	hypothetical protein	1					1	0.470
		cgd8_2400	hypothetical protein	1					1	0.388
		cgd8_2460	hypothetical protein with possible SAP domain	1					1	0.462
		cgd8_2530	sushi-domain containing secreted protein; with a signal peptide, low complexity region followed by a sushi domain		1				1	1.921
		cgd8_2570	hypothetical protein	1					1	0.352
		cgd8_2590	hypothetical protein	1					1	0.403
		cgd8_260	hypothetical protein	1					1	0.576
		cgd8_2600	hypothetical protein	1					1	0.362
		cgd8_2620	hypothetical protein		1				1	1.871
		cgd8_2660	hypothetical protein				1		1	13.192
		cgd8_2680	hypothetical secreted protein, signal peptide	1					1	0.551
		cgd8_2690	hypothetical protein		1				1	1.860
		cgd8_2740	hypothetical protein	1					1	0.359
		cgd8_2750	hypothetical protein			1			1	2.487
		cgd8_2830	LRR repeats protein	1					1	0.511

		cgd8_2980	hypothetical protein		1				1	1.514
		cgd8_30	signal peptide, predicted secreted protein of cryptosprodidium-specific SKSR gene family			1			1	3.067
		cgd8_3010	Smc ABC ATpase	1					1	0.424
		cgd8_3020	hypothetical protein	1					1	0.388
		cgd8_3030	hypothetical protein, signal peptide, secreted protein	1					1	0.529
		cgd8_3050	hypothetical protein				1		1	21.755
		cgd8_3080	Swr1p like SWI/SNF2 family ATpase with a HSA domain at the N-terminus probably involved in chromatin remodelling	1					1	0.427
		cgd8_310	hypothetical protein		1				1	1.760
		cgd8_3110	12x WD40 repeat containing protein					1	1	123.457
		cgd8_3160	hypothetical protein			1			1	5.056
		cgd8_3170	hypothetical protein	1					1	0.438
		cgd8_320	hypothetical protein	1	1				2	1.092
		cgd8_330	hypothetical protein	1		1			2	1.696
		cgd8_3410	hypothetical protein	1					1	0.469
		cgd8_3490	hypothetical protein				1		1	9.775
		cgd8_3500	hypothetical protein			1			1	4.821
		cgd8_3530	predicted secreted protein				1		1	16.731
		cgd8_3540	predicted secreted protein					1	1	54.426
		cgd8_3550	hypothetical protein			1			1	2.390
		cgd8_3560	predicted secreted protein				1		1	6.427
		cgd8_3570	predicted secreted protein			1			1	4.477
		cgd8_3580	hypothetical protein, signal peptide, paralogs				1		1	9.708
		cgd8_3590	predicted secreted protein				1		1	12.880
		cgd8_3650	predicted DM-LD37668p			1			1	4.149
		cgd8_3700	hypothetical protein				1		1	6.749
		cgd8_3710	hypothetical protein				1		1	8.772
		cgd8_3720	D441/Mpe1p like protein with a zinc knuckle (C2HC) and PhD Zn finger domain					1	1	35.163
		cgd8_3750	predicted secreted protein					1	1	343.094
		cgd8_3780	hypothetical protein	1					1	0.541
		cgd8_3790	Sec14'Sec14'	1					1	0.523
		cgd8_3800	hypothetical protein					1	1	94.614
		cgd8_3810	hypothetical protein				1		1	15.164
		cgd8_3830	hypothetical protein				1		1	18.637
		cgd8_3840	hypothetical protein		1				1	1.306
		cgd8_390	hypothetical protein			1			1	2.521
		cgd8_3930	LepA like TRAFAC class GTpase, 2 transmembrane domain near C	1					1	0.438
		cgd8_3960	RIKEN cDNA 9430077D24 gene, putative	1					1	0.339
		cgd8_3980	hypothetical protein	1					1	0.395
		cgd8_4020	hypothetical protein		1				1	1.597
		cgd8_4070	SANT domain containing protein	1					1	0.337
		cgd8_4080	WD-repeat protein	1					1	0.421
		cgd8_410	possible secreted protein with long stretch of threonines, putative mucin					1	1	39.095
		cgd8_4110	hypothetical protein	1					1	0.519
		cgd8_4190	uncharacterized glycine-rich low complexity protein	1					1	0.313
		cgd8_420	Low complexity coiled coil protein	1					1	0.473
		cgd8_4200	uncharacterized protein with several coiled-coil regions				1		1	8.164
		cgd8_4280	zz domain protein				1		1	7.415
		cgd8_4300	hypothetical protein		1				1	1.468
		cgd8_4310	hypothetical protein		1				1	2.230
		cgd8_4380	SNW family nuclear protein			1			1	5.523
		cgd8_4390	hypothetical protein			1			1	3.044
		cgd8_4420	WD40 repeat and RING finger domain-containing protein				1		1	19.626
		cgd8_4450	hypothetical protein		1				1	1.924
		cgd8_4470	hypothetical protein	1					1	0.583
		cgd8_4510	hypothetical protein				1		1	11.685
		cgd8_4520	large protein with possible domain of nucleoporin, Non-repetitive/WGA-negative nucleoporin		1				1	2.142
		cgd8_4540	predicted coiled coil protein		1				1	1.668
		cgd8_4590	hypothetical protein	1					1	0.327
		cgd8_4600	hypothetical protein	1					1	0.430
		cgd8_4610	hypothetical protein	1					1	0.405
		cgd8_4620	ISWI related chromatinic protein with an apicomplexan specific domain architecture composed of 3x PHD+SNF2 ATpase+2xPHD					1	1	48.753
		cgd8_4640	WD40 repeat				1		1	18.713
		cgd8_4660	hypothetical protein, signal peptide		1				1	1.447
		cgd8_470	MRG/Alp3 like proteini with a chromodomain and an MRG domain	1					1	0.316
		cgd8_4700	hypothetical protein	1					1	0.307
		cgd8_4710	hypothetical protein					1	1	71.536
		cgd8_4770	hypothetical protein	1					1	0.500
		cgd8_4780	large protein with possible central conserved domain	1					1	0.442

		cgd8_4800	SSM4 like ring finger with a forkhead associated (FHA) domain (apicomplexan-specific architecture)		1				1	1.933
		cgd8_4820	hypothetical protein	1					1	0.375
		cgd8_4840	large protein with 2 MYB domains plus low complexity; GA repeat and Q repeat at the C-terminus			1			1	5.452
		cgd8_490	uncharacterized large low complexity coiled coil protein	1					1	0.372
		cgd8_4920	WD repeats containing protein	1					1	0.288
		cgd8_4960	hypothetical protein, signal peptide				1		1	9.100
		cgd8_4980	hypothetical protein	1					1	0.483
		cgd8_50	signal peptide, predicted secreted protein of cryptosprodidium-specific FGLN gene family			1			1	4.918
		cgd8_5010	hypothetical protein				1		1	19.586
		cgd8_5020	starch binding domain containing protein, possible plant origin	1					1	0.409
		cgd8_5040	hypothetical protein	1					1	0.299
		cgd8_5070	hypothetical protein	1					1	0.467
		cgd8_5100	hypothetical protein			1			1	2.796
		cgd8_5110	hypothetical protein				1		1	6.575
		cgd8_5170	hypothetical protein	1					1	0.289
		cgd8_5180	hypothetical protein	1					1	0.444
		cgd8_5190	large protein with central conserved domain, possible BCRT domain	1					1	0.443
		cgd8_5250	protein with 2x PHD domains	1					1	0.346
		cgd8_5290	large protein with possible signal peptide and acidic plus glycine repeats			1			1	2.282
		cgd8_5300	signal peptide containing protein			1			1	5.676
		cgd8_5310	signal peptide containing protein		1				1	1.431
		cgd8_5330	hypothetical protein				1		1	24.100
		cgd8_5340	coiled coil protein					1	1	71.171
		cgd8_550	large uncharacterized protein	1					1	0.438
		cgd8_620	hypothetical protein	1					1	0.398
		cgd8_660	large low complexity protein	1					1	0.494
		cgd8_700	predicted secreted protein, signal peptide with several threonines, possible mucin	1					1	0.457
		cgd8_720	hypothetical protein	1					1	0.281
		cgd8_740	prefoldin like molecular chaperone	1					1	0.337
		cgd8_760	predicted secreted protein, 1 or more transmembrane domain, signal peptide	1					1	0.512
		cgd8_770	hypothetical protein	1					1	0.384
		cgd8_830	hypothetical protein	1					1	0.487
		cgd8_970	VPS13 like protein involved in vacuolar protein trafficking	1					1	0.438
	Unknown Total			612	211	196	185	148	1352	16.705
**Unknown Total**				801	293	272	267	216	1849	20.490
**Grand Total**				**###**	**481**	**481**	**481**	**481**	**3137**	**26.292**
