# Supplementary material for: Transcriptome analysis reveals unique metabolic features in the Cryptosporidium parvum Oocysts associated with environmental survival and stresses
Source: BMC Genomics. 2012 Nov 21;13:647. doi: 10.1186/1471-2164-13-647 (PMC3542205; doi:10.1186/1471-2164-13-647)

Figure S2.
Comparison of data derived from Mauzy et al (2012) and the present qRT-PCR sutdy on the relative levels of *Cryptosporidium parvum* LDH, ADH and AceCL genes in the intracellular developmental stages. Data from Mauzy et al were extracted from the CryptoDB databases (http://www.CryptoDB.org).

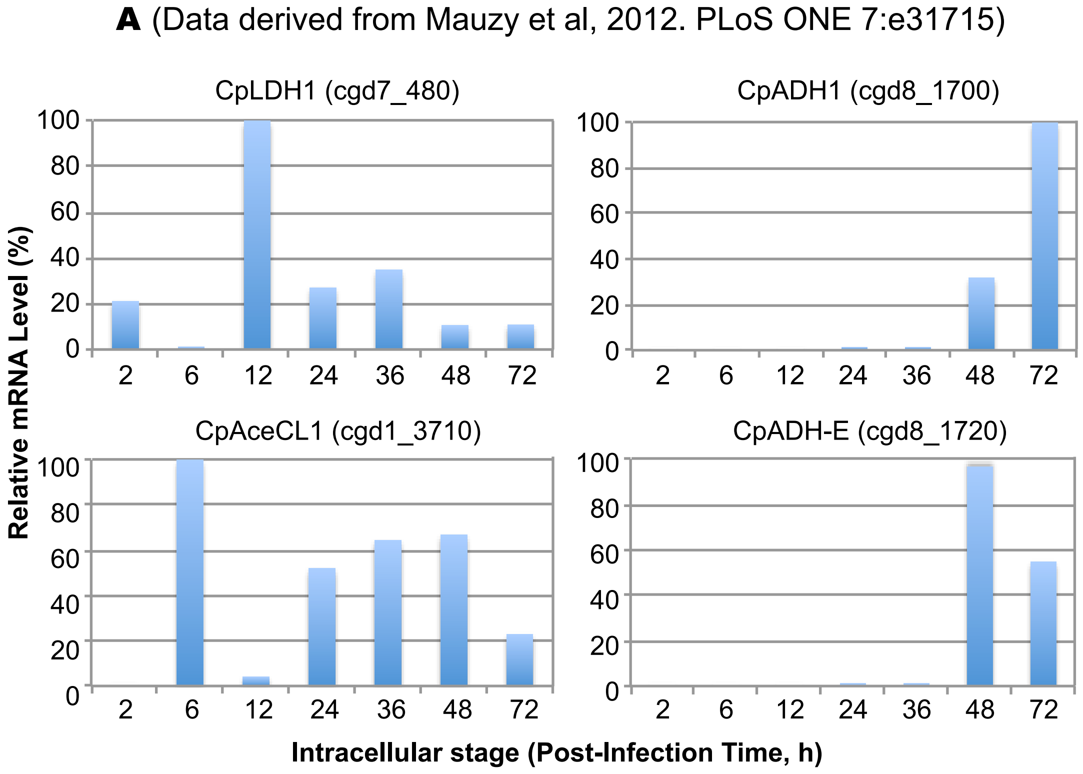

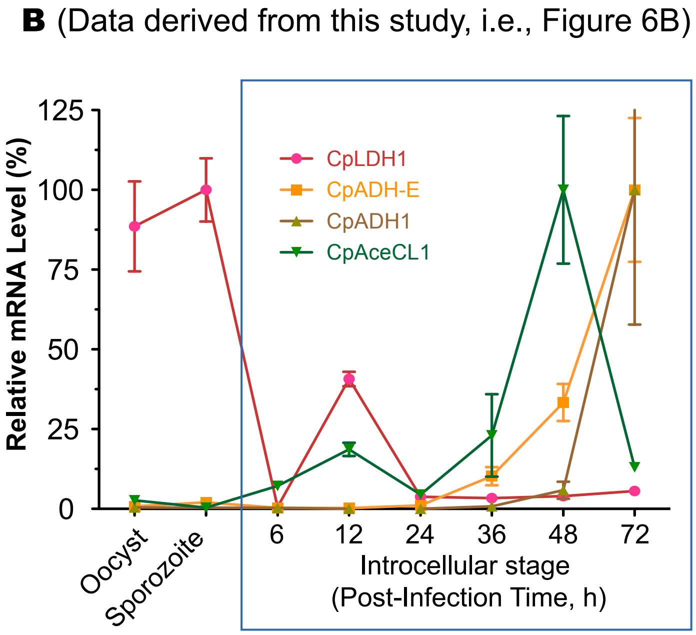

Supplement: Additional file 3 — Figure S2. Comparison of data derived from Mauzy et al. (2012) and this study by qRT-PCR on the relative levels of Cryptosporidium parvum LDH, ADH and AceCL genes in the intracellular developmental stages. Data from Mauzy et al. (2012) were extracted from the CryptoDB databases ( http://www.CryptoDB.org). [file 1471-2164-13-647-S3.pdf]
