# Supplementary material for: Transcriptome analysis reveals unique metabolic features in the Cryptosporidium parvum Oocysts associated with environmental survival and stresses
Source: BMC Genomics. 2012 Nov 21;13:647. doi: 10.1186/1471-2164-13-647 (PMC3542205; doi:10.1186/1471-2164-13-647)
Supplement: Additional file 4 — Table S2. A) Functional clarification of the top 100 genes in Toxoplasma gondii with the highest levels of expression in the oocysts sporulated for 10 days; B) List of the top 100 highly expressed genes in the Toxoplasma gondii oocysts sporulated for 10 days. Original data were generated by Fritz et al. (2012) and extracted from http://www.ToxoDB.org. [file 1471-2164-13-647-S4.pdf]

Table S2A: Functional clarification of top 100 genes in *Toxoplasma gondii* with the highest levels of expression in the oocysts sporulated for 10 days

Functional group	Gene count
**Antigen and/or secretory protein**	18
beta antigen (1)	
GRA (6)	
MIC (7)	
SRS (4)	
**Enzyme**	10
AA synthesis (1)	
energy (2)	
glycosylases (1)	
protein modification (3)	
undefined (3)	
**Chromatin (Histone)**	3
**Cytoskeletal**	3
**Redox homeostasis**	3
**Ribosomal proteins**	3
**Chaperonin (HSP)**	2
**Gene expression (TIF)**	2
**Protein degradation (ubiquitin)**	2
**Calcium-binding (CaM)**	1
**Structure (COWP)**	1
**Hypothetical**	52
**Grand Total**	100

Figure S2B. List of the top 100 highly expressed genes in the Toxoplasma gondii oocysts sporulated for 10 days. Original data were generated by Fritz et al. (2012) and extracted from http://www.ToxoDB.org.

Rank	Gene ID	Level (d10 oocysts)	Product Description	Functional group	Subgroup
1	TGME49_081590	13.7009755	hypothetical protein	hypothetical	hypothetical
2	TGME49_070250	13.616716	dense granule protein 1 / major antigenp24	antigen	GRA
3	TGME49_037080	13.3381775	hypothetical protein	hypothetical	hypothetical
4	TGME49_093690	13.2964225	profilin family protein	cytoskeletal	profilin
5	TGME49_120530	13.1842575	hypothetical protein	hypothetical	hypothetical
6	TGME49_086450	13.181792	dense granule protein 5 precursor	antigen	GRA
7	TGME49_050710	13.149738	microneme protein 10	antigen	MIC
8	TGME49_002100	13.106612	hypothetical protein	hypothetical	hypothetical
9	TGME49_058550	13.0106595	SRS28 (= SporoSAG)	antigen	SRS
10	TGME49_076850	12.9646045	late embryogenesis abundant domain-containing protein	hypothetical	hypothetical
11	TGME49_014220	12.9475755	hypothetical protein	hypothetical	hypothetical
12	TGME49_020400	12.908191	actin depolymerizing factor	cytoskeletal	actin
13	TGME49_059900	12.903928	hypothetical protein, conserved	hypothetical	hypothetical
14	TGME49_002110	12.854348	hypothetical protein	hypothetical	hypothetical
15	TGME49_119890	12.849931	hypothetical protein	hypothetical	hypothetical
16	TGME49_094600	12.7870565	hypothetical protein	hypothetical	hypothetical
17	TGME49_120540	12.760554	hypothetical protein	hypothetical	hypothetical
18	TGME49_013280	12.7605255	hypothetical protein	hypothetical	hypothetical
19	TGME49_003310	12.749144	dense granule protein 7	antigen	GRA
20	TGME49_027100	12.748301	glutaredoxin, putative	Redox homeostasis	redoxin
21	TGME49_116190	12.738828	superoxide dismutase, putative	Redox homeostasis	SOD
22	TGME49_076880	12.6711495	late embryogenesis abundant domain-containing protein	hypothetical	hypothetical
23	TGME49_108840	12.6364755	SRS51 (= SRS3)	antigen	SRS
24	TGME49_004520	12.586474	hypothetical protein	hypothetical	hypothetical
25	TGME49_087250	12.5827725	hypothetical protein	hypothetical	hypothetical
26	TGME49_008450	12.546591	serine proteinase inhibitor, putative	enzyme	protein modification
27	TGME49_003720	12.5409265	vitamin K epoxide reductase complex subunit 1, putative	enzyme	unknown
28	TGME49_106270	12.4310705	hypothetical protein	hypothetical	hypothetical
29	TGME49_000360	12.430844	hypothetical protein	hypothetical	hypothetical
30	TGME49_108020	12.3837425	SRS57 (= SAG3, P43)	antigen	SRS
31	TGME49_004530	12.36251	microneme protein MIC11	antigen	MIC
32	TGME49_119560	12.3558365	microneme protein MIC3	antigen	MIC
33	TGME49_066860	12.3482205	BTB/POZ domain-containing protein	hypothetical	hypothetical
34	TGME49_088650	12.3452855	hypothetical protein	hypothetical	hypothetical
35	TGME49_070950	12.3446345	hypothetical protein	hypothetical	hypothetical
36	TGME49_019820	12.3179475	polyubiquitin, putative	protein degradation	ubiquitin
37	TGME49_053820	12.2409775	hypothetical protein, conserved	hypothetical	hypothetical
38	TGME49_067470	12.209846	hypothetical protein	hypothetical	hypothetical
39	TGME49_009610	12.1808045	oocyst wall protein COWP, putative	structure	COWP
40	TGME49_049370	12.1675755	translation initiation factor SUI1, putative	gene expression	TIF
41	TGME49_058470	12.136311	hypothetical protein	hypothetical	hypothetical
42	TGME49_089690	12.115494	glyceraldehyde-3-phosphate dehydrogenase	enzyme	energy
43	TGME49_093740	12.020502	hypothetical protein	hypothetical	hypothetical
44	TGME49_009910	11.995331	histone H2B variant 1	chromatin	histone
45	TGME49_039740	11.968661	hypothetical protein	hypothetical	hypothetical
46	TGME49_057380	11.9351895	hypothetical protein	hypothetical	hypothetical
47	TGME49_053690	11.8690545	hypothetical protein	hypothetical	hypothetical
48	TGME49_017890	11.8021	peroxiredoxin	Redox homeostasis	redoxin
49	TGME49_071930	11.798724	hypothetical protein, conserved	hypothetical	hypothetical
50	TGME49_060190	11.7461065	microneme protein, putative	antigen	MIC

51	TGME49_049240	11.70397	calmodulin	Calcium-binding	CaM
52	TGME49_075810	11.66303	ribosomal protein S10, putative	ribosome biogenesis	ribosomal protein
53	TGME49_080430	11.65981	hypothetical protein	hypothetical	hypothetical
54	TGME49_071490	11.649778	hypothetical protein	hypothetical	hypothetical
55	TGME49_086090	11.6027615	translation initiation factor SUI1, putative	gene expression	TIF
56	TGME49_091890	11.596418	microneme protein MIC1	antigen	MIC
57	TGME49_032940	11.5887815	small heat shock protein 20	Chaperonin	HSP
58	TGME49_115260	11.5792415	alanine dehydrogenase, putative	enzyme	AA synthesis
59	TGME49_076860	11.5683955	late embryogenesis abundant domain-containing protein	hypothetical	hypothetical
60	TGME49_112940	11.5603165	hypothetical protein	hypothetical	hypothetical
61	TGME49_031080	11.5151295	hypothetical protein	hypothetical	hypothetical
62	TGME49_005090	11.497294	hypothetical protein	hypothetical	hypothetical
63	TGME49_027620	11.496448	28 kDa antigen	antigen	GRA
64	TGME49_055660	11.487851	hypothetical protein	hypothetical	hypothetical
65	TGME49_066460	11.473115	ubiquitin-like protein SMT3 precursor, putative	protein degradation	ubiquitin
66	TGME49_018260	11.471261	histone H3.3 variant	chromatin	Histone
67	TGME49_002090	11.449114	hypothetical protein	hypothetical	hypothetical
68	TGME49_059630	11.41997	hypothetical protein, conserved	hypothetical	hypothetical
69	TGME49_032410	11.4188375	hypothetical protein	hypothetical	hypothetical
70	TGME49_014940	11.410756	MIC2-associated protein M2AP	antigen	MIC
71	TGME49_089600	11.4105075	Hsp20/alpha crystallin domain-containing protein	Chaperonin	HSP
72	TGME49_013940	11.3763095	CHCH domain-containing protein	hypothetical	hypothetical
73	TGME49_077080	11.3529265	microneme TgMIC5 protein	antigen	MIC
74	TGME49_072240	11.345837	hypothetical protein	hypothetical	hypothetical
75	TGME49_062470	11.3437315	beta antigen, putative	antigen	beta antigen
76	TGME49_109810	11.331183	60S acidic ribosomal protein P2, putative	ribosome biogenesis	ribosomal protein
77	TGME49_001860	11.283525	hypothetical protein	hypothetical	hypothetical
78	TGME49_029320	11.2675365	haloacid dehalogenase-like hydrolase domain-containing protein	enzyme	unknown
79	TGME49_029330	11.2512265	haloacid dehalogenase-like hydrolase domain containing protein	enzyme	unknown
80	TGME49_034550	11.2503755	60S ribosomal protein L29, putative	ribosome biogenesis	ribosomal protein
81	TGME49_036040	11.218365	fructose-1,6-bisphosphate aldolase	enzyme	energy
82	TGME49_046130	11.1989775	serine protease inhibitor, putative	enzyme	protein modification
83	TGME49_033460	11.163425	SRS29B (= SAG1, P30)	antigen	SRS
84	TGME49_024130	11.1455005	hypothetical protein	hypothetical	hypothetical
85	TGME49_013850	11.137022	hypothetical protein	hypothetical	hypothetical
86	TGME49_080380	11.1196235	non-transmembrane antigen	enzyme	Glycosylases
87	TGME49_026020	11.113592	transmembrane domain-containing protein	hypothetical	hypothetical
88	TGME49_026500	11.1130655	hypothetical protein	hypothetical	hypothetical
89	TGME49_046550	11.094218	eukaryotic aspartyl protease, putative	enzyme	protein modification
90	TGME49_016180	11.0912385	hypothetical protein	hypothetical	hypothetical
91	TGME49_054800	11.0320385	hypothetical protein	hypothetical	hypothetical
92	TGME49_116400	11.0203125	tubulin alpha chain	cytoskeletal	tubulin
93	TGME49_039260	11.020137	histone H4, putative	chromatin	histone
94	TGME49_075440	11.0162525	granule antigen protein GRA6	antigen	GRA
95	TGME49_048990	10.971121	hypothetical protein	hypothetical	hypothetical
96	TGME49_092350	10.970051	hypothetical protein	hypothetical	hypothetical
97	TGME49_055380	10.9643055	hypothetical protein	hypothetical	hypothetical
98	TGME49_116710	10.9216445	hypothetical protein	hypothetical	hypothetical
99	TGME49_054720	10.9094295	dense granule protein GRA8	antigen	GRA
100	TGME49_058410	10.895404	hypothetical protein	hypothetical	hypothetical
