# Supplementary material for: Transcriptome analysis reveals unique metabolic features in the Cryptosporidium parvum Oocysts associated with environmental survival and stresses
Source: BMC Genomics. 2012 Nov 21;13:647. doi: 10.1186/1471-2164-13-647 (PMC3542205; doi:10.1186/1471-2164-13-647)

Figure S3.
Relative levels of the two *Toxoplasma gondii* LDH genes in oocysts, trachyzoites and bradyzoites. Data were extracted from the ToxoDB databases (http://www.ToxoDB.org).

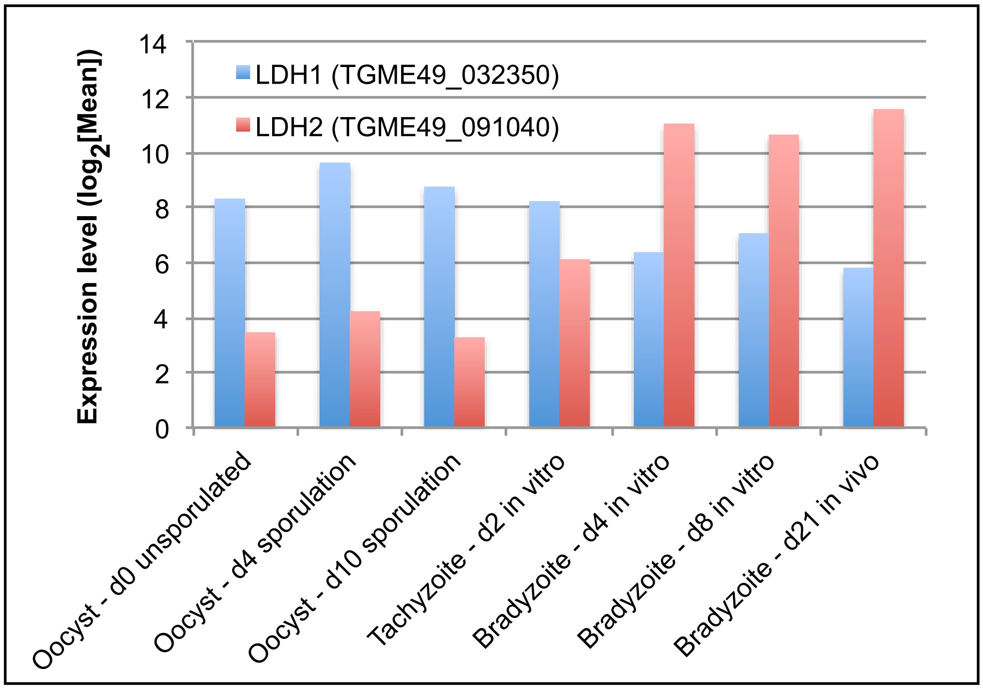

Supplement: Additional file 5 — Figure S3. Relative levels of the two Toxoplasma gondii LDH genes in oocysts, tachyzoites and bradyzoites. Data used in this analysis were extracted from the ToxoDB databases ( http://www.ToxoDB.org). [file 1471-2164-13-647-S5.pdf]
