# Supplementary material for: Transcriptome analysis reveals unique metabolic features in the Cryptosporidium parvum Oocysts associated with environmental survival and stresses
Source: BMC Genomics. 2012 Nov 21;13:647. doi: 10.1186/1471-2164-13-647 (PMC3542205; doi:10.1186/1471-2164-13-647)
Supplement: Additional file 6 — Table S3. Complete list of significantly regulated genes in the oocysts upon UV-irradiation and recovered for 0.5 h and 5 h. Genes are grouped into 9 clusters based their expression patterns. [file 1471-2164-13-647-S6.pdf]

Table S3. Complete list of significantly regulated genes in the oocysts upon UV irradiation and recovered for 0.5 h and 5 h

Cluster 1		0.5 h: Up-regulated (Ratio > 1.2) vs. 5 h: Up-regulated (Ratio > 1.2)		
No.	GeneID	Fold Change (0.5 h)	Fold change (5 h)	Gene Description
1	cgd3_1410	2.192	1.287	small hypothetical protein
2	cgd4_3850	1.694	1.222	putative t-complex protein 1, gamma subunit
3	cgd7_1790	1.578	1.240	protein with BRIGHT domain like HTH domain at N-terminus
4	cgd2_1290	1.521	1.238	hypothetical protein, signal peptide, transmembrane domain near C-terminus
5	cgd1_2880	1.467	1.340	13 kda membrane protein subunit
6	cgd7_1610	1.434	1.362	uncharacterized low complexity protein
7	cgd5_3380	1.402	1.253	protein with signal peptide and possible kazal domain
8	cgd5_2590	1.392	1.322	Alg5 like dolichyl-phosphate beta-glucosyltransferase
9	cgd5_2530	1.387	1.225	protein with 12 transmembrane domains, possible apicomplexan-specific protein
10	cgd1_1140	1.387	1.331	conserved hypothetical protein
11	cgd2_3490	1.380	1.204	protein with low complexity regions and an apicomplexan specific domain
12	cgd6_5230	1.366	1.229	hypothetical protein
13	cgd5_3090	1.352	1.210	N-acetyltransferase subunit ARD1
14	cgd2_1010	1.350	1.215	conserved hypothetical protein
15	cgd2_3560	1.322	1.525	hypothetical protein with 8 transmembrane domains
16	cgd7_2030	1.319	1.243	bacterial-like Sir2 family protein
17	cgd4_4360	1.281	1.270	ring finger domain containing protein
18	cgd1_2340	1.274	1.267	hypothetical protein
19	cgd8_1240	1.265	1.521	DNA polymerase epsilon catalytic subunit
20	cgd2_1250	1.261	1.357	DNA replication licensing factor MCM4 like AAA+ ATpase
21	cgd6_3370	1.260	1.227	microtubule-associated protein
22	cgd2_1590	1.257	1.322	extracellular protein with signal peptide, 5xEGF and apple domains
23	cgd2_1110	1.245	1.414	silencer-associated factor, putative
24	cgd7_3420	1.237	1.745	hypothetical protein
25	cgd6_5500	1.222	1.236	hypothetical protein
26	cgd2_960	1.221	1.235	hypothetical protein
27	cgd1_3660	1.214	1.201	hypothetical protein
28	cgd6_4280	1.210	1.247	hypothetical protein having a signal peptide
29	cgd3_3480	1.208	1.393	large Sec7 domain containing protein
30	cgd7_1980	1.207	1.327	gamma tubulin
31	cgd6_4740	1.204	1.215	hypothetical protein with 6 transmembrane domains and signal peptide

Cluster 2		0.5 h: Up-regulated (Ratio > 1.2) vs. 5 h: Normal (Ratio = 0.8-1.2)		
No.	GeneID	Signal Ratio (0.5 h)	Signal Ratio (5 h)	Description
1	cgd8_5030	2.479	1.169	articulin family protein, Pfs77 protein-related
2	cgd7_3840	2.432	1.079	conserved hypothetical protein
3	cgd3_2070	2.119	1.068	hypothetical protein
4	cgd7_990	2.003	1.074	hypothetical protein
5	cgd1_840	1.985	1.032	conserved hypothetical protein
6	cgd8_3510	1.861	0.987	thioredoxin-like protein, fragment
7	cgd7_270	1.860	1.111	conserved hypothetical protein
8	cgd3_3320	1.845	1.142	putative phenylalanyl-tRNA synthetase
9	cgd3_270	1.808	1.068	hypothetical protein
10	cgd4_4030	1.808	1.089	hypothetical protein
11	cgd7_4710	1.786	1.127	conserved hypothetical protein
12	cgd3_220	1.782	1.074	hypothetical protein
13	cgd7_5380	1.746	0.919	CtIP type zinc knuckle (C2H2)
14	cgd4_4270	1.729	0.980	hypothetical protein
15	cgd4_2480	1.723	1.078	hypothetical protein with a signal peptide, gene within locus of signal-peptide containing hypothetical proteins
16	cgd7_4690	1.660	1.112	RF-C paralog (Ctf18p) AAA+ ATpase
17	cgd2_1090	1.600	1.129	diacylglycerol acyltransferase 1
18	cgd8_320	1.595	1.034	hypothetical protein
19	cgd8_4450	1.576	1.153	hypothetical protein
20	cgd8_1340	1.564	1.122	conserved hypothetical protein
21	cgd5_2020	1.532	1.075	extracellular protein
22	cgd8_3850	1.514	1.153	Ubc6p like ubiquiting conjugating enzyme E2, possible transmembrane domain at C
23	cgd6_5160	1.467	1.101	hypothetical protein with conserved carboxy terminal region (KIAA1078 protein)
24	cgd8_2170	1.466	0.983	histone H2A
25	cgd8_3250	1.459	1.070	ABC1 like protein kinase
26	cgd1_3680	1.454	1.129	extracellular membrane associated protein with 3 EGF domains and a transmembrane domain

27	cgd6_2850	1.450	0.843	hypothetical protein
28	cgd3_2000	1.433	0.988	WD repeat protein
29	cgd2_2360	1.422	1.026	Dop1p family protein
30	cgd4_4300	1.422	1.146	beta adaptin
31	cgd5_2320	1.420	0.958	putative prohibitin with PHB domain
32	cgd7_2100	1.419	1.058	WD repeat protein
33	cgd7_2740	1.416	0.843	WD40 protein
34	cgd2_3140	1.414	1.053	hypothetical protein
35	cgd1_620	1.407	1.129	hypothetical protein
36	cgd4_3390	1.399	1.186	neutral sphingomyelinase activation associated factor-like BEACH domain containing protein
37	cgd2_440	1.392	1.086	signal peptide, serine proline rich, possible low mw mucin glycoprotein locus of 6 genes
38	cgd7_1400	1.391	1.123	signal peptide containing protein
39	cgd8_2150	1.379	0.850	proliferating cell nuclear antigen PCNA
40	cgd2_1000	1.378	0.978	bruno-like, RRM domain RNA binding protein
41	cgd8_3430	1.372	1.097	zincin/aminopeptidase N like metalloprotease
42	cgd8_860	1.371	1.048	coatomer protein complex subunit alpha, putative
43	cgd3_3520	1.364	1.168	putative sodium/calcium cation exchanger having 12 transmembrane domains and signal peptide
44	cgd5_1590	1.363	1.063	hypothetical protein
45	cgd4_3690	1.362	0.944	large glycine-rich repeat low complexity protein
46	cgd1_1540	1.361	1.097	dynein intermediate chain
47	cgd8_4460	1.360	1.058	cell differentiation protein rcd1, putative
48	cgd1_3810	1.359	1.074	conserved hypothetical protein
49	cgd4_3760	1.358	1.156	hypothetical protein
50	cgd3_3260	1.357	1.071	hypothetical protein
51	cgd3_3430	1.350	0.928	extracellular protein with a signal peptide sequence, MAM domain and a Cu amine oxidase domain
52	cgd4_3410	1.348	0.946	RNA binding protein, putative
53	cgd6_2060	1.348	1.044	cdc39p protein-like; C-terminal Not1, CCR4-Not complex component; Not1
54	cgd5_4100	1.345	1.031	hypothetical protein conserved in apicomplexa
55	cgd8_3440	1.342	1.136	importin beta like ARM repeat alpha superhelix
56	cgd8_2090	1.342	1.054	cation-transporting ATpase 2 with 8 transmembrane domains
57	cgd6_1290	1.338	1.151	ring finger, membrane associated, possible signal peptide
58	cgd6_3440	1.337	0.972	hypothetical protein
59	cgd1_2770	1.332	1.110	RNA polymerase 1 beta subunit
60	cgd8_4330	1.331	1.105	fibrillarin. RNA methylase
61	cgd7_4110	1.331	1.091	WD40 protein (part of U3 processesome)
62	cgd6_4370	1.330	1.056	SPBC24E9.10-like. RRM domain protein, no PWI domain.
63	cgd4_490	1.329	1.115	hypothetical protein
64	cgd4_2260	1.322	1.112	ribosomal protein PO like protein of the L10 family
65	cgd7_4510	1.321	1.181	ATP-binding cassette, sub-family C (CFTR/MRP), member 2; Canalicular multispecific organic anion transporter; multidrug resist
66	cgd7_1090	1.320	0.982	hypothetical protein
67	cgd3_1100	1.320	0.969	hypothetical protein
68	cgd4_3280	1.320	1.043	hypothetical protein
69	cgd3_2620	1.318	0.989	putative DNA-directed RNA polymerase,possible RNA polymerase A/beta'/A'' subunit, long PHYSPTS repeat at C-terminus
70	cgd8_4910	1.312	0.828	PAN2 family 3'5' exonuclease, UBC hydrolase (inactive) plus nuclease
71	cgd8_4180	1.312	1.001	uncharacterized glycine-rich low complexity protein
72	cgd1_2200	1.311	0.810	hypothetical protein
73	cgd7_1000	1.311	1.023	F27M3_19 plant like RRM plus AlkB domain containing protein
74	cgd7_1890	1.310	1.007	KH domain protein
75	cgd4_2970	1.308	1.106	RAD1, NH2 terminal ERCC4-like helicase domain
76	cgd8_4950	1.300	0.923	RecQ4 SF II RNA helicase
77	cgd8_3230	1.299	1.018	conserved hypothetical protein
78	cgd3_1980	1.299	1.040	hypothetical protein
79	cgd5_1050	1.299	0.915	hypothetical protein
80	cgd7_1440	1.298	1.064	protein with 5 TPR domains
81	cgd5_2370	1.294	1.171	60S acidic ribosomal protein LP1 like protein of possible plant origin
82	cgd8_2800	1.294	1.063	extracellular protein with a signal peptide, a mucin-like stretch of threonine residues and 6 notch domains; possible mucin
83	cgd5_2650	1.290	1.198	hypothetical protein
84	cgd3_1910	1.290	0.995	hypothetical protein
85	cgd7_4520	1.289	1.074	ABC ATpase (2 ABC domains) with 10 transmembrane domains, adjacent duplicated gene
86	cgd8_260	1.288	0.972	hypothetical protein
87	cgd1_1580	1.288	1.084	RING finger domain at N-terminus, CCCH domain at C-terminus, involved in RNA metabolism, HC-23 protein
88	cgd8_1580	1.287	1.051	saccharomyces Yor006cp like protein conserved across euks and archaea
89	cgd7_2480	1.287	1.046	hypothetical membrane protein

90	cgd3_1540	1.286	1.044	large protein with signal peptide. cysteine-rich, threonine-rich, possible mucin
91	cgd3_510	1.286	1.117	putative fucose translocatorw ith 8 transmembrane domains, within locus of 3 paralogous genes
92	cgd2_2980	1.286	1.156	hypothetical protein
93	cgd6_3160	1.284	1.032	large low complexity protein
94	cgd7_2300	1.283	1.093	translation initiation factor if-5A
95	cgd6_3900	1.281	1.075	hypothetical protein
96	cgd5_1220	1.280	1.087	hypothetical protein
97	cgd8_330	1.278	0.925	hypothetical protein
98	cgd7_3180	1.278	1.054	T complex chaperonin
99	cgd5_650	1.277	1.131	conserved hypothetical protein
100	cgd7_4060	1.276	1.105	signal recognition particle SPR72
101	cgd4_2770	1.275	1.088	11 transmembrane domain protein, possible aa transporter
102	cgd5_970	1.275	1.122	60S ribosomal protein L13A
103	cgd5_2410	1.274	1.054	conserved possible esterase of the beta-lactamase family, of possible plant or bacterial origin, possible transmembrane domain within N terminal region
104	cgd8_1920	1.274	1.042	mannose-6-phosphate isomerase
105	cgd1_1290	1.273	1.094	apicomplexa specific secreted protein Pf (23508265), signal peptide
106	cgd6_2960	1.272	1.019	Bax inhibitor-1 (BI-1). integral membrane protien with 6 or more transmembrane domains
107	cgd8_130	1.272	1.020	conserved hypothetical protein
108	cgd5_2910	1.271	0.930	alpha amylase
109	cgd2_4080	1.271	0.826	RP-A, OB fold single strand binding protein
110	cgd3_4290	1.270	0.902	DNA polymerase alpha catalytic subunit
111	cgd5_3490	1.267	1.162	hypothetical protein with signal peptide
112	cgd6_110	1.266	1.014	conserved protein with signal peptide and transmembrane domain or GPI anchor signal near C-terminus
113	cgd4_30	1.266	1.102	hypothetical protein
114	cgd8_5180	1.266	0.925	hypothetical protein
115	cgd5_2060	1.266	1.065	hypothetical protein with signal peptide and mucin-like threonine repeats
116	cgd7_4450	1.265	1.081	elongation factor EF1-gamma (glutathione S-transferase family)
117	cgd6_4690	1.264	1.102	SAC3/GANP/Rpn12p family
118	cgd2_2670	1.264	1.071	UDP N-acetylglucosamine transporter-like nucleotide sugar transporter with 10 transmembrane domains
119	cgd7_1970	1.263	1.110	nuclear pore associated protein (NLP4) with N-terminal ubiquitin domain
120	cgd6_5130	1.262	0.965	Trm5 RNA methyltransferase
121	cgd4_1920	1.262	0.921	hypothetical protein
122	cgd3_1390	1.261	0.986	hypothetical protein
123	cgd7_5220	1.259	1.172	splicing factor with 3 RRM domains
124	cgd1_680	1.259	1.086	hypothetical protein
125	cgd5_3320	1.258	1.039	hypothetical protein
126	cgd2_3680	1.258	1.064	eukaryotic ribosome biogenesis; Erb1p, putative
127	cgd3_3910	1.256	0.976	prolyl 4-hydroxylase alpha subunit
128	cgd7_1460	1.255	1.100	ribosomal protein S27
129	cgd7_4610	1.255	1.063	divergent membrane protein
130	cgd8_1500	1.254	1.058	FH2 domain containing protein
131	cgd7_4150	1.254	1.096	PP2C phosphatase
132	cgd7_4930	1.254	1.009	secreted glycosyltransferase, possible transmembrane domain near C-terminus
133	cgd7_3660	1.254	0.942	proteasome subunit alpha2, protease of the acylase family and NTN hydrolase fold
134	cgd6_770	1.253	1.023	hypothetical protein
135	cgd8_4770	1.252	0.929	hypothetical protein
136	cgd6_4360	1.252	1.021	hypothetical protein having a signal peptide and 4 transmembrane domains
137	cgd7_1900	1.251	1.057	mitochondrial NADH dehydrogenase
138	cgd3_2400	1.251	1.154	Yn1201cp-family protein
139	cgd3_1590	1.250	1.091	Spb4p, eIF4a-1-family RNA SFII helicase, DEXDc+HELICc domains
140	cgd6_4610	1.249	1.013	mRNA export protein, putative
141	cgd7_70	1.248	1.192	integral membrane protein with 6 transmembrane domains and possible ring domain
142	cgd7_650	1.248	1.097	conserved hypothetical protein
143	cgd7_610	1.248	0.942	apicomplexan conserved protein
144	cgd2_1630	1.247	0.925	cytidine and deoxycytidylate deaminase family, putative
145	cgd8_2590	1.246	1.183	hypothetical protein
146	cgd6_1910	1.246	1.117	conserved hypothetical protein
147	cgd6_1410	1.245	1.048	pre-mRNA splicing factor ATP-dependent RNA helicase, putative
148	cgd3_1850	1.245	1.058	putative casein kinase II regulatory subunit; besthit Pf 23508244
149	cgd5_2420	1.245	0.907	hypothetical protein
150	cgd7_3780	1.244	1.113	cyclin
151	cgd7_140	1.244	1.106	SprT family metallopeptidase
152	cgd3_300	1.243	1.179	rasputin. nuclear transport factor 2 (NTF2) domain plus RRM domain
153	cgd8_2930	1.242	1.147	Eft2p GTpase; translation elongation factor 2 (EF-2)

154	cgd2_2700	1.242	0.985	Brf1p like coiled coil protein
155	cgd6_2470	1.241	1.165	thioredoxin (quiescin Q6 like) having signal peptide and 12 transmembrane domains
156	cgd4_1960	1.241	1.044	ThiF/moeB family
157	cgd5_2070	1.240	1.028	SPAC694.02. SKI family SFII helicase
158	cgd5_1570	1.240	0.884	YIP1 protein like transporter GTpase interacting factor having 5 transmembrane domain
159	cgd8_70	1.238	1.186	conserved hypothetical protein
160	cgd8_1060	1.238	1.010	uncharacterized conserved protein
161	cgd6_3880	1.238	1.017	mitochondrial carrier protein MRS2 with 2 transmembrane domains
162	cgd6_5430	1.237	1.035	hypothetical protein, signal peptide plus transmembrane domain or GPI anchor
163	cgd8_4240	1.237	0.944	possible spliceosome factor
164	cgd1_810	1.237	1.006	protein kinase
165	cgd6_2750	1.236	0.911	cysteine protease, putative
166	cgd7_2830	1.236	0.968	hypothetical low complexity protein
167	cgd2_840	1.235	1.048	phosphatidylinositol N-acetylglucosaminyltransferase subunit PIG-P, involved in GPI anchor biosynthesis, multitransmembrane domain
168	cgd6_1380	1.235	1.027	histone deacetylase HDA2/Rpd3p
169	cgd8_340	1.235	1.063	exoribonuclease PH, putative
170	cgd8_870	1.234	0.941	DNA dependent DNA polymerase alpha subunit, inactive calcineurin like phosphatase subunit
171	cgd3_1230	1.233	1.016	SPAC14C4.06c-like PWI+4CCCH domains
172	cgd8_3590	1.232	1.052	predicted secreted protein
173	cgd4_3310	1.231	1.006	phosphoacetyl glucosamine mutase
174	cgd2_2720	1.230	1.079	ARM repeat protein
175	cgd7_250	1.229	1.132	conserved protein
176	cgd4_4230	1.229	1.130	ATP-dependent helicase, putative
177	cgd5_50	1.228	0.831	signal peptide containing protein
178	cgd2_60	1.227	1.037	hypothetical protein
179	cgd3_4040	1.227	0.977	hypothetical protein with signal peptide, proline rich
180	cgd3_3460	1.227	1.105	hypothetical protein with cysteine rich regions
181	cgd2_4340	1.226	1.059	mitogen-activated protein kinase 2, putative
182	cgd7_4190	1.226	1.032	cation-transporting P-type ATpase with 11 or more transmembrane domains
183	cgd6_1420	1.225	1.053	cdc2-related protein kinase, putative
184	cgd2_2080	1.225	0.977	hypothetical protein
185	cgd2_1480	1.224	0.974	splicing factor U2AF U2 SnRNP auxiliary factor large subunit, RRM domain
186	cgd3_2520	1.224	1.005	conserved hypothetical protein
187	cgd6_2590	1.223	0.940	histone acetyltransferase (MYST family); chromo domain + C2H2 domain
188	cgd4_3000	1.223	1.079	Dbp1p, eIF4a-1 family RNA SFII helicase (DEXDC+HELICc)
189	cgd8_3760	1.223	1.151	predicted membrane associated protein, signal peptide, transmembrane domain near C
190	cgd6_2970	1.221	1.065	tyrosyl-tRNA synthetase (tyrosyl-tRNA ligase; TyrRS). class-I aaRS
191	cgd8_3520	1.220	1.034	secreted protein with cysteine rich repeats and a mucin like threonine rich repeat, signal peptide
192	cgd7_1550	1.220	0.981	uncharacterized protein
193	cgd3_1450	1.220	1.067	replication factor C like AAA+ ATpase
194	cgd6_4580	1.219	1.084	RNA polymerase III C34 subunit; rpc34p ortholog
195	cgd6_5080	1.219	1.088	GroEL-like chaperone (ATpase); T-complex protein 1
196	cgd7_1540	1.219	1.036	aspartate--tRNA ligase
197	cgd6_3810	1.219	1.075	NUDIX domain protein; mRNA cleavage factor-like protein Im like, plant+animal group
198	cgd6_1400	1.219	1.013	hypothetical protein
199	cgd4_2270	1.218	1.141	oxidoreductase, short-chain dehydrogenase family, putative
200	cgd2_1960	1.218	1.119	mitogen-activated protein kinase 1, serine/threonine protein kinase, putative
201	cgd2_3060	1.218	0.989	hypothetical protein
202	cgd2_470	1.217	0.946	very low complexity large protein, possible unreal ORF?
203	cgd8_2730	1.217	0.934	multidomain chromatinic protein with the following architecture: 3x PHD-bromo-3xPHD-SET domain and associated cysteine cluster at the C-terminus
204	cgd7_1780	1.217	1.151	tRNA exportin type nuclear export protein
205	cgd5_3300	1.217	1.078	large protein with 4 PHD domains
206	cgd6_2150	1.217	1.003	U1 snrnp, putative
207	cgd8_4220	1.216	1.076	conserved hypothetical protein
208	cgd4_3090	1.215	1.030	oocyst wall protein 6
209	cgd7_4470	1.215	1.127	CDC14 phosphatase
210	cgd1_1700	1.214	1.090	GATA type DNA binding domain fused to an AT hook
211	cgd5_1460	1.213	1.126	hypothetical protein with signal peptide
212	cgd3_2080	1.212	1.069	large protein with 8 or more transmembrane domains within NH2 region
213	cgd6_3190	1.212	1.069	60S ribosomal protein-like, putative
214	cgd1_1280	1.211	1.035	domain KOG1676, K-homology type RNA binding proteins
215	cgd7_2660	1.211	1.100	RAD24/Rf-C activator 1 AAA+ ATpase
216	cgd3_3870	1.209	1.036	conserved hypothetical protein
217	cgd2_3640	1.208	1.075	hypothetical protein with region conserved in apicomplexa
218	cgd7_4030	1.208	1.026	hypothetical low complexity protein

219	cgd3_360	1.208	1.124	hypothetical protein
220	cgd4_560	1.207	0.933	hypothetical protein
221	cgd4_3890	1.207	0.917	hypothetical protein
222	cgd3_1040	1.207	1.164	hypothetical protein
223	cgd6_3580	1.207	1.064	hypothetical protein with 3 or more transmembrane domains
224	cgd5_320	1.207	0.949	carboxylesterase , lysophospholipase, signal peptide
225	cgd2_3100	1.207	1.137	hypothetical protein, 8 transmembrane domains
226	cgd6_2360	1.206	0.991	mitochondrial FAD carrier protein
227	cgd4_3810	1.205	1.064	putative transcription factor IIE alpha subunit
228	cgd3_1810	1.204	1.043	casein kinase I
229	cgd6_5410	1.204	0.989	signal peptide plus thr stretch, charged repeats, likely mucin
230	cgd7_4590	1.204	1.074	Mth1. SpoU superfamily - SPOUT methylase
231	cgd3_1520	1.203	1.007	hypothetical protein with transmembrane or GPI anchor sequence at carboxy terminus
232	cgd2_2710	1.202	1.081	TrmA RNA methylase
233	cgd7_4090	1.202	1.079	alpha superhelical protein, divergent TPR like repeats, possible microtubule associated protein
234	cgd1_2610	1.201	0.953	hypothetical protein
235	cgd7_4790	1.201	1.068	Ptc7p phosphatase (PP2C family)
236	cgd1_1570	1.201	1.088	transcription factor E2F

Cluster 3 **0.5 h: Up-regulated (Ratio > 1.2) vs. 5 h: Down-regulated (Ratio < 0.8)**

No.	GeneID	Signal Ratio (0.5 h)	Signal Ratio (5 h)	Description
1	cgd6_4390	1.419	0.772	hypothetical low complexity protein
2	cgd6_4240	1.417	0.769	MO25 protein
3	cgd8_2420	1.338	0.797	transcription initiation factor IIF/ Rap30 like winged HTH
4	cgd3_1860	1.326	0.791	extracellular protein with a signal peptide and 9 EGF domains
5	cgd6_50	1.306	0.682	hypothetical protein
6	cgd3_60	1.238	0.742	hypothetical protein

Cluster 4 **0.5 h: Normal (Ratio = 0.8-1.2) vs. 5 h: Up-regulated (Ratio > 1.2)**

No.	GeneID	Signal Ratio (0.5 h)	Signal Ratio (5 h)	Description
1	cgd2_300	1.199	1.210	kelch repeat-containing proteins that is fused to a HSP90-like ATpase
2	cgd5_1950	1.189	1.214	hypothetical protein with 5 transmembrane domains
3	cgd4_3990	1.182	1.207	hypothetical protein with possible transmembrane domain near N-terminus
4	cgd7_930	1.182	1.233	hypothetical transmembrane protein
5	cgd7_3830	1.173	1.243	conserved hypothetical protein
6	cgd4_2010	1.168	1.258	conserved hypothetical protein
7	cgd3_2630	1.159	1.400	diacylglycerol kinase
8	cgd8_5190	1.154	1.615	large protein with central conserved domain, possible BCRT domain
9	cgd1_1240	1.147	1.263	Sec14 domain containing protein
10	cgd7_3200	1.132	1.278	DHHC family palmitoyltransferase, 4 transmembrane domains plus possible signal peptide
11	cgd8_740	1.132	1.261	prefoldin like molecular chaperone
12	cgd7_2510	1.131	1.217	conserved hypothetical protein
13	cgd7_4980	1.126	1.230	large uncharacterized low complexity protein
14	cgd6_2560	1.122	1.232	hypothetical protein
15	cgd6_3270	1.122	1.218	26S proteasome-associated Mov34/MPN/PAD-1 family. JAB domain.
16	cgd2_2610	1.119	1.331	conserved hypothetical protein
17	cgd7_3470	1.112	1.275	large low comlexity protein, potential zinc ribbon
18	cgd7_1470	1.110	1.202	hypothetical protein
19	cgd6_4790	1.104	1.259	predicted degraded Zn-finger +bbox Zn finger protein
20	cgd7_2960	1.103	1.376	hypothetical protein
21	cgd4_1900	1.097	1.682	hypothetical protein
22	cgd1_2790	1.094	1.498	hypothetical protein
23	cgd5_470	1.085	1.325	hypothetical protein
24	cgd2_2630	1.083	1.260	hypothetical protein, signal peptide plus 11 transmembrane domains
25	cgd3_2730	1.077	1.438	protein with 10 transmembrane domains, possible calcium transporting ATpase or aminophospholipid transporter
26	cgd1_1500	1.075	1.299	hypothetical protein
27	cgd7_520	1.074	1.212	cyclin'cyclophilin like peptidyl-prolyl cis-trans isomerase fused to WD40 repeats at the N-terminus'
28	cgd8_2720	1.069	1.364	insulinase like metalloprotease
29	cgd6_3690	1.067	1.245	hypothetical, PF13_0020_pfal-like; PUG (domain in protein kinases, N-glycanases and other nuclear proteins) + UBX (domain present in ubiquitin-regulatory proteins)
30	cgd1_2620	1.059	1.205	calcium antiporter, Na/Ca exchange associated membrane protein with 11 transmembrane domains
31	cgd6_250	1.058	1.394	hypothetical protein
32	cgd1_3050	1.058	1.249	cation diffusion facilitator like membrane protein transporter, 6x transmembrane domains
33	cgd7_2980	1.057	1.968	Low complexity protein witha potential C2C2 zinc ribbon

34	cgd6_3840	1.056	1.236	SET domain containing protein
35	cgd2_1200	1.050	1.340	steroid reductase like intregral membrane protein with 4x transmembrane domains and an ubiquitin domain at its N-terminus
36	cgd5_520	1.049	1.582	MJ1157-like thiouridine synthase (Pploop atpase) plus Zn ribbon. involved in RNA metabolism.
37	cgd2_3730	1.044	1.206	conserved hypothetical protein
38	cgd3_1620	1.042	1.243	conserved hypothetical protein
39	cgd8_2360	1.040	1.281	hypothetical protein
40	cgd2_1040	1.032	1.831	flavohemoprotein b5+b5R (DJ676J13.1) (flavohemoprotein b5+b5R cytochrome b-type NAD(P)H oxidoreductase), putative
41	cgd1_1230	1.027	1.237	predicted secreted protein, signal peptide
42	cgd4_570	1.027	1.223	ubiquitin-conjugating enzyme
43	cgd1_3120	1.023	1.222	peptide synthase like condensation domain
44	cgd8_3040	1.022	1.204	predicted membrane associated protein, transmembrane domain near C-terminus
45	cgd8_10	1.018	1.236	uncharacterized protein
46	cgd6_1560	1.014	1.255	Yju2p / cwf16-like; Zn finger
47	cgd6_920	1.013	1.298	26s protease regulatory subunit 8, putative
48	cgd1_2640	1.007	1.293	hypothetical protein with RING-H2 like RING domain
49	cgd5_1020	1.004	1.428	apicomplexan conserved protein with 9 transmembrane domain
50	cgd6_4330	1.004	1.372	phosphoglycerate mutase family
51	cgd1_2070	0.997	1.368	hypothetical protein
52	cgd2_1650	0.996	1.328	dolichyl-diphosphooligosaccharide-protein glycosyltransferase beta subunit (Wbp1p)
53	cgd2_1920	0.990	1.412	hypothetical protein
54	cgd1_1150	0.988	1.359	hypothetical protein
55	cgd1_2910	0.980	1.660	conserved hypothetical protein
56	cgd5_3430	0.979	1.244	DNAj protein with possible transmembrane domain within C-terminal region
57	cgd1_2120	0.976	1.249	protein phosphatase regulator like heat repeats
58	cgd4_400	0.972	1.384	protein with possible 2 TPR domains
59	cgd2_670	0.972	1.298	hypothetical protein, 6 transmembrane domains
60	cgd5_570	0.967	1.327	hypothetical protein, 5 transmembrane domains near N-terminus
61	cgd3_3710	0.961	1.699	hypothetical protein
62	cgd1_1650	0.953	1.278	hypothetical protein
63	cgd5_3600	0.952	1.463	predicted secreted protein
64	cgd3_2050	0.951	1.508	putative prolyl 4-hydroxylase alpha subunit homolog, 2-oxoglutarate-dependent dioxygenase
65	cgd8_2390	0.947	1.310	diacylglycerol kinase
66	cgd4_3900	0.945	1.664	hypothetical protein
67	cgd7_3690	0.945	1.415	crooked neck protein HAT repeats
68	cgd7_2010	0.943	1.264	hypothetical membrane protein
69	cgd6_5040	0.942	1.360	transcription factor, putative
70	cgd1_2110	0.940	1.347	protein kinase with WD40 repeat at C-terminus
71	cgd6_4180	0.935	1.241	ubiquitin-conjugating enzyme
72	cgd2_1810	0.934	1.253	CysQ, sulfite synthesis pathway protein, putative
73	cgd4_2680	0.933	1.208	N-methyl-D-aspartate receptor-associated protein, 7 transmembrane domain protein
74	cgd4_3450	0.932	1.202	large low complexity protein with signal peptide, within large locus of signal peptide containing proteins
75	cgd5_1930	0.930	1.287	hypothetical protein
76	cgd1_2980	0.918	1.210	14-3-3 protein
77	cgd1_3150	0.916	1.334	cyclin'cyclin'
78	cgd7_1250	0.912	1.248	hypothetical protein
79	cgd5_3880	0.912	1.210	hypothetical protein
80	cgd2_3940	0.911	1.590	predicted fatty acid binding protein
81	cgd2_3160	0.910	1.262	hypothetical protein
82	cgd5_4500	0.907	1.325	possible vacuolar protein sorting associated protein (VPS)
83	cgd8_1730	0.902	1.268	Uba3p like ubiquitin activating enzyme E1
84	cgd4_3720	0.900	1.495	possible 2 TPR domains at N-terminus
85	cgd1_770	0.900	1.364	hypothetical protein, possible apicomplexan-conserved
86	cgd7_3900	0.898	1.294	hypothetical protein
87	cgd4_1750	0.885	1.238	hypothetical protein
88	cgd7_40	0.880	1.222	calcium/calmodulin-dependent protein kinase with a kinase domain and 2 calmodulin-like EF hands
89	cgd6_1090	0.871	1.270	DnaJ(hsp40)'DnaJ(hsp40)'
90	cgd8_3020	0.870	1.260	hypothetical protein
91	cgd3_2820	0.861	1.200	conserved hypothetical protein
92	cgd2_930	0.858	1.524	peptidase'insulinase-like peptidase'
93	cgd2_3170	0.850	1.258	tbc domain-containing protein
94	cgd3_4330	0.844	1.645	Dbr1p-like RNA lariat debranching enzyme
95	cgd2_2490	0.839	1.262	hypothetical protein, possible transmembrane domain at C-terminus
96	cgd5_2360	0.838	1.270	p27 like 26S proteasomal subunit with a PDZ domain
97	cgd8_2540	0.837	1.218	apicomplexan specific coiled coil protein
98	cgd8_90	0.835	1.292	hypothetical protein

99	cgd7_720	0.834	1.266	PASILLA splice variant 3-like 2KH domains, transmembrane domain at C-terminus
100	cgd4_550	0.816	1.372	hypothetical protein

Cluster 5		0.5 h: Normal (Ratio = 0.8-1.2) vs. 5 h: Normal (Ratio = 0.8-1.2)		
**No.**	**GeneID**	**Signal Ratio (0.5 h)**	**Signal Ratio (5 h)**	**Description**
1	cgd2_220	1.200	1.049	transcription factor TFIIF
2	cgd7_4310	1.199	1.041	extracellular protein with a signal peptide and 4 SCP domains
3	cgd8_2180	1.199	0.921	protein kinase
4	cgd8_2210	1.199	0.812	RCC1 like beta propeller protein
5	cgd1_3400	1.199	1.072	hypothetical protein
6	cgd5_3690	1.198	0.938	protein with 8 transmembrane domains
7	cgd1_3020	1.197	0.995	fructose-1,6-bisphosphate aldolase
8	cgd8_3720	1.196	1.103	D441/Mpe1p like protein with a zinc knuckle (C2HC) and PhD Zn finger domain
9	cgd6_1760	1.196	0.824	phosphatidylinositol 3-kinase; PI3ka plus PI3Kc domains
10	cgd5_3370	1.196	1.008	hypothetical protein
11	cgd3_3880	1.196	1.133	Snu114p GTpase, U5 snRNP-specific protein, 116 kDa
12	cgd8_1850	1.196	1.004	E2F like domain containing transcription factor with the wHTH fold
13	cgd2_1390	1.195	1.133	hypothetical protein
14	cgd1_3510	1.193	0.972	TSP1 domain-containing protein TSP3 precursor
15	cgd3_2710	1.193	0.977	hypothetical protein
16	cgd5_1920	1.192	1.071	pleckstrin homology (PH) domain containing protein
17	cgd2_1860	1.192	0.992	TBC domain containing protein
18	cgd2_2690	1.192	1.049	hypothetical protein
19	cgd6_120	1.191	0.998	disulfide-isomerase, signal peptide plus ER retention motif, putative ER protein
20	cgd4_3010	1.190	1.075	hypothetical protein
21	cgd2_750	1.190	1.184	bub3'bub3-like protein with WD40 repeats'
22	cgd2_2960	1.190	1.109	phosphoprotein phosphatase related, putative
23	cgd5_4390	1.190	1.047	Ser/Thr protein kinase
24	cgd4_3080	1.189	1.024	ribosomal protein S27a, ubiquitin plus zincribbon, UB13p
25	cgd6_3960	1.189	1.079	elongation factor-like protein
26	cgd4_3360	1.189	1.071	WD repeat protein
27	cgd1_1270	1.189	1.004	hypothetical protein with PUG domain fused to a UBA domain and a predicted protease at the N-terminus
28	cgd6_1590	1.188	1.055	eukaryotic translation initiation factor 4 gamma; Nic domain containing protein
29	cgd4_590	1.188	1.002	conserved hypothetical protein
30	cgd8_4060	1.188	0.880	26S proteasomal subunit S3; PINT domain containing protein
31	cgd4_2550	1.186	0.935	putative farnesyl pyrophosphate synthase
32	cgd7_30	1.184	1.030	large uncharacterized protein
33	cgd8_4730	1.184	1.080	DHHC family palmitoyl transferase with a signal peptide and 4 transmembrane domains
34	cgd5_4150	1.184	1.135	hypothetical protein
35	cgd4_210	1.183	0.883	Ubc1p like ubiquitin-conjugating enzyme E2 fused to a UBA domain (UBC+UBA)
36	cgd1_1560	1.183	1.052	domain KOG2577, transcription factor E2F/dimerization partner (TDP)
37	cgd8_1660	1.183	1.027	protein kinase
38	cgd7_5280	1.183	1.091	structure-specific recognition protein 1 (SSRP1) (recombination signal sequence recognition protein)
39	cgd1_1310	1.182	1.083	cleavage stimulation factor subunit 2 that has a CSF1 like RRM domain
40	cgd3_490	1.182	1.125	putative fucose translocator with 8 transmembrane domains, within locus of 3 paralogous genes
41	cgd2_310	1.182	1.162	nucleoside transporter, 10 transmembrane domain domain, nucleoside binding domain
42	cgd1_470	1.182	0.949	predicted secreted protein, signal peptide, low complexity serine-threonine rich, possible mucin
43	cgd6_2650	1.182	0.956	heat shock protein, putative
44	cgd1_1790	1.181	0.876	RING-H2 finger containing membrane associated protein, 8x transmembrane domain
45	cgd2_4070	1.181	0.861	hypothetical protein
46	cgd7_4910	1.180	1.085	conserved 3 transmembrane domain membrane associated RING finger domain (shared by plants and apicomplexans)
47	cgd4_1700	1.180	1.035	GNog1p. GTpase
48	cgd7_5480	1.180	1.029	very large membrane protein
49	cgd4_3320	1.179	1.140	Noc3 like protein involved in nuclear export of pre-ribosomes
50	cgd8_2500	1.179	1.119	hypothetical protein, signal peptide, membrane protein, transmembrane domain at C-terminus, paralogs
51	cgd3_2670	1.179	1.073	protein with UBC domain, ubiquitin conjugating enzyme E2
52	cgd3_3040	1.178	1.049	protein kinase, cAMP-dependent, catalytic chain
53	cgd2_1780	1.178	0.903	PDI like thioredoxin domain containing protein
54	cgd3_1600	1.178	0.890	hypothetical protein with 4 transmembrane domains
55	cgd3_3670	1.177	1.056	putative protein kinase CK2 regulatory subunit CK2B1
56	cgd7_2590	1.177	1.012	integral membrane protein with 10 transmembrane domains
57	cgd4_3300	1.177	1.012	articulin family protein, adjacent paralogous gene
58	cgd2_2750	1.176	0.968	SMC1 structural maintenance of chromosomes 1

59	cgd7_1760	1.176	1.141	P-type ATpase (calcium/phospholipid-transporter), 9 transmembrane domains
60	cgd4_2300	1.176	1.149	ubiquitin-activating enzyme E1 (UBA)
61	cgd2_1150	1.176	1.049	hypothetical protein
62	cgd5_2210	1.176	1.102	40S ribosomal protein S13
63	cgd8_2670	1.175	1.044	secreted glucose methanol choline like oxidoreductase of the FAD dependent oxidoreductase like fold, signal peptide
64	cgd6_2040	1.174	1.016	oligosaccharyl transferase STT3 protein
65	cgd5_1290	1.174	1.082	membrane associated adenyl cyclase with 2 transmembrane regions and an adenylyl cyclase domain
66	cgd5_2130	1.173	0.969	chromatin associated proein with a chromodomain at the C-terminus
67	cgd7_2950	1.173	1.112	phospholipid cytidyltransferase HIGH family
68	cgd6_860	1.173	0.983	hypothetical protein
69	cgd6_2600	1.173	1.009	hypothetical protein
70	cgd4_4340	1.172	1.083	diacylglycerol kinase
71	cgd3_290	1.172	0.842	hypothetical protein
72	cgd6_1900	1.172	1.120	kinesin heavy chain, putative
73	cgd8_2770	1.172	0.966	SNF2L ortholog with a SWI/SNF2 like ATpase and a Myb domain
74	cgd6_4660	1.171	1.028	possible apicomplexan-specific protein
75	cgd3_1260	1.171	1.178	RING domain protein
76	cgd1_2490	1.171	1.021	proteasome subunit beta type 1, putative
77	cgd6_2570	1.171	1.032	MORN domain repeat containing protein
78	cgd8_240	1.169	1.169	hypothetical protein with 6 transmembrane domains
79	cgd7_3810	1.169	1.033	conserved hypothetical protein
80	cgd4_3880	1.169	0.988	emap RNA binding domain protein (N terminal low complexity region)
81	cgd6_5180	1.168	1.047	hypothetical protein having a transmembrane domain within N-terminus
82	cgd6_5260	1.167	0.923	ABC transporter ATpase with 2 AAA domains
83	cgd1_1760	1.167	1.095	Cdc50p like membrane protein, 2x transmembrane domains
84	cgd2_1350	1.167	1.101	26S proteasome regulatory subunit, S6a like AAA ATpase
85	cgd6_2440	1.166	0.968	hypothetical protein having 6 transmembrane domains
86	cgd2_2680	1.165	1.114	possible MgtA, cation transport ATpase, signal peptide, 12 transmembrane domains
87	cgd1_2260	1.165	1.073	RNA polymerase II B8 subunit
88	cgd1_440	1.165	1.047	hypothetical protein
89	cgd3_2690	1.165	0.937	DNAj domain, possible transmembrane domain
90	cgd2_3020	1.164	1.053	hypothetical protein
91	cgd3_250	1.164	0.992	protein serine/threonine phosphatase alpha
92	cgd6_630	1.164	1.028	conserved hypothetical protein
93	cgd5_1610	1.163	1.113	hypothetical protein, possibly apicomplexan conserved
94	cgd6_2720	1.163	0.994	hypothetical protein having a signal peptide and transmembrane domain within C- terminus
95	cgd2_3340	1.163	0.992	Ser/Thr protein kinase
96	cgd4_2990	1.162	1.140	glycosyl transferase
97	cgd6_1110	1.162	1.062	signal peptide-containing protein
98	cgd8_2380	1.162	1.030	CDC45 like protein, possible horizontal transfer
99	cgd3_1640	1.161	1.156	hypothetical protein with 7 transmembrane domains
100	cgd3_680	1.160	0.959	cathepsin like thiol protease possibly membrane associated, putative
101	cgd6_3510	1.159	1.158	hypothetical protein
102	cgd7_320	1.159	1.045	putative 60S ribosomal protein L18A
103	cgd7_5010	1.159	1.031	coatomer complex beta
104	cgd5_3480	1.159	0.972	hypothetical protein with possible conserved HGG motif
105	cgd4_2560	1.159	0.958	phospholipase C, delta 1 ortholog with 2 EF hands plus phospholipase C domain plus C2 domain
106	cgd3_4100	1.159	0.962	conserved protein, COG SSU ribosomal protein S8E
107	cgd3_3800	1.158	1.000	hypothetical protein
108	cgd6_70	1.158	1.050	conserved hypothetical protein
109	cgd6_5310	1.158	1.079	hypothetical protein with signal peptide
110	cgd6_3830	1.158	1.018	rab1a protein, putative
111	cgd1_1830	1.158	0.885	TB2/DP1/HVA22 family integral membrane protein that may be involved in membrane trafficking, 3x transmembrane domains
112	cgd7_2170	1.158	1.091	hypothetical membrane protein
113	cgd3_1650	1.158	1.144	eIF2G GTpase. eukaryotic translation initiation factor 2 gamma subunit (ZnR+GTpase)
114	cgd7_310	1.157	0.905	conserved hypothetical protein
115	cgd7_3290	1.157	1.132	cleft lip and palate family of eukaryotic membrane proteins (potential transporters) with 9 transmembrane domains
116	cgd1_230	1.157	1.140	ALG-2 like alpha-1,3 mannosyltransferase
117	cgd6_840	1.156	0.908	shanti/Ykl100cp/Minor histocompatibility antigen H13-like; presenilin, signal peptide peptidase family, with 10 transmembrane domains and a signal peptide
118	cgd7_2090	1.156	1.107	Ruv DNA-helicase-related protein
119	cgd7_5050	1.155	1.036	NIMA-related kinase 5
120	cgd7_1570	1.155	0.944	hypothetical protein
121	cgd8_4640	1.155	1.130	WD40 repeat

122	cgd6_3290	1.155	1.058	DNA-directed RNA polymerase III C1 subunit
123	cgd4_2360	1.155	1.045	DSK2 like protein with a ubiquitin domain, 2 STI1 motifs and a UBA domain at its C-terminus
124	cgd7_3600	1.154	0.803	Hs17p, histone methylase
125	cgd8_4620	1.154	0.922	ISWI related chromatinic protein with an apicomplexan specific domain architecture composed of 3x PHD+SNF2 ATpase+2xPHD
126	cgd8_150	1.154	1.166	CpTSP4; extracellular protien with signal peptide and 2 repeats of an apple domain followed by a TSP1 domain
127	cgd3_1550	1.153	1.020	sec14 domain containing protein
128	cgd8_890	1.153	0.819	CobW/nitrile hydratase activator like P-loop ATpase
129	cgd2_2480	1.152	1.100	methionine aminopeptidase, type II, putative , an1 domain
130	cgd5_3830	1.151	0.962	hypothetical protein
131	cgd4_4020	1.150	1.093	40S ribosomal protein S2/S5. DSRBD RNA binding domain
132	cgd1_880	1.150	1.049	eukaryotic initiation factor 4A (eIF4A) (eIF-4A)
133	cgd8_4710	1.150	1.122	hypothetical protein
134	cgd5_2340	1.150	0.999	possible SET domain containing protein
135	cgd7_4100	1.150	1.049	Ssm4 ring finger fused to a forkhead associated (FHA) domain (apicomplexan specific architecture)
136	cgd3_2090	1.150	1.029	40S ribosomal protein SAe
137	cgd8_3740	1.150	0.990	Yer119cp like amino acid transporter, 11 transmembrane domain
138	cgd8_3160	1.150	0.866	hypothetical protein
139	cgd8_5300	1.150	0.978	signal peptide containing protein
140	cgd2_4180	1.149	0.980	hypothetical protein
141	cgd1_2760	1.148	1.029	yyaF/YCHF TRANSFAC/OBG family small GTpase plus RNA binding domain TGS
142	cgd2_1020	1.148	1.038	proteasome regulatory subunit Rpn9, PINT domain
143	cgd1_730	1.148	0.894	glutamine cyclotransferase, predicted bacterial/plant origin
144	cgd8_4720	1.147	1.029	seryl-tRNA synthetase, cytoplasmic
145	cgd6_200	1.147	1.124	oocyst wall protein 8
146	cgd7_1170	1.146	0.984	protein containing ringfinger+4xC2H2+littlefinger domains
147	cgd7_2850	1.145	1.197	cathepsin like thiol protease possibly membrane associated
148	cgd7_3640	1.145	1.035	YjeF family of predicted nucleotide binding proteins
149	cgd2_2880	1.144	0.830	WD-40 repeat protein, putative
150	cgd4_2120	1.143	0.938	PX domain containing protein
151	cgd6_3570	1.143	0.804	myotubularin related protein 1, C-terminus protein tyrosine/serine phosphatase, catalytic domain
152	cgd3_2530	1.143	1.051	PUP1/proteasome subunit beta type 7, NTN hydrolase fold
153	cgd5_1540	1.143	1.007	conserved hypothetical protein
154	cgd2_3710	1.143	1.136	Pfa MAL6P1.309 like protein
155	cgd8_1650	1.142	1.000	partner of Nob1; Pno1p; Yor145cp like KH domain containing protein
156	cgd8_3640	1.141	1.105	niemann-Pick type C1 disease protein/ patched like cholesterol transporter of the SecD family, 12 transmembrane domain
157	cgd6_4920	1.141	1.079	hypothetical protein with 4 transmembrane domains
158	cgd4_2370	1.141	0.968	lysyl-tRNA synthetase (NOB+tRNA synthetase)
159	cgd1_850	1.141	1.155	40S ribosomal protein S19
160	cgd8_2470	1.140	1.092	hypothetical protein, signal peptide, 4 transmembrane domains
161	cgd3_2410	1.140	1.182	ubiquitin-fusion degadation-2 (UFD2) family protein with a UBOX at the C-terminus
162	cgd2_980	1.140	1.149	putative DNA-directed RNA polymerase 2
163	cgd7_5090	1.140	1.100	SET domain protein with MYND insert (Skm/BOP family)
164	cgd3_600	1.140	0.871	hypothetical protein, signal peptide
165	cgd5_1600	1.139	1.157	translation initiation factor if-2B beta; eIF-2
166	cgd7_3130	1.139	0.988	ER vesicle protein; Erv41p, transmembrane region near C terminus and possible N region transmembrane
167	cgd4_3210	1.139	1.160	hypothetical protein
168	cgd6_4500	1.139	1.148	hypothetical low complexity multi-pass transmembrane protein
169	cgd3_3020	1.139	1.055	possible guaning nucleotide exchange factor, eIF-2B
170	cgd8_3730	1.138	1.012	conserved hypothetical protein
171	cgd5_2440	1.137	1.091	Ylr401cp-like protein with 2 CCCH domains plus Dus1p tRNA dihydrouridine synthase Tim barrel
172	cgd7_5330	1.137	1.182	thioredoxin/PDI, cyanobacterial type, signal peptide plus 4 transmembrane domains
173	cgd5_3740	1.137	0.956	40S ribosomal protein S12. pelota RNA binding domain containing protein
174	cgd6_570	1.136	1.067	60S ribosomal protein L23
175	cgd7_1960	1.136	0.971	membrane protein with multiple cysteines at the N-terminus.
176	cgd2_4380	1.136	1.018	signal peptide, repeats, gene anchored to telomere
177	cgd1_3640	1.136	1.032	hypothetical protein, signal peptide
178	cgd8_4660	1.135	1.022	hypothetical protein, signal peptide
179	cgd8_3690	1.135	1.074	mitosis protein DIM1
180	cgd1_1980	1.135	1.070	protein geranyl-geranyltransferase beta subunit
181	cgd5_1200	1.135	1.017	ring finger and bbox containing protein, putative
182	cgd2_3410	1.134	1.095	ARF1/2 like small GTpase, putative
183	cgd3_3050	1.134	1.087	hypothetical protein
184	cgd4_4330	1.133	0.951	hypothetical protein

185	cgd1_3390	1.133	1.192	katanin p60/fidgetin family AAA ATpase
186	cgd7_5250	1.133	1.092	methyltransfer with N-terminal ankyrin repeats
187	cgd4_740	1.133	1.080	thioredoxin peroxidase-like protein
188	cgd6_3640	1.133	1.048	protein disulfide isomerase ; secreted protein with thioredoxin, signal peptide plus ER retention motif
189	cgd7_3010	1.132	1.142	cysteine-rich protein
190	cgd1_2270	1.132	1.021	40S ribosomal protein S26
191	cgd8_220	1.132	1.070	ubiquitin-conjugating enzyme e2
192	cgd6_5350	1.132	0.921	putative ABC transporter protein
193	cgd4_2520	1.132	1.042	hypothetical protein with a signal peptide, gene within locus of signal-peptide containing hypothetical proteins
194	cgd4_1950	1.132	0.977	RPR46-like RNAse PH domain
195	cgd1_1670	1.131	1.121	bystin/ S.cerevisiae En1p like adaptor domain
196	cgd5_3630	1.131	0.881	thymidylate kinase
197	cgd7_5180	1.130	1.153	eIF3 gamma/P40 with JAB/PAD domains; translation initiation factor IF-3 subunit 3
198	cgd5_1890	1.130	1.110	hypothetical protein
199	cgd3_4250	1.130	1.173	secreted insulinase like peptidase, signal peptide
200	cgd4_2070	1.129	0.922	cyclin B like
201	cgd2_940	1.129	0.985	signal peptide plus transmembrane domain or GPI anchor
202	cgd8_440	1.129	1.071	60S ribosomal protein L5
203	cgd1_2780	1.129	1.068	hypothetical protein, 4 transmembrane domains
204	cgd2_2570	1.127	1.157	hypothetical protein, possible signal peptide
205	cgd2_2350	1.127	0.998	MA3 domain containing protein
206	cgd8_3060	1.127	1.072	adaptin AP complex subunit alpha
207	cgd7_2860	1.126	1.119	uncharacterized protein with predicted coiled coil regions
208	cgd2_380	1.125	0.851	COG0237: dephospho-CoA kinase
209	cgd2_2940	1.125	0.959	conserved hypothetical protein
210	cgd7_3720	1.125	0.985	RNA polymerase beta subunit
211	cgd1_980	1.125	1.074	RNase L inhibitor-like protein
212	cgd4_3030	1.125	1.062	hypothetical protein with a signal peptide
213	cgd6_2870	1.122	1.068	hypothetical protein
214	cgd1_2130	1.121	0.960	glutaminyl-tRNA synthetase, of predicted bacterial origin
215	cgd8_200	1.121	0.853	hypothetical protein
216	cgd8_3840	1.121	1.085	hypothetical protein
217	cgd3_4270	1.120	1.056	peptidase'insulinase like peptidase'
218	cgd6_1990	1.120	0.991	hypothetical protein with signal peptide
219	cgd7_1450	1.120	1.062	hypothetical protein
220	cgd1_3030	1.120	1.071	hypothetical protein containing WD40 repeats fused to a RING finger
221	cgd5_1190	1.119	0.956	nucleolar protein NOP4; rrm domain containing protein
222	cgd1_2570	1.119	0.925	possible conserved eukaryotic alpha beta hydrolase
223	cgd4_460	1.119	0.976	hypothetical protein
224	cgd8_5070	1.119	1.199	hypothetical protein
225	cgd2_900	1.118	1.054	putative T complex chaperonin
226	cgd8_4160	1.118	0.970	hypothetical protein, transmembrane domain
227	cgd5_40	1.118	0.871	signal peptide containing large protein with proline stretches
228	cgd4_1970	1.118	1.196	protein with central transmembrane domain followed by gly-met-pro repeat
229	cgd1_1450	1.117	1.150	predicted secreted protein, signal peptide
230	cgd7_5410	1.117	0.930	WD40 protein
231	cgd3_3750	1.117	1.023	multiprotein bridging factor type 1 like transcriptional co-activator
232	cgd5_2010	1.116	0.996	nuclear VCP like protein with 2 AAA ATpase domains
233	cgd8_4420	1.115	1.086	WD40 repeat and RING finger domain-containing protein
234	cgd1_910	1.115	1.078	ZnF A20 and Znf AN1 domains, involved in signaling
235	cgd3_3100	1.115	1.125	12 transmembrane domain protein MFS family sugar transporter
236	cgd7_5360	1.115	1.142	uncharacterized protein
237	cgd1_3860	1.115	1.041	hypothetical protein
238	cgd2_1740	1.114	1.090	RSC8 ortholog with a swirm domain, ZZ finger and Myb
239	cgd5_1080	1.114	1.047	hypothetical protein
240	cgd6_5400	1.114	0.995	protein with signal peptide plus thr stretch, cys rich, possible mucin
241	cgd1_3820	1.114	0.953	hypothetical protein, transmembrane domain or GPI anchor near C-terminus
242	cgd4_1220	1.114	1.150	conserved hypothetical protein
243	cgd7_450	1.113	1.179	putative cytidine diphosphate-diacylglycerol synthase; integral membrane protein with 7 or more transmembrane domains
244	cgd8_1050	1.113	1.118	hypothetical protein
245	cgd8_2000	1.113	1.102	KRE33p like superfamily I ATPAse fused to an acetylase
246	cgd1_1300	1.113	0.978	spindly like TPR repeats, predicted plant origin
247	cgd6_5440	1.112	1.115	hypothetical protein, signal peptide, paralogs
248	cgd4_1940	1.112	1.099	putative nucleoside-diphosphate kinase
249	cgd7_3540	1.112	1.052	conserved hypothetical protein

250	cgd8_2490	1.111	1.044	conserved hypothetical protein
251	cgd1_3420	1.110	0.951	conserved possible MUS81 endonuclease
252	cgd8_1860	1.110	1.070	LisH domain at N + WD40 repeats
253	cgd7_4270	1.110	1.025	glycolytic phosphoglycerate mutase
254	cgd4_2870	1.110	1.120	putative sugar transporter, 12x transmembrane domain protein
255	cgd1_110	1.110	1.051	predicted secreted protein, signal peptide, paralogs
256	cgd8_2980	1.110	0.952	hypothetical protein
257	cgd7_3820	1.110	1.050	conserved hypothetical protein
258	cgd7_5000	1.110	0.961	vacuolar ATP synthase subunit A
259	cgd8_80	1.110	1.182	10 transmembrane domain, possible aa transporter
260	cgd3_4180	1.109	1.080	secreted insulinase like peptidase
261	cgd2_1450	1.109	1.197	major facilitator superfamily protein, putative, 12 transmembrane domain
262	cgd8_350	1.109	1.145	asparaginyl-tRNA synthetase (NOB+tRNA synthase)
263	cgd6_3740	1.109	0.959	41-2 protein antigen precursor
264	cgd4_680	1.109	0.965	hypothetical protein
265	cgd7_5520	1.108	0.931	serine/threonine rich low complexity protein
266	cgd1_1970	1.108	1.180	hypothetical protein
267	cgd8_3890	1.108	1.097	membrane protein with signal peptide plus 12x transmembrane domains
268	cgd6_1520	1.108	0.957	2-methylthioadenine synthetase; MiaB
269	cgd8_3490	1.108	1.066	hypothetical protein
270	cgd3_470	1.107	1.131	hypothetical protein with 10 transmembrane domains
271	cgd8_1600	1.107	0.965	sugar phosphate permease with 11 transmembrane domains
272	cgd3_3780	1.107	0.852	hypothetical protein
273	cgd3_3920	1.106	1.067	DEAD/DEAH box ATP-dependent RNA helicase, putative
274	cgd2_2530	1.106	1.000	hypothetical protein, signal peptide
275	cgd5_2150	1.106	1.185	protein with signal peptide and 8 transmembrane domains; possible ZIP zinc transporter family member
276	cgd6_3990	1.106	1.072	elongation factor 1 alpha
277	cgd7_3250	1.105	1.153	membrane protein with no close homologs,
278	cgd2_3210	1.105	1.188	hypothetical protein
279	cgd8_190	1.104	0.940	Mak5 pre-mRNA splicing RNA SFII helicase
280	cgd5_2200	1.104	1.033	T8M16_190-plant like protein with RRM domain
281	cgd7_4250	1.104	1.070	RNA pol II carboxy terminal domain phosphatase of the HAD superfamily with a BRCT domain at the C-terminus
282	cgd6_390	1.104	0.805	hypothetical protein
283	cgd7_3670	1.103	1.034	heat shock protein 90 (Hsp90), signal peptide plus ER retention motif
284	cgd3_2180	1.103	0.946	type I fatty acid synthase
285	cgd8_3800	1.102	1.097	hypothetical protein
286	cgd8_4630	1.102	1.137	7 pass integral membrane proteinwith FLHWFHH motif shared with fatty-acyl elongase
287	cgd5_2450	1.102	1.188	hypothetical protein
288	cgd7_230	1.102	1.080	RPR45-like archaeo-eukaryotic exosomal RNAse PH
289	cgd4_880	1.101	1.031	ADA2 ortholog with a ZZ finger, SANT domain and a SWIRM domain
290	cgd1_3250	1.101	0.953	signal peptide, possible transmembrane domain near N-terminus, proline rich and His-stretch at C-terminus
291	cgd6_2240	1.100	1.106	protein with N-terminal apicomplexan-specific globular domain plus PHD domain
292	cgd8_1840	1.100	1.131	40S ribosomal subunit protein S9, putative
293	cgd2_1060	1.099	1.101	calcium/calmodulin dependent protein kinase with a kinas domain and 4 calmodulin-like EF hands
294	cgd3_750	1.099	1.009	hypothetical protein
295	cgd5_1560	1.099	0.995	hypothetical protein, 2 transmembrane domains near C-terminus
296	cgd2_1820	1.098	1.040	hypothetical protein
297	cgd8_600	1.098	0.917	secrted alpha/beta hydrolase superfamiy protein
298	cgd8_3830	1.098	1.122	hypothetical protein
299	cgd6_560	1.097	1.151	alanyl-tRNA synthetase (with HxxxH domain)
300	cgd1_1480	1.097	1.142	predicted membrane associated protein, 4 transmembrane domains and signal peptide
301	cgd5_4280	1.097	1.188	hypothetical protein with signal peptide and possible transmembrane region at C-terminus
302	cgd7_5100	1.096	1.190	protein disulfide isomerase, signal peptide plus ER retention motif
303	cgd8_3350	1.096	0.905	oocyst wall protein 4
304	cgd4_3150	1.096	1.055	cullin-like protein of probable plant origin
305	cgd6_2880	1.096	1.169	hypothetical protein
306	cgd8_5200	1.096	1.118	5 transmembrane domain proteiin
307	cgd2_3080	1.095	1.086	CpTSP10 protein
308	cgd8_530	1.095	0.984	hypothetical protein with 4 transmembrane domains
309	cgd4_2910	1.094	1.020	aminopeptidase
310	cgd8_4800	1.094	1.123	SSM4 like ring finger with a forkhead associated (FHA) domain (apicomplexan-specific architecture)
311	cgd2_780	1.093	1.073	hypothetical protein
312	cgd4_3220	1.093	1.116	histone H3
313	cgd7_1510	1.093	0.877	hypothetical protein

314	cgd1_2730	1.092	0.961	Ro ribonucleoprotein-binding protein 1, RNA binding protein with 3x RRM domains
315	cgd7_4080	1.092	1.137	protein disulfide isomerase, signal peptide, ER retention motif
316	cgd6_3130	1.092	0.988	hypothetical protein
317	cgd5_3040	1.091	1.022	40S ribosomal protein S7
318	cgd2_330	1.091	1.069	notchless
319	cgd7_5020	1.090	0.990	ubiqutin family protein
320	cgd7_1370	1.090	1.055	hypothetical protein
321	cgd2_4350	1.090	1.134	putative patched family protein with 12 transmembrane domain
322	cgd7_2250	1.090	1.045	40S ribosomal protein S3, KH domain
323	cgd3_200	1.090	0.972	zfwd1 protein, CCCH like RNA binding domain fused to WD repeats
324	cgd3_2160	1.090	0.804	SprT like metalloprotease
325	cgd2_1030	1.090	1.070	mitochondrial carrier protein, putative
326	cgd2_1850	1.089	1.048	stress-induced protein sti1-like protein, putative
327	cgd3_780	1.089	1.020	hypothetical protein
328	cgd2_3750	1.089	1.067	hypothetical protein, transmembrane domain near C-terminus
329	cgd3_4140	1.089	1.076	P-type ATpase3, 13 transmembrane regions
330	cgd2_290	1.088	1.194	hypothetical protein
331	cgd5_1370	1.088	1.010	hypothetical protein
332	cgd7_90	1.088	1.079	high affinity sulfate transporter-related
333	cgd1_1940	1.088	0.933	possible RAN guanine nucleotide release factor
334	cgd8_2080	1.087	0.928	conserved hypothetical protein
335	cgd8_3000	1.087	1.064	SYG1/ ERD1 like integral membrane protein required for retention of ER lumen proteins, with 8-10 transmembrane domains
336	cgd3_540	1.087	1.108	protein with 12 transmembrane domains
337	cgd5_810	1.086	1.040	leucine-rich repeat protein
338	cgd6_2490	1.086	1.076	e3 ubiquitin-protein ligase, putative
339	cgd6_5270	1.086	0.856	hypothetical protein with possible signal peptide
340	cgd8_930	1.085	1.179	RBX1 ortholog, RING finger
341	cgd6_2380	1.085	0.876	mRNA capping enzyme; RNA guanylyltransferase Ceg1p
342	cgd3_1120	1.085	0.912	SNAPc like transcription factor with 4 MYB/SANT domains
343	cgd7_200	1.085	0.995	hypothetical protein with signal peptide
344	cgd7_60	1.085	1.018	Hat1-like acetyltransferase (histone acetyltransferase-like)
345	cgd1_830	1.085	1.073	sen1p/ NAM7 like superfamily I RNA helicase
346	cgd3_430	1.084	0.940	VPS13p like protein involved in vacuolar sorting
347	cgd7_1920	1.084	1.092	hypothetical protein
348	cgd5_180	1.083	0.838	hypothetical protein with signal peptide
349	cgd3_2360	1.083	1.122	putative nucleoporin, FG-rich motifs within N-terminal region
350	cgd7_3620	1.083	1.097	PP2C phosphatase
351	cgd7_1120	1.083	1.050	3CCCH domain containing protein
352	cgd2_2230	1.082	1.052	conserved hypothetical protein
353	cgd8_3470	1.082	1.052	membrane associated protein with a RING finger, 4xtransmembrane domain
354	cgd5_690	1.082	1.109	extracellular protein with a ricin domain and a family 2 glycosyltransferase domain
355	cgd8_2340	1.082	1.030	cold shock RNA binding domain of the OB fold
356	cgd2_2070	1.082	1.024	translation elongation factor EF-1, subunit alpha, putative
357	cgd7_1280	1.081	0.949	hypothetical protein
358	cgd3_210	1.081	0.968	hypothetical protein
359	cgd7_5190	1.081	1.198	uncharacterized protein with coiled coil regions
360	cgd8_1960	1.081	0.887	hypothetical protein
361	cgd7_1580	1.080	0.825	mbl domain containing protein
362	cgd1_3530	1.080	1.051	Gbp1/Gbp2p-like, putative single stranded G-strand telomeric DNA-binding protein
363	cgd6_580	1.079	1.093	inactive CPSFs Cft2p metallobeta-lactamase
364	cgd5_750	1.079	1.127	Mdh; malate dehydrogenase (oxaloacetate-decarboxylating)(NADP+)
365	cgd7_3020	1.079	1.135	rhomboid family membrane associated protease, 7 transmembrane domain
366	cgd8_1820	1.079	0.953	ATP-dependent RNA helicase, putative
367	cgd5_1850	1.078	1.105	GPI1/PIG-Q like N-acetylglucosaminyl-phosphatidylinositol transferase involved in GIP anchor biosynthesis
368	cgd6_1210	1.078	1.034	hypothetical protein
369	cgd7_3100	1.078	1.099	conserved hypothetical protein
370	cgd7_210	1.078	0.933	fkbp
371	cgd5_2180	1.077	0.873	large low complexity protein with proline/alanine-rich repeat
372	cgd4_3460	1.077	1.063	hypothetical protein with signal peptide, within larege locus of signal peptide containing proteins
373	cgd3_2190	1.077	0.964	ubiquitin domain containing protein with a UBA domain at the C-terminus
374	cgd7_2910	1.076	0.811	Abd1p; RNA (guanine-7-methyltransferase (cap methyltransferase)
375	cgd8_2120	1.076	0.965	hypothetical protein
376	cgd7_120	1.076	1.099	cAMP-dependent protein kinase regulatory subunit
377	cgd2_360	1.076	1.071	prtip-like IF39 eukaryotic translation initiation factor 3
378	cgd4_3570	1.073	1.043	conserved hypothetical protein

379	cgd4_4490	1.073	1.152	conserved hypothetical protein
380	cgd8_3770	1.073	1.069	DNAJ like chaperone
381	cgd8_570	1.072	0.842	proteasome regulatory subunit S2 (RPN1)
382	cgd3_1880	1.072	1.164	protein with 2x rrm domains
383	cgd3_3770	1.072	1.023	Hsp90
384	cgd5_1140	1.072	0.893	cinnamyl-alcohol dehydrogenase-like nucleoside diphosphate sugar epimerase
385	cgd7_110	1.071	1.122	putative sortilin, SMART_VSP10 domain, possible signal peptide
386	cgd5_3170	1.071	0.951	histone H2B
387	cgd7_2800	1.071	0.914	ubiquitin-conjugating enzyme E2, putative
388	cgd4_4410	1.070	0.921	2x PHD domain containing protein
389	cgd4_2020	1.070	1.110	conserved hypothetical protein
390	cgd3_480	1.070	1.120	hypothetical protein with a signal peptide
391	cgd3_2260	1.069	1.048	hypothetical protein
392	cgd2_990	1.069	1.035	queuine tRNA-guanine transglycosylase
393	cgd8_3750	1.068	1.048	predicted secreted protein
394	cgd5_900	1.066	1.116	MRP like MinD family ATpase
395	cgd1_3750	1.065	1.001	conserved hypothetical protein
396	cgd5_1430	1.064	0.832	hypothetical protein
397	cgd3_980	1.064	1.070	hypothetical protein
398	cgd1_2000	1.063	0.995	predicted coiled coil protein
399	cgd7_2920	1.063	1.147	DNA replication licensing factor MCM5 like AAA+ ATpase
400	cgd8_2370	1.063	1.121	adenosine kinase like ribokinase
401	cgd2_1870	1.062	1.084	guanine nucleotide-binding protein, putative
402	cgd8_1770	1.062	0.930	HxYxP motif containing repeats seen only in C. elegans F29G6.3b, signal peptide
403	cgd7_2320	1.062	1.040	thioesterase of the a/b hydrolase superfamily, possible bacterial origin
404	cgd8_3360	1.062	1.147	S. pombe SPAC1687.04 like conserved eukaryotic protein that shares a domain with several MCM7, probably involved in DNA replication
405	cgd7_5170	1.061	1.065	deoxyuridinetriphosphatase, possible bacterial origin
406	cgd7_5270	1.061	1.052	Translation initiation factor 2, alpha subunit(eIF2-alpha); S1-like RNA binding domain
407	cgd4_1930	1.061	0.959	possible origin recognition complex protein subunit 2, orc2
408	cgd3_1750	1.061	0.851	conserved hypothetical protein
409	cgd1_890	1.060	1.047	protein kinase domain
410	cgd7_5490	1.060	1.041	PRP18 (SFM+PRP18 domains)
411	cgd4_1390	1.060	1.136	possible ABC transporter with AAA domain and 12 transmembrane domains
412	cgd8_3380	1.059	1.001	Rrp12p like nucleolar protein
413	cgd2_770	1.059	1.118	hypothetical protein
414	cgd3_1340	1.059	1.076	NMD3p like protein involved in nonsense mediated decay
415	cgd8_5010	1.059	1.120	hypothetical protein
416	cgd4_60	1.059	0.915	extracellular membrane associated protein with a signal peptide, an EGF domain followed by 10 transmembrane domains
417	cgd6_3070	1.059	0.981	hypothetical protein with a signal peptide
418	cgd2_2260	1.059	1.119	zuotin related factor-1 like protein with a DNAJ domain at the N-terminus and 2 SANT domains
419	cgd3_3190	1.058	1.090	GCN5 like acetylase + bromodomain
420	cgd5_2080	1.058	1.004	protein with PHD finger and a nipped-B/Mis4/Scc2 like domain at the C-terminus
421	cgd8_4390	1.058	0.975	hypothetical protein
422	cgd4_1290	1.058	1.081	small hypothetical protein, possible conserved
423	cgd8_900	1.057	1.146	Ydr449cp/Utp6p; small (ribosomal) subunit (SSU) processosome (contains U3 snoRNA). HAT repeats
424	cgd8_3580	1.057	1.098	hypothetical protein, signal peptide, paralogs
425	cgd4_2610	1.057	1.090	hypothetical protein
426	cgd3_3350	1.057	1.099	hypothetical protein
427	cgd2_2600	1.055	1.003	hypothetical protein
428	cgd8_2630	1.055	1.074	APG10/ Aut1p like like autophagocytosis protein involved in vacuolar transport
429	cgd7_2430	1.055	1.013	translation initiation factor eIF-5; Tif5p, ZnR+W2 domains
430	cgd2_1310	1.054	1.115	11 transmembrane domain protein
431	cgd7_5210	1.054	1.090	bromodomain protein
432	cgd3_590	1.054	0.918	hypothetical protein with 4 transmembrane domains, possible unusual phyletic distribution
433	cgd6_1830	1.054	0.899	HT-1080/Cdc123p - like protein
434	cgd6_3400	1.054	1.097	protein kinase
435	cgd3_3440	1.053	0.981	heat shock protein HSP70, mitochondrial
436	cgd7_3790	1.053	1.064	unconventional myosin
437	cgd8_3660	1.053	1.071	RNA 3'-Terminal Phosphate Cyclase-like protein
438	cgd6_2280	1.052	0.960	hypothetical protein with signal peptide and 12 or more transmembrane domains
439	cgd4_510	1.051	0.886	hypothetical protein
440	cgd1_1470	1.050	1.101	hypothetical protein, possible transmembrane domain within N-terminus
441	cgd2_2990	1.050	1.092	60S ribosomal protein L13, putative
442	cgd7_4460	1.049	1.059	60S ribosomal protein L6
443	cgd8_120	1.047	1.030	hypothetical protein

444	cgd7_4620	1.047	1.093	Rf-A (OB fold protein)
445	cgd8_2790	1.045	1.075	pleckstrin homology (PH domain) containing protein
446	cgd7_2280	1.045	1.136	60S ribosomal protein L40
447	cgd3_2320	1.045	1.037	cGMP phosphodiesterase A4
448	cgd7_100	1.045	1.087	ring domain-containing protein
449	cgd5_3470	1.045	0.946	E. coli yfiP family protein
450	cgd5_1780	1.045	1.014	MJ1625/yease Yp1009cp-like HhH domain
451	cgd8_140	1.044	0.925	hypothetical protein
452	cgd4_1990	1.044	1.181	RRp1-like protein
453	cgd3_4150	1.042	1.094	cutinase negative acting protein, putative
454	cgd4_440	1.041	0.868	XPG, DNA excision repair protein, flap endonuclease
455	cgd3_1940	1.040	1.100	conserved hypothetical protein
456	cgd4_860	1.039	1.145	possible WWE domain
457	cgd5_1620	1.037	1.121	hypothetical protein with short conserved N-terminal motif, possible cysteine binding domain
458	cgd6_150	1.036	1.137	synaptobrevin'synaptobrevin, adjacent duplicated gene'
459	cgd3_760	1.036	0.877	HMG-like nuclear protein, Nhp2p, pelota RNA binding domain containing protein
460	cgd7_600	1.036	1.179	hypothetical protein
461	cgd1_2530	1.036	0.991	TRAFAC type P-loop GTpase that may be related to kinesin
462	cgd3_2140	1.036	0.814	hypothetical protein
463	cgd4_890	1.034	1.105	hypothetical protein
464	cgd6_4110	1.034	0.925	hypothetical protein
465	cgd2_1840	1.033	1.082	signal peptide
466	cgd8_3240	1.033	1.044	apicomplexan specific protein
467	cgd6_5010	1.033	0.865	hypothetical protein
468	cgd3_3540	1.032	0.999	hypothetical protein
469	cgd7_4350	1.032	0.906	myosin'unconventional myosin fused to an RCC1 domain (unique)'
470	cgd3_3690	1.031	1.169	U5 snRNP 100 kD protein, putative
471	cgd2_1050	1.030	0.927	signal peptide
472	cgd2_2290	1.029	1.134	cyclin 6 pcl7
473	cgd5_4420	1.029	1.151	MJ0710-like (thump + methylase domains). RNA methylase
474	cgd3_850	1.027	1.125	hypothetical protein
475	cgd3_4060	1.027	0.938	histone transcription regulator (HIR1)-like WD40 repeat protein
476	cgd3_2950	1.027	1.021	ribosomal RNA assembly protein mis3/dribble/Krr1p. KH domain
477	cgd5_2030	1.025	1.033	thioredoxin, putative
478	cgd5_730	1.024	0.901	DNA-directed RNA polymerase I, putative
479	cgd3_4260	1.024	1.009	peptidase'insulinase like peptidase'
480	cgd4_4040	1.023	1.066	Yer006wp-like. Yjeq GTpase
481	cgd3_3180	1.023	0.859	Ser/Thr protein kinase
482	cgd4_1820	1.023	0.826	hypothetical protein
483	cgd5_960	1.023	1.119	Oxr1p like TLDc domain containing protein
484	cgd6_2610	1.023	1.133	ERCC1 excision repair 1; C-terminal HhH domain
485	cgd2_180	1.023	0.848	hypothetical protein
486	cgd2_270	1.022	1.086	translation initiation factor eIF-3 subunit 7
487	cgd6_1390	1.022	1.052	ribosomal protein S23
488	cgd5_2870	1.022	1.079	F11M21.28-like; 3 CCH RNA binding domain protein involved in RNA metabolism
489	cgd4_2630	1.021	0.894	hypothetical protein
490	cgd3_130	1.021	1.121	DRG like OBG family GTpase fused to an RNA binding domain TGS domain, Fun11p
491	cgd5_2790	1.021	1.105	katanin p60/fidgetin family with AAA ATpase
492	cgd4_2430	1.020	1.119	hypothetical protein containing a signal peptide and 2 transmembrane domains
493	cgd7_3320	1.019	1.071	RING finger protein
494	cgd8_4110	1.019	0.890	hypothetical protein
495	cgd2_2660	1.017	1.093	UDP N-acetylglucosamine transporter-like nucleotide sugar transporter with 10 transmembrane domains
496	cgd5_2640	1.017	0.850	little finger (CxxC...CxxC) domain containing protein
497	cgd3_3820	1.016	1.060	DNA LIGASE I
498	cgd3_2270	1.016	0.976	WD repeat protein
499	cgd2_70	1.016	1.068	ABC transporter, with 12 x transmembrane domains and 2x AAA domains
500	cgd7_2020	1.014	1.061	hypothetical protein
501	cgd4_3590	1.013	1.038	conserved hypothetical protein
502	cgd2_1530	1.012	1.071	conserved hypothetical protein
503	cgd8_4410	1.012	1.108	hypothetical protein, possible transmembrane domain
504	cgd6_4870	1.011	1.104	hypothetical protein
505	cgd8_1400	1.011	0.903	glycerol-3-phosphate acyltransferase family, possible plant origin, possible signal peptide plus 2 transmembrane domains
506	cgd3_3080	1.011	0.994	hypothetical protein
507	cgd5_3220	1.008	0.881	proteasome subunit beta7; NTN hydrolase fold
508	cgd2_4360	1.006	0.953	hypothetical protein

509	cgd7_3650	1.005	0.854	DP1 DNA binding protein
510	cgd4_700	1.005	1.000	conserved protein of possible plant or bacterial origin
511	cgd6_3060	1.005	1.197	hypothetical protein
512	cgd7_2770	1.004	0.824	uncharacterized low complexity protein
513	cgd6_3890	1.004	0.928	hypothetical protein
514	cgd3_2540	1.004	1.059	histone H3
515	cgd7_2350	1.003	1.168	hypothetical small protein, possible signal peptide
516	cgd3_2100	1.002	1.067	phosphatidylserine decarboxylase, putative
517	cgd6_3460	1.001	0.917	hypothetical protein
518	cgd1_3130	1.001	0.940	conserved eukaryotic nuclear protein that shares a domain with yeast Lcp5p, a component of the U3 small nucleolar ribonucleoprotein
519	cgd1_780	1.001	0.804	hypothetical protein, possible 2 transmembrane domains
520	cgd7_430	1.000	0.874	cyclin-dependent kinase-related kinase, putative
521	cgd4_3330	1.000	0.932	hypothetical protein
522	cgd8_1090	1.000	0.864	hypothetical protein
523	cgd2_2840	1.000	1.117	pleckstrin homology (PH) domain containing protein with N-terminal ankyrin repeats
524	cgd6_3920	0.997	0.899	hypothetical protein with signal peptide
525	cgd6_2700	0.997	0.905	Low complexity protein, possible apicomplexan conserved
526	cgd5_170	0.996	0.832	hypothetical protein
527	cgd1_1210	0.996	1.163	conserved hypothetical protein
528	cgd2_1230	0.996	1.140	hypothetical protein
529	cgd3_2910	0.995	0.939	hypothetical protein
530	cgd7_4020	0.995	0.905	cryptopsoridial mucin, large thr stretch, signal peptide sequence
531	cgd3_3470	0.995	0.951	proliferating cell nuclear antigen
532	cgd4_320	0.994	1.106	Rab11, putative
533	cgd4_630	0.994	0.954	RAD14/XpA, DNA excision repair protein
534	cgd5_2600	0.993	1.173	putative leucine aminopeptidase; of possible plant or bacterial origin
535	cgd1_2800	0.992	1.138	conserved hypothetical protein
536	cgd2_2400	0.992	0.884	ATPase, class II, type 9B, putative
537	cgd2_320	0.992	0.906	conserved hypothetical protein
538	cgd7_640	0.991	1.045	Prp16p pre-mRNA splicing factor. HrpA family SFII helicase
539	cgd2_90	0.991	1.105	ABC transporter with 9x transmembrande domains and 2xAAA
540	cgd6_3970	0.991	0.923	glutaredoxin-like protein; 2 thioredoxin folds
541	cgd3_4220	0.990	1.169	secreted insulinase like peptidase, signal peptide
542	cgd4_1910	0.990	0.985	conserved hypothetical protein
543	cgd5_2720	0.990	1.067	large protein containing a signal peptide
544	cgd6_3080	0.989	1.003	hypothetical protein with a signal peptide
545	cgd7_2670	0.988	1.105	serine/threonine protein phosphatase, putative
546	cgd8_2280	0.987	0.962	uncharacterized protein with 5 transmembrane domain
547	cgd3_1250	0.986	1.155	ribosomal protein L14
548	cgd7_960	0.985	1.010	hypothetical protein
549	cgd6_1530	0.985	1.144	DNA repair protein Rad4p
550	cgd5_10	0.985	0.925	signal peptide containing protein
551	cgd7_3520	0.985	0.875	hypothetical protein
552	cgd2_1220	0.985	1.170	conserved hypothetical protein
553	cgd8_4840	0.984	0.912	large protein with 2 MYB domains plus low complexity; GA repeat and Q repeat at the C-terminus
554	cgd1_360	0.983	1.050	conserved hypothetical protein
555	cgd7_840	0.982	0.846	hypothetical protein
556	cgd1_760	0.982	0.897	dual specificity phosphatase
557	cgd7_3350	0.982	1.110	eukaryotic DNA topoisomerase I
558	cgd8_3980	0.982	1.052	hypothetical protein
559	cgd8_4320	0.982	0.917	possible conserved domain (Zn finger?)
560	cgd7_1190	0.981	1.096	Ser/Thr protein kinase
561	cgd3_4210	0.980	0.972	secreted insulinase like peptidase, signal peptide
562	cgd4_3820	0.980	0.850	hypothetical protein
563	cgd6_3100	0.979	0.896	dual specificity phosphatase, signal peptide
564	cgd2_4130	0.979	1.121	ORF 73, contains large complex repeat CR 73, putative
565	cgd7_2550	0.978	1.133	hypothetical protein
566	cgd6_5110	0.978	0.921	large hypothetical protein with possible signal peptide
567	cgd6_2200	0.976	1.146	leucine-rich repeats protein
568	cgd5_2550	0.976	1.128	polyubiquitin with 3 Ub domains
569	cgd2_1170	0.975	1.082	zinc finger protein
570	cgd7_5340	0.975	1.185	uncharacterized protein
571	cgd2_4320	0.974	1.071	thioredoxin reductase 1
572	cgd8_2650	0.974	1.085	conserved hypothetical protein
573	cgd7_3730	0.974	0.875	long chain fatty acyl diphosphate synthase
574	cgd4_1350	0.974	0.885	regulator of chromosome condensation

575	cgd3_1020	0.973	1.025	hypothetical protein
576	cgd6_2340	0.973	0.924	hypothetical protein with signal peptide and 12 or more transmembrane domains
577	cgd5_2460	0.973	0.947	RNA binding RGG repeats plus RRM domain containing protein
578	cgd8_3330	0.973	0.872	conserved hypothetical protein
579	cgd7_4760	0.972	1.053	ribosomal protein S4, putative
580	cgd6_2170	0.972	0.930	60S ribosomal protein L11
581	cgd8_3220	0.971	1.133	La domain fused to two RRMs (as in the La autoantigen) which is further fused to a C-terminal domain present in the plant DCL protein and the C-terminus of the alpha subunit of the plant RNA polymeras
582	cgd4_2130	0.971	1.101	TBC domain containing protein
583	cgd4_2540	0.971	1.054	26S proteasome regulatory subunit 26b like AAA ATpase
584	cgd3_560	0.970	0.810	hypothetical protein with a signal peptide
585	cgd2_2150	0.970	0.867	hypothetical protein
586	cgd2_2800	0.970	0.855	THUMP RNA binding domain containing protein, Yg1232wp-like
587	cgd6_680	0.969	0.921	conserved protein with possible C2 domain
588	cgd1_2670	0.968	0.937	ankyrin-related protein, putative
589	cgd3_3850	0.968	0.922	Cbf5p; centromere-binding factor 5 like PUA domain containing protein with a type I pseudouridine synthase domain
590	cgd8_2250	0.966	0.933	Rat1 Kar1/Rat1 like 5'-3' exonuclease
591	cgd6_1200	0.965	0.977	diphthine synthase; diphthamide biosynthesis methyltransferase
592	cgd8_3570	0.965	1.060	predicted secreted protein
593	cgd6_4290	0.965	0.881	large protein having a MATH plus possible UBC hydrolase plus type I pseurousyn plus hexapeptide
594	cgd6_940	0.964	1.071	pescadillo containing BRCT domain
595	cgd8_1150	0.964	0.883	choline-phosphate cytidylyltransferase
596	cgd6_1540	0.963	0.857	possible histone mRNA hairpin-binding protein
597	cgd2_560	0.962	0.868	large low complexity protein
598	cgd4_3160	0.962	1.091	putative 40S ribosomal protein S3A
599	cgd4_3270	0.962	1.105	heat shock 105kD; heat shock 105kD alpha; heat shock 105kD beta; heat shock 105kDa protein 1
600	cgd1_3610	0.961	1.119	secreted protein of the alpha beta hydrolase superfamily, signal peptide
601	cgd1_2450	0.961	0.959	ELP3 like acetyltransferase involved in transcription
602	cgd4_3020	0.961	0.939	hypothetical protein with a signal peptide
603	cgd3_4190	0.960	1.113	secreted insulinase like peptidase
604	cgd7_130	0.959	1.111	40S ribosomal protein S14
605	cgd8_5240	0.959	0.805	U2AG splicing factor U2AF U2snRNP auxilliary factor small subunit CCCh+RRM+CCCh-like
606	cgd6_2860	0.959	0.895	uncharacterized coiled coil protein
607	cgd6_3480	0.958	0.888	hypothetical protein
608	cgd4_710	0.957	0.886	Ynr054cp, basal transcriptional activator hABT1, RRM domain containing protein
609	cgd4_3200	0.957	0.918	hypothetical protein
610	cgd4_1980	0.957	1.152	TBC domain containing protein
611	cgd1_3780	0.957	0.890	domain KOG1015, transcription regulator XNP/ATRX, DEAD-box superfamily, signal peptide
612	cgd5_30	0.956	0.947	hypothetical protein
613	cgd2_1790	0.956	0.946	hypothetical protein
614	cgd6_2350	0.956	1.056	mitochondrial carrier protein, 4 transmembrane domain
615	cgd3_1300	0.956	0.946	60S ribosomal protein L12
616	cgd8_1680	0.955	1.041	hypothetical protein
617	cgd5_850	0.955	0.850	vacuolar ATP synthase subunit F
618	cgd1_2220	0.955	0.871	apicomplexan specific protein
619	cgd8_5220	0.955	0.876	signal peptide, 4+ transmembrane domains
620	cgd3_390	0.954	1.090	RecQ SF II RNA helicase, DEXDc+HELICc
621	cgd8_5160	0.953	0.957	uncharacterized apicomplexan-specific protein
622	cgd2_390	0.952	1.156	hypothetical protein
623	cgd6_1000	0.952	0.929	hypothetical protein with signal peptide
624	cgd7_420	0.952	0.893	protein with DEXDc plus ring plus HELICc; possible SNF2 domain
625	cgd7_1690	0.952	1.002	meiotic recombination protein DMC1-like protein
626	cgd5_1960	0.951	0.926	enolase (2-phosphoglycerate dehydratase)
627	cgd7_3210	0.950	1.048	hypothetical protein
628	cgd5_910	0.950	1.183	protein with conserved domain that is fused in vertebrates to neuralized domain repeats
629	cgd4_2440	0.949	0.918	small hypothetical protein with signal peptide
630	cgd7_3120	0.948	0.955	pyruvate decarboxylase
631	cgd1_3710	0.948	0.873	acetyl-coenzyme A synthetase
632	cgd2_3070	0.948	0.991	high mobility group (HMG)-box
633	cgd6_4170	0.947	1.174	hypothetical protein
634	cgd5_3160	0.947	1.040	actin
635	cgd7_3260	0.947	0.895	SWIB domain protein
636	cgd4_3550	0.947	0.959	secreted protein with signal peptide and 12 KAZAL repeats and a mucin-like stretch of threonines
637	cgd6_1440	0.946	1.183	hypothetical protein
638	cgd5_3330	0.946	0.998	hypothetical protein with 5 transmembrane domains
639	cgd2_4310	0.946	0.879	hypothetical protein

640	cgd4_3370	0.945	0.880	hypothetical protein
641	cgd4_4460	0.945	0.861	dihydrofolate reductase-thymidylate synthase
642	cgd7_1880	0.944	0.828	60S ribosomal protein L44
643	cgd2_2560	0.944	0.883	hypothetical protein, signal peptide
644	cgd3_1440	0.943	0.891	U1 small nuclear ribonucleoprotein c like finger
645	cgd5_3990	0.942	0.886	protein with 4 transmembrane domains, signal peptide and a RING domain
646	cgd1_1070	0.942	0.816	RRM domain protein
647	cgd6_4410	0.942	1.178	DNA polymerase delta catalytic subunit. DNAQ-like 3'-5' exonuclease; RNAseH fold
648	cgd2_3700	0.942	0.942	SWI/SNF related putative transcriptional regulator ATpase
649	cgd5_1240	0.942	1.040	Tsr1p GTpase, multitransmembrane region protein
650	cgd8_1890	0.941	0.858	hypothetical protein
651	cgd5_3230	0.941	1.062	superoxide dismutase
652	cgd7_240	0.941	0.932	hypothetical protein
653	cgd1_240	0.940	1.012	predicted secreted protein
654	cgd4_3650	0.940	0.969	conserved hypothetical protein
655	cgd6_3930	0.940	0.859	hypothetical protein
656	cgd5_3560	0.940	0.993	CNcl1p/MJ0026/YebU-like. SUN family methylase
657	cgd4_2880	0.939	0.868	hypothetical protein
658	cgd4_3670	0.939	0.917	uncharacterized low complexity protein
659	cgd8_430	0.938	1.043	60S ribosomal protein L7A
660	cgd7_4500	0.938	1.096	sgnal peptide, large secreted protein
661	cgd5_660	0.938	0.820	thioredoxin domain containing protein
662	cgd6_1010	0.937	0.922	PP2C like protein phosphatase
663	cgd5_2780	0.936	0.924	40S ribosomal protein S10
664	cgd5_3360	0.936	1.067	adenylate kinase
665	cgd7_3410	0.936	0.803	SNF5 like protein
666	cgd4_430	0.936	0.949	ORC5 like AAA+ ATpase
667	cgd8_940	0.936	0.951	conserved hypothetical protein
668	cgd8_4680	0.935	0.804	conserved protein; Py hit 23481194
669	cgd3_880	0.935	0.958	hypothetical protein
670	cgd8_2020	0.935	0.856	OGG1 like 8-oxoguanine DNA glycosylase involved in DNA repair
671	cgd4_3130	0.935	0.986	putative eIF6, translation initiation factor 6
672	cgd6_930	0.935	0.934	Dim1p-like ERMB/KSGA methylase
673	cgd7_940	0.935	1.078	splicing factor RRM domain containing protein; T22E16.120 SC35-like splicing factor
674	cgd8_1360	0.934	0.969	hypothetical protein
675	cgd4_670	0.933	1.061	oocyst wall protein 3
676	cgd7_4540	0.933	0.909	CP15/60 sporozoite 60K protein
677	cgd5_940	0.933	0.904	histone H2A
678	cgd8_880	0.933	1.007	transcription initiation factor TFIIB Sua7p; ZnR+2cyclins
679	cgd7_970	0.932	1.150	Syf1p. protein with 8 HAT domains
680	cgd7_3870	0.932	1.181	conserved hypothetical protein
681	cgd1_40	0.932	0.801	UDP-galactose transporter, predicted signal peptide and 9 transmembrane domains
682	cgd3_460	0.932	0.920	glutathione peroxidase
683	cgd5_3710	0.931	0.811	small nuclear ribonucleoprotein U6
684	cgd7_1230	0.931	1.167	hypothetical protein
685	cgd6_3340	0.931	0.925	60S ribosomal protein L32
686	cgd7_5060	0.930	0.964	putative 40S ribosomal protein S20
687	cgd5_3840	0.930	0.827	hypothetical protein
688	cgd8_3710	0.930	0.905	hypothetical protein
689	cgd1_390	0.930	1.162	hypothetical protein with low amino acid complexity
690	cgd7_4860	0.928	1.008	uncharacterized protein
691	cgd2_490	0.928	0.870	hypothetical protein with signal peptide
692	cgd6_4990	0.927	0.925	predicted peptidase of the alpha/beta-hydrolase fold
693	cgd6_2260	0.926	1.038	Hca4p helicase DBP4 (helicase CA4). EIF4A-1-family RNA SFII helicase
694	cgd1_340	0.925	1.114	N-terminal machado-Joseph disease protein like domain, C-terminal UBX, DNA repair like domain
695	cgd7_810	0.925	0.900	protein phosphatase PP2A, calcineurin like phosphoesterase superfamily
696	cgd1_3430	0.925	1.178	hypothetical protein
697	cgd2_870	0.925	0.896	drosophila CG6013 like HMG domain containing protein with a coiled coil region at the N-terminus
698	cgd6_3710	0.923	0.922	40S ribosomal protein S30
699	cgd7_4050	0.923	0.823	ribosomal protein L38
700	cgd3_2990	0.922	1.062	hypothetical protein with signal peptide and 7 transmembrane domains
701	cgd1_870	0.921	1.014	cyclophilin like peptidyl-prolyl cis-trans isomerase, signal peptide
702	cgd6_1430	0.921	1.020	transcription factor E2F wHTH only
703	cgd2_820	0.921	0.964	putative translation initiation factor 1 (eIF1), SUI1p
704	cgd2_2650	0.921	1.087	hypothetical protein, signal peptide, transmembrane domain near C-terminus
705	cgd5_4230	0.921	0.849	hypothetical protein
706	cgd1_1990	0.921	0.877	hypothetical protein

707	cgd7_4360	0.921	1.031	hypothetical protein
708	cgd8_1940	0.921	0.830	RAD25, helicase involved in DNA repair
709	cgd3_2890	0.920	0.978	Prp8. JAB/PAD domain
710	cgd8_3480	0.919	0.981	60S ribosomal protein L34
711	cgd8_4540	0.919	1.070	predicted coiled coil protein
712	cgd8_5350	0.919	0.976	conserved hypothetical protein
713	cgd3_1420	0.918	0.931	conserved small protein
714	cgd7_4880	0.918	0.935	signal peptide plus GPI anchored membrane protein
715	cgd2_20	0.917	0.959	heat shock 70 (HSP70) protein
716	cgd8_3700	0.917	0.891	hypothetical protein
717	cgd8_40	0.917	0.858	signal peptide, predicted secreted protein of cryptosprodidium-specific SKSR gene family
718	cgd5_1380	0.917	0.955	F11M21.28-like protein having 3 CCCH RNA binding domains; involved in RNA metabolism
719	cgd4_1560	0.916	1.044	hypothetical protein
720	cgd8_2850	0.916	1.037	hypothetical protein
721	cgd3_170	0.916	0.837	hypothetical protein
722	cgd2_810	0.916	1.104	calmodulin
723	cgd7_1180	0.915	0.917	conserved hypothetical protein
724	cgd5_530	0.915	0.900	vacuolar H-ATpase subunit D
725	cgd3_1290	0.915	1.117	14-3-3 domain containing protein
726	cgd8_660	0.915	1.086	large low complexity protein
727	cgd8_2870	0.915	0.988	60S ribosomal protein L10A
728	cgd5_1520	0.914	0.968	hypothetical protein
729	cgd4_2840	0.914	1.023	Mra1/NEP1 like protein, involved in pre-rRNA processing, adjacent genes putative paralogs
730	cgd8_4280	0.914	1.091	zz domain protein
731	cgd6_2690	0.914	1.130	macrophage infectivity potentiator (MIP);FKBP peptidyl-prolyl cis-trans isomerase-like fold, signal peptide + ER retention motif
732	cgd1_3230	0.913	1.164	protein kinase
733	cgd3_3370	0.913	0.908	large hypothetical protein with signal peptide
734	cgd6_40	0.913	1.098	conserved hypothetical protein
735	cgd8_4200	0.913	0.818	uncharacterized protein with several coiled-coil regions
736	cgd6_5030	0.913	0.963	hypothetical protein
737	cgd6_4630	0.913	0.940	40S ribosomal protein S8
738	cgd5_410	0.913	0.925	Rad51
739	cgd4_4080	0.912	0.800	putative rab GDI alpha
740	cgd5_4560	0.912	1.003	tryptophan synthase trpB of possible bacterial origin
741	cgd4_2220	0.912	0.830	possible double-stranded DNA-binding domain, small conserved protein
742	cgd2_2100	0.911	0.841	hypothetical protein, possible signal peptide
743	cgd4_2290	0.911	0.974	hypothetical protein
744	cgd2_3190	0.911	1.052	protein kinase, putative
745	cgd2_1500	0.911	1.058	possible translation initiation factor with possible PINT domain
746	cgd5_1650	0.910	1.087	hypothetical protein
747	cgd5_1980	0.910	1.058	hypothetical protein
748	cgd7_920	0.910	0.914	protein with 2 possible TPR domains, possible n-terminal acetyltransferase
749	cgd2_4210	0.910	0.880	uncharacterized protein with several coiled coil regions
750	cgd5_2630	0.909	0.969	60S ribosomal protein L7-B; Rp17bp; L30 RNA binding domain
751	cgd5_1470	0.909	1.023	signal peptide containing protein having a NDK (nucleoside-diphosphate kinase) domain
752	cgd2_4200	0.909	1.020	hypothetical protein
753	cgd3_710	0.908	0.880	large hypothetical protein with signal peptide
754	cgd6_1980	0.908	0.837	hypothetical protein with a signal peptide
755	cgd3_1960	0.908	1.135	hypothetical protein
756	cgd2_1370	0.907	1.082	hypothetical coiled coil protein
757	cgd7_3390	0.907	0.936	secreted alpha beta hydrolyse, signal peptide, secreted patatin like esterase (ralstonia best hits)
758	cgd6_4210	0.907	0.920	kinesin-like boursin, putative
759	cgd6_400	0.907	0.865	Pre-mRNA polyadenylation factor
760	cgd5_2310	0.907	1.009	Sin3 like paired amphipathic helix containing protein
761	cgd5_4290	0.906	0.979	Swi/SNf2 RAD26
762	cgd4_2800	0.906	0.915	hypothetical protein having a signal peptide
763	cgd2_2860	0.906	0.867	hypothetical protein
764	cgd2_2240	0.905	0.840	hypothetical protein
765	cgd8_3390	0.905	1.043	S.cerevisiae Ssf2p/drosophila peter pan like protein that has an IMP4 domain at its N-terminus and is involved in rRNA processing
766	cgd8_4100	0.905	1.136	PRP43 involved in spliceosome disassembly mRNA splicing
767	cgd3_3000	0.904	1.034	TBC domain containing protein
768	cgd6_2090	0.904	0.951	CpCOWP1, oocyst wall protein with type I and type II cysteine-rich repeats
769	cgd6_4670	0.904	0.996	Prp9p-like splicing factor 3a subunit 3 snRNP. C-terminal C2H2
770	cgd3_1530	0.904	0.842	hypothetical protein
771	cgd7_360	0.903	0.993	heat shock protein, Hsp70

772	cgd1_1590	0.902	1.030	hypothetical protein
773	cgd3_4320	0.902	0.964	conserved hypothetical protein
774	cgd6_140	0.901	0.947	possible Na+/H+ and K+/H+ antiporter with 12 transmembrane domains, duplicated adjacent gene
775	cgd7_1080	0.901	0.833	eIF3-p47 with JAB/PAD domain
776	cgd1_2050	0.901	0.919	hypothetical protein
777	cgd7_4630	0.900	0.850	hypothetical protein
778	cgd4_250	0.900	0.883	proteasome subunit alpha type 4, NTN hydrolase fold
779	cgd1_1320	0.900	1.112	developmental protein, putative
780	cgd4_140	0.900	1.014	Swi2/Snf2 ATpase,Rad16 ortholog
781	cgd5_1990	0.900	0.997	hypothetical protein
782	cgd7_5390	0.900	1.159	uncharacterized protein
783	cgd1_220	0.900	0.972	glycosylphosphatidylinositol transamidase, involved in GPI anchor biosynthesis
784	cgd6_4160	0.900	0.927	EF-hands domain containing protein
785	cgd1_1900	0.899	1.043	Fur1p like uracil phosphoribosyltransferase
786	cgd2_3290	0.899	0.830	hypothetical protein with a signal peptide plus thr stretch, possible mucin
787	cgd6_4590	0.899	0.897	ankyrin-related protein, putative
788	cgd4_940	0.899	0.902	hypothetical protein
789	cgd3_2230	0.899	1.014	hypothetical protein
790	cgd4_930	0.899	0.875	pap1p; poly A polymerase (eukaryotic type)
791	cgd5_1900	0.898	0.996	XPA binding protein 1
792	cgd1_270	0.898	1.118	zinc finger protein
793	cgd7_2240	0.898	0.990	hypothetical protein, signal peptide, possible secreted protein
794	cgd4_1570	0.897	0.880	hypothetical protein, possible conserved
795	cgd4_2600	0.897	0.988	UDP-glucose 4-epimerase
796	cgd6_2390	0.896	1.045	DNA topoisomerase III beta-1, putative
797	cgd6_850	0.896	1.140	thioredoxin; protein disulfide isomerase A6, signal peptide, possible transmembrane domain in C-terminal region
798	cgd8_5390	0.896	0.963	conserved hypothetical protein
799	cgd4_2830	0.895	0.836	Mra1/NEP1 like protein, involved in pre-rRNA processing, adjacent genes putative paralogs
800	cgd5_2380	0.895	1.117	unchacterized apicomplexan-specific serine rich low complexity protein
801	cgd8_5340	0.895	0.897	coiled coil protein
802	cgd8_2520	0.895	1.019	Mtr4p like SKI family SFII helicase
803	cgd1_1660	0.895	0.974	60S ribosomal protein L36
804	cgd1_1840	0.895	0.922	hypothetical protein
805	cgd2_1120	0.895	1.016	putative molybdopterin synthase sulphurylase
806	cgd4_240	0.894	0.820	hypothetical protein
807	cgd7_5160	0.894	1.054	U1 small nuclear ribonucleoprotein A, rrm domain
808	cgd7_300	0.894	0.953	CpCCP2; extracellular protein with a signal peptide, ricin, discoidin, NEC, LCCL, 2 levanase and an apicomplexan-specific cysteine-rich repeat (apicA repeat)
809	cgd4_530	0.894	0.940	26S proteasome regulatory subunit 5a with a vWA domain and two ubiquitin interacting motifs (UIM)
810	cgd6_100	0.893	0.983	leucine rich repeat (LRR) protein
811	cgd1_970	0.893	1.011	RING finger containing protein
812	cgd4_2750	0.893	0.951	PWP1 family protein with WD40 repeats
813	cgd8_2350	0.893	0.921	cyclophilin-RNA interacting protein, putative
814	cgd6_1870	0.893	0.875	signal recognition particle SPR19
815	cgd1_2840	0.893	0.987	contains a UVDDB domain that is present in CPSF_A and damage specific DNA binding protein 1
816	cgd4_150	0.893	1.005	pre-mRNA splicing protein; Prp31p--like
817	cgd5_1010	0.893	0.975	hypothetical protein
818	cgd5_2500	0.892	0.804	ubiquitin C-terminal hydrolase of the cysteine proteinase fold
819	cgd4_32	0.892	1.038	hypothetical protein
820	cgd2_3110	0.891	0.905	hypothetical protein containing a signal peptide
821	cgd1_3650	0.891	0.983	hypothetical protein, signal peptide
822	cgd6_830	0.891	0.837	conserved hypothetical protein
823	cgd4_3290	0.890	0.926	articulin family protein, adjacent paralogous gene
824	cgd2_3000	0.890	1.073	40S ribosomal protein S16
825	cgd1_3590	0.890	0.805	hypothetical protein, possible transmembrane domain
826	cgd5_2240	0.890	1.128	DPAGT1 like N-acetylglucosamine-1-phosphate transferase
827	cgd8_1380	0.890	0.960	hypothetical protein
828	cgd2_2790	0.890	1.060	conserved hypothetical protein
829	cgd2_1410	0.890	0.906	possible domain KOG3410, conserved alpha-helical protein
830	cgd6_2730	0.889	1.071	protein with signal peptide plus possible GPI signal or transmembrane domain at C-terminus
831	cgd1_960	0.889	1.001	RING finger containing protein
832	cgd1_2080	0.889	1.122	hypothetical protein containing signal peptide and 3 transmembrane domain regions
833	cgd5_3590	0.889	0.911	putative transport protein
834	cgd1_180	0.889	1.057	hypothetical protein
835	cgd6_540	0.888	1.121	Ser/Thr protein kinase
836	cgd8_2740	0.888	1.168	hypothetical protein

837	cgd5_2230	0.888	0.961	membrane associated thioredoxin
838	cgd7_2790	0.888	0.833	regena domain protein (CCR-Not complex protein subunit 3), putative
839	cgd3_1380	0.888	0.936	hypothetical protein
840	cgd2_150	0.888	0.831	hypothetical protein
841	cgd6_3730	0.888	0.902	large extracellular protein with a signal peptide, apple domain and a transmembrane region
842	cgd8_20	0.887	0.870	signal peptide, predicted secreted protein of cryptosprodidium-specific SKSR gene family
843	cgd3_2440	0.886	0.893	ribosomal protein S18A, rps18ap, HhH domain
844	cgd6_10	0.886	1.027	hypothetical protein with signal peptide and cysteine rich region at the C-terminus
845	cgd6_1660	0.886	0.972	conserved hypothetical protein
846	cgd8_4760	0.886	0.847	arginine n-methyltransferase
847	cgd6_490	0.886	1.133	zinc ZIP transporter protein, putative
848	cgd1_3040	0.886	1.024	triosephosphate isomerase
849	cgd5_1800	0.885	1.064	hypothetical protein
850	cgd2_2440	0.884	0.842	hypothetical protein
851	cgd6_2760	0.884	0.914	hypothetical protein
852	cgd4_2640	0.884	0.887	hypothetical protein
853	cgd2_3230	0.884	0.956	heat shock protein DnaJ Pfj2, putative
854	cgd6_5250	0.884	1.193	transcription elongation factor, SPT6-like
855	cgd6_3820	0.883	1.007	membrane bound aspartyl proteinase with a signal peptide plus transmembrane domain
856	cgd4_3040	0.883	1.030	NifS-like protein; cysteine desulfurase
857	cgd5_2810	0.883	0.929	ULP1 like chllamydin domain containing protease
858	cgd4_4240	0.883	1.146	insulinase like peptidase
859	cgd5_2270	0.883	0.907	maternal embryonic leucine zipper kinase
860	cgd3_2940	0.883	0.996	probable phosphatidylserine/phosphatidylglycerophosphate/cardiolipin synthase, 2x SMART_PLDc domains, possible bacterial origin
861	cgd2_110	0.882	0.856	Dbp6p, eIF4a-1 family RNA SFII helicase
862	cgd8_3810	0.882	1.015	hypothetical protein
863	cgd5_590	0.882	0.806	hypothetical protein with possible transmembrane domain within C
864	cgd5_3050	0.882	0.939	hypothetical protein
865	cgd3_930	0.882	1.033	DnaJ domain, possible zf-CSL following
866	cgd1_2030	0.881	0.965	membrane associated protein with over 9 transmembrane domains, signal peptide
867	cgd8_4150	0.880	0.975	RRM+RGG RNA binding repeats
868	cgd3_2460	0.880	1.040	UbiA prenyltransferase family member (3I27) , putative
869	cgd5_1310	0.880	1.072	conserved protein with possible signal peptide and ER retention motif; possible ER protein
870	cgd3_2790	0.880	0.942	cullin domain containing protein
871	cgd8_2330	0.879	0.966	pyridine nucleotide/ NAD(P) transhydrogenase alpha plus beta subunits, duplicated gene, possible signal peptide plus 12 transmembrane regions
872	cgd1_2400	0.879	0.996	signal peptide, secreted protein
873	cgd4_1030	0.879	0.841	eukaryotic translation initiation factor
874	cgd4_3480	0.878	0.881	hypothetical protein with signal peptide plus possible GPI anchor or transmembrane domain near C-terminus, within large locus of signal peptide containing proteins
875	cgd6_1040	0.878	0.818	RNAse P Rpr2/Rpp21 subunit domain
876	cgd7_2200	0.878	0.929	hypothetical protein, signal peptide
877	cgd1_2360	0.878	1.000	protein phosphatase 4 (formerly X), catalytic subunit; Protein phosphatase 4, catalytic subunit
878	cgd3_3940	0.878	0.887	predicted membrane associated protein
879	cgd1_150	0.878	0.968	predicted secreted protein, signal peptide
880	cgd8_4850	0.877	0.882	conserved hypothetical protein
881	cgd3_2010	0.877	1.155	hypothetical protein
882	cgd5_2040	0.877	0.834	dolichol phosphate mannose synthase, putative
883	cgd6_5320	0.876	1.133	conserved hypothetical protein
884	cgd2_2550	0.876	0.940	hypothetical protein, signal peptide
885	cgd8_4020	0.875	1.022	hypothetical protein
886	cgd3_350	0.875	1.057	Pop2p-like 3'5' exonuclease, CCR4-NOT transcription complex
887	cgd3_3930	0.875	0.971	60S ribosomal protein L27A or L27a
888	cgd8_2900	0.874	0.918	ERV41 like membrane associated protein involved in vesicular transport with a transmembrane region near the C-terminus
889	cgd2_410	0.874	1.058	signal peptide, serine stretches, possible low mw mucin glycoprotein, locus of 6 genes
890	cgd3_4340	0.874	0.900	hypothetical protein
891	cgd5_2220	0.874	0.827	small nuclear ribonucleoprotein D1. SM domain containing protein
892	cgd8_2050	0.873	0.917	RRP5 like protein involved in rRNA biogenesis with 7 S1 domains and 5 HAT repeats
893	cgd5_4460	0.873	0.842	hypothetical protein
894	cgd2_720	0.872	0.910	hypothetical protein
895	cgd4_3540	0.872	0.907	conserved hypothetical protein
896	cgd5_1340	0.872	0.978	conserved hypothetical protein
897	cgd4_1650	0.871	0.968	possible thioredoxin H-type of possible fungal or plant origin, small protein
898	cgd7_830	0.871	1.004	queunine tRNA-ribosyltransferase
899	cgd3_1330	0.870	1.077	hypothetical protein having a signal peptide

900	cgd8_5280	0.869	1.048	Deg1p-like type II pseudouridylate synthase TruA
901	cgd8_450	0.869	0.918	DNAj-like protein
902	cgd7_5460	0.869	1.037	VPS16 vacuolar sorting protein
903	cgd3_3170	0.869	0.951	replication factor RFC3 AAA+ ATpase
904	cgd4_3750	0.868	0.879	hypothetical protein with signal peptide
905	cgd6_2430	0.868	1.000	BTF domain, basal transcription factor
906	cgd7_820	0.868	0.957	RAD3'DEXDc+HELICc protein'
907	cgd5_3340	0.868	1.055	putative vacuolar ATP synthase subunit d
908	cgd3_2110	0.868	0.986	nucleolar protein NOP5/NOP58-like pre-mRNA splicinig factor prp31
909	cgd4_2460	0.867	0.959	hypothetical protein with a signal peptide, gene within locus of signal-peptide containing hypothetical proteins
910	cgd7_2640	0.867	0.924	TPR repeat protein
911	cgd4_920	0.867	1.022	conserved hypothetical protein
912	cgd3_160	0.867	1.021	hypothetical protein
913	cgd3_2280	0.866	0.808	hypothetical protein
914	cgd7_2820	0.866	0.882	hypothetical large low complexity protein
915	cgd4_3970	0.866	0.878	signal peptide plus transmembrane domain or GPI anchor, proline rich, acidic stretches
916	cgd7_5200	0.866	1.184	2-phosphoglycerate kinase involved in 2,3 diphosphoglycerate synthesis (P-loop kinase, distorted P-loop)
917	cgd1_1010	0.866	1.126	hypothetical protein
918	cgd2_520	0.866	0.916	mitochondrial phosphate translocator
919	cgd1_1080	0.866	0.931	alpha beta hydrolase
920	cgd1_700	0.866	1.146	ATP-binding cassette protein
921	cgd6_4910	0.865	1.025	F11M21.28-like protein with 3 CCCH RNA binding domains, involved in RNA metabolism
922	cgd1_1440	0.865	0.983	hypothetical protein
923	cgd6_3760	0.865	0.897	hypothetical protein
924	cgd2_1400	0.864	0.910	hypothetical protein
925	cgd8_2840	0.864	1.122	hypothetical protein
926	cgd2_400	0.864	1.044	signal peptide, serine stretches, possible low mw mucin glycoprotein locus of 6 genes
927	cgd2_3050	0.864	1.033	hypothetical protein
928	cgd5_2850	0.864	0.920	hypothetical protein
929	cgd2_1160	0.864	1.135	HSPC021/HSPC025 family protein
930	cgd1_2650	0.864	0.970	hypothetical protein
931	cgd5_4360	0.863	0.983	large protein with possible RING domain
932	cgd4_1260	0.863	1.078	possible tRNA-INTRON ENDONUCLEASE
933	cgd6_5000	0.863	0.928	phosphoprotein phosphatase 2A 65K regulatory chain-like with HEAT repeats
934	cgd8_1120	0.863	0.894	HBS1 eRFS. GTpase. (RNA metabolism; translation)
935	cgd2_3950	0.863	1.070	putative translation elongation factor 1 beta 1
936	cgd7_3550	0.863	1.029	conserved hypothetical protein
937	cgd7_3480	0.862	0.946	cytidylyltransferase (HIGH family) exon-1
938	cgd1_70	0.862	0.879	hypothetical protein
939	cgd6_1140	0.862	0.920	hypothetical protein
940	cgd7_950	0.861	1.158	putative Sec61; signal peptide plus 9 transmembrane domain-containing protein
941	cgd2_4290	0.861	0.918	hypothetical protein
942	cgd1_990	0.861	1.159	pyridine nucleotide/ NAD(P) transhydrogenase alpha plus beta subunits, duplicated gene, 12 transmembrane domain
943	cgd2_80	0.861	1.008	ABC transporter family protein, 2x AAA domain
944	cgd3_4310	0.861	0.983	sexual stage-specific protein kinase
945	cgd6_330	0.860	0.841	hypothetical protein
946	cgd1_320	0.860	1.048	oxysterol binding protein
947	cgd3_4070	0.860	1.074	putative sugar transporter with 12 transmembrane domains
948	cgd3_1630	0.859	1.002	hypothetical protein
949	cgd4_410	0.859	0.945	protein with 4xEZ_heat domains
950	cgd7_790	0.858	0.925	hypothetical protein
951	cgd5_3350	0.858	1.031	hypothetical protein
952	cgd7_5470	0.858	0.915	uncharacterized protein
953	cgd1_1620	0.858	1.054	RPB7 like OB fold RNA polymerase subunit
954	cgd8_4820	0.858	0.988	hypothetical protein
955	cgd3_3010	0.857	0.968	hypothetical protein
956	cgd7_1560	0.857	1.027	DHHC family palmitoyl transferase with a signal peptide and 4 transmembrane domains
957	cgd8_3310	0.857	1.057	S. cerevisiae Ylr022cp like protein that has a C2H2 zinc finger and is a component of the exosome
958	cgd4_1200	0.857	0.925	ubiquitin fusion degradation protein (UFD1); double Psi beta barrel fold
959	cgd5_3010	0.857	0.987	hypothetical protein with possible plus3 domain
960	cgd7_4130	0.857	1.025	Ynl022cp-like. SUN family methylase
961	cgd7_1340	0.856	1.000	conserved hypothetical protein
962	cgd2_230	0.856	1.127	phosphatidylinositol phosphate phosphatase, putative
963	cgd4_1420	0.856	0.906	hypothetical protein

964	cgd4_420	0.855	0.855	signal peptide + 4 transmembrane domain protein
965	cgd7_4580	0.855	0.977	U6 snRNA-associated Sm-like protein LSm5. SM domain
966	cgd7_5320	0.854	0.826	Cwf5-like with 2 ZnR domains (CCCH and RRM domains missing)
967	cgd7_1590	0.854	0.919	uracil-DNA glycosylase, putative
968	cgd5_2760	0.854	0.867	hypothetical protein
969	cgd4_2150	0.854	0.862	hypothetical protein
970	cgd3_2680	0.854	0.968	possible signal peptidase subunit, signal peptide
971	cgd4_2000	0.853	1.031	apicomplexan CP 15/60K like protein
972	cgd1_3630	0.853	0.910	conserved hypothetical protein
973	cgd4_1440	0.853	1.122	apicomplexan specific protein
974	cgd3_4160	0.853	0.820	conserved hypothetical protein
975	cgd7_1410	0.853	1.037	AAA domain containing protein
976	cgd1_2510	0.852	1.085	hypothetical protein, transmembrane domain within N-terminus
977	cgd1_2700	0.852	0.987	methionine aminopeptidase with MYND finger at N-terminus
978	cgd8_2220	0.852	1.036	hypothetical protein with a domain with conserved cysteines shared in dictyostelium, leishmania and plants
979	cgd5_4320	0.852	1.073	frataxin like protein
980	cgd6_3020	0.852	0.963	hypothetical protein with 8 transmembrane domains
981	cgd2_1130	0.852	0.933	hypothetical protein
982	cgd1_2440	0.851	1.196	eukaryotic orthologous group, signal peptide
983	cgd7_2040	0.850	0.902	small nuclear ribonucleo protein
984	cgd4_2470	0.850	0.959	hypothetical protein with a signal peptide, gene within locus of signal-peptide containing hypothetical proteins
985	cgd3_330	0.850	1.017	scully CG7113-PA, putative
986	cgd3_1510	0.850	1.052	cyclin-dependent kinase 3, putative
987	cgd8_2480	0.850	1.079	signal peptide plus possible transmembrane domain paralogs
988	cgd8_3210	0.849	1.076	UvrD like super family I helicase involved in nucleotide excision repair, possible bacterial horizontal transfer
989	cgd6_1510	0.849	0.886	hypothetical protein with 6 transmembrane domains
990	cgd5_4400	0.849	0.870	RIKEN cDNA 2610011N19 gen
991	cgd4_70	0.849	1.042	conserved hypothetical protein
992	cgd3_2300	0.849	0.926	hypothetical protein with transmembrane domain within C- terminus
993	cgd6_1460	0.849	0.971	SEC14 domain containing protein
994	cgd3_3970	0.848	0.867	Yer007c-ap/MCT-1 like PUA RNA binding domain containing protein
995	cgd4_1840	0.848	0.928	Dbp7p, eIF4A-a-family RNA SFII helicase (DEXDc+HELICc)
996	cgd4_2700	0.848	1.194	probable NADPH-cytochrome p450 reductase
997	cgd6_2530	0.848	0.835	hypothetical protein
998	cgd7_460	0.848	0.849	P-type ATpase 3; integral membrane protein with signal peptide and 12 or more transmembrane domains; possible MgtA, cation tranport ATpase.
999	cgd5_820	0.847	1.037	calcium/calmodulin dependent protein kinase with a kinase domain and 4 calmodulin like EF hands
1000	cgd1_2750	0.847	1.185	AAA superfamily ATpase
1001	cgd3_1200	0.847	0.837	possible prefoldin-related protein
1002	cgd3_2640	0.847	1.005	hypothetical protein with conserved domain
1003	cgd8_180	0.847	0.944	hypothetical protein
1004	cgd6_2110	0.846	0.938	hypothetical protein
1005	cgd3_3420	0.846	1.123	protein with 2x EF hands and 4 transmembrane regions
1006	cgd5_2970	0.846	0.834	hypothetical protein
1007	cgd3_1730	0.846	1.051	conserved hypothetical protein
1008	cgd6_4320	0.846	0.891	40S ribosomal protein S5
1009	cgd4_1310	0.846	0.981	hypothetical protein with transmembrane domain
1010	cgd2_170	0.846	0.915	40s ribosomal protein s24
1011	cgd8_1440	0.846	0.961	nucleotide sugar transporter like integral membrane protein with 9 transmembrane domains
1012	cgd7_370	0.846	1.076	protein with a conserved N-terminal region
1013	cgd5_3510	0.845	0.928	co-chaperone GrpE, putative
1014	cgd5_830	0.845	1.084	membrane associated protein with a transmembrane domain near C-terminus
1015	cgd4_1470	0.845	1.023	vacuolar proton translocating ATpase with 7 transmembrane regions near C-terminus
1016	cgd5_100	0.845	0.972	RRPp/PMC2 like exosome 3'-5' exoribonuclease subunit with an RNAseD domain and an HRDc domain
1017	cgd2_3760	0.844	0.988	conserved hypothetical protein
1018	cgd2_3830	0.844	0.914	hypothetical protein
1019	cgd8_310	0.844	0.962	hypothetical protein
1020	cgd2_2000	0.843	0.877	apicomplexan protein with signal peptide and 3 transmembrane domains
1021	cgd2_50	0.842	0.882	SIK1 nucleolar protein Nop56
1022	cgd5_3720	0.842	0.928	40S ribosomal protein S17
1023	cgd5_1580	0.842	0.973	hypothetical protein
1024	cgd6_4700	0.842	0.997	ATP-binding cassette transporter
1025	cgd2_1420	0.841	0.876	hypothetical protein
1026	cgd2_4220	0.841	1.105	hypothetical protein

1027	cgd8_3530	0.840	1.039	predicted secreted protein
1028	cgd4_1520	0.840	0.818	Brx1p nucleolar protein required for biogenesis of the 60S ribosomal subunit
1029	cgd7_3310	0.840	0.857	hypothetical protein
1030	cgd7_3590	0.840	1.043	Mpp10 family of U3 processosome protein
1031	cgd4_3610	0.840	0.937	hypothetical protein with signal peptide
1032	cgd5_4370	0.839	0.965	conserved hypothetical protein
1033	cgd7_1700	0.838	1.018	histone H2B
1034	cgd4_1140	0.838	1.029	conserved hypothetical protein
1035	cgd2_3780	0.838	0.879	uncharacterized protein rich in Thr and Ser residues
1036	cgd6_4720	0.838	0.926	hypothetical protein
1037	cgd3_2330	0.837	0.908	hypothetical protein
1038	cgd2_2910	0.837	0.998	large WD repeat protein
1039	cgd1_1120	0.837	0.940	epsin like ENTH domain (alpha-alpha superhelix)involved in vesicular transport
1040	cgd3_3860	0.836	1.023	coiled coil protein
1041	cgd1_30	0.836	0.817	predicted membrane protein with 12+xtransmembrane domain
1042	cgd1_1910	0.836	1.023	predicted DNAJ protein
1043	cgd6_270	0.836	1.024	hypothetical protein
1044	cgd2_3570	0.836	0.801	YjbN-like Dus1p tRNA dihydouriding synthase Tim barrel
1045	cgd2_1680	0.836	0.978	hypothetical protein
1046	cgd2_3380	0.835	0.992	conserved hypothetical protein
1047	cgd4_1580	0.835	0.916	Spb1p-like, FtsJ methylase
1048	cgd8_1300	0.834	0.896	Smc like ABC ATpase involved in DNA repair
1049	cgd6_4130	0.834	0.887	conserved hypothetical protein
1050	cgd5_3940	0.834	0.864	hypothetical protein, possible apicomplexan-specific
1051	cgd8_3540	0.834	0.974	predicted secreted protein
1052	cgd3_4020	0.833	0.856	eIF-3 p25/subunit 11
1053	cgd2_3270	0.832	0.946	phosphoglucomutase, tandemly duplicated gene
1054	cgd5_2730	0.832	0.937	hypothetical protein
1055	cgd6_4540	0.832	1.119	DHHC family palmitoyl transferase
1056	cgd1_2010	0.832	0.934	hypothetical low complexity coiled coil
1057	cgd2_160	0.832	0.833	IMP4-like U3 small nucleolar ribonucleoprotein (snoRNP)
1058	cgd8_520	0.831	1.091	signal peptide containing protien
1059	cgd4_2670	0.831	1.018	FRP1 like protein involved in DNA repair with a FAT domain and a phosphatidylinositol kinase domain at the C-terminus
1060	cgd4_1530	0.831	0.807	conserved protein
1061	cgd3_3680	0.831	0.945	UIM domain and EF hand containing protein that also has a conserved domain between
1062	cgd4_1210	0.830	0.927	Ms15p; KH + 2 Znknuckle (C2HC)
1063	cgd6_2500	0.830	1.024	hypothetical protein with possible signal peptide
1064	cgd7_5310	0.830	0.947	60S ribosomal protein L24
1065	cgd3_2210	0.830	1.027	replication protein A1 large subunit
1066	cgd7_1070	0.830	0.821	snRNP core protein
1067	cgd7_400	0.830	0.941	hypothetical protein, signal peptide
1068	cgd2_1910	0.830	1.027	putative phosphatidylinositol-4-phosphate 5-kinase, MORN beta hairpin repeats glycine-rich protein
1069	cgd6_2790	0.830	1.003	kinesin heavy chain, putative
1070	cgd2_4370	0.830	0.972	signal peptide
1071	cgd2_4300	0.829	1.011	Maf protein like, involved in RNA metabolism
1072	cgd5_3980	0.828	0.987	hypothetical protein with possible transmembrane domain near N-terminus
1073	cgd4_1010	0.828	1.155	hypothetical protein
1074	cgd2_2060	0.828	0.948	possible transcription factor TFIIH
1075	cgd7_4570	0.827	1.154	DID domain proteins (DID4/DID2 family)
1076	cgd1_420	0.826	0.927	20S proteasome beta subunit D2 (PBD2) , putative
1077	cgd8_500	0.826	0.845	caltractin, centrin
1078	cgd7_3580	0.826	0.927	kelch repeats protein
1079	cgd5_4050	0.826	0.956	hypothetical protein with 4 transmembrane domains
1080	cgd6_1060	0.825	1.149	protein with spectrin repeats, CG12008-like
1081	cgd4_160	0.825	1.004	hypothetical protein, possible transmembrane domain within N-terminus
1082	cgd3_2870	0.825	0.856	putative acyl-CoA synthetases
1083	cgd8_4880	0.825	1.022	3 transmembrane region protein
1084	cgd7_570	0.825	0.950	protein with 2 TPR domains
1085	cgd2_3040	0.825	1.063	hypothetical protein
1086	cgd8_2450	0.824	0.910	formin homology 2 domain (FH2) protein
1087	cgd3_730	0.824	1.057	conserved expressed protein
1088	cgd8_1490	0.824	1.008	bacterial type serine/tyrosine phosphatase
1089	cgd7_2070	0.823	0.859	large membrane associated channel
1090	cgd2_3030	0.823	1.092	hypothetical protein, signal peptide
1091	cgd5_1030	0.823	0.982	apicomplexan conserved protein
1092	cgd7_4550	0.823	1.054	ubiquitin-like protein, putative

1093	cgd2_970	0.822	1.022	hypothetical protein
1094	cgd7_2500	0.822	1.093	Skp1 family protein, putative
1095	cgd2_3690	0.822	0.976	WD repeat protein
1096	cgd6_3490	0.822	0.934	RNA-binding protein, putative
1097	cgd4_1550	0.822	0.800	conserved hypothetical protein
1098	cgd2_3970	0.821	0.923	RRM domain containing protein
1099	cgd4_520	0.821	1.042	hypothetical protein
1100	cgd2_2730	0.821	0.883	Po1 beta superfamily nucleotidyltransferase
1101	cgd1_580	0.821	0.854	dynamin-like protein
1102	cgd3_3720	0.820	0.829	hypothetical protein
1103	cgd1_520	0.820	0.974	vacuolar ATP synthetase subunit
1104	cgd3_3500	0.820	0.854	Nop12p nucleolar protein, RRM domain
1105	cgd7_490	0.820	0.891	hypothetical protein
1106	cgd7_1430	0.820	0.826	possible phosphodiesterase/alkaline phosphatase D, of possible plant or bacterial origin
1107	cgd5_370	0.820	0.809	predicted coiled coil protein
1108	cgd1_310	0.820	1.010	AP endonuclease of the TIM barrel fold, possible bacterial horizontal transfer
1109	cgd2_3930	0.819	0.895	deoxyhypusine synthase
1110	cgd4_1770	0.819	0.986	hypothetical protein
1111	cgd1_140	0.818	0.957	conserved hypothetical protein
1112	cgd7_2410	0.818	0.989	signal peptide, secreted low complexity protein
1113	cgd8_50	0.818	0.913	signal peptide, predicted secreted protein of cryptosprodidium-specific FGLN gene family
1114	cgd7_3920	0.818	0.910	hypothetical protein
1115	cgd5_1450	0.817	1.041	hypothetical protein with signal peptide
1116	cgd3_1220	0.817	0.996	hypothetical protein
1117	cgd5_2740	0.817	0.948	conserved hypothetical protein
1118	cgd3_1500	0.817	0.999	hypothetical protein
1119	cgd5_3550	0.817	0.951	possible glycolipid transfer protein
1120	cgd6_820	0.817	0.843	predicted secreted low complexity protein with a signal peptide
1121	cgd8_580	0.816	1.115	ubiquitin-conjugating enzyme E2, putative
1122	cgd4_2280	0.816	0.963	hypothetical protein
1123	cgd7_2580	0.816	0.942	AMSJ/WSAK like polysaccharide polymerase (glycosyltransferase family)
1124	cgd1_3850	0.816	0.889	signal peptide, possible 4-5x transmembrane domain, SKSR family paralog, telomeric gene
1125	cgd4_4480	0.816	1.051	putative secreted, membrane-associated protein
1126	cgd6_1470	0.816	0.993	protein with SET domain flanked by cysteine clusters plus a C-terminal PHD domain
1127	cgd8_5320	0.815	0.957	Ser/Thr protein kinase
1128	cgd2_2200	0.815	1.020	60S ribosomal protein L37
1129	cgd7_500	0.814	0.867	hypothetical protein
1130	cgd7_1940	0.814	0.873	coiled coil protein
1131	cgd6_4080	0.814	1.036	conserved hypothetical protein
1132	cgd6_5140	0.814	1.016	ERGIC-53-like mannose binding lectin that is a type I membrane protein, transmembrane domain near C, signal peptide
1133	cgd8_280	0.813	0.863	apcomplexan conserved protein
1134	cgd2_1430	0.813	1.028	dynactin subunit p62, putative
1135	cgd7_3490	0.813	0.888	conserved hypothetical protein
1136	cgd8_1530	0.813	0.946	ubiquitin carboxyl terminal hydrolase domain that is fused to a MATH domain
1137	cgd7_2570	0.812	0.895	adrift-like. FTSJ family RNA methylase
1138	cgd7_2450	0.812	0.946	possible membrane associated transporter
1139	cgd4_660	0.812	0.869	Nip7 like PUA domain containing protein involved in ribosomal biogenesis
1140	cgd6_4960	0.811	1.042	serine/threonine-protein kinase, putative
1141	cgd2_710	0.811	0.832	predicted protease
1142	cgd8_4300	0.811	0.982	hypothetical protein
1143	cgd2_2120	0.811	1.009	high mobility group small protein
1144	cgd1_250	0.810	0.943	ATP-dependent RNA helicase, putative
1145	cgd5_3790	0.810	0.813	SNF7 ortholog
1146	cgd6_4570	0.810	0.865	glutamate synthetase, possible bacterial origin, beta-grasp+glutamate synthase catalytic domain
1147	cgd5_2190	0.810	0.942	Nuc1p like endonuclease G
1148	cgd2_2640	0.810	0.902	hypothetical protein
1149	cgd6_4770	0.810	1.059	conserved hypothetical protein
1150	cgd4_1590	0.809	0.919	chromodomain-helicase-DNA-binding'multidomain chromatin protein with the following architecture: chromo-bromo-chromo-SNF2 ATpase'
1151	cgd3_1090	0.809	0.896	Rrp9p/U3-55K-family snoRNP-associated protein with several WD40 repeats
1152	cgd7_1360	0.809	0.902	protein with signal peptide and Sec14d domain, probably secreted
1153	cgd3_1710	0.809	0.889	conserved hypothetical protein
1154	cgd7_2380	0.809	1.031	RRP45-like archaeo-eukaryotic exosomal rnase, PH domain
1155	cgd1_790	0.808	1.031	leucine rich repeats, coiled coil
1156	cgd8_700	0.808	0.999	predicted secreted protein, signal peptide with several threonines, possible mucin
1157	cgd7_2560	0.808	1.015	Sgd1p; NIC plus MI domains containing protein involved in RNA metabolism

1158	cgd8_3280	0.808	0.957	phosphatidylinositol-4-phosphate 5-kinase, putative
1159	cgd2_860	0.808	1.141	Pre3p/proteasome regulatory subunit beta type 6, NTN hydrolase fold
1160	cgd1_600	0.808	1.006	oxoglutarate/malate translocator protein
1161	cgd5_3650	0.807	1.024	hypothetical protein
1162	cgd6_4460	0.806	0.942	large protein with ARM repeats
1163	cgd5_3660	0.806	0.812	hypothetical conserved protein
1164	cgd8_1760	0.805	0.985	secreted protein, signal peptide
1165	cgd5_2980	0.805	0.812	hypothetical protein
1166	cgd7_5450	0.805	1.063	conserved hypothetical protein
1167	cgd2_1190	0.805	0.952	methylase
1168	cgd2_3980	0.804	0.943	Myb domain containing protein
1169	cgd1_3580	0.804	0.917	hypothetical protein
1170	cgd3_4280	0.804	0.967	secreted insulinase like peptidase, signal peptide
1171	cgd3_3230	0.804	0.930	Ser/thr protein kinase
1172	cgd1_300	0.803	0.931	40S ribosomal protein S21
1173	cgd2_1340	0.803	1.103	possible phosphatidylinositol 3- and 4-kinase family protein
1174	cgd6_4070	0.803	0.932	hypothetical protein
1175	cgd1_3270	0.803	0.811	hypothetical protein
1176	cgd7_170	0.803	0.890	hypothetical protein
1177	cgd1_20	0.803	0.892	predicted membrane associated protein with 11 transmembrane domains regions
1178	cgd6_1570	0.802	0.962	apyrase; calcium-activated nucleotidase SCAN-1-like protein; signal peptide
1179	cgd7_4180	0.802	0.945	hypothetical protein
1180	cgd8_4510	0.801	0.897	hypothetical protein
1181	cgd6_5420	0.801	0.843	hypothetical protein, signal peptide
1182	cgd5_1860	0.800	1.002	conserved hypothetical protein
1183	cgd8_3450	0.800	1.011	60S ribosomal protein L17

Cluster 6 **0.5 h: Normal (Ratio = 0.8-1.2) vs. 5 h: Down-regulated (Ratio < 0.8)**

No.	GeneID	Signal Ratio (0.5 h)	Signal Ratio (5 h)	Description
1	cgd8_2160	1.200	0.606	shares a domain with poly(ADP) ribose glycohydrolases, some protein kinase A anchoring proteins and baculovirus HzNV Orf103, possible transmembrane domain within N-terminus
2	cgd7_1260	1.193	0.737	calcium/calmodulin dependent protein kinase with an EF hand N-terminal to the kinase domain and 4 calmodulin like EF hands at the C-terminus
3	cgd4_1460	1.190	0.760	hypothetical protein
4	cgd5_4270	1.180	0.543	hypothetical protein with signal peptide and transmembrane region at C-terminus, possible N-terminal region transmembrane domain
5	cgd5_3410	1.139	0.792	possible phosphate/phosphoenolpyruvate translocator with 9 transmembrane domains
6	cgd6_1800	1.125	0.775	acetyltransferase, GNAT family
7	cgd1_820	1.119	0.606	DHHC family palmitoyl transferases with 4 transmembrane domains
8	cgd6_4430	1.098	0.589	hypothetical protein
9	cgd2_1620	1.096	0.754	hypothetical protein
10	cgd2_4060	1.086	0.587	hypothetical protein with 12 transmembrane domains
11	cgd1_1400	1.069	0.770	hypothetical protein
12	cgd1_260	1.067	0.787	predicted secreted protein, signal peptide
13	cgd5_4470	1.065	0.722	CpTSP7; extracellular membrane associated protein with a signal peptide followed by 2 TSP1 repeats, an EGF domain and a transmembrane region
14	cgd4_2350	1.064	0.752	multitransmembrane protein with signal peptide and GMGPP repeat at C-terminus
15	cgd4_50	1.057	0.736	hypothetical protein
16	cgd4_4320	1.046	0.733	ORC/CDC6 like AAA+ ATpase
17	cgd2_2310	1.044	0.757	Ser/Thr protein kinase
18	cgd6_320	1.040	0.769	conserved hypothetical protein
19	cgd6_2510	1.034	0.728	farnesyltransferase
20	cgd2_3320	1.030	0.778	secreted papain like protease, signal peptide
21	cgd6_2550	1.029	0.737	conserved hypothetical protein
22	cgd8_1610	1.029	0.774	sacsin like HSP90 chaperone domain, likely plant origin
23	cgd4_2060	1.028	0.736	conserved hypothetical protein
24	cgd8_2920	1.025	0.707	Vps60p /Vps20p like protein involved in vacuolar protein sorting
25	cgd1_1710	1.024	0.793	phosphoglycerate mutase
26	cgd4_2100	1.021	0.623	PIG-A like N-acetylglucosaminyl-phosphatidylinositol biosynthetic protein
27	cgd5_2770	1.019	0.758	ARF like small GTpase
28	cgd7_3860	1.017	0.692	conserved hypothetical protein
29	cgd4_960	1.005	0.595	phosphomannomutase
30	cgd6_3620	1.005	0.793	sf-assemblin; Low complexity protein
31	cgd3_320	1.000	0.799	N-myristoyltransferase
32	cgd6_2160	0.994	0.779	hypothetical protein
33	cgd8_3780	0.992	0.787	hypothetical protein

34	cgd8_2970	0.992	0.790	glutathione S-transferase
35	cgd6_5170	0.985	0.714	hypothetical protein
36	cgd6_4880	0.982	0.726	cryptopain - cysteine proteinase secreted, possible transmembrane domain near N-terminus
37	cgd5_280	0.979	0.626	signal peptide containing protein with 6 transmembrane domains
38	cgd6_290	0.979	0.549	hypothetical protein
39	cgd3_3600	0.977	0.756	hypothetical protein
40	cgd5_260	0.974	0.552	hypothetical protein
41	cgd8_1000	0.965	0.643	hypothetical protein
42	cgd7_3030	0.956	0.743	importin/karyopherin
43	cgd6_5470	0.952	0.735	hypothetical protein, signal peptide, transmembrane domain with N-terminus
44	cgd8_2960	0.948	0.754	NIC+MI domains containing protein. nucampholin/yeast Cwc22p like protein involved in mRNA splicing
45	cgd6_4970	0.948	0.747	Hsp60; GroEL-like chaperone (ATpase), predicted mitochondrial
46	cgd5_240	0.941	0.785	G patch domain containing protein
47	cgd7_1720	0.935	0.765	endonuclease III, putative
48	cgd4_350	0.933	0.653	hypothetical protein with 7 transmembrane domains
49	cgd3_4120	0.925	0.800	hypothetical protein
50	cgd8_5020	0.924	0.745	starch binding domain containing protein, possible plant origin
51	cgd5_2890	0.917	0.737	hypothetical protein
52	cgd7_3170	0.915	0.799	thioredoxin fold protein related to phosducin
53	cgd1_2940	0.909	0.673	hypothetical protein
54	cgd3_1470	0.905	0.787	dual specificity phosphatase
55	cgd7_2610	0.895	0.767	POZ+kelch domain protein with kelch repeats at the C-terminus
56	cgd4_4190	0.894	0.758	hypothetical protein with signal peptide
57	cgd6_980	0.890	0.760	SET domain containing protein
58	cgd6_3500	0.874	0.771	hypothetical protein
59	cgd8_5270	0.873	0.770	integral membrane protein predicted amino acid permease, 11x transmembrane domains
60	cgd3_3240	0.867	0.714	hypothetical protein
61	cgd5_4530	0.867	0.757	conserved hypothetical protein
62	cgd3_3280	0.866	0.753	hypothetical protein
63	cgd6_220	0.864	0.636	hypothetical protein, possible G-patch domain
64	cgd2_2520	0.860	0.755	hypothetical protein
65	cgd5_4190	0.859	0.777	hypothetical protein
66	cgd1_3570	0.857	0.798	myosin regulatory light chain
67	cgd6_4020	0.856	0.591	phosphdiesterase, putative
68	cgd6_450	0.840	0.775	hypothetical protein
69	cgd2_1760	0.833	0.758	gata/ArfGAP, putative
70	cgd1_3300	0.825	0.765	ring domain protein
71	cgd7_690	0.823	0.750	small nuclear ribonucleoprotein
72	cgd4_990	0.820	0.692	Ser/Thr protein kinase
73	cgd4_1500	0.815	0.778	hypothetical protein
74	cgd8_720	0.813	0.781	hypothetical protein
75	cgd7_4070	0.803	0.691	arsenical pump-driving ATPase
76	cgd5_4570	0.803	0.784	hypothetical protein
77	cgd7_1060	0.800	0.783	apicomplexan conserved protein with 2 or more transmembrane domains

Cluster 7 — **0.5 h: Down-regulated (Ratio < 0.8) vs. 5 h: Up-regulated (Ratio > 1.2)**

No.	GeneID	Signal Ratio (0.5 h)	Signal Ratio (5 h)	Description
1	cgd3_1490	0.799	1.288	hypothetical protein
2	cgd8_1200	0.790	1.316	E3A like HECT domain containing ubiquitin protein ligase
3	cgd5_560	0.789	1.220	hypothetical protein
4	cgd8_2460	0.752	1.239	hypothetical protein with possible SAP domain
5	cgd7_1240	0.744	1.266	hypothetical protein, transmembrane domain near N-terminus
6	cgd6_2910	0.739	1.483	hypothetical protein with signal peptide
7	cgd5_1090	0.732	1.268	hypothetical protein
8	cgd7_3460	0.715	1.367	hypothetical protein
9	cgd8_3920	0.710	1.275	hypothetical protein, signal peptide, predicted membrane protein, transmembrane domain near C-terminus
10	cgd2_2140	0.627	1.211	hypothetical protein with 14 transmembrane domains
11	cgd8_2760	0.582	1.260	conserved hypothetical protein

Cluster 8 — **0.5 h: Down-regulated (Ratio < 0.8) vs. 5 h: Normal (Ratio = 0.8 - 1.2)**

No.	GeneID	Signal Ratio (0.5 h)	Signal Ratio (5 h)	Description
1	cgd2_3860	0.799	0.958	BBOX Zn finger domain containing protein
2	cgd2_3520	0.797	1.046	conserved hypothetical protein
3	cgd3_4030	0.797	1.033	hypothetical protein with 7 transmembrane domain domain

4	cgd3_240	0.796	0.997	hypothetical protein
5	cgd7_1380	0.796	1.042	conserved hypothetical protein
6	cgd3_190	0.795	1.032	latent transforming growth factor beta binding protein like (3F865)
7	cgd2_540	0.795	0.891	hypothetical protein with 6 transmembrane domains
8	cgd2_2390	0.795	0.997	hypothetical protein with 8 transmembrane domains
9	cgd5_2620	0.794	0.962	penguin protein containing pumolio repeats
10	cgd7_2220	0.794	0.819	hypothetical protein
11	cgd7_5290	0.794	0.853	hedgehog-type HINT domain protein, possible animal horizontal transfer, signal peptide
12	cgd2_3840	0.794	0.818	hypothetical protein
13	cgd6_3450	0.793	0.804	EBNA1 binding protein 2; nucleolar protein p40
14	cgd3_2020	0.792	1.171	PP2C like protein phosphatase
15	cgd5_4220	0.792	0.913	membrane associated protein with a signal peptide, EGF domain and 7 transmembrane domains at the C-terminus
16	cgd4_3630	0.791	0.853	conserved hypothetical protein
17	cgd7_680	0.791	1.095	hypothetical protein with a signal peptide
18	cgd1_3140	0.790	0.898	P-loop nucleotide (UMP) kinase
19	cgd8_2510	0.790	0.828	Cyclin dependent kinase regulatory subunit, putative
20	cgd8_3090	0.790	0.883	proteasome activator p28/ Ki autoantigen
21	cgd3_920	0.789	1.045	calmodulin-domain protein kinase 1, putative
22	cgd1_280	0.788	1.029	short chain dehydrogenase/reductase of the rossmann fold, signal peptide
23	cgd4_3340	0.788	0.945	pleckstrin homology (PH) domain containing protein
24	cgd2_950	0.788	0.828	hypothetical protein
25	cgd2_2040	0.787	0.933	hypothetical protein
26	cgd2_1880	0.787	0.812	protein kinase, putative
27	cgd6_1610	0.787	0.940	hypothetical protein
28	cgd7_1530	0.785	0.893	ART-4 protein; PIN+Zn ribbon domains. involved in RNA metabolism
29	cgd7_1600	0.785	0.829	ribosomal protein of the PO/L10 family
30	cgd7_750	0.784	0.883	hypothetical protein with signal peptide and transmembrane domain near C-terminus
31	cgd6_3380	0.784	0.930	Erf1 eukaryotic translation termination factor 1; N-terminal RNAseH plus pelota domain containing protein
32	cgd5_3120	0.784	0.973	hypothetical protein
33	cgd6_2130	0.784	1.094	hypothetical protein with a C-terminus transmembrane domain
34	cgd8_2530	0.783	0.847	sushi-domain containing secreted protein; with a signal peptide, low complexity region followed by a sushi domain
35	cgd1_1490	0.783	0.960	NEK2 protein, putative
36	cgd8_4750	0.783	0.858	Dbp5p-like eIF4A-1-family RNA SFII helicase
37	cgd2_2540	0.783	1.003	glutaredoxin related protein
38	cgd8_60	0.783	0.931	signal peptide, possible transmembrane domain near N
39	cgd7_1420	0.781	0.868	hypothetical protein
40	cgd5_1040	0.781	1.025	arfgap'arfgap like finger domain containing protein'
41	cgd7_3070	0.780	0.852	hypothetical protein, signal peptide, transmembrane domain near C-terminus
42	cgd8_3140	0.780	0.884	MRP like MinD family ATpase of the SIMIBI class of P-loop GTpases
43	cgd6_950	0.779	0.937	Pdr17p-like Sec14 domain containing protein
44	cgd5_1830	0.779	0.953	F11M21.28-like 3 CCCH RNA binding domain protein involved in RNA metabolism
45	cgd7_5140	0.779	0.868	A surface protein with 2 conserved cysteines
46	cgd1_3240	0.779	0.873	large low complexity protein
47	cgd8_650	0.778	0.812	60S ribosomal protein L23A
48	cgd3_1160	0.778	1.010	conserved hypothetical protein
49	cgd8_550	0.777	0.905	large uncharacterized protein
50	cgd6_3520	0.777	0.933	hypothetical protein
51	cgd3_180	0.776	0.947	domain KOG1666, V-SNARE (intracellular trafficking, secretion, and vesicular transport)
52	cgd6_960	0.775	0.923	cysteinyl-tRNA synthetase
53	cgd5_3960	0.774	0.988	secreted protein of the alpha beta hydrolase superfamily
54	cgd5_3580	0.773	0.808	putative transport protein
55	cgd6_20	0.773	0.869	inosine-5-monophosphate dehydrogenase
56	cgd4_2740	0.772	0.942	IMP4 U3 small nucleolar ribonucleoprotein
57	cgd7_4720	0.772	0.850	hypothetical protein
58	cgd5_2110	0.772	0.860	protein with conserved N-terminal localized cysteine-rich domain; predicted archaeo-eukaryotic ribosomal protein
59	cgd2_10	0.772	0.946	hypothetical protein
60	cgd3_1030	0.772	0.910	uncharacterized low complexity protein
61	cgd5_2750	0.772	1.023	hypothetical protein, signal peptide
62	cgd1_540	0.772	0.888	vacuolar ATP synthase subunit, possible signal peptide
63	cgd4_3440	0.772	1.025	hypothetical protein with signal peptide, within large locus of signal peptide containing proteins
64	cgd5_1490	0.771	1.023	large cysteine rich protein
65	cgd3_440	0.771	0.904	C-type lectin containing protein with a transmembrane domain and mucin-like rich regions
66	cgd2_420	0.771	0.966	signal peptide, serine proline rich, possible low mw mucin glycoprotein locus of 6 genes

67	cgd7_410	0.771	0.985	hypothetical protein
68	cgd4_780	0.770	0.906	DNA topoisomerase II
69	cgd4_4140	0.769	0.814	conserved hypothetical protein
70	cgd6_1080	0.768	1.014	conserved hypothetical protein
71	cgd5_3920	0.768	1.004	calmodulin-like protein
72	cgd8_4530	0.767	0.903	ribosomal processing protein, putative
73	cgd6_5380	0.763	0.970	conserved hypothetical protein
74	cgd3_4080	0.762	1.071	conserved protein with UAS domain, possible ubiquitin protein
75	cgd5_3000	0.761	1.016	hypothetical protein
76	cgd4_1640	0.761	0.968	hypothetical protein
77	cgd1_1630	0.760	0.913	hypothetical protein
78	cgd4_4110	0.760	0.835	hypothetical conserved protein, possible transmembrane domain near C-terminus
79	cgd6_990	0.759	0.895	U1 snrp Snp1p. RRM domain containing protein
80	cgd8_1910	0.758	0.946	epsin like ENTH/VHS domain involved in endocytosis/vesicular trafficking
81	cgd8_4430	0.758	0.900	translation initiation factor if-2 betam beta subunit ZnR
82	cgd6_2920	0.757	0.889	hypothetical protein with signal peptide
83	cgd6_3950	0.756	0.942	uncharacterized protein
84	cgd3_640	0.756	0.852	acyl-CoA synthetase
85	cgd5_700	0.755	0.820	musashi. RRM domain containing protein, splicing related
86	cgd8_3100	0.755	1.010	valyl-tRNA synthetase
87	cgd1_3620	0.754	0.993	transcription factor TAF7p/TAFII55
88	cgd6_180	0.754	0.872	hypothetical protein
89	cgd8_4650	0.754	0.856	hypothetical protein
90	cgd2_690	0.753	0.955	large low complexity protein with glycine-rich repeats
91	cgd4_4400	0.753	0.828	hypothetical protein
92	cgd2_1800	0.753	0.878	heat shock 40 kDa protein, putative
93	cgd1_3170	0.752	0.834	large hypothetical protein, possible coiled coil domain at amino terminus
94	cgd1_190	0.752	0.902	narf-like protein nuclear prelamin A recognition factor
95	cgd8_1670	0.752	0.882	vacuolar ATP synthase subunit B
96	cgd7_3190	0.752	1.008	protein with 3 CCCH domains
97	cgd6_3210	0.751	1.002	Rok1p, eIF4A-1-family RNA SFII helicase
98	cgd6_1230	0.751	0.975	GTpase Grc3p-like Pre-mRNA cleavage complex
99	cgd7_550	0.750	0.849	hypothetical protein wtih 9 transmembrane domains, possible apicomplexan-specific protein
100	cgd4_1890	0.750	0.948	adenosine monophosphate deaminase 2
101	cgd1_460	0.749	1.027	hypothetical protein
102	cgd8_5000	0.749	1.003	hypothetical protein, possible conserved
103	cgd4_3920	0.749	0.809	DinB/family X-type DNA polymerase
104	cgd6_610	0.749	0.801	ATP synthase alpha chain
105	cgd6_1190	0.749	0.912	DNAJ protein-like, putative
106	cgd7_1740	0.749	0.930	Ing1 like PHD finger protein
107	cgd7_3270	0.748	0.919	importin/karyopherin (Arm repeats)
108	cgd4_4200	0.748	0.812	2xTPR domain containing protein
109	cgd3_3840	0.748	0.984	isoleucine-tRNA synthetase
110	cgd4_1620	0.747	0.898	hypothetical protein
111	cgd3_870	0.747	0.948	flavoprotein, putative
112	cgd7_2690	0.745	0.871	conserved hypothetical protein
113	cgd4_3680	0.745	0.881	hypothetical protein
114	cgd2_4120	0.744	1.076	20k cyclophilin, putative
115	cgd2_3120	0.744	0.910	possible transmembrane domain near N
116	cgd3_4230	0.743	0.990	hypothetical protein, signal peptide
117	cgd7_4010	0.743	1.045	hypothetical protein
118	cgd1_3720	0.743	0.879	PIG-M mannosyltransferase,8 transmembrane domain
119	cgd7_2870	0.742	0.928	uncharacterized low complexity protein
120	cgd2_4160	0.740	0.977	hypothetical protein having possible hemolytic activity
121	cgd6_3860	0.736	0.811	SNF2 helicase, putative
122	cgd6_4230	0.736	0.887	hypothetical protein containing a signal peptide
123	cgd4_540	0.736	0.923	putative vacuolar ATP synthase subunit C
124	cgd7_2990	0.735	1.030	hypothetical protein
125	cgd6_1480	0.735	1.038	conserved hypothetical protein
126	cgd5_2930	0.735	0.848	hypothetical protein
127	cgd6_4900	0.735	0.863	acylphosphatase, putative
128	cgd7_980	0.734	0.803	protein with MYND plus SET domains plus 5 Ank repeats
129	cgd1_500	0.734	1.065	hypothetical protein
130	cgd6_1960	0.733	0.889	membrane-associated protein with a family 1 glycosyltransferase domain and a ricin domain with 3 transmembraned domains near C-terminus
131	cgd2_4330	0.733	0.965	hypothetical protein
132	cgd1_690	0.733	0.937	hypothetical protein

133	cgd8_4960	0.731	0.964	hypothetical protein, signal peptide
134	cgd6_2400	0.730	0.913	hypothetical protein
135	cgd6_1930	0.730	0.898	uncharacterized coiled-coil protein
136	cgd2_2170	0.729	0.970	Ynr053p-like, Yjeq GTpase
137	cgd3_610	0.728	0.822	hypothetical protein with possible 4 transmembrane domains
138	cgd2_500	0.728	1.048	hypothetical protein
139	cgd5_2000	0.728	0.901	protein with RIO domain within N-terminal region
140	cgd5_3640	0.728	0.880	hypothetical protein
141	cgd8_3150	0.727	0.818	hypothetical conserved protein
142	cgd6_2300	0.726	1.073	Cut4/Apc1p/TSG24 family protein; meiotic check point regulator and 26S proteasome regulatory complex; PC-rep repeats
143	cgd7_760	0.725	0.888	bruno-like protein with 2 RRM domains
144	cgd5_1840	0.719	0.829	Vir superfamily protein
145	cgd3_3340	0.717	0.870	SECIS binding protein. pelota RNA binding domain containing protein
146	cgd1_640	0.715	0.944	signal peptide, large protein
147	cgd8_630	0.710	0.929	26S proteasome regulatory complex, subunit PSMD5
148	cgd3_1740	0.710	0.949	hypothetical protein with signal peptide and 2 transmembrane domains
149	cgd8_1750	0.710	1.057	signal peptide, possible ER retention motif
150	cgd1_10	0.708	0.902	predicted membrane associated protein with 11 transmembrane domains regions
151	cgd5_3290	0.708	0.859	transcription elongation factor TFIIS
152	cgd3_3380	0.708	0.953	Dbp9p, eIF4A-1-family RNA SFII helicase, DEXDc+HELICc
153	cgd3_3950	0.705	0.862	PUG domain fused to an UBA domain
154	cgd5_4130	0.704	1.052	hypothetical protein having a signal peptide and transmembrane domain near C-terminus
155	cgd1_3070	0.703	0.904	multidomain protein with a conserved eukaryotic domain also present in the human DRIM protein at N-terminus and an archaeal-bacterial domain at C-terminus
156	cgd6_2540	0.703	0.898	tRNA delta(2)-isopentenylpyrophosphate transferase, putative
157	cgd4_3430	0.703	0.889	hypothetical protein with signal peptide and central coiled-coil region, within large locus of signal peptide containing proteins
158	cgd4_4420	0.701	0.877	hypothetical protein
159	cgd4_1370	0.700	0.893	possible ABC transporter with 11 transmembrane domains, AAA domain
160	cgd2_4170	0.699	0.993	conserved hypothetical protein
161	cgd5_2330	0.698	0.947	actin-like
162	cgd3_950	0.697	1.110	hypothetical conserved protein
163	cgd4_1480	0.697	0.944	BMS1 like GTpase involved in ribosome biogenesis
164	cgd6_4830	0.696	0.890	Drs1p, eIF4a-1-family RNA SFII helicase
165	cgd2_800	0.685	0.963	hypothetical protein with 10 transmembrane domains
166	cgd8_5110	0.683	0.877	hypothetical protein
167	cgd6_3980	0.677	0.875	hypothetical protein
168	cgd2_2850	0.676	1.016	hypothetical protein, possible signal peptide
169	cgd7_1030	0.674	0.831	hypothetical protein
170	cgd8_3610	0.672	0.956	predicted integral membrane protein, 7-pass transmembrane domain
171	cgd3_1570	0.665	0.833	sporozoite antigen, putative
172	cgd8_3930	0.658	1.094	LepA like TRAFAC class GTpase, 2 transmembrane domain near C
173	cgd6_3180	0.646	0.895	40S ribosomal protein S15
174	cgd5_1510	0.639	1.060	hypothetical protein
175	cgd6_4090	0.637	1.011	hypothetical low complexity protein
176	cgd3_3270	0.634	0.830	possible nucleotide kinase related to CMP and AMP kinases
177	cgd8_1790	0.625	0.889	hypothetical protein
178	cgd5_840	0.604	0.966	PP2C like phosphatase
179	cgd6_410	0.594	0.946	Sgn1p-like RRM domain containing protein
180	cgd6_1100	0.591	1.040	signal peptide-containing protein
181	cgd7_10	0.577	0.850	hypothetical protein
182	cgd1_3290	0.529	0.965	carboxylesterase, putative
183	cgd7_1290	0.516	1.091	hypothetical protein containing a signal peptide
184	cgd1_1690	0.329	1.117	PHD finger containing protein

Cluster 9 **0.5 h: Down-regulated (Ratio < 0.8) vs. 5 h: Down-regulated (Ratio < 0.8)**

No.	GeneID	Signal Ratio (0.5 h)	Signal Ratio (5 h)	Description
1	cgd7_1620	0.799	0.791	nop15p/nopp34; nucleolar protein with 1 RRM domain
2	cgd2_570	0.795	0.776	tRNA/rRNA methyltransferase-like protein, putative
3	cgd3_3660	0.787	0.779	conserved hypothetical protein
4	cgd8_2110	0.785	0.735	conserved hypothetical protein
5	cgd4_1070	0.782	0.793	MYND finger domain protein
6	cgd5_160	0.780	0.736	hypothetical protein
7	cgd6_1320	0.777	0.773	hypothetical protein, possible transmembrane domain near N-terminus
8	cgd4_840	0.773	0.686	possible 60S ribosomal protein L24

9	cgd4_4210	0.768	0.794	hypothetical protein with signal peptide plus transmembrane domain
10	cgd6_230	0.759	0.693	conserved hypothetical protein
11	cgd1_530	0.750	0.788	hypothetical protein
12	cgd8_2690	0.748	0.799	hypothetical protein
13	cgd7_4200	0.734	0.729	hypothetical protein
14	cgd5_3400	0.726	0.749	mitochondrial processing peptidase beta subunit
15	cgd7_1050	0.717	0.763	hypothetical protein
16	cgd4_1510	0.694	0.651	conserved hypothetical protein
17	cgd4_130	0.689	0.765	RRM domain and KH domain protein (SPAC30D11.14-like KH)
18	cgd5_2990	0.687	0.770	hypothetical protein with transmembrane domain within C-terminal region
19	cgd2_260	0.677	0.758	hypothetical protein
20	cgd5_4540	0.676	0.736	asparagine synthetase A (AsnA) like protein
21	cgd8_4360	0.672	0.746	40S ribosomal protein S15A
22	cgd4_1270	0.665	0.782	transcription factor TFIIIB, SANT domain
23	cgd5_4510	0.640	0.647	hypothetical protein
24	cgd4_3930	0.631	0.767	archeo-eukaryotic exosomal RNAse
25	cgd6_4950	0.627	0.798	hypothetical protein
26	cgd3_3790	0.619	0.790	60S ribosomal protein L19
27	cgd6_4260	0.590	0.776	Low complexity protein
