# Supplementary material for: Transcriptome analysis reveals unique metabolic features in the Cryptosporidium parvum Oocysts associated with environmental survival and stresses
Source: BMC Genomics. 2012 Nov 21;13:647. doi: 10.1186/1471-2164-13-647 (PMC3542205; doi:10.1186/1471-2164-13-647)

Figure S4.
Distribution of regulated genes into 9 clusters based on the expression dynamics in the UV-irradiated oocysts after 0.5 h and 5 h of recovery times . Fold changes in gene expression are shown as ratios of normalized median signals between treated and control groups (T/C).

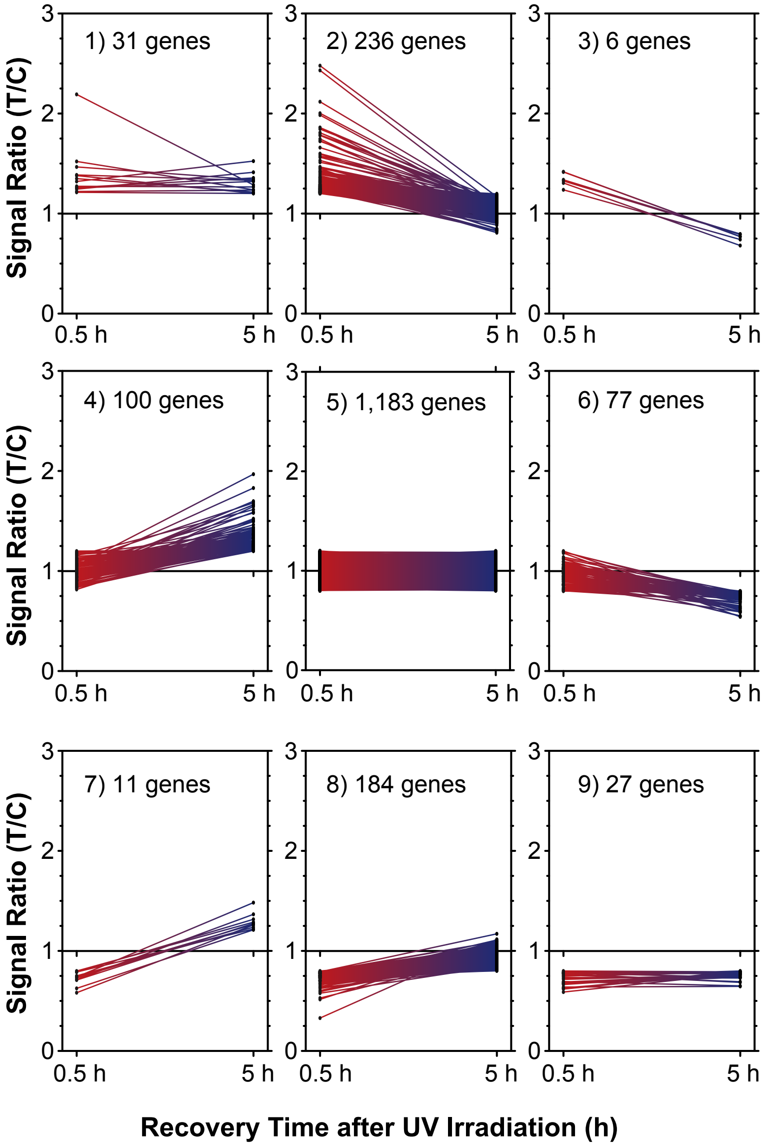

Supplement: Additional file 7 — Figure S4. Distribution of regulated genes into 9 clusters based on the expression dynamics in the UV-irradiated oocysts after 0.5 h and 5 h of recovery times. Fold changes in gene expression are shown as ratios of normalized median signals between treated and control groups (T/C). [file 1471-2164-13-647-S7.pdf]
