# Supplementary material for: Transcriptome analysis reveals unique metabolic features in the Cryptosporidium parvum Oocysts associated with environmental survival and stresses
Source: BMC Genomics. 2012 Nov 21;13:647. doi: 10.1186/1471-2164-13-647 (PMC3542205; doi:10.1186/1471-2164-13-647)
Supplement: Additional file 8 — Table S4. List of primers used in this study. [file 1471-2164-13-647-S8.pdf]

Table S4. List of primers used in this study

Gene ID		Sequence (5'-3')
cgd7_480	forward	TGGTGGCCATGGCGATGGTATG
	reverse	AGCAGCGGCTGGTGCAAAGT
cgd3_1570	forward	TGACGGAGTTTCAGAGGCTGCT
	reverse	TTCCTCCGACCCAAACGCCG
cgd3_3770	forward	CGATGGCAAGAAGTTGAGATGCTGT
	reverse	AGGCTTCCTTCTCCTCAGCAGT
cgd1_880	forward	TGGGCAAGCTCAATCTGGTACTGGT
	reverse	ACCACCAACGCAAGCATGGCA
cgd5_3160	forward	TGGTCAAGGCAGGTGTTGCAGG
	reverse	ACACCTGGCATTTTTGGGCGAC
cgd2_20	forward	TGGTGATGCAGCAAAGAACCAAGT
	reverse	TGATTGGCTTGTCCTTTGGACCTCT
cgd2_2540	forward	GGGAGCTAACTGGAAGTGGAACTGT
	reverse	GCCGTCTTCTAACAGCTTCAGGGT
cgd3_3850	forward	TGGCGGTAAGTCAGCAGTGAGT
	reverse	GTCCCTGCCTCACATTTTGCCCA
cgd1_2770	forward	AGGGGGCCCTTCCAATGCAAC
	reverse	AGCCCCATCTCCTCCCCCTC
cgd7_360	forward	ACTCGCTCAGAAACTCGAGGAGGA
	reverse	CTGCATGACCAGCAGCGCCT
cgd4_3400	forward	AGGCCCAGACGGAGTAGCACAT
	reverse	TCGCCTCCATCTTCTGGCCTT
cgd7_3430	forward	ACGCTGTGCTTCTCTATCGCCA
	reverse	GCGCATTCAACCCATCACGCC
cgd8_580	forward	GGGCGTGTACTATTTTTGGTCCTCC
	reverse	GGGCTGACAAATCCATGCCCA
cgd3_3750	forward	GCTCACTCAGGCTCAACTTGCACA
	reverse	TCTTTGGGGCGGGCTTTGGT
cgd5_1290	forward	AGCTGCGGTAGCTGCGTCTTT
	reverse	GCATCCAATGTTCCCAATCAGGACG
cgd8_1970	forward	CAACACCCTCTGGATCTACGGCA
	reverse	CCAAAGCAACCTGGACGCCAT
cgd7_3170	forward	TGCCACTTCTTCAGGCCATCAACT
	reverse	ACAAACAAAGTGCGATTTTTCCGCA
cgd4_780	forward	TCCTCTTCAGGAGGGGTTCCTGC
	reverse	TCTGACTTCCAGATTTCGCTGTCCT
cgd2_1450	forward	CGGTGGTCCGGACTCGTTCA
	reverse	TGCGATGGCTGATGCGAGTTGT
cgd2_3180	forward	GGCCCTTCAGGGGGAGGGAAA
	reverse	CTGCATATGATGGGGGCTTTGGCA
cgd2_3200	forward	TGGGATTGGGTTGGTGGTAGGT
	reverse	ATTCCTGCACGAGGTTGGCG
cgd7_910	forward	TGCTAATGGCCCTCCAGGTGT
	reverse	AGCACCTCCGCCTGTAGAAACA
cgd1_3710	forward	GGCCCTGTGGCAACACCTGA
	reverse	ATTGTGCCCAGGATGACTCAATGG
cgd8_3510	forward	GCCACTTCTTACAGTATAGG

	reverse	ACATTCTTGAACTTGACCAT
cgd7_4710	forward	AACCAAGGTACTTCGTCCCCGA
	reverse	GCCGTTCTCCAATGATAGCCCCA
cgd8_3250	forward	AATGGTGTCTGAGGTGTT
	reverse	ATACGCCTGTGTAGTGAATA
cgd6_2060	forward	TCGTGCACAGGCCACATCCA
	reverse	ACCTCCGGAGCCAGATGCACA
cgd4_2260	forward	AGGAACTGCGTTGCGCTTGGT
	reverse	GGGGCAGACGCGCCAACAG
cgd5_3490	forward	CCTCGCAAGTGCTACTCCACG
	reverse	TGCAGCTCCACTTCCGTGCT
cgd7_4110	forward	TCCACTGGCCAAGTTGCAGCC
	reverse	ACAACTCCATCGCTGTGCCCT
cgd8_4330	forward	GGTCGGCCCGGAAGGTAGTGT
	reverse	TGGTCAGGCTGGGCAACATCA
cgd5_2060	forward	ACTCGCCCTCGCCTTTGTCA
	reverse	TGTCGGCGACTGTTGGTGAAAGT
cgd7_4470	forward	ACACTGCAAAGCAGGACTTGGACG
	reverse	AGGGAGGATTGAGCCTCTCGCA
cgd3_2540	forward	TGGCAAGGACCAAACAGACAGCG
	reverse	AGGAGCTGATTTCCTTGCGCCC
cgd5_1960	forward	AGAAAGGCGCATGCAACGCT
	reverse	CCCCAGCCAAACGATCTTGCGA
cgd5_3160	forward	TGGTCAAGGCAGGTGTTGCAGG
	reverse	ACACCTGGCATTTTTGGGCGAC
cgd7_940	forward	TGGTGGAAGGAGACAAGGTGGCT
	reverse	TGCTTCCGCCTCTGTATGGAGACC
cgd8_5000	forward	TGGAGGACCATTCAGAACAGAGTGT
	reverse	ACAAACGGTTCCTCCTGTCACCT
cgd6_4900	forward	TGGCGAGTTGGAGGGAGACGA
	reverse	TGTGGAACCGGGACTACCTTCCC
cgd5_2000	forward	ACCAGGCTAGAGCTAGCGATGAA
	reverse	AGCATCGTCCGCCCATCAAGC
cgd5_3290	forward	GCTCTTGAGCAAGGCTTGGTGG
	reverse	TCATCAATTCAAACCTCCTCTGGCG
cgd3_1570	forward	TGACGGAGTTTCAGAGGCTGCT
	reverse	TTCCTCCGACCCAAACGCCG
cgd6_4950	forward	AGCATCAGCAACATTTGCAAAGCCA
	reverse	CCACTTCCCTTGCTGTTGTTGAAGC
cgd3_1400	forward	TGTTTACCTCATCAGACTGGGAGAC
	reverse	ATGGAAGATTTTGACTGGCTGTTC
cgd7_480	forward	AAGCAAGGTCTTATCACCCAG
	reverse	GCAAAGTAGGCAGTTCCTGTC
cgd7_470	forward	GTATTTACAAAACTCTGATGTCGTC
	reverse	GCATTAGGGCAGTATTTACCG
cgd6_3800	forward	AGCAGCATCTCTTGTCTCAGC
	reverse	TGATCCATCTATGGCAATGGT
cgd7_7	forward	TTGTTCCTTACTCCTTCAGCAC
	reverse	TCCTTCCTATGTCTGGACCTG
